# Combining search filters for randomized controlled trials with the Cochrane RCT Classifier in Covidence: a methodological validation study

**DOI:** 10.1017/rsm.2025.10023

**Published:** 2025-08-28

**Authors:** Klas Moberg, Carl Gornitzki

**Affiliations:** Swedish Agency for Health Technology Assessment and Assessment of Social Services (SBU), Stockholm, Sweden

**Keywords:** literature searching, machine learning, randomized controlled trials, search filters, study classifiers, systematic review software

## Abstract

Our objective was to evaluate the recall and number needed to read (NNR) for the Cochrane RCT Classifier compared to and in combination with established search filters developed for Ovid MEDLINE and Embase.com. A gold standard set of 1,103 randomized controlled trials (RCTs) was created to calculate recall for the Cochrane RCT Classifier in Covidence, the Cochrane sensitivity-maximizing RCT filter in Ovid MEDLINE and the Cochrane Embase RCT filter for Embase.com. In addition, the classifier and the filters were validated in three case studies using reports from the Swedish Agency for Health Technology Assessment and Assessment of Social Services to assess impact on search results and NNR. The Cochrane RCT Classifier had the highest recall with 99.64% followed by the Cochrane sensitivity-maximizing RCT filter in Ovid MEDLINE with 98.73% and the Cochrane Embase RCT filter with 98.46%. However, the Cochrane RCT Classifier had a higher NNR than the RCT filters in all case studies. Combining the RCT filters with the Cochrane RCT Classifier reduced NNR compared to using the RCT filters alone while achieving a recall of 98.46% for the Ovid MEDLINE/RCT Classifier combination and 98.28% for the Embase/RCT Classifier combination. In conclusion, we found that the Cochrane RCT Classifier in Covidence has a higher recall than established search filters but also a higher NNR. Thus, using the Cochrane RCT Classifier instead of current state-of-the-art RCT filters would lead to an increased workload in the screening process. A viable option with a lower NNR than RCT filters, at the cost of a slight decrease in recall, is to combine the Cochrane RCT Classifier with RCT filters in database searches.

## Highlights

### What is already known?

- The Cochrane RCT Classifier is a useful tool for automatically identifying possible RCTs and non-RCTs with a 99.5% recall.

### What is new?

- The Cochrane RCT Classifier in Covidence does not reduce the number of hits retrieved in database searches to the same extent as existing state-of-the-art RCT filters in Ovid MEDLINE and Embase.com.
- Using RCT filters in Ovid MEDLINE and Embase.com prior to the application of the Cochrane RCT Classifier in Covidence reduces the number of hits to screen while maintaining a high recall.

### Potential impact for RSM readers

- A reduced workload with a slight decrease in recall can be achieved in the title and abstract screening process by combining the use of RCT filters in Ovid MEDLINE and Embase.com with the Cochrane RCT Classifier in Covidence.

## 1 Introduction

Randomized controlled trials (RCTs) are the standard study design for inclusion in systematic reviews on effects of health care interventions. For this purpose, RCT search filters have been developed and validated^1^ ^–^ ^5^ for efficient information retrieval.

In recent years, machine classifiers have been developed to increase efficiency in the abstract screening process. These classifiers are trained on data sets of title and abstract records meeting specific criteria using supervised machine learning. One such tool is the Cochrane RCT Classifier, originally built as a part of the Cochrane evidence pipeline with a purpose to populate the Cochrane CENTRAL database.^6^ It has also been used as one of three components in the Cochrane Screen4Me workflow to aid authors of Cochrane reviews by distinguishing RCTs from non-RCTs.^6^ ^–^ ^9^ In addition, the Cochrane RCT Classifier is integrated in EPPI-Reviewer^10^ and Covidence.^11^

In this study, we evaluate the performance of the Cochrane RCT Classifier in Covidence. This tool enables the automatic tagging of references as either being a “Possible RCT” or “Not RCT” and facilitates the removal of references reporting on non-RCTs before screening. References marked as non-RCTs can be screened separately allowing the user to move individual references back to the screening workflow. This feature can be disabled at any point moving references marked as non-RCTs back to the title and abstract screening step for manual screening.^12^

To the best of our knowledge, no validation study evaluating the combination of search filters and the Cochrane RCT Classifier has been published. Therefore, the aim of this study is to evaluate the recall (also known as “sensitivity”), number needed to read (NNR) and number of hits to screen for the Cochrane RCT Classifier compared to and in combination with established search filters developed for the bibliographic databases Ovid MEDLINE and Embase.com.

For this purpose, we identified RCTs from a repository of included studies at the Swedish Agency for Health Technology Assessment and Assessment of Social Services (SBU) to generate a gold standard set (GS). The repository is populated with bibliographic references from tables of included studies for all SBU reports from year 2019 onwards with SBU specific metadata such as information on study type and risk of bias. As of April 2025, the repository contains more than 6,000 references, including 1,249 manually classified RCT studies, and is continuously updated.

## 2 Methods

### 2.1 Generating the gold standard set

The relative recall method was used to identify RCTs to include in the GS. This method involves the collection of references included in evidence syntheses on a specific topic to calculate the recall of search filters.^13^ The GS was populated with RCTs (*n* = 1,249) from all 47 SBU reports published between 2019 and 2024 that has included RCTs (see Appendix 1 of the Supplementary Material for the SBU reports). The number of RCTs included in each report ranges from 1 to 139. One hundred forty-six references were then excluded to achieve the exact same GS, including the same set of references, for both Ovid MEDLINE and Embase.com. The final GS contains 1,103 RCTs published between 1970 and 2024 (see Appendix 2 of the Supplementary Material for the GS references). Approximately, 20% of the GS was identified in reports searching without a filter for study design, 30% with an RCT filter and 50% with a broader search filter for clinical trials.

### 2.2 Testing RCT Classifier and search filter recall

We validated the Cochrane RCT Classifier in Covidence, the Cochrane sensitivity-maximizing RCT filter in Ovid MEDLINE^14^ and the Cochrane Embase RCT filter for Embase.com^3^ using recall, that is, the proportion of studies correctly identified as relevant relative to the total number of relevant studies.^15^ The search filters were tested in Ovid MEDLINE and Embase.com by first running the search filter and then adding a search line for the GS with the PubMed identifiers (PMID) and Embase identification numbers for these records. The searches were then compared using the NOT Boolean operator to detect references in the GS not identified by the search filters. The Cochrane RCT Classifier was tested by exporting the GS to Covidence from the EndNote software, using the RefMan (RIS) Export format, to identify the number of references labeled as either “Possible RCT” or “Not RCT.” Recall was then calculated as: (number of gold standard records identified by a search filter, the RCT Classifier or a combination of both/total number of gold standard records) × 100 to express as a percentage.^15^

### 2.3 Case study

To establish the efficiency of the Cochrane RCT Classifier in Covidence compared to and in combination with the Cochrane sensitivity-maximizing RCT filter in Ovid MEDLINE and the Cochrane Embase RCT filter for Embase.com, we conducted a case study. We reran the original search strategies from three SBU reports in Ovid MEDLINE (see Appendix 3 of the Supplementary Material for the Ovid MEDLINE search strategies) and Embase.com (see Appendix 4 of the Supplementary Material for the Embase.com search strategies) in January 2025, using date limits matching the search period from the original searches. Search results with and without RCT filters were documented and exported separately from Ovid MEDLINE, using the EndNote export format, and from Embase.com, using the RIS format. Number of included references retrieved by each individual search was recorded in order to be able to calculate recall. Search results were then imported into empty Covidence libraries to run the Cochrane RCT Classifier. The selection of the SBU reports was based on the topical coverage of the organization and for the purposes of illustrating the impact on searches of varied size. The topics of the three reports are listed below:

- SBU Assessment 337: Internet-delivered psychological treatment versus other available treatment options for common mental disorders .^16^
- SBU Assessment 372: Treatment and social support for adults with co-occurring addictive and psychiatric disorders.^17^
- SBU Policy support 379: Treatment and rehabilitation of post-COVID-19 and other post-infectious conditions.^18^

NNR, the number of studies a researcher must read to identify a relevant study,^19^ was used to assess the impact on screening workload. NNR was calculated as: 1/Precision, with precision equal to the proportion of references retrieved by a filter or classifier that are relevant: (Number of gold standard records identified by a search filter, the RCT Classifier or a combination of both/Number of records retrieved in a retrospective search).^19^

## 3 Results

### 3.1 RCT Classifier and search filter recall

The Cochrane RCT Classifier in Covidence had the highest recall with 99.64% (1,099 out of 1,103 references). Three out of four missed references were indexed as RCTs in Ovid MEDLINE and therefore identified by the RCT filter. Two out of four missed references were indexed as RCTs in Embase.com and identified by the Cochrane Embase RCT filter for Embase.com. All four missed references lacked terms related to the RCT study design in the titles and abstracts. The Cochrane sensitivity-maximizing RCT filter in Ovid MEDLINE reached a recall of 98.73% and the Cochrane Embase RCT filter 98.46% missing 14 and 17 references, respectively. When combining the Cochrane RCT Classifier with RCT filters in Ovid MEDLINE and Embase.com, there was a slight decrease in recall in comparison to using the RCT filters alone (Ovid MEDLINE: 98.46%, Embase.com: 98.28%) missing three and two additional references, respectively (Table 1).

**Table 1:** Validation of recall with the entire gold standard (1,103 articles)

	Recall	No of missed RCTs
Cochrane RCT Classifier in Covidence	99.64%	4
Cochrane sensitivity-maximizing RCT filter in Ovid MEDLINE	98.73%	14
Cochrane Embase RCT filter for Embase.com	98.46%	17
MEDLINE RCT filter AND RCT classifier combined	98.46%	17
Embase RCT filter AND RCT classifier combined	98.28%	19

### 3.2 Case study

In the case study, the Cochrane RCT Classifier in Covidence and the RCT filters all achieved 100% recall for the included studies found by the original search strategies (Table 2). The Cochrane RCT Classifier in Covidence had a higher NNR than the RCT filters in all case studies, producing an average of 34% more references to screen. The lowest NNR was achieved when combining the Cochrane RCT Classifier with RCT filters in Ovid MEDLINE and Embase.com. The RCT classifier and RCT filter combination reduced NNR compared to when using RCT filters alone: For SBU Assessment 337, NNR was reduced from 263 to 219 in Ovid MEDLINE and from 270 to 236 in Embase.com. For SBU Assessment 372, NNR was reduced from 284 to 132 in Ovid MEDLINE and from 172 to 78 in Embase.com. For SBU Policy support 379, NNR was reduced from 77 to 34 in Ovid MEDLINE and from 81 to 34 in Embase.com. The RCT classifier and RCT filter combination reduced the number of references to screen with more than 50% compared to using RCT filters alone in two out of three case studies while the decreased screening burden remained at 13–17% in one case study (Table 2).

**Table 2:** Results of the case studies

	Original search strategy											
		without RCT									With RCT FILTER		
		FILTER/RCT CLASSIFIER			With RCT FILTER			With RCT CLASSIFIER			and RCT CLASSIFIER		
						Recall of			Recall of			Recall of	
					included			included			included	
					references			references			references	
		No. of			found by			found by			found by	
	No. of	included		No. of	original		No. of	original		No. of	original	
	search	references		search	search		search	search		search	search	
	hits	retrieved	NNR	hits	strategy	NNR	hits	strategy	NNR	hits	strategy	NNR
SBU Assessment 337: Internet-delivered psychological treatment versus other available treatment options for common mental disorders												
Ovid MEDLINE	10,083	20	504	5,264	100%	263	6,340	100%	317	4,373	100%	219
Embase.com	11,603	17	683	4,597	100%	270	7,356	100%	433	4,013	100%	236

SBU Assessment 372: Treatment and social support for adults with co-occurring addictive and psychiatric disorders												
Ovid MEDLINE	91,324	88	1,038	25,016	100%	284	33,817	100%	384	11,609	100%	132
Embase.com	71,396	77	927	13,274	100%	172	19,703	100%	256	6,040	100%	78

SBU Policy support 379: Treatment and rehabilitation of post-COVID–19 and other post-infectious conditions												
Ovid MEDLINE	16,149	39	414	2,993	100%	77	4,836	100%	124	1,337	100%	34
Embase.com	18,131	36	504	2,914	100%	81	6,021	100%	167	1,230	100%	34

## 4 Discussion

This study shows that the Cochrane RCT Classifier has a higher recall (99.64%) than state-of the-art RCT filters in Ovid MEDLINE (98.46%) and Embase.com (98.28%). However, at the cost of an average of 34% more references to screen than the validated search filters. An alternative is to combine searching with RCT filters and using the Cochrane RCT Classifier. In our study, this combination reduced NNR while achieving a recall comparable to using search filters in Ovid MEDLINE and Embase.com missing only three and two extra references, respectively.

The lack in overlap in missed RCTs between the Cochrane RCT Classifier and the search filters is due to the fact that none of the four studies not identified by the Cochrane RCT Classifier contained terms related to the RCT study design in titles or abstracts. However, three out of four missed references were indexed as RCTs in either MEDLINE or Embase. In other words, three out of four missed references would have been identified by the Cochrane RCT Classifier if it incorporated metadata from databases describing study design (e.g., Publication Type in MEDLINE). In a previous work on an RCT classifier, the highest recall was achieved when metadata for study design was incorporated in a machine classifier.^7^ However, for the purposes of creating the Cochrane RCT Classifier with a main focus on new records, usually lacking metadata about study design, a model was preferred that uses titles and abstract text without additional metadata.^6^

There is no universal definition of an acceptable level of recall for a search filter or a machine classifier. Considering recall, the best option is not to use a search filter or machine classifier at all since every limiting concept added to a search increases the risk of missing relevant studies. Nevertheless, 90%^20^ and 95%,^21^ respectively, have been suggested as thresholds in previous filter validation studies, numbers that can be compared to 99% which was the target recall used when creating the Cochrane RCT Classifier.^6^ The results from our study can thus, based on their idea of an acceptable level of recall, assist review authors decisions on choosing RCT-filters, the Cochrane RCT Classifier or a combination of the two.

An important aspect to consider when using the Cochrane RCT Classifier is quality of metadata. For instance, we discovered that during export of references from Ovid MEDLINE to Covidence there were minor differences in performance of the Cochrane RCT Classifier depending on the export format used. Using the RIS export format, special characters appeared in some abstracts causing a slight decrease in recall as opposed to when using the EndNote format. It should be stressed that the RIS export format in Ovid MEDLINE is not identical to the RefMan (RIS) Export format in EndNote, using the latter does not cause any changes in the performance of the Cochrane RCT Classifier. Another metadata issue concerns short titles and presence or absence of abstracts. In the training, calibration, and validation of the Cochrane RCT Classifier references with less than 15 characters in the title or less than 400 characters in the abstract where excluded and in a secondary analysis of the validation recall was reduced to 94% when records with limited information in their titles and/or abstracts were included.^6^ This issue is resolved in the Cochrane RCT Classifier in Covidence by only processing references that meet the minimum text requirements mentioned above leaving references that do not meet these thresholds to manual screening.^12^

Systematic review handbooks have started to mention the use of machine classifiers in general as well as the Cochrane RCT Classifier specifically. The IQWiG General Methods handbook declares that validated search filters or machine classifiers are used if available.^22^ NICE states in their handbook for developing guidelines that they support the use of machine classifiers if they improve efficiency in the search and screening process and their performance characteristics are known.^23^ Neither IQWiG nor NICE comment on the possible combination of search filters and machine classifiers. The Cochrane Handbook acknowledges the use of study design classifiers in general but underlines that they should probably not be used in combination with study design filters.^24^

This study provides validation data demonstrating a slight decrease in recall when the Cochrane RCT Classifier in Covidence is combined with an RCT filter as opposed to using an RCT filter alone, while reducing the burden of screening references by more than 50% in two out of three case studies.

### 4.1 Study limitations

The Cochrane RCT Classifier is currently available in Cochrane Screen4Me, EPPI-Reviewer, and Covidence. Due to lack of access, we have only evaluated it in Covidence. A head-to-head comparison would provide valuable information about the performance of the Cochrane RCT Classifier in different systematic review software tools.

The GS was developed using the relative recall method. A potential limitation of this method is that it is dependent on the quality of the individual searches used to create the GS.^13^ Ideally, the GS would consist of references identified in searches without an RCT filter, otherwise it could be argued that the tested filters perform better because they were, in part, used to create the GS. The GS in this study does not exclusively contain references identified in searches without an RCT search filter. Approximately, 20% of the GS was identified in reports searching without a filter for study design, 30% with an RCT filter and 50% with a broader search filter for clinical trials. The share of references in the GS identified in searches without an RCT search filter is comparable to a much larger RCT filter validation study published in 2020.^4^

Finally, the majority of SBU reports used to populate the GS address issues within the health care domain making the results most applicable to this context. Furthermore, the reduction in NNR when combining the Cochrane RCT Classifier with RCT filters was more significant in two out of the three case studies. A more comprehensive evaluation including a larger set of case studies on different topics would provide more conclusive data on possible screening workload reductions.

## 5 Conclusions

The Cochrane RCT Classifier in Covidence has a higher recall than established search filters but also a higher NNR. Thus, using the Cochrane RCT Classifier instead of current state-of-the-art RCT filters would lead to an increased workload in the screening process. A viable option with a lower NNR than the RCT filters, at the cost of a slight decrease in recall, is to combine the Cochrane RCT Classifier with RCT filters in database searches. Larger evaluations of the combination of RCT filters and the Cochrane RCT Classifier are needed to further investigate time and resource savings and impact on recall.

## Supporting information

Moberg and Gornitzki supplementary material Moberg and Gornitzki supplementary material

## Data Availability

Data available within the article or its Supplementary Material (Appendices 1–4).

## Supplementary material

To view supplementary material for this article, please visit http://doi.org/10.1017/rsm.2025.10023.
