# Supplementary material for: Combining search filters for randomized controlled trials with the Cochrane RCT Classifier in Covidence: a methodological validation study
Source: Res Synth Methods. 2025 Aug 28;16(6):953–60. doi: 10.1017/rsm.2025.10023 (PMC12657657; doi:10.1017/rsm.2025.10023)
Supplement: Moberg and Gornitzki supplementary material [file S1759287925100239sup001.zip › Appendix S4.docx]

**Appendix S4**

**Case study: Embase.com search strategies**

*SBU Assessment 337: Internet-delivered psychological treatment versus other available treatment options for common mental disorders*

| **#** | **Searches** |
| --- | --- |
| 1 | 'adjustment disorder'/de |
| 2 | 'anxiety'/exp |
| 3 | 'anxiety disorder'/exp |
| 4 | 'body dysmorphic disorder'/de |
| 5 | 'depression'/de OR 'adolescent depression'/de OR 'agitated depression'/de OR 'atypical depression'/de OR 'chronic depression'/de OR 'depressive psychosis'/de OR 'dysphoria'/de OR 'dysthymia'/de OR 'endogenous depression'/de OR 'involutional depression'/de OR 'late life depression'/de OR 'major depression'/de OR 'masked depression'/de OR 'melancholia'/de OR 'minor depression'/de OR 'mixed anxiety and depression'/de OR 'mixed depression and dementia'/de OR 'mourning syndrome'/de OR 'organic depression'/de OR 'perinatal depression'/exp OR 'perry syndrome'/de OR 'post-stroke depression'/de OR 'postoperative depression'/de OR 'premenstrual dysphoric disorder'/de OR 'pseudodementia'/de OR 'reactive depression'/de OR 'recurrent brief depression'/de OR 'seasonal affective disorder'/de OR 'subsyndromal depression'/de OR 'treatment resistant depression'/de |
| 6 | 'hoarding'/de |
| 7 | 'hypochondriasis'/de |
| 8 | 'mood disorder'/de |
| 9 | 'major affective disorder'/de |
| 10 | 'minor affective disorder'/de |
| 11 | 'mutism'/exp |
| 12 | 'premenstrual syndrome'/de |
| 13 | 'psychotrauma'/exp |
| 14 | 'psychosocial disorder'/de |
| 15 | 'trichotillomania'/de |
| 16 | 'dermatillomania'/exp |
| 17 | 'disinhibited social engagement disorder'/de |
| 18 | 'disruptive mood dysregulation disorder'/de |
| 19 | 'excoriation disorder'/de |
| 20 | 'olfactory reference syndrome'/de |
| 21 | 'prolonged grief'/exp |
| 22 | acrophobi*:ti,kw OR 'acute stress':ti,kw OR 'adjustment disorder*':ti,kw OR 'adjustment react*':ti,kw OR aerophobi*:ti,kw OR 'affective disorder*':ti,kw OR agoraphobi*:ti,kw OR anxiety*:ti,kw OR anxieties:ti,kw OR anxiodepressive:ti,kw OR anxious*:ti,kw OR arachnophobi*:ti,kw OR 'attachment disorder*':ti,kw OR aviophobi*:ti,kw OR 'body dysmorphic disorder*':ti,kw OR 'body image disfunction*':ti,kw OR 'body image disorder*':ti,kw OR dentophobi*:ti,kw OR depressed:ti,kw OR depression:ti,kw OR depressive:ti,kw OR depressiveness:ti,kw OR depressivity:ti,kw OR dermatillomani*:ti,kw OR 'disinhibited social engagement disorder*':ti,kw OR 'disruptive mood dysregulation disorder':ti,kw OR dysmorphophobi*:ti,kw OR dysphoria:ti,kw OR 'dysphori* disorder*':ti,kw OR 'dysphori* syndrom*':ti,kw OR dysthymi*:ti,kw OR emetophobi*:ti,kw OR 'excoriation disorder*':ti,kw OR gad:ti,kw OR glossophobi*:ti,kw OR 'hair pulling':ti,kw OR hoarding:ti,kw OR hypochondri*:ti,kw OR mdd:ti,kw OR melanchol*:ti,kw OR 'mood disorder*':ti,kw OR mute:ti,kw OR mutism:ti,kw OR 'obsessive compulsive':ti,kw OR ocd:ti,kw OR odontophobi*:ti,kw OR 'olfactory reference syndrome*':ti,kw OR 'olfactory reference disorder*':ti,kw OR ophidiophobi*:ti,kw OR panic:ti,kw OR phobi*:ti,kw OR (((posttrauma* OR 'post trauma*') NEAR/3 (disorder* OR neuros* OR psych* OR stress* OR symptom* OR syndrom*)):ti,kw) OR 'premenstrual syndrom*':ti,kw OR 'prolonged grief disorder*':ti,kw OR ptsd:ti,kw OR 'reactive disorder*':ti,kw OR 'skin picking':ti,kw OR 'transient situational disturbance':ti,kw OR 'traumatic stress':ti,kw OR trichotillomani*:ti,kw |
| 23 | #1 OR #2 OR #3 OR #4 OR #5 OR #6 OR #7 OR #8 OR #9 OR #10 OR #11 OR #12 OR #13 OR #14 OR #15 OR #16 OR #17 OR #18 OR #19 OR #20 OR #21 OR #22 |
| 24 | 'web-based intervention'/de |
| 25 | 'teleconsultation'/de OR 'e-counseling'/de OR 'self-care software'/de OR 'therapy software'/de OR 'cognitive therapy software'/de |
| 26 | 'telemedicine'/de |
| 27 | 'telepsychiatry'/de |
| 28 | 'teletherapy'/de |
| 29 | 'computer assisted therapy'/de |
| 30 | 'digital health'/de |
| 31 | 'telehealth'/de |
| 32 | 'mhealth'/de |
| 33 | ccbt:ti,ab,kw OR 'c cbt':ti,ab,kw OR 'cyber counseling':ti,ab,kw OR 'cyber counselling':ti,ab,kw OR cybercounseling:ti,ab,kw OR cybercounselling:ti,ab,kw OR 'digital health':ti,ab,kw OR 'e consultation':ti,ab,kw OR ecbt:ti,ab,kw OR 'e cbt':ti,ab,kw OR econsultation:ti,ab,kw OR 'e counseling':ti,ab,kw OR 'e counselling':ti,ab,kw OR ecounseling:ti,ab,kw OR ecounselling:ti,ab,kw OR 'e health':ti,ab,kw OR ehealth:ti,ab,kw OR emedicine:ti,ab,kw OR 'e medicine':ti,ab,kw OR 'emental health*':ti,ab,kw OR 'e-mental health*':ti,ab,kw OR 'e portal':ti,ab,kw OR eportal:ti,ab,kw OR epsych*:ti,ab,kw OR 'e psych*':ti,ab,kw OR 'e therapy':ti,ab,kw OR etherapy:ti,ab,kw OR 'i cbt':ti,ab,kw OR icbt:ti,ab,kw OR 'm health':ti,ab,kw OR mhealth:ti,ab,kw OR 'mobile health':ti,ab,kw OR deprexis:ti,ab,kw OR interapy:ti,ab,kw OR wechat*:ti,ab,kw |
| 34 | ((android OR app OR apps OR blackberry OR cellphone OR 'cell* phone*' OR 'mobile phone*' OR 'smart phone*' OR chat* OR computer* OR cyber* OR digital OR 'electronic mail*' OR 'e mail' OR email OR 'handheld device*' OR 'instant messag*' OR internet* OR ipad* OR 'i pad*' OR iphone* OR 'i phone*' OR messaging OR 'mobile application*' OR online* OR 'on line*' OR 'personal digital assistant*' OR 'short messag*' OR smartphone* OR sms OR tablet* OR 'text messag*' OR texting OR webbased OR webdeliver* OR 'web based' OR 'web deliver*' OR website*) NEAR/10 (act OR 'attention modification' OR 'attention bias modification' OR 'attention training' OR 'behavio* activat*' OR 'behavio* component$' OR 'behavio* guidance' OR 'behavio* management' OR 'behavio* modif*' OR 'behavio* rehab*' OR 'behavio* restructur*' OR 'behavio* technique*' OR 'behavio* train*' OR 'cognitive activat*' OR 'cognitive defusion' OR 'cognitive guidance' OR 'cognitive management' OR 'cognitive modif*' OR 'cognitive rehab*' OR 'cognitive restructur*' OR 'cognitive technique*' OR 'cognitive train*' OR cbt OR ccbt OR 'cognitive behavio*' OR consultation* OR counceling OR councelling OR dbt OR 'dialectical behavio*' OR hypnosis OR hypnotherapy OR intervention* OR meditation OR 'memory specificity training' OR mindfulness OR 'minimal therapist support' OR 'motivational interviewing' OR program* OR psychoanaly* OR 'psycho analy*' OR psychodrama OR 'psycho drama*' OR psychodynamic OR psychoeducat* OR 'psycho educat*' OR psychotherap* OR relaxation OR 'self care' OR selfcare OR 'self help' OR selfhelp OR 'self administered' OR selfadministered OR 'self management' OR support OR therap* OR treatment*)):ti,kw |
| 35 | #24 OR #25 OR #26 OR #27 OR #28 OR #29 OR #30 OR #31 OR #32 OR #33 OR #34 |
| 36 | 'mobile phone'/exp |
| 37 | 'personal digital assistant'/de |
| 38 | 'e-mail'/de |
| 39 | 'internet'/de |
| 40 | 'mobile application'/exp |
| 41 | android:ti OR app:ti OR apps:ti OR blackberry:ti OR cellphone:ti OR (((cell* OR mobile OR smart) NEAR/3 (phone* OR telephone*)):ti) OR chat*:ti OR computer*:ti OR cyber*:ti OR digital:ti OR 'electronic mail*':ti OR 'e mail':ti OR email:ti OR 'handheld device*':ti OR 'instant messag*':ti OR internet*:ti OR ipad*:ti OR 'i pad*':ti OR iphone*:ti OR 'i phone*':ti OR messaging:ti OR 'mobile application*':ti OR online*:ti OR 'on line*':ti OR 'personal digital assistant*':ti OR 'short messag*':ti OR smartphone*:ti OR sms:ti OR tablet*:ti OR 'text messag*':ti OR texting:ti OR webbased:ti OR webdeliver*:ti OR 'web based':ti OR 'web deliver*':ti OR website*:ti |
| 42 | #36 OR #37 OR #38 OR #39 OR #40 OR #41 |
| 43 | 'counseling'/de |
| 44 | 'directive counseling'/de |
| 45 | 'motivational interviewing'/de |
| 46 | 'patient counseling'/de |
| 47 | 'patient guidance'/de |
| 48 | 'psychotherapy'/exp |
| 49 | 'self care'/de |
| 50 | 'self help'/de |
| 51 | therapy:lnk |
| 52 | behavio*:ti OR cognitive:ti |
| 53 | act:ti,ab,kw OR ((attention NEAR/2 modification):ti,ab,kw) OR 'attention training':ti,ab,kw OR (((behavio* OR cognitive) NEAR/2 (activat* OR component$ OR contracting OR defusion OR guidance OR management OR modif* OR rehab* OR restructur* OR technique* OR train*)):ti,ab,kw) OR cbt:ti,ab,kw OR 'cognitive behavio*':ti,ab,kw OR consultation*:ti,ab,kw OR counceling:ti,ab,kw OR councelling:ti,ab,kw OR dbt:ti,ab,kw OR 'dialectical behavio*':ti,ab,kw OR hypnosis:ti,ab,kw OR hypnotherapy:ti,ab,kw OR intervention*:ti,ab,kw OR meditation:ti,ab,kw OR 'memory specificity training':ti,ab,kw OR mindfulness:ti,ab,kw OR 'minimal therapist support':ti,ab,kw OR 'motivational interviewing':ti,ab,kw OR program*:ti,ab,kw OR psychoanaly*:ti,ab,kw OR 'psycho analy*':ti,ab,kw OR psychodrama:ti,ab,kw OR 'psycho drama*':ti,ab,kw OR psychodynamic:ti,ab,kw OR psychoeducat*:ti,ab,kw OR 'psycho educat*':ti,ab,kw OR psychotherap*:ti,ab,kw OR relaxation:ti,ab,kw OR ((('self care' OR selfcare OR 'self help' OR selfhelp) NEAR/3 (administered OR guided OR instruct* OR strateg* OR supervised OR tool*)):ti,ab,kw) OR ((('self administered' OR selfadministered) NEAR/3 (guided OR instruct* OR strateg* OR supervised)):ti,ab,kw) OR 'self management':ti,ab,kw OR support:ti,ab,kw OR therap*:ti,ab,kw OR treatment*:ti,ab,kw |
| 54 | #43 OR #44 OR #45 OR #46 OR #47 OR #48 OR #49 OR #50 OR #51 OR #52 OR #53 |
| 55 | #23 AND #35 |
| 56 | #23 AND #42 AND #54 |
| 57 | #55 OR #56 |
| 58 | (#55 OR #56) AND [2013-2021]/py |
| 59 | (#55 OR #56) AND [2013-2021]/py AND ([danish]/lim OR [english]/lim OR [norwegian]/lim OR [swedish]/lim) |
| 60 | ('randomized controlled trial'/exp OR 'controlled clinical trial'/de OR random*:ti,ab,tt OR 'randomization'/de OR 'intermethod comparison'/de OR placebo:ti,ab,tt OR compare:ti,tt OR compared:ti,tt OR comparison:ti,tt OR ((evaluated:ab OR evaluate:ab OR evaluating:ab OR assessed:ab OR assess:ab) AND (compare:ab OR compared:ab OR comparing:ab OR comparison:ab)) OR ((open NEXT/1 label):ti,ab,tt) OR (((double OR single OR doubly OR singly) NEXT/1 (blind OR blinded OR blindly)):ti,ab,tt) OR 'double blind procedure'/de OR ((parallel NEXT/1 group*):ti,ab,tt) OR crossover:ti,ab,tt OR 'cross over':ti,ab,tt OR (((assign* OR match OR matched OR allocation) NEAR/6 (alternate OR group OR groups OR intervention OR interventions OR patient OR patients OR subject OR subjects OR participant OR participants)):ti,ab,tt) OR assigned:ti,ab,tt OR allocated:ti,ab,tt OR ((controlled NEAR/8 (study OR design OR trial)):ti,ab,tt) OR volunteer:ti,ab,tt OR volunteers:ti,ab,tt OR 'human experiment'/de OR trial:ti,tt) NOT (((random* NEXT/1 sampl* NEAR/8 ('cross section*' OR questionnaire* OR survey OR surveys OR database OR databases)):ti,ab,tt) NOT ('comparative study'/de OR 'controlled study'/de OR 'randomised controlled':ti,ab,tt OR 'randomized controlled':ti,ab,tt OR 'randomly assigned':ti,ab,tt) OR ('cross‐sectional study' NOT ('randomized controlled trial'/exp OR 'controlled clinical trial'/de OR 'controlled study'/de OR 'randomised controlled':ti,ab,tt OR 'randomized controlled':ti,ab,tt OR 'control group':ti,ab,tt OR 'control groups':ti,ab,tt)) OR ('case control*':ti,ab,tt AND random*:ti,ab,tt NOT ('randomised controlled':ti,ab,tt OR 'randomized controlled':ti,ab,tt)) OR ('systematic review':ti,tt NOT (trial:ti,tt OR study:ti,tt)) OR (nonrandom*:ti,ab,tt NOT random*:ti,ab,tt) OR 'random field*':ti,ab,tt OR (('random cluster' NEAR/4 sampl*):ti,ab,tt) OR (review:ab AND review:it NOT trial:ti,tt) OR ('we searched':ab AND (review:ti,tt OR review:it)) OR 'update review':ab OR ((databases NEAR/5 searched):ab) OR ((rat:ti,tt OR rats:ti,tt OR mouse:ti,tt OR mice:ti,tt OR swine:ti,tt OR porcine:ti,tt OR murine:ti,tt OR sheep:ti,tt OR lambs:ti,tt OR pigs:ti,tt OR piglets:ti,tt OR rabbit:ti,tt OR rabbits:ti,tt OR cat:ti,tt OR cats:ti,tt OR dog:ti,tt OR dogs:ti,tt OR cattle:ti,tt OR bovine:ti,tt OR monkey:ti,tt OR monkeys:ti,tt OR trout:ti,tt OR marmoset*:ti,tt) AND 'animal experiment'/de) OR ('animal experiment'/de NOT ('human experiment'/de OR 'human'/de))) |
| 61 | #59 AND #60 |
| 62 | 'premenstrual dysphoric disorder'/de |
| 63 | 'mutism'/exp |
| 64 | 'hypochondriasis'/de |
| 65 | 'health anxiety'/de |
| 66 | 'body dysmorphic disorder'/exp |
| 67 | 'olfactory reference syndrome'/de |
| 68 | 'hoarding disorder'/exp |
| 69 | 'trichotillomania'/de |
| 70 | 'excoriation disorder'/de |
| 71 | 'excoriation' |
| 72 | 'skin picking disorder'/de |
| 73 | 'skin picking'/de |
| 74 | 'psychosocial disorder'/de |
| 75 | 'disinhibited social engagement disorder'/de |
| 76 | 'posttraumatic stress disorder'/de |
| 77 | 'acute stress disorder'/de |
| 78 | 'acute stress'/de |
| 79 | 'adjustment disorder'/de |
| 80 | 'prolonged grief'/exp |
| 81 | 'acute stress':ti,kw OR 'adjustment disorder*':ti,kw OR 'adjustment react*':ti,kw OR 'body dysmorphic disorder*':ti,kw OR 'body image disfunction*':ti,kw OR 'body image disorder*':ti,kw OR 'dermatillomani*':ti,kw OR 'disinhibited social engagement disorder*':ti,kw OR 'disruptive mood dysregulation disorder*':ti,kw OR dysmorphophobi*:ti,kw OR 'excoriation disorder*':ti,kw OR 'hair pulling':ti,kw OR 'health anxiety':ti,kw OR hoarding:ti,kw OR hypochondri*:ti,kw OR 'illness anxiety':ti,kw OR 'mixed anxi*':ti,kw OR 'mixed depressive':ti,kw OR mutism:ti,kw OR 'olfactory reference disorder*':ti,kw OR 'olfactory reference syndrom*':ti,kw OR (((posttrauma* OR 'post trauma*') NEAR/3 (disorder* OR neuros* OR psych* OR stress* OR symptom* OR syndrom*)):ti,kw) OR 'premenstrual dysphori*':ti,kw OR 'premenstrual syndrom*':ti,kw OR 'prolonged grief disorder*':ti,kw OR ptsd:ti,kw OR 'reactive attachment disorder*':ti,kw OR 'skin picking':ti,kw OR trichotillomani*:ti,kw |
| 82 | #62 OR #63 OR #64 OR #65 OR #66 OR #67 OR #68 OR #69 OR #70 OR #71 OR #72 OR #73 OR #74 OR #75 OR #76 OR #77 OR #78 OR #79 OR #80 OR #81 |
| 83 | #35 AND #82 |
| 84 | #42 AND #54 AND #82 |
| 85 | #83 OR #84 |
| 86 | #60 AND #85 |
| 87 | #86 AND ([danish]/lim OR [english]/lim OR [norwegian]/lim OR [swedish]/lim) |
| 88 | #61 OR #87 |
| 89 | (#59 OR #85) AND [01-01-1000]/sd NOT [27-08-2021]/sd |
| 90 | (#61 OR #87) AND [01-01-1000]/sd NOT [27-08-2021]/sd |
| 91 | l1370322048 or l2000857383 or l2006122165 or l2007406786 or l2011399271 or l2011536606 or l36221454 or l373330714 or l52772658 or l604025173 or l605976055 or l609227575 or l613367050 or l614638587 or l618360856 or l619354556 or l620211073 or l631847368 or l633475008 or l635026182 |
| 92 | #91 NOT #89 |
| 93 | #91 NOT #90 |

*SBU Assessment 372: Treatment and social support for adults with co-occurring addictive and psychiatric disorders*

| **#** | **Searches** |
| --- | --- |
| 1 | 'drug dependence'/de OR 'alcoholism'/exp OR 'amphetamine dependence'/de OR 'benzodiazepine dependence'/de OR 'cannabis addiction'/de OR 'cocaine dependence'/de OR 'congenital drug dependence'/exp OR 'drug abuse pattern'/de OR 'drug craving'/de OR 'drug misuse'/exp OR 'drug seeking behavior'/exp OR 'glue sniffing'/exp OR 'methamphetamine dependence'/exp OR 'multiple drug abuse'/exp OR 'narcotic dependence'/exp OR 'phencyclidine dependence'/exp OR 'substance abuse'/de |
| 2 | ((alcohol* OR amphetamine* OR cannabis OR cocaine OR crack OR drug OR hallucinogen* OR hashish OR heroin OR inhalant* OR marihuana OR marijuana OR narcotic* OR opiate* OR opioid* OR phencyclidine OR psychedelic* OR substance*) NEAR/3 (abuse OR addict* OR dependen* OR disorder* OR misuse)):ti,ab,kw |
| 3 | alcoholism:ti,ab,kw OR alcoholic*:ti,ab,kw OR 'chronic alcohol misuse':ti,ab,kw OR 'chronic excessive alcohol':ti,ab,kw |
| 4 | ((harmful OR hazardous OR heavy) NEXT/1 (drink* OR alcohol*)):ti,ab,kw |
| 5 | #1 OR #2 OR #3 OR #4 |
| 6 | 'mental disease'/de OR 'mental patient'/de OR 'adjustment disorder'/exp OR 'alexithymia'/de OR 'anxiety disorder'/exp OR 'autism'/exp OR 'behavior disorder'/exp OR 'dissociative disorder'/exp OR 'emotional disorder'/exp OR 'hikikomori'/exp OR 'mental deficiency'/exp OR 'mental infantilism'/exp OR 'mental instability'/exp OR 'mental overstimulation'/exp OR 'mood disorder'/exp OR 'neurosis'/exp OR 'organic brain syndrome'/exp OR 'organic psychosyndrome'/exp OR 'pathological lying'/exp OR 'personality disorder'/exp OR 'psychiatric complication'/exp OR 'psychosexual disorder'/exp OR 'psychosis'/exp OR 'psychosomatic disorder'/exp OR 'psychotrauma'/de OR 'schizophrenia spectrum disorder'/exp OR 'stupor'/exp OR 'thought disorder'/exp |
| 7 | (((persistent OR serious OR severe*) NEAR/3 (psychiatric OR mental)):ti,ab,kw) OR 'psychiatric inpatient*':ti,ab,kw OR 'psychiatric in-patients':ti,ab,kw OR 'psychiatric disorder*':ti,ab,kw OR 'psychiatric condition*':ti,ab,kw OR 'mental healthcare':ti,ab,kw OR 'mental disorders*':ti,ab,kw |
| 8 | ((borderline OR 'anti social' OR antisocial OR paranoid) NEAR/3 ('personality disorder*' OR behaviour OR behavior)):ti,kw |
| 9 | adhd:ti,kw OR agoraphobia:ti,kw OR anorexi*:ti,kw OR 'anxiety disorder*':ti,kw OR arson*:ti,kw OR 'attentiondeficit/hyperactivity-disorder':ti,kw OR 'attention deficit disorder*':ti,kw OR 'attention deficit hyperactivity':ti,kw OR autism:ti,kw OR autistic:ti,kw OR 'binge-eating disorder':ti,kw OR bipol*:ti,kw OR 'body dysmorphic disorder*':ti,kw OR 'body integrity identity disorder*':ti,kw OR bulimi*:ti,kw OR 'chronic stress':ti,kw OR 'conversion disorder*':ti,kw OR 'depressive disorder*':ti,kw OR depression:ti,kw OR 'dissociative disorder*':ti,kw OR 'dissociative identity disorder*':ti,kw OR 'disruptive behavior':ti,kw OR 'disruptive behaviour':ti,kw OR 'dual personality':ti,kw OR dyspareunia:ti,kw OR 'eating disorder*':ti,kw OR 'elimination disorder*':ti,kw OR 'erectile dysfunction':ti,kw OR exhibitionism:ti,kw OR 'factitious disorder*':ti,kw OR 'gender dysphoria':ti,kw OR 'globus sensation':ti,kw OR 'hyperactivity syndrome*':ti,kw OR 'hyperkinetic disorder*':ti,kw OR 'hyperkinetic conduct disorder':ti,kw OR 'hyperkinetic syndrome*':ti,kw OR hypochondrias*:ti,kw OR 'impulse control disorder*':ti,kw OR 'inattentive hyperactive':ti,kw OR 'intellectual disability':ti,kw OR kleptomania:ti,kw OR 'major depressive':ti,kw OR masochism:ti,kw OR 'medically unexplained syndrome*':ti,kw OR 'mental healthcare':ti,kw OR 'mental disorders*':ti,kw OR 'mental illness':ti,kw OR 'mentally ill':ti,kw OR 'minimal brain dysfunction*':ti,kw OR 'mood disorder*':ti,kw OR 'motor disorder*':ti,kw OR 'motor skills disorder*':ti,kw OR 'motor skill disorder*':ti,kw OR 'multiple identity disorder':ti,kw OR 'multiple personalit*':ti,kw OR 'munchausen syndrome':ti,kw OR neurasthenia:ti,kw OR 'neurocognitive disorder*':ti,kw OR neurodevelop*:ti,kw OR 'neurotic disorder*':ti,kw OR neuroticism:ti,kw OR neuroses:ti,kw OR 'obsessivecompulsive disorder':ti,kw OR 'panic disorder':ti,kw OR paraphilia*:ti,kw OR 'paraphilic disorder*':ti,kw OR pedophilia:ti,kw OR phobia:ti,kw OR 'phobic disorder*':ti,kw OR 'posttraumatic stress':ti,kw OR 'post-traumatic stress':ti,kw OR 'psychiatric condition*':ti,kw OR 'psychiatric disorder*':ti,kw OR 'psychiatric fetishism':ti,kw OR 'psychiatric inpatient*':ti,kw OR 'psychiatric in-patients':ti,kw OR 'psychological sexual dysfunction*':ti,kw OR 'psychosexual disorder*':ti,kw OR psychosis:ti,kw OR 'psychotic disorder*':ti,kw OR ptsd:ti,kw OR pyromania:ti,kw OR sadism:ti,kw OR schizoaffective:ti,kw OR schizoid:ti,kw OR schizophren*:ti,kw OR schizotypal:ti,kw OR 'sex deviation*':ti,kw OR 'sexual aversion disorder*':ti,kw OR 'sexual and gender disorders':ti,kw OR 'sleep disorder*':ti,kw OR 'somatization disorder*':ti,kw OR 'somatoform disorder*':ti,kw OR transvestism:ti,kw OR 'trauma-related mental health problem*':ti,kw OR trichotillomania:ti,kw OR vaginismus:ti,kw OR voyeurism:ti,kw |
| 10 | #6 OR #7 OR #8 OR #9 |
| 11 | ('randomized controlled trial'/exp OR 'controlled clinical trial'/de OR random*:ti,ab,tt OR 'randomization'/de OR 'intermethod comparison'/de OR placebo:ti,ab,tt OR compare:ti,tt OR compared:ti,tt OR comparison:ti,tt OR ((evaluated:ab OR evaluate:ab OR evaluating:ab OR assessed:ab OR assess:ab) AND (compare:ab OR compared:ab OR comparing:ab OR comparison:ab)) OR ((open NEXT/1 label):ti,ab,tt) OR (((double OR single OR doubly OR singly) NEXT/1 (blind OR blinded OR blindly)):ti,ab,tt) OR 'double blind procedure'/de OR ((parallel NEXT/1 group*):ti,ab,tt) OR crossover:ti,ab,tt OR 'cross over':ti,ab,tt OR (((assign* OR match OR matched OR allocation) NEAR/6 (alternate OR group OR groups OR intervention OR interventions OR patient OR patients OR subject OR subjects OR participant OR participants)):ti,ab,tt) OR assigned:ti,ab,tt OR allocated:ti,ab,tt OR ((controlled NEAR/8 (study OR design OR trial)):ti,ab,tt) OR volunteer:ti,ab,tt OR volunteers:ti,ab,tt OR 'human experiment'/de OR trial:ti,tt) NOT (((random* NEXT/1 sampl* NEAR/8 ('cross section*' OR questionnaire* OR survey OR surveys OR database OR databases)):ti,ab,tt) NOT ('comparative study'/de OR 'controlled study'/de OR 'randomised controlled':ti,ab,tt OR 'randomized controlled':ti,ab,tt OR 'randomly assigned':ti,ab,tt) OR ('cross‐sectional study' NOT ('randomized controlled trial'/exp OR 'controlled clinical trial'/de OR 'controlled study'/de OR 'randomised controlled':ti,ab,tt OR 'randomized controlled':ti,ab,tt OR 'control group':ti,ab,tt OR 'control groups':ti,ab,tt)) OR ('case control*':ti,ab,tt AND random*:ti,ab,tt NOT ('randomised controlled':ti,ab,tt OR 'randomized controlled':ti,ab,tt)) OR ('systematic review':ti,tt NOT (trial:ti,tt OR study:ti,tt)) OR (nonrandom*:ti,ab,tt NOT random*:ti,ab,tt) OR 'random field*':ti,ab,tt OR (('random cluster' NEAR/4 sampl*):ti,ab,tt) OR (review:ab AND review:it NOT trial:ti,tt) OR ('we searched':ab AND (review:ti,tt OR review:it)) OR 'update review':ab OR ((databases NEAR/5 searched):ab) OR ((rat:ti,tt OR rats:ti,tt OR mouse:ti,tt OR mice:ti,tt OR swine:ti,tt OR porcine:ti,tt OR murine:ti,tt OR sheep:ti,tt OR lambs:ti,tt OR pigs:ti,tt OR piglets:ti,tt OR rabbit:ti,tt OR rabbits:ti,tt OR cat:ti,tt OR cats:ti,tt OR dog:ti,tt OR dogs:ti,tt OR cattle:ti,tt OR bovine:ti,tt OR monkey:ti,tt OR monkeys:ti,tt OR trout:ti,tt OR marmoset*:ti,tt) AND 'animal experiment'/de) OR ('animal experiment'/de NOT ('human experiment'/de OR 'human'/de))) |
| 12 | 'psychiatric dual diagnosis'/exp OR (('co occurr*':ti OR comorbid:ti OR concurrent:ti) AND (mental:ti OR psychiatric*:ti OR substance*:ti)) |
| 13 | #5 AND #10 |
| 14 | #12 OR #13 |
| 15 | #14 NOT ('juvenile'/exp NOT 'adult'/exp) |
| 16 | #11 AND #15 |
| 17 | #16 AND ([danish]/lim OR [english]/lim OR [norwegian]/lim OR [swedish]/lim) AND [embase]/lim |
| 18 | #16 AND ([danish]/lim OR [english]/lim OR [norwegian]/lim OR [swedish]/lim) AND [embase]/lim NOT ([conference abstract]/lim OR [conference paper]/lim OR [conference review]/lim OR [editorial]/lim OR [letter]/lim OR [note]/lim OR [short survey]/lim) |
| 19 | #16 AND ([danish]/lim OR [english]/lim OR [norwegian]/lim OR [swedish]/lim) AND [embase]/lim NOT ([conference abstract]/lim OR [conference paper]/lim OR [conference review]/lim OR [editorial]/lim OR [letter]/lim OR [note]/lim OR [short survey]/lim) AND [01-01-1000]/sd NOT [12-04-2023]/sd |
| 20 | #15 AND ([danish]/lim OR [english]/lim OR [norwegian]/lim OR [swedish]/lim) AND [embase]/lim |
| 21 | #20 AND ([danish]/lim OR [english]/lim OR [norwegian]/lim OR [swedish]/lim) AND [embase]/lim NOT ([conference abstract]/lim OR [conference paper]/lim OR [conference review]/lim OR [editorial]/lim OR [letter]/lim OR [note]/lim OR [short survey]/lim) |
| 22 | #21 AND ([danish]/lim OR [english]/lim OR [norwegian]/lim OR [swedish]/lim) AND [embase]/lim NOT ([conference abstract]/lim OR [conference paper]/lim OR [conference review]/lim OR [editorial]/lim OR [letter]/lim OR [note]/lim OR [short survey]/lim) AND [01-01-1000]/sd NOT [12-04-2023]/sd |
| 23 | l13094932 OR l13198437 OR l18243265 OR l2005728200 OR l2010544285 OR l2021698033 OR l21026750 OR l22056919 OR l23005139 OR l23156782 OR l25128475 OR l25309389 OR l26085205 OR l27344096 OR l28087601 OR l28271639 OR l28496692 OR l30202353 OR l32545407 OR l34919936 OR l34919937 OR l350293892 OR l351106760 OR l351549391 OR l351838678 OR l351982668 OR l352177034 OR l354185195 OR l358927108 OR l359694426 OR l359967414 OR l361076772 OR l361497287 OR l361804902 OR l364262705 OR l368931521 OR l369509978 OR l369836856 OR l37443186 OR l37455862 OR l38146549 OR l38294864 OR l38380746 OR l38392322 OR l38501686 OR l40095683 OR l40096069 OR l40363422 OR l40446132 OR l40615774 OR l41318273 OR l41427635 OR l41821797 OR l43167083 OR l43167084 OR l43530392 OR l44166341 OR l44427772 OR l46074379 OR l46434899 OR l46594932 OR l47415050 OR l50115213 OR l51719069 OR l52047388 OR l52656357 OR l53057911 OR l53217758 OR l53283965 OR l550070272 OR l601711026 OR l602904009 OR l603057332 OR l6030635 OR l603512595 OR l604878179 OR l606251480 OR l606662894 OR l607404060 OR l609182158 OR l613281539 OR l613493949 OR l615003333 OR l616352099 OR l622939978 OR l624688493 OR l636961368 OR l90485853 |
| 24 | #23 NOT #22 |
| 25 | #23 NOT #19 |

*SBU Policy support 379: Treatment and rehabilitation of post-covid and other post-infectious conditions*

| **#** | **Searches** |
| --- | --- |
| 1 | 'coronavirus disease 2019'/exp/dm_rh |
| 2 | 'coronavirus infection'/dm_rh |
| 3 | ((covid* OR coronavirus* OR 'corona virus*' OR 'sars cov 2' OR '2019 ncov') NEAR/3 ('after care' OR aftercare OR chronic* OR continuing OR convalesc* OR enduring OR late OR lingering OR long* OR 'non specific' OR nonspecific OR permanent OR persist* OR post* OR prolong* OR rehab* OR recover* OR recurr* OR relaps* OR remaining OR residing OR residual OR sequela* OR sequelae* OR telerehab*)):ti,kw |
| 4 | ((covid* OR coronavirus* OR 'corona virus*' OR 'sars cov 2' OR '2019 ncov') NEAR/5 ('after care' OR aftercare OR chronic* OR continuing OR convalesc* OR enduring OR late OR lingering OR long* OR longterm* OR 'non specific' OR nonspecific OR permanent OR persist* OR post* OR prolong* OR rehab* OR recover* OR recurr* OR relaps* OR remaining OR residing OR residual OR sequela* OR sequelae* OR telerehab*) NEAR/5 (anxiet* OR arrhythmia* OR arrythmia* OR asthma* OR brain OR breath* OR cardiac* OR cognit* OR depress* OR dizz* OR dyspnea* OR dysrhythmia* OR fatigue OR fever* OR gustat* OR heart* OR insomnia OR kidney* OR lung* OR nausea OR neuro* OR olfact* OR pain OR palpitation* OR 'postural orthostatic tachycardia syndrome' OR pots OR pneumoni* OR pulmonary OR respirat* OR sleep* OR smell* OR spinning OR symptom* OR tast* OR vertigo*)):ab |
| 5 | 'long covid'/de |
| 6 | postcovid*:ti,ab,kw |
| 7 | #1 OR #2 OR #3 OR #4 OR #5 OR #6 |
| 8 | 'coronavirus disease 2019'/exp |
| 9 | 'coronavirus infection'/de |
| 10 | covid*:ti,ab,kw OR coronavirus*:ti,ab,kw OR 'corona virus*':ti,ab,kw OR 'sars cov 2':ti,ab,kw OR '2019 ncov':ti,ab,kw |
| 11 | #8 OR #9 OR #10 |
| 12 | (persist* NEAR/5 symptom*):ti,ab,kw |
| 13 | 'long haul*':ti,ab,kw OR 'post acute':ti,ab,kw OR 'post viral':ti,ab,kw OR 'post virus':ti,ab,kw OR sequela*:ti,ab,kw OR sequelae*:ti,ab,kw |
| 14 | #12 OR #13 |
| 15 | #11 AND #14 |
| 16 | 'long covid*':ab OR 'post covid*':ab |
| 17 | #7 OR #15 OR #16 |
| 18 | #17 NOT 'conference abstract'/it |
| 19 | #18 AND [01-01-2022]/sd NOT [04-03-2024]/sd |
| 20 | ('randomized controlled trial'/exp OR 'controlled clinical trial'/de OR random*:ti,ab,tt OR 'randomization'/de OR 'intermethod comparison'/de OR placebo:ti,ab,tt OR compare:ti,tt OR compared:ti,tt OR comparison:ti,tt OR ((evaluated:ab OR evaluate:ab OR evaluating:ab OR assessed:ab OR assess:ab) AND (compare:ab OR compared:ab OR comparing:ab OR comparison:ab)) OR ((open NEXT/1 label):ti,ab,tt) OR (((double OR single OR doubly OR singly) NEXT/1 (blind OR blinded OR blindly)):ti,ab,tt) OR 'double blind procedure'/de OR ((parallel NEXT/1 group*):ti,ab,tt) OR crossover:ti,ab,tt OR 'cross over':ti,ab,tt OR (((assign* OR match OR matched OR allocation) NEAR/6 (alternate OR group OR groups OR intervention OR interventions OR patient OR patients OR subject OR subjects OR participant OR participants)):ti,ab,tt) OR assigned:ti,ab,tt OR allocated:ti,ab,tt OR ((controlled NEAR/8 (study OR design OR trial)):ti,ab,tt) OR volunteer:ti,ab,tt OR volunteers:ti,ab,tt OR 'human experiment'/de OR trial:ti,tt) NOT (((random* NEXT/1 sampl* NEAR/8 ('cross section*' OR questionnaire* OR survey OR surveys OR database OR databases)):ti,ab,tt) NOT ('comparative study'/de OR 'controlled study'/de OR 'randomised controlled':ti,ab,tt OR 'randomized controlled':ti,ab,tt OR 'randomly assigned':ti,ab,tt) OR ('cross‐sectional study' NOT ('randomized controlled trial'/exp OR 'controlled clinical trial'/de OR 'controlled study'/de OR 'randomised controlled':ti,ab,tt OR 'randomized controlled':ti,ab,tt OR 'control group':ti,ab,tt OR 'control groups':ti,ab,tt)) OR ('case control*':ti,ab,tt AND random*:ti,ab,tt NOT ('randomised controlled':ti,ab,tt OR 'randomized controlled':ti,ab,tt)) OR ('systematic review':ti,tt NOT (trial:ti,tt OR study:ti,tt)) OR (nonrandom*:ti,ab,tt NOT random*:ti,ab,tt) OR 'random field*':ti,ab,tt OR (('random cluster' NEAR/4 sampl*):ti,ab,tt) OR (review:ab AND review:it NOT trial:ti,tt) OR ('we searched':ab AND (review:ti,tt OR review:it)) OR 'update review':ab OR ((databases NEAR/5 searched):ab) OR ((rat:ti,tt OR rats:ti,tt OR mouse:ti,tt OR mice:ti,tt OR swine:ti,tt OR porcine:ti,tt OR murine:ti,tt OR sheep:ti,tt OR lambs:ti,tt OR pigs:ti,tt OR piglets:ti,tt OR rabbit:ti,tt OR rabbits:ti,tt OR cat:ti,tt OR cats:ti,tt OR dog:ti,tt OR dogs:ti,tt OR cattle:ti,tt OR bovine:ti,tt OR monkey:ti,tt OR monkeys:ti,tt OR trout:ti,tt OR marmoset*:ti,tt) AND 'animal experiment'/de) OR ('animal experiment'/de NOT ('human experiment'/de OR 'human'/de))) |
| 21 | #18 AND #20 |
| 22 | #21 AND [01-01-2022]/sd NOT [04-03-2024]/sd |
| 23 | l2018768964 OR l2019542328 OR l2020130695 OR l2020484054 OR l2020725287 OR l2021403559 OR l2021960885 OR l2022035420 OR l2022285605 OR l2022447916 OR l2022534632 OR l2023606418 OR l2023872090 OR l2024043044 OR l2024200331 OR l2024754656 OR l2024880874 OR l2024999502 OR l2025140548 OR l2025404064 OR l2026037552 OR l2026735540 OR l2027344672 OR l2028217553 OR l2028224621 OR l2028952890 OR l2029636832 OR l2030299306 OR l2030329973 OR l2030773261 OR l638467304 OR l639039935 OR l639912378 OR l641294999 OR l641350881 OR l641724928 OR l642181720 OR l642864325 OR l643344788 OR l643441893 |
| 24 | #23 NOT #19 |
| 25 | #23 NOT #22 |
