# Supplementary material for: Combining search filters for randomized controlled trials with the Cochrane RCT Classifier in Covidence: a methodological validation study
Source: Res Synth Methods. 2025 Aug 28;16(6):953–60. doi: 10.1017/rsm.2025.10023 (PMC12657657; doi:10.1017/rsm.2025.10023)
Supplement: Moberg and Gornitzki supplementary material [file S1759287925100239sup001.zip › Appendix S1.docx]

**Appendix S1**

**SBU reports used to generate the gold standard set**

1. *[Apnea test in the diagnosis of brain death]*. Stockholm: Swedish Agency for Health Technology Assessment and Assessment of Social Services (SBU); 2020. SBU Policy support 310. https://www.sbu.se/310. Published May 3, 2020. Accessed Feb 14 2025.

2. *[Associations between the use of snus (moist tobacco) or electronic cigarettes and tobacco smoking]*. Stockholm: Swedish Agency for Health Technology Assessment and Assessment of Social Services (SBU); 2020. SBU Policy support 312. https://www.sbu.se/312e. Published Jun 15, 2020. Accessed Feb 14 2025.

3. *[Care for parents and siblings of stillborn children]*. Stockholm: Swedish Agency for Health Technology Assessment and Assessment of Social Services (SBU); 2022. SBU Assessment 354. https://www.sbu.se/354. Published Nov 28, 2022. Accessed Feb 14 2025.

4. *[Care for persons exposed to sexual violence: psychological treatment of complex PTSD]*. Stockholm: Swedish Agency for Health Technology Assessment and Assessment of Social Services (SBU); 2024. SBU Policy support 381. https://www.sbu.se/381e. Published Aug 26, 2024. Accessed Feb 14 2025.

5. *[Cervical cancer screening with HPV self-sampling]*. Stockholm: Swedish Agency for Health Technology Assessment and Assessment of Social Services (SBU); 2021. SBU Policy support 338. https://www.sbu.se/338. Published Nov 14, 2021. Accessed Feb 14 2025.

6. *[Continuity of care]*. Stockholm: Swedish Agency for Health Technology Assessment and Assessment of Social Services (SBU); 2021. SBU Assessment 329. https://www.sbu.se/329e. Published Aug 18, 2021. Accessed Feb 14 2025.

7. *[Development of a Core Outcome Set (COS) for treatment of depression during or after pregnancy (antenatal and postpartum depression)]*. Stockholm: Swedish Agency for Health Technology Assessment and Assessment of Social Services (SBU); 2020. SBU Policy support 314. https://www.sbu.se/314e. Published Jun 7, 2020. Accessed Feb 14 2025.

8. *[Diagnostics and treatment of provoked vestibulodynia]*. Stockholm: Swedish Agency for Health Technology Assessment and Assessment of Social Services (SBU); 2021. SBU Assessment 326. https://www.sbu.se/326e. Published Jun 29, 2021. Accessed Feb 14 2025.

9. *[Dialectical behavior therapy (DBT) and mentalization-based treatment (MBT)]*. Stockholm: Swedish Agency for Health Technology Assessment and Assessment of Social Services (SBU); 2024. SBU Policy support 385. https://www.sbu.se/385. Published Oct 23, 2024. Accessed Feb 14 2025.

10. *[Diets for diabetes]*. Stockholm: Swedish Agency for Health Technology Assessment and Assessment of Social Services (SBU); 2022. SBU Assessment 345. https://www.sbu.se/345e. Published Mar 16, 2022. Accessed Feb 14 2025.

11. *[The effect of vitamins, minerals and medical devices on the common cold]*. Stockholm: Swedish Agency for Health Technology Assessment and Assessment of Social Services (SBU); 2024. SBU Policy support 383. https://www.sbu.se/383. Published Aug 28, 2024. Accessed Feb 14 2025.

12. *[The effectiveness of policies, programs or interventions to improve health and psychosocial outcomes for young people leaving the out of home care system]*. Stockholm: Swedish Agency for Health Technology Assessment and Assessment of Social Services (SBU); 2020. SBU Policy support 316. https://www.sbu.se/316. Published Sep 16, 2020. Accessed Feb 14 2025.

13. *[Effects of active labour market programs for long-term social assistance recipients]*. Stockholm: Swedish Agency for Health Technology Assessment and Assessment of Social Services (SBU); 2022. SBU Assessment 351. https://www.sbu.se/351e. Published Oct 3, 2022. Accessed Feb 14 2025.

14. *[Effects of return-to-work interventions for persons on long-term sick-leave due to mood-, anxiety- or adjustment disorders]*. Stockholm: Swedish Agency for Health Technology Assessment and Assessment of Social Services (SBU); 2022. SBU Assessment 352. https://www.sbu.se/352e. Published Oct 3, 2022. Accessed Feb 14 2025.

15. *[Evaluation of taking mifepristone at home during a medical abortion]*. Stockholm: Swedish Agency for Health Technology Assessment and Assessment of Social Services (SBU); 2023. SBU Assessment 363. https://www.sbu.se/363e. Published May 8, 2023. Accessed Feb 14 2025.

16. *[Fear of childbirth, depression and anxiety during pregnancy]*. Stockholm: Swedish Agency for Health Technology Assessment and Assessment of Social Services (SBU); 2021. SBU Assessment 322. https://www.sbu.se/322e. Published Feb 24, 2021. Accessed Feb 14 2025.

17. *[Health care interventions in case of long-term sick leave]*. Stockholm: Swedish Agency for Health Technology Assessment and Assessment of Social Services (SBU); 2022. SBU Assessment 359. https://www.sbu.se/359e. Published Dec 21, 2022. Accessed Feb 14 2025.

18. *[Impact on coagulation due to coronavirus]*. Stockholm: Swedish Agency for Health Technology Assessment and Assessment of Social Services (SBU); 2020. SBU Enquiry Service ut202026. https://www.sbu.se/ut202026. Published Jun 23, 2022. Accessed Feb 14 2025.

19. *[Internet-delivered psychological treatment versus other available treatment options for common mental disorders]*. Stockholm: Swedish Agency for Health Technology Assessment and Assessment of Social Services (SBU); 2021. SBU Assessment 337. https://www.sbu.se/337. Published Nov 7, 2021. Accessed Feb 14 2025.

20. *[Lipoedema – diagnosis, treatment, and experiences]*. Stockholm: Swedish Agency for Health Technology Assessment and Assessment of Social Services (SBU); 2021. SBU Assessment 327. https://www.sbu.se/327e. Published Jul 1, 2021. Accessed Feb 14 2025.

21. *[Multimodal and interdisciplinary interventions for long term pain]*. Stockholm: Swedish Agency for Health Technology Assessment and Assessment of Social Services (SBU); 2021. SBU Assessment 341. https://www.sbu.se/341e. Published Dec 15, 2021. Accessed Feb 14 2025.

22. *[Pharmacological treatment of common pain conditions in older persons]*. Stockholm: Swedish Agency for Health Technology Assessment and Assessment of Social Services (SBU); 2020. SBU Assessment 315. https://www.sbu.se/315e. Published Sep 2, 2020. Accessed Feb 14 2025.

23. *[Post COVID-19 – effective treatment and rehabilitation]*. Stockholm: Swedish Agency for Health Technology Assessment and Assessment of Social Services (SBU); 2021. SBU Policy support 328. https://www.sbu.se/328e. Published Aug 24, 2021. Accessed Feb 14 2025.

24. *[Practices to improve detection of perineal tears and women’s views and experiences of healthcare providers following sustained perineal tear]*. Stockholm: Swedish Agency for Health Technology Assessment and Assessment of Social Services (SBU); 2021. SBU Assessment 323. https://www.sbu.se/323e. Published Feb 28, 2021. Accessed Feb 14 2025.

25. *[Preventing problems with gambling for money]*. Stockholm: Swedish Agency for Health Technology Assessment and Assessment of Social Services (SBU); 2019. SBU Policy support 310. https://www.sbu.se/310. Published Mar 28, 2019. Accessed Feb 14 2025.

26. *[Prevention of self-harm and suicide attempts in children and adolescents at risk]*. Stockholm: Swedish Agency for Health Technology Assessment and Assessment of Social Services (SBU); 2024. SBU Assessment 378. https://www.sbu.se/378e. Published Jun 23, 2024. Accessed Feb 14 2025.

27. *[Programs for preventing suicide and suicide attempt in children]*. Stockholm: Swedish Agency for Health Technology Assessment and Assessment of Social Services (SBU); 2021. SBU Assessment 336. https://www.sbu.se/en/publications/sbu-assesses/programs-for-preventing-suicide-and-suicide-attempt-in-children/. Published Nov 18, 2021. Accessed Feb 14 2025.

28. *[Programs to prevent mental illness in children]*. Stockholm: Swedish Agency for Health Technology Assessment and Assessment of Social Services (SBU); 2021. SBU Assessment 339. https://www.sbu.se/339. Published Nov 18, 2021. Accessed Feb 14 2025.

29. *[Promoting mental well-being in children and adolescents - an in-depth analysis of the SEL programs]*. Stockholm: Swedish Agency for Health Technology Assessment and Assessment of Social Services (SBU); 2024. SBU Assessment 373. https://www.sbu.se/373. Published Feb 27, 2024. Accessed Feb 14 2025.

30. *[Promoting mental well-being in children and adolescents]*. Stockholm: Swedish Agency for Health Technology Assessment and Assessment of Social Services (SBU); 2022. SBU Assessment 350. https://www.sbu.se/350e. Published Oct 2, 2022. Accessed Feb 14 2025.

31. *[Psychological and psychosocial interventions for children aged 7 years or younger who have been exposed to sexual violence]*. Stockholm: Swedish Agency for Health Technology Assessment and Assessment of Social Services (SBU); 2024. SBU Policy support 380. https://www.sbu.se/380e. Published Aug 26, 2024. Accessed Feb 14 2025.

32. *[Psychological Treatment for Postpartum Depression]*. Stockholm: Swedish Agency for Health Technology Assessment and Assessment of Social Services (SBU); 2022. SBU Assessment 358. https://www.sbu.se/358e. Published Dec 18, 2022. Accessed Feb 14 2025.

33. *[Psychosocial interventions preventing gang-related crime among children and young adults under the age of 30]*. Stockholm: Swedish Agency for Health Technology Assessment and Assessment of Social Services (SBU); 2023. SBU Assessment 369. https://www.sbu.se/369e. Published Nov 30, 2023. Accessed Feb 14 2025.

34. *[Rehabilitation for adults with traumatic brain injury]*. Stockholm: Swedish Agency for Health Technology Assessment and Assessment of Social Services (SBU); 2019. SBU Assessment 304. https://www.sbu.se/304e. Published Dec 16, 2019. Accessed Feb 14 2025.

35. *[Relationship between S-CTX or PINP levels and decreased bone density]*. Stockholm: Swedish Agency for Health Technology Assessment and Assessment of Social Services (SBU); 2019. SBU Enquiry Service ut201921. https://www.sbu.se/ut201921. Published Sep 18, 2019. Accessed Feb 14 2025.

36. *[Scientific basis for national guidelines for the treatment of eating disorders]*. Stockholm: Swedish Agency for Health Technology Assessment and Assessment of Social Services (SBU); 2024. SBU Policy support 313. https://www.sbu.se/sbu2022_313. Published May 2, 2024. Accessed Feb 14 2025.

37. *[Scientific basis for the National Board of Health and Welfare's national guidelines for dental care]*. Stockholm: Swedish Agency for Health Technology Assessment and Assessment of Social Services (SBU); 2021. SBU Policy support 334. https://www.sbu.se/334. Published Sep 29, 2021. Accessed Feb 14 2025.

38. *[Scientific basis for the National Board of Health and Welfare's national guidelines for obstetric care]*. Stockholm: Swedish Agency for Health Technology Assessment and Assessment of Social Services (SBU); 2023. SBU Policy support 371. https://www.sbu.se/371. Published Dec 20, 2023. Accessed Feb 14 2025.

39. *[Treatment and rehabilitation of post-covid and other post-infectious conditions]*. Stockholm: Swedish Agency for Health Technology Assessment and Assessment of Social Services (SBU); 2024. SBU Policy support 379. https://www.sbu.se/379. Published Aug 13, 2024. Accessed Feb 14 2025.

40. *[Treatment and social support for adults with co-occurring addictive and psychiatric disorders - Part I: Pharmacological interventions, a preliminary report]*. Stockholm: Swedish Agency for Health Technology Assessment and Assessment of Social Services (SBU); 2024. SBU Assessment 372. https://www.sbu.se/372e. Published Feb 6, 2024. Accessed Feb 14 2025.

41. *[Treatment methods for rehabilitation of fibromyalgia]*. Stockholm: Swedish Agency for Health Technology Assessment and Assessment of Social Services (SBU); 2021. SBU Assessment 340. https://www.sbu.se/340e. Published Dec 15, 2021. Accessed Feb 14 2025.

42. *[Treatment of bile duct stones]*. Stockholm: Swedish Agency for Health Technology Assessment and Assessment of Social Services (SBU); 2019. SBU Assessment 297. https://www.sbu.se/297. Published Mar 19, 2019. Accessed Feb 14 2025.

43. *[Treatment of depression with transcranial magnetic stimulation using an H-coil (dTMS)]*. Stockholm: Swedish Agency for Health Technology Assessment and Assessment of Social Services (SBU); 2020. SBU Assessment 318. https://www.sbu.se/318e. Published Nov 26, 2020. Accessed Feb 14 2025.

44. *[Treatment of women with diastasis recti]*. Stockholm: Swedish Agency for Health Technology Assessment and Assessment of Social Services (SBU); 2022. SBU Assessment 346. https://www.sbu.se/346e. Published Mar 14, 2022. Accessed Feb 14 2025.

45. *[Treatments for extreme nausea and vomiting in pregnancy (hyperemesis gravidarum)]*. Stockholm: Swedish Agency for Health Technology Assessment and Assessment of Social Services (SBU); 2022. SBU Assessment 355. https://www.sbu.se/355e. Published Dec 13, 2022. Accessed Feb 14 2025.

46. *[Interventions to prevent juvenile offender recidivism]*. Stockholm: Swedish Agency for Health Technology Assessment and Assessment of Social Services (SBU); 2020. SBU Assessment 308. https://www.sbu.se/308. Published Mar 4, 2020. Accessed Feb 14 2025.

47. *[Wheelchairs and wheelchair accessories]*. Stockholm: Swedish Agency for Health Technology Assessment and Assessment of Social Services (SBU); 2022. SBU Assessment 347. https://www.sbu.se/347e. Published May 5, 2022. Accessed Feb 14 2025.
