# Supplementary material for: Combining search filters for randomized controlled trials with the Cochrane RCT Classifier in Covidence: a methodological validation study
Source: Res Synth Methods. 2025 Aug 28;16(6):953–60. doi: 10.1017/rsm.2025.10023 (PMC12657657; doi:10.1017/rsm.2025.10023)
Supplement: Moberg and Gornitzki supplementary material [file S1759287925100239sup001.zip › Appendix S2.docx]

**Appendix S2**

**References in the gold standard set**

1. Aasdahl L, Pape K, Vasseljen O, et al. Effect of Inpatient Multicomponent Occupational Rehabilitation Versus Less Comprehensive Outpatient Rehabilitation on Sickness Absence in Persons with Musculoskeletal- or Mental Health Disorders: A Randomized Clinical Trial. *J Occup Rehabil* 2018; **28**(1): 170-179. doi:10.1007/s10926-017-9708-z

2. Aasdahl L, Vasseljen O, Gismervik SØ, Johnsen R, Fimland MS. Two-Year Follow-Up of a Randomized Clinical Trial of Inpatient Multimodal Occupational Rehabilitation Vs Outpatient Acceptance and Commitment Therapy for Sick Listed Workers with Musculoskeletal or Common Mental Disorders. *J Occup Rehabil* 2021; **31**(4): 721-728. doi:10.1007/s10926-021-09969-4

3. Abas MN, Tan PC, Azmi N, Omar SZ. Ondansetron compared with metoclopramide for hyperemesis gravidarum: A randomized controlled trial. *Obstet Gynecol* 2014; **123**(6): 1272-1279. doi:10.1097/AOG.0000000000000242

4. Abásolo L, Blanco M, Bachiller J, et al. A health system program to reduce work disability related to musculoskeletal disorders. *Ann Intern Med* 2005; **143**(6): 404-414+I430. doi:10.7326/0003-4819-143-6-200509200-00005

5. Acosta J, Chinman M, Ebener P, Malone PS, Phillips A, Wilks A. Evaluation of a Whole-School Change Intervention: Findings from a Two-Year Cluster-Randomized Trial of the Restorative Practices Intervention. *J Youth Adolesc* 2019. doi:10.1007/s10964-019-01013-2

6. Acosta MC, Possemato K, Maisto SA, et al. Web-Delivered CBT Reduces Heavy Drinking in OEF-OIF Veterans in Primary Care With Symptomatic Substance Use and PTSD. *Behav Ther* 2017; **48**(2): 262-276. doi:10.1016/j.beth.2016.09.001

7. Adamson SJ, Sellman JD, Foulds JA, et al. A randomized trial of combined citalopram and naltrexone for nonabstinent outpatients with co-occurring alcohol dependence and major depression. *J Clin Psychopharmacol* 2015; **35**(2): 143-149. doi:10.1097/JCP.0000000000000287

8. Adlan AS, Chooi KY, Mat Adenan NA. Acupressure as adjuvant treatment for the inpatient management of nausea and vomiting in early pregnancy: A double-blind randomized controlled trial. *J Obstet Gynaecol Res* 2017; **43**(4): 662-668. doi:10.1111/jog.13269

9. Agley J, Jun M, Eldridge L, et al. Effects of ACT out! social issue theater on social-emotional competence and bullying in youth and adolescents: cluster randomized controlled trial. *JMIR Ment Heal* 2021; **8**(1). doi:10.2196/25860

10. Agras WS, Walsh BT, Fairburn CG, Wilson GT, Kraemer HC. A multicenter comparison of cognitive-behavioral therapy and interpersonal psychotherapy for bulimia nervosa. *Arch Gen Psychiatry* 2000; **57**(5): 459-466. doi:10.1001/archpsyc.57.5.459

11. Ahlen J, Hursti T, Tanner L, Tokay Z, Ghaderi A. Prevention of Anxiety and Depression in Swedish School Children: a Cluster-Randomized Effectiveness Study. *Prev Sci* 2018; **19**(2): 147-158. doi:10.1007/s11121-017-0821-1

12. Ahmadi J, Sefidfard Jahromi M. Ultrarapid Influence of Buprenorphine on Major Depression in Opioid-Dependent Patients: A Double Blind, Randomized Clinical Trial. *Subst Use Misuse* 2018; **53**(2): 286-289. doi:10.1080/10826084.2017.1400063

13. Aho AL, Tarkka MT, Åstedt-Kurki P, Sorvari L, Kaunonen M. Evaluating a bereavement follow-up intervention for grieving fathers and their experiences of support after the death of a child-a pilot study. *Death Stud* 2011; **35**(10): 879-904. doi:10.1080/07481187.2011.553318

14. Akbar I, Iqbal A, Al-Omiri MK. Flare-up rate in molars with periapical radiolucency in one-visit vs two-visit endodontic treatment. *J Contemp Dental Pract* 2013; **14**(3): 414-418. doi:10.5005/jp-journals-10024-1337

15. Alda M, Luciano JV, Andrés E, et al. Effectiveness of cognitive behaviour therapy for the treatment of catastrophisation in patients with fibromyalgia: A randomised controlled trial. *Arthritis Res Ther* 2011; **13**(5). doi:10.1186/ar3496

16. Aleyasin A, Saffarieh E, Torkamandi H, et al. Comparison of Efficacy of Granisetron and Promethazine in Control of Hyperemesis Gravidarum. *J Obstet Gynecol India* 2016; **66**(6): 409-414. doi:10.1007/s13224-015-0709-6

17. Allen JP, Narr RK, Nagel AG, Costello MA, Guskin K. The Connection Project: Changing the peer environment to improve outcomes for marginalized adolescents. *Dev Psychopathol* 2021; **33**(2): 647-657. doi:10.1017/S0954579419001731

18. Altamura AC, Mauri MC, Girardi T, Panetta B. Alcoholism and depression: A placebo controlled study with viloxazine. *INT J CLIN PHARMACOL RES* 1990; **10**(5): 293-298. doi:N

19. Altan L, Korkmaz N, Bingol Ü, Gunay B. Effect of Pilates Training on People With Fibromyalgia Syndrome: A Pilot Study. *Arch Phys Med Rehabil* 2009; **90**(12): 1983-1988. doi:10.1016/j.apmr.2009.06.021

20. Altintoprak AE, Zorlu N, Coskunol H, Akdeniz F, Kitapcioglu G. Effectiveness and tolerability of mirtazapine and amitriptyline in alcoholic patients with co-morbid depressive disorder: A randomized, double-blind study. *Hum Psychopharmacol* 2008; **23**(4): 313-319. doi:10.1002/hup.935

21. Amiel S, Beveridge S, Bradley C, et al. Training in flexible, intensive insulin management to enable dietary freedom in people with type 1 diabetes: Dose adjustment for normal eating (DAFNE) randomised controlled trial. *Br Med J* 2002; **325**(7367): 746-749. doi:10.1136/bmj.325.7367.746

22. Ammerman RT, Putnam FW, Altaye M, Stevens J, Teeters AR, Van Ginkel JB. A Clinical Trial of In-Home CBT for Depressed Mothers in Home Visitation. *Behav Ther* 2013; **44**(3): 359-372. doi:10.1016/j.beth.2013.01.002

23. Amris K, Wæhrens EE, Christensen R, Bliddal H, Danneskiold-Samsøe B. Interdisciplinary rehabilitation of patients with chronic widespread pain: Primary endpoint of the randomized, nonblinded, parallel-group IMPROvE trial. *Pain* 2014; **155**(7): 1356-1364. doi:10.1016/j.pain.2014.04.012

24. Andersen TE, Ravn SL, Armfield N, Maujean A, Requena SS, Sterling M. Trauma-focused cognitive behavioural therapy and exercise for chronic whiplash with comorbid posttraumatic stress disorder: A randomised controlled trial. *Pain* 2021; **162**(4): 1221-1232. doi:10.1097/j.pain.0000000000002117

25. Andersson G, Hesser H, Veilord A, et al. Randomised controlled non-inferiority trial with 3-year follow-up of internet-delivered versus face-to-face group cognitive behavioural therapy for depression. *Journal of Affective Disorders* 2013; **151**(3): 986-994. doi:10.1016/j.jad.2013.08.022

26. Andersson G, Waara J, Jonsson U, Malmaeus F, Carlbring P, Öst LG. Internet-based self-help versus one-session exposure in the treatment of spider phobia: A randomized controlled trial. *Cogn Behav Ther* 2009; **38**(2): 114-120. doi:10.1080/16506070902931326

27. Andersson G, Waara J, Jonsson U, Malmaeus F, Carlbring P, Öst LG. Internet-Based Exposure Treatment Versus One-Session Exposure Treatment of Snake Phobia: A Randomized Controlled Trial. *Cogn Behav Ther* 2013; **42**(4): 284-291. doi:10.1080/16506073.2013.844202

28. Andrews G, Davies M, Titov N. Effectiveness randomized controlled trial of face to face versus Internet cognitive behaviour therapy for social phobia. *AUST NEW ZEALAND J PSYCHIATRY* 2011; **45**(4): 337-340. doi:10.3109/00048674.2010.538840

29. Andrews R, Cooper AR, Montgomery AA, et al. Diet or diet plus physical activity versus usual care in patients with newly diagnosed type 2 diabetes: The Early ACTID randomised controlled trial. *Lancet* 2011; **378**(9786): 129-139. doi:10.1016/S0140-6736(11)60442-X

30. Anema JR, Steenstra IA, Bongers PM, et al. Multidisciplinary rehabilitation for subacute low back pain: Graded activity or workplace intervention or both? A randomized controlled trial. *Spine* 2007; **32**(3): 291-298. doi:10.1097/01.brs.0000253604.90039.ad

31. Ang DC, Kaleth AS, Bigatti S, et al. Research to encourage exercise for fibromyalgia (REEF): Use of motivational interviewing, outcomes from a randomized-controlled trial. *Clin J Pain* 2013; **29**(4): 296-304. doi:10.1097/AJP.0b013e318254ac76

32. Angeles RN, Cuenter D, McCarthy L, et al. Group interprofessional chronic pain management in the primary care setting: A pilot study of feasibility and effectiveness in a family health team in ontario. *Pain Res Manage* 2013; **18**(5): 237-242. doi:10.1155/2013/491279

33. Arends I, Van Der Klink JJL, Van Rhenen W, De Boer MR, Bültmann U. Prevention of recurrent sickness absence in workers with common mental disorders: Results of a cluster-randomised controlled trial. *Occupational and Environmental Medicine* 2014; **71**(1): 21-29. doi:10.1136/oemed-2013-101412

34. Arnarson EO, Craighead WE. Prevention of depression among Icelandic adolescents. *Behav Res Ther* 2009; **47**(7): 577-585. doi:10.1016/j.brat.2009.03.011

35. Arnarson EO, Craighead WE. Prevention of depression among Icelandic adolescents: A 12-month follow-up. *Behav Res Ther* 2011; **49**(3): 170-174. doi:10.1016/j.brat.2010.12.008

36. Arnold AC, Okamoto LE, Diedrich A, et al. Low-dose propranolol and exercise capacity in postural tachycardia syndrome a randomized study. *Neurology* 2013; **80**(21): 1927-1933. doi:10.1212/WNL.0b013e318293e310

37. Arnold LM, Clauw D, Wang F, Ahl J, Gaynor PJ, Wohlreich MM. Flexible dosed duloxetine in the treatment of fibromyalgia: A randomized, double-blind, placebo-controlled trial. *J Rheumatol* 2010; **37**(12): 2578-2586. doi:10.3899/jrheum.100365

38. Arnold LM, Goldenberg DL, Stanford SB, et al. Gabapentin in the treatment of fibromyalgia: A randomized, double-blind, placebo-controlled, multicenter trial. *Arthritis Rheum* 2007; **56**(4): 1336-1344. doi:10.1002/art.22457

39. Arnold LM, Russell IJ, Diri EW, et al. A 14-week, Randomized, Double-Blinded, Placebo-Controlled Monotherapy Trial of Pregabalin in Patients With Fibromyalgia. *J Pain* 2008; **9**(9): 792-805. doi:10.1016/j.jpain.2008.03.013

40. Arnold LM, Zhang S, Pangallo BA. Efficacy and safety of duloxetine 30 mg/d in patients with fibromyalgia: A randomized, double-blind, placebo-controlled study. *Clin J Pain* 2012; **28**(9): 775-781. doi:10.1097/AJP.0b013e3182510295

41. Arthur RA, Zenkner JE, d’Ornellas Pereira Júnior JC, Correia RT, Alves LS, Maltz M. Proximal carious lesions infiltration—a 3-year follow-up study of a randomized controlled clinical trial. *Clin Oral Invest* 2018; **22**(1): 469-474. doi:10.1007/s00784-017-2135-x

42. Asarnow JR, Berk MS, Bedics J, et al. Dialectical Behavior Therapy for Suicidal Self-Harming Youth: Emotion Regulation, Mechanisms, and Mediators. *J AM ACAD CHILD ADOLESC PSYCHIATRY* 2021; **60**(9): 1105-1115.e1104. doi:10.1016/j.jaac.2021.01.016

43. Asarnow JR, Hughes JL, Babeva KN, Sugar CA. Cognitive-Behavioral Family Treatment for Suicide Attempt Prevention: A Randomized Controlled Trial. *J AM ACAD CHILD ADOLESC PSYCHIATRY* 2017; **56**(6): 506-514. doi:10.1016/j.jaac.2017.03.015

44. Aspvall K, Andersson E, Melin K, et al. Effect of an Internet-Delivered Stepped-Care Program vs In-Person Cognitive Behavioral Therapy on Obsessive-Compulsive Disorder Symptoms in Children and Adolescents: A Randomized Clinical Trial. *JAMA* 2021; **325**(18): 1863-1873. doi:10.1001/jama.2021.3839

45. Assefi NP, Sherman KJ, Jacobsen C, Goldberg J, Smith WR, Buchwald D. A randomized clinical trial of acupuncture compared with sham acupuncture in fibromyalgia. *Ann Intern Med* 2005; **143**(1): 10-19+I-24. doi:10.7326/0003-4819-143-1-200507050-00005

46. Attia E, Haiman C, Timothy Walsh B, Flater SR. Does fluoxetine augment the inpatient treatment of anorexia nervosa? *AM J PSYCHIATRY* 1998; **155**(4): 548-551. doi:10.1176/ajp.155.4.548

47. Axelsson E, Andersson E, Ljótsson B, Björkander D, Hedman-Lagerlöf M, Hedman-Lagerlöf E. Effect of internet vs face-to-face cognitive behavior therapy for health anxiety: A randomized noninferiority clinical trial. *JAMA Psychiatry* 2020; **77**(9): 915-924. doi:10.1001/jamapsychiatry.2020.0940

48. Bachmann GA, Brown CS, Phillips NA, et al. Effect of gabapentin on sexual function in Vulvodynia: A randomized, placebo-controlled trial. *Obstet Gynecol Surv* 2019; **74**(2): 82-83. doi:10.1097/OGX.0000000000000643

49. Back SE, McCauley JL, Korte KJ, et al. A double-blind, randomized, controlled pilot trial of N-acetylcysteine in veterans with posttraumatic stress disorder and substance use disorders. *J CLIN PSYCHIATRY* 2016; **77**(11): e1439-e1446. doi:10.4088/JCP.15m10239

50. Bagger YZ, Tankó LB, Alexandersen P, Ravn P, Christiansen C. Alendronate has a residual effect on bone mass in postmenopausal Danish women up to 7 years after treatment withdrawal. *Bone* 2003; **33**(3): 301-307. doi:10.1016/S8756-3282(03)00112-1

51. Baker S, Sanders MR, Turner KMT, Morawska A. A randomized controlled trial evaluating a low-intensity interactive online parenting intervention, Triple P Online Brief, with parents of children with early onset conduct problems. *Behav Res Ther* 2017; **91**: 78-90. doi:10.1016/j.brat.2017.01.016

52. Bakker IM, Terluin B, van Marwijk HWJ, et al. A cluster-randomised trial evaluating an intervention for patients with stress-related mental disorders and sick leave in primary care. *PLoS Clin Trials* 2007; **2**(6): e26. doi:10.1371/journal.pctr.0020026

53. Banasiak SJ, Paxton SJ, Hay P. Guided self-help for bulimia nervosa in primary care: A randomized controlled trial. *Psychol Med* 2005; **35**(9): 1283-1294. doi:10.1017/S0033291705004769

54. Baptista AS, Villela AL, Jones A, Natour J. Effectiveness of dance in patients with fibromyalgia: A randomised, single-blind, controlled study. *Clin Exp Rheumatol* 2012; **30**(SUPPL.74): S18-S23. doi:N

55. Barnard ND, Cohen J, Jenkins DJA, et al. A low-fat vegan diet and a conventional diabetes diet in the treatment of type 2 diabetes: A randomized, controlled, 74-wk clinical trial. *Am J Clin Nutr* 2009; **89**(5): 1588S-1596S. doi:10.3945/ajcn.2009.26736H

56. Baron R, Mayoral V, Leijon G, Binder A, Steigerwald I, Serpell M. 5% Lidocaine medicated plaster versus pregabalin in post-herpetic neuralgia and diabetic polyneuropathy: An open-label, non-inferiority two-stage RCT study. *Curr Med Res Opin* 2009; **25**(7): 1663-1676. doi:10.1185/03007990903047880

57. Barrenengoa-Cuadra MJ, Muñoa-Capron-Manieux M, Fernández-Luco M, et al. Effectiveness of a structured group intervention based on pain neuroscience education for patients with fibromyalgia in primary care: A multicentre randomized open-label controlled trial. *Eur J Pain* 2021; **25**(5): 1137-1149. doi:10.1002/ejp.1738

58. Barreras González JE, Torres Peña R, Ruiz Torres J, Martínez Alfonso M, Brizuela Quintanilla R, Morera Pérez M. Endoscopic versus laparoscopic treatment for choledocholithiasis: a prospective randomized controlled trial. *Endosc Int Open* 2016; **4**(11): E1188-e1193. doi:10.1055/s-0042-116144

59. Barrett PM, Farrell LJ, Ollendick TH, Dadds M. Long-term outcomes of an Australian universal prevention trial of anxiety and depression symptoms in children and youth: An evaluation of the friends program. *J Clin Child Adolesc Psychol* 2006; **35**(3): 403-411. doi:10.1207/s15374424jccp3503_5

60. Bateman A, Constantinou MP, Fonagy P, Holzer S. Eight-year prospective follow-up of mentalization-based treatment versus structured clinical management for people with borderline personality disorder. *Pers Disord Theory Res Treat* 2021; **12**(4): 291-299. doi:10.1037/per0000422

61. Bateman A, Fonagy P. Effectiveness of partial hospitalization in the treatment of borderline personality disorder: A randomized controlled trial. *AM J PSYCHIATRY* 1999; **156**(10): 1563-1569. doi:10.1176/ajp.156.10.1563

62. Bateman A, Fonagy P. Treatment of borderline personality disorder with psychoanalytically oriented partial hospitalization: An 18-month follow-up. *AM J PSYCHIATRY* 2001; **158**(1): 36-42. doi:10.1176/appi.ajp.158.1.36

63. Bateman A, Fonagy P. 8-Year follow-up of patients treated for borderline personality disorder: Mentalization-based treatment versus treatment as usual. *AM J PSYCHIATRY* 2008; **165**(5): 631-638. doi:10.1176/appi.ajp.2007.07040636

64. Bateman A, Fonagy P. Randomized controlled trial of outpatient mentalization-based treatment versus structured clinical management for borderline personality disorder. *AM J PSYCHIATRY* 2009; **166**(12): 1355-1364. doi:10.1176/appi.ajp.2009.09040539

65. Batki SL, Pennington DL, Lasher B, et al. Topiramate Treatment of Alcohol Use Disorder in Veterans with Posttraumatic Stress Disorder: A Randomized Controlled Pilot Trial. *Alcohol Clin Exp Res* 2014; **38**(8): 2169-2177. doi:10.1111/acer.12496

66. Beardslee WR, Brent DA, Weersing VR, et al. Prevention of depression in at-risk adolescents: Longer-term effects. *JAMA Psychiatry* 2013; **70**(11): 1161-1170. doi:10.1001/jamapsychiatry.2013.295

67. Beardslee WR, Wright EJ, Gladstone TRG, Forbes P. Long-Term Effects From a Randomized Trial of Two Public Health Preventive Interventions for Parental Depression. *J Fam Psychol* 2007; **21**(4): 703-713. doi:10.1037/0893-3200.21.4.703

68. Beck E, Bo S, Jørgensen MS, et al. Mentalization-based treatment in groups for adolescents with borderline personality disorder: a randomized controlled trial. *J Child Psychol Psychiatry Allied Discip* 2020; **61**(5): 594-604. doi:10.1111/jcpp.13152

69. Bejerholm U, Larsson ME, Johanson S. Supported employment adapted for people with affective disorders—A randomized controlled trial. *Journal of Affective Disorders* 2017; **207**: 212-220. doi:10.1016/j.jad.2016.08.028

70. Bell KR, Fann JR, Brockway JA, et al. Telephone Problem Solving for Service Members with Mild Traumatic Brain Injury: A Randomized, Clinical Trial. *J Neurotrauma* 2017; **34**(2): 313-321. doi:10.1089/neu.2016.4444

71. Berenguel Senén A, Gadella Fernández A, Godoy López J, et al. Functional rehabilitation based on therapeutic exercise training in patients with postacute COVID syndrome (RECOVER). *Rev Esp Cardiol* 2024; **77**(2): 167-175. doi:10.1016/j.recesp.2023.06.010

72. Berger R, Benatov J, Cuadros R, VanNattan J, Gelkopf M. Enhancing resiliency and promoting prosocial behavior among Tanzanian primary-school students: A school-based intervention. *Transcult Psychiatry* 2018; **55**(6): 821-845. doi:10.1177/1363461518793749

73. Bergeron S, Binik YM, Khalifé S, et al. A randomized comparison of group cognitive-behavioral therapy, surface electromyographic biofeedback, and vestibulectomy in the treatment of dyspareunia resulting from vulvar vestibulitis. *Pain* 2001; **91**(3): 297-306. doi:10.1016/S0304-3959(00)00449-8

74. Bergeron S, Khalifé S, Dupuis MJ, McDuff P. A randomized clinical trial comparing group cognitive-behavioral therapy and a topical steroid for women with dyspareunia. *J Consult Clin Psychol* 2016; **84**(3): 259-268. doi:10.1037/ccp0000072

75. Bergeron S, Khalifé S, Glazer HI, Binik YM. Surgical and behavioral treatments for vestibulodynia: Two-and-one-half- year follow-up and predictors of outcome. *Obstet Gynecol* 2008; **111**(1): 159-166. doi:10.1097/01.AOG.0000295864.76032.a7

76. Bergh C, Brodin U, Lindberg G, Södersten P. Randomized controlled trial of a treatment for anorexia and bulimia nervosa. *Proc Natl Acad Sci U S A* 2002; **99**(14): 9486-9491. doi:10.1073/pnas.142284799

77. Bergström J, Andersson G, Ljótsson B, et al. Internet-versus group-administered cognitive behaviour therapy for panic disorder in a psychiatric setting: A randomised trial. *BMC Psychiatry* 2010; **10**. doi:10.1186/1471-244X-10-54

78. Berk MS, Gallop R, Asarnow JR, et al. Trajectories of Treatment Response and Nonresponse in Youth at High Risk for Suicide. *J AM ACAD CHILD ADOLESC PSYCHIATRY* 2022; **61**(9): 1119-1130. doi:10.1016/j.jaac.2022.01.010

79. Berkel C, Fu E, Carroll AJ, et al. Effects of the Family Check-Up 4 Health on Parenting and Child Behavioral Health: A Randomized Clinical Trial in Primary Care. *Prev Sci* 2021; **22**(4): 464-474. doi:10.1007/s11121-021-01213-y

80. Berkovits MD, O'Brien KA, Carter CG, Eyberg SM. Early Identification and Intervention for Behavior Problems in Primary Care: A Comparison of Two Abbreviated Versions of Parent-Child Interaction Therapy. *Behav Ther* 2010; **41**(3): 375-387. doi:10.1016/j.beth.2009.11.002

81. Bermejo-Martins E, Mujika A, Iriarte A, et al. Social and emotional competence as key element to improve healthy lifestyles in children: A randomized controlled trial. *J Adv Nurs* 2019; **75**(8): 1764-1781. doi:10.1111/jan.14024

82. Bernhardt MK, Southard KA, Batterson KD, Logan HL, Baker KA, Jakobsen JR. The effect of preemptive and/or postoperative ibuprofen therapy for orthodontic pain. *Am J Orthod Dentofacial Orthop* 2001; **120**(1): 20-27. doi:10.1067/mod.2001.115616

83. Bérubé S, Demers C, Bussière N, et al. Olfactory Training Impacts Olfactory Dysfunction Induced by COVID-19: A Pilot Study. *ORL* 2023; **85**(2): 57-66. doi:10.1159/000528188

84. Best KL, Kirby RL, Smith C, MacLeod DA. Wheelchair skills training for community-based manual wheelchair users: A randomized controlled trial. *Arch Phys Med Rehabil* 2005; **86**(12): 2316-2323. doi:10.1016/j.apmr.2005.07.300

85. Best KL, Miller WC, Huston G, Routhier F, Eng JJ. Pilot study of a peer-led wheelchair training program to improve self-efficacy using a manual wheelchair: A randomized controlled trial. *Arch Phys Med Rehabil* 2016; **97**(1): 37-44. doi:10.1016/j.apmr.2015.08.425

86. Beydoun A, Shaibani A, Hopwood M, Wan Y. Oxcarbazepine in painful diabetic neuropathy: Results of a dose-ranging study. *Acta Neurol Scand* 2006; **113**(6): 395-404. doi:10.1111/j.1600-0404.2006.00631.x

87. Bianchini V, Cofini V, Curto M, et al. Dialectical behaviour therapy (DBT) for forensic psychiatric patients: An Italian pilot study. *Crim Behav Ment Health* 2019; **29**(2): 122-130. doi:10.1002/cbm.2102

88. Bierman KL, Domitrovich CE, Nix RL, et al. Promoting academic and social-emotional school readiness: The head start REDI program. *Child Dev* 2008; **79**(6): 1802-1817. doi:10.1111/j.1467-8624.2008.01227.x

89. Bierman KL, Heinrichs BS, Welsh JA, Nix RL. Reducing adolescent psychopathology in socioeconomically disadvantaged children with a preschool intervention: A randomized controlled trial. *AM J PSYCHIATRY* 2021; **178**(4): 305-312. doi:10.1176/appi.ajp.2020.20030343

90. Bierman KL, Nix RL, Heinrichs BS, et al. Effects of Head Start REDI on Children's Outcomes 1 Year Later in Different Kindergarten Contexts. *Child Dev* 2014; **85**(1): 140-159. doi:10.1111/cdev.12117

91. Biesbroeck R, Bril V, Hollander P, et al. A double-blind comparison of topical capsaicin and oral amitriptyline in painful diabetic neuropathy. *ADV THER* 1995; **12**(2): 111-120. doi:N

92. Bjureberg J, Ojala O, Hesser H, et al. Effect of Internet-Delivered Emotion Regulation Individual Therapy for Adolescents with Nonsuicidal Self-Injury Disorder: A Randomized Clinical Trial. *JAMA Netw Open* 2023; **6**(7). doi:10.1001/jamanetworkopen.2023.22069

93. Björkelund C, Svenningsson I, Hange D, et al. Clinical effectiveness of care managers in collaborative care for patients with depression in Swedish primary health care: A pragmatic cluster randomized controlled trial. *BMC Fam Pract* 2018; **19**(1). doi:10.1186/s12875-018-0711-z

94. Bjørndal L, Fransson H, Bruun G, et al. Randomized Clinical Trials on Deep Carious Lesions: 5-Year Follow-up. *J Dent Res* 2017; **96**(7): 747-753. doi:10.1177/0022034517702620

95. Bjørndal L, Reit C, Bruun G, et al. Treatment of deep caries lesions in adults: randomized clinical trials comparing stepwise vs. direct complete excavation, and direct pulp capping vs. partial pulpotomy. *Eur J Oral Sci* 2010; **118**(3): 290-297. doi:10.1111/j.1600-0722.2010.00731.x

96. Björneklett HG, Rosenblad A, Lindemalm C, et al. A randomized controlled trial of support group intervention after breast cancer treatment: Results on sick leave, health care utilization and health economy. *Acta Oncol* 2013; **52**(1): 38-47. doi:10.3109/0284186X.2012.734921

97. Bjørseth Å, Wichstrøm L. Effectiveness of Parent-Child interaction therapy (PCIT) in the treatment of young children's behavior problems. A randomized controlled study. *PLoS ONE* 2016; **11**(9). doi:10.1371/journal.pone.0159845

98. Bleker LS, Milgrom J, Sexton-Oates A, et al. Exploring the effect of antenatal depression treatment on children's epigenetic profiles: Findings from a pilot randomized controlled trial. *Clin Epigenetics* 2019; **11**(1). doi:10.1186/s13148-019-0616-2

99. Bodenmann G, Cina A, Ledermann T, Sanders MR. The efficacy of the Triple P-Positive Parenting Program in improving parenting and child behavior: A comparison with two other treatment conditions. *Behav Res Ther* 2008; **46**(4): 411-427. doi:10.1016/j.brat.2008.01.001

100. Boerma D, Rauws EAJ, Keulemans YCA, et al. Wait-and-see policy or laparoscopic cholecystectomy after endoscopic sphincterotomy for bile-duct stones: A randomised trial. *Lancet* 2002; **360**(9335): 761-765. doi:10.1016/S0140-6736(02)09896-3

101. Bondok RS, El Sharnouby NM, Eid HE, Abd Elmaksoud AM. Pulsed steroid therapy is an effective treatment for intractable hyperemesis gravidarum. *Crit Care Med* 2006; **34**(11): 2781-2783. doi:10.1097/01.CCM.0000242156.15757.70

102. Book SW, Thomas SE, Randall PK, Randall CL. Paroxetine reduces social anxiety in individuals with a co-occurring alcohol use disorder. *J Anxiety Disord* 2008; **22**(2): 310-318. doi:10.1016/j.janxdis.2007.03.001

103. Botelho MG, Chan AWK, Leung NCH, Lam WYH. Long-term evaluation of cantilevered versus fixed–fixed resin-bonded fixed partial dentures for missing maxillary incisors. *J Dent* 2016; **45**: 59-66. doi:10.1016/j.jdent.2015.12.006

104. Botella C, Gallego MJ, Garcia-Palacios A, et al. An internet-based self-help treatment for fear of public speaking: A controlled trial. *Cyberpsychol Behav Soc Networking* 2010; **13**(4): 407-421. doi:10.1089/cyber.2009.0224

105. Bowen ME, Cavanaugh KL, Wolff K, et al. The diabetes nutrition education study randomized controlled trial: A comparative effectiveness study of approaches to nutrition in diabetes self-management education. *Patient Educ Couns* 2016; **99**(8): 1368-1376. doi:10.1016/j.pec.2016.03.017

106. Boyle J, Eriksson MEV, Gribble L, et al. Randomized, placebo-controlled comparison of amitriptyline, duloxetine, and pregabalin in patients with chronic diabetic peripheral neuropathic pain: Impact on pain, polysomnographic sleep, daytime functioning, and quality of life. *Diabetes Care* 2012; **35**(12): 2451-2458. doi:10.2337/dc12-0656

107. Bradshaw CP, Waasdorp TE, Leaf PJ. Effects of school-wide positive behavioral interventions and supports on child behavior problems. *Pediatrics* 2012; **130**(5): e1136-e1145. doi:10.1542/peds.2012-0243

108. Brady KT, Sonne S, Anton RF, Randall CL, Back SE, Simpson K. Sertraline in the treatment of co-occurring alcohol dependence and posttraumatic stress disorder. *Alcohol Clin Exp Res* 2005; **29**(3): 395-401. doi:10.1097/01.ALC.0000156129.98265.57

109. Brady KT, Sonne SC, Malcolm RJ, et al. Carbamazepine in the treatment of cocaine dependence: Subtyping by affective disorder. *Exp Clin Psychopharmacol* 2002; **10**(3): 276-285. doi:10.1037/1064-1297.10.3.276

110. Brattberg G. Internet-based rehabilitation for individuals with chronic pain and burnout II: A long-term follow-up. *Int J Rehabil Res* 2007; **30**(3): 231-234. doi:10.1097/MRR.0b013e32829fa545

111. Brehm BJ, Lattin BL, Summer SS, et al. One-year comparison of a high-monounsaturated fat diet with a high-carbohydrate diet in type 2 diabetes. *Diabetes Care* 2009; **32**(2): 215-220. doi:10.2337/dc08-0687

112. Brendbekken R, Eriksen HR, Grasdal A, Harris A, Hagen EM, Tangen T. Return to Work in Patients with Chronic Musculoskeletal Pain: Multidisciplinary Intervention Versus Brief Intervention: A Randomized Clinical Trial. *J Occup Rehabil* 2017; **27**(1): 82-91. doi:10.1007/s10926-016-9634-5

113. Brendbekken R, Harris A, Ursin H, Eriksen HR, Tangen T. Multidisciplinary Intervention in Patients with Musculoskeletal Pain: a Randomized Clinical Trial. *Int J Behav Med* 2016; **23**(1): 1-11. doi:10.1007/s12529-015-9486-y

114. Brendbekken R, Vaktskjold A, Harris A, Tangen T. Predictors of return-to-work in patients with chronic musculoskeletal pain: A randomized clinical trial. *J Rehabil Med* 2018; **50**(2): 193-199. doi:10.2340/16501977-2296

115. Brenner LA, Forster JE, Hoffberg AS, et al. Window to Hope: A Randomized Controlled Trial of a Psychological Intervention for the Treatment of Hopelessness among Veterans with Moderate to Severe Traumatic Brain Injury. *J Head Trauma Rehabil* 2018; **33**(2): E64-E73. doi:10.1097/HTR.0000000000000351

116. Brenninkmeijer V, Blonk RWB. The effectiveness of the JOBS program among the long-term unemployed: A randomized experiment in the Netherlands. *Health Promot Int* 2012; **27**(2): 220-229. doi:10.1093/heapro/dar033

117. Brent DA, Brunwasser SM, Hollon SD, et al. Effect of a cognitive-behavioral prevention program on depression 6 years after implementation among at-risk adolescents: A randomized clinical trial. *JAMA Psychiatry* 2015; **72**(11): 1110-1118. doi:10.1001/jamapsychiatry.2015.1559

118. Brienza DM, Karg PE, Bertolet M, et al. A Randomized Clinical Trial of Wheeled Mobility for Pressure Injury Prevention and Better Function. *J Am Geriatr Soc* 2018; **66**(9): 1752-1759. doi:10.1111/jgs.15495

119. Brinkworth GD, Luscombe-Marsh ND, Thompson CH, et al. Long-term effects of very low-carbohydrate and high-carbohydrate weight-loss diets on psychological health in obese adults with type 2 diabetes: randomized controlled trial. *J Intern Med (GBR)* 2016; **280**(4): 388-397. doi:10.1111/joim.12501

120. Broderick JE, Junghaenel DU, Schwartz JE. Written emotional expression produces health benefits in fibromyalgia patients. *Psychosom Med* 2005; **67**(2): 326-334. doi:10.1097/01.psy.0000156933.04566.bd

121. Brotman LM, Dawson-Mcclure S, Gouley KK, McGuire K, Burraston B, Bank L. Older siblings benefit from a family-based preventive intervention for preschoolers at risk for conduct problems. *J Fam Psychol* 2005; **19**(4): 581-591. doi:10.1037/0893-3200.19.4.581

122. Brotman LM, Gouley KK, Huang KY, et al. Preventive intervention for preschoolers at high risk for antisocial behavior: Long-term effects on child physical aggression and parenting practices. *J Clin Child Adolesc Psychol* 2008; **37**(2): 386-396. doi:10.1080/15374410801955813

123. Brotto LA, Bergeron S, Zdaniuk B, Basson R. Mindfulness and cognitive behavior therapy for provoked vestibulodynia: Mediators of treatment outcome and long-term effects. *J Consult Clin Psychol* 2020; **88**(1): 48-64. doi:10.1037/ccp0000473

124. Brotto LA, Bergeron S, Zdaniuk B, et al. A Comparison of Mindfulness-Based Cognitive Therapy Vs Cognitive Behavioral Therapy for the Treatment of Provoked Vestibulodynia in a Hospital Clinic Setting. *J Sex Med* 2019; **16**(6): 909-923. doi:10.1016/j.jsxm.2019.04.002

125. Brouwers EPM, Tiemens BG, Terluin B, Verhaak PFM. Effectiveness of an intervention to reduce sickness absence in patients with emotional distress or minor mental disorders: a randomized controlled effectiveness trial. *Gen Hosp Psychiatry* 2006; **28**(3): 223-229. doi:10.1016/j.genhosppsych.2006.02.005

126. Brown A, Dornhorst A, McGowan B, et al. Low-energy total diet replacement intervention in patients with type 2 diabetes mellitus and obesity treated with insulin: A randomized trial. *BMJ Open Diabetes Res Care* 2020; **8**(1). doi:10.1136/bmjdrc-2019-001012

127. Brown CS, Bachmann GA, Wan J, Foster DC. Gabapentin for the treatment of vulvodynia: A randomized controlled trial. *Obstet Gynecol* 2018; **131**(6): 1000-1007. doi:10.1097/AOG.0000000000002617

128. Brown ES, Garza M, Carmody TJ. A randomized, double-blind, placebo-controlled add-on trial of quetiapine in outpatients with bipolar disorder and alcohol use disorders. *J CLIN PSYCHIATRY* 2008; **69**(5): 701-705. doi:10.4088/JCP.v69n0502

129. Brown ES, Gorman AR, Hynan LS. A randomized, placebo-controlled trial of citicoline add-on therapy in outpatients with bipolar disorder and cocaine dependence. *J Clin Psychopharmacol* 2007; **27**(5): 498-502. doi:10.1097/JCP.0b013e31814db4c4

130. Brown ES, Sunderajan P, Hu LT, Sowell SM, Carmody TJ. A randomized, double-blind, placebo-controlled, trial of lamotrigine therapy in bipolar disorder, depressed or mixed phase and cocaine dependence. *Neuropsychopharmacology* 2012; **37**(11): 2347-2354. doi:10.1038/npp.2012.90

131. Brown ES, Todd JP, Hu LT, et al. A randomized, double-blind, placebo-controlled trial of citicoline for cocaine dependence in bipolar i disorder. *AM J PSYCHIATRY* 2015; **172**(10): 1014-1021. doi:10.1176/appi.ajp.2015.14070857

132. Brunette MF, Correll CU, O'Malley SS, et al. Olanzapine plus samidorphan (ALKS 3831) in schizophrenia and comorbid alcohol use disorder: A phase 2, randomized clinical trial. *J CLIN PSYCHIATRY* 2020; **81**(2). doi:10.4088/JCP.19m12786

133. Brunette MF, Dawson R, O'Keefe CD, et al. A randomized trial of clozapine versus other antipsychotics for cannabis use disorder in patients with schizophrenia. *J Dual Diagn* 2011; **7**(1-2): 50-63. doi:10.1080/15504263.2011.570118

134. Bryant RA, Moulds M, Guthrie R, Nixon RDV. Treating acute stress disorder following mild traumatic brain injury. *AM J PSYCHIATRY* 2003; **160**(3): 585-587. doi:10.1176/appi.ajp.160.3.585

135. Bullard L, Wachlarowicz M, DeLeeuw J, et al. Effects of the oregon model of parent management training (PMTO) on marital adjustment in new stepfamilies: A randomized trial. *J Fam Psychol* 2010; **24**(4): 485-496. doi:10.1037/a0020267

136. Bullen C, Howe C, Laugesen M, et al. Electronic cigarettes for smoking cessation: A randomised controlled trial. *Lancet* 2013; **382**(9905): 1629-1637. doi:10.1016/S0140-6736(13)61842-5

137. Burckhardt R, Manicavasagar V, Batterham PJ, Miller LM, Talbot E, Lum A. A web-based adolescent positive psychology program in schools: Randomized controlled trial. *J Med Internet Res* 2015; **17**(7). doi:10.2196/jmir.4329

138. Burger H, Verbeek T, Aris-Meijer JL, et al. Effects of psychological treatment of mental health problems in pregnant women to protect their offspring: Randomised controlled trial. *Br J Psychiatry* 2020; **216**(4): 182-188. doi:10.1192/bjp.2019.260

139. Burns A, O'Mahen H, Baxter H, et al. A pilot randomised controlled trial of cognitive behavioural therapy for antenatal depression. *BMC Psychiatry* 2013; **13**. doi:10.1186/1471-244X-13-33

140. Busch H, Bodin L, Bergström G, Jensen IB. Patterns of sickness absence a decade after pain-related multidisciplinary rehabilitation. *Pain* 2011; **152**(8): 1727-1733. doi:10.1016/j.pain.2011.02.004

141. Butler S, Baruch G, Hickey N, Fonagy P. A randomized controlled trial of multisystemic therapy and a statutory therapeutic intervention for young offenders. *J AM ACAD CHILD ADOLESC PSYCHIATRY* 2011; **50**(12): 1220-1235.e1222. doi:10.1016/j.jaac.2011.09.017

142. Byatt N, Moore Simas TA, Biebel K, et al. PRogram In Support of Moms (PRISM): a pilot group randomized controlled trial of two approaches to improving depression among perinatal women. *J Psychosom Obstet Gynecol* 2018; **39**(4): 297-306. doi:10.1080/0167482X.2017.1383380

143. Bültmann U, Sherson D, Olsen J, Hansen CL, Lund T, Kilsgaard J. Coordinated and tailored work rehabilitation: A randomized controlled trial with economic evaluation undertaken with workers on sick leave due to musculoskeletal disorders. *J Occup Rehabil* 2009; **19**(1): 81-93. doi:10.1007/s10926-009-9162-7

144. Byrne S, Wade T, Hay P, et al. A randomised controlled trial of three psychological treatments for anorexia nervosa. *Psychol Med* 2017; **47**(16): 2823-2833. doi:10.1017/S0033291717001349

145. Cachelin FM, Gil-Rivas V, Palmer B, et al. Randomized controlled trial of a culturally-adapted program for latinas with binge eating. *Psychol Serv* 2019; **16**(3): 504-512. doi:10.1037/ser0000182

146. Caillon J, Grall-Bronnec M, Perrot B, et al. Effectiveness of At-Risk Gamblers’ Temporary Self-Exclusion from Internet Gambling Sites. *J Gambl Stud* 2019; **35**(2): 601-615. doi:10.1007/S10899-018-9782-Y

147. Calandre EP, Rodriguez-Claro ML, Rico-Villademoros F, Vilchez JS, Hidalgo J, Delgado-Rodriguez A. Effects of pool-based exercise in fibromyalgia symptomatology and sleep quality: A prospective randomized comparison between stretching and Tai Chi. *Clin Exp Rheumatol* 2009; **27**(5 SUPPL. 56): S13-S20. doi:N

148. Calner T, Nordin C, Eriksson MK, Nyberg L, Gard G, Michaelson P. Effects of a self-guided, web-based activity programme for patients with persistent musculoskeletal pain in primary healthcare: A randomized controlled trial. *Eur J Pain* 2017; **21**(6): 1110-1120. doi:10.1002/ejp.1012

149. Calvo-Paniagua J, Díaz-Arribas MJ, Valera-Calero JA, et al. Educational, Exercise, and Occupational Therapy-Based Telerehabilitation Program Versus "Wait-and-See" for Improving Self-perceived Exertion in Patients With Post-COVID Fatigue and Dyspnea: A Randomized Clinical Trial. *Am J Phys Med Rehabil* 2024; **103**(9): 797-804. doi:10.1097/phm.0000000000002441

150. Capin JJ, Jolley SE, Morrow M, et al. Safety, feasibility and initial efficacy of an app-facilitated telerehabilitation (AFTER) programme for COVID-19 survivors: A pilot randomised study. *BMJ Open* 2022; **12**(7). doi:10.1136/bmjopen-2022-061285

151. Cardemil EV, Reivich KJ, Beevers CG, Seligman MEP, James J. The prevention of depressive symptoms in low-income, minority children: Two-year follow-up. *Behav Res Ther* 2007; **45**(2): 313-327. doi:10.1016/j.brat.2006.03.010

152. Cardi V, Albano G, Ambwani S, et al. A randomised clinical trial to evaluate the acceptability and efficacy of an early phase, online, guided augmentation of outpatient care for adults with anorexia nervosa. *Psychol Med* 2020; **50**(15): 2610-2621. doi:10.1017/S0033291719002824

153. Carlbring P, Nilsson-Ihrfelt E, Waara J, et al. Treatment of panic disorder: Live therapy vs. self-help via the Internet. *Behav Res Ther* 2005; **43**(10): 1321-1333. doi:10.1016/j.brat.2004.10.002

154. Carlsson CPO, Axemo P, Bodin A, et al. Manual acupuncture reduces hyperemesis gravidarum: A placebo-controlled, randomized, single-blind, crossover study. *J Pain Symptom Manage* 2000; **20**(4): 273-279. doi:10.1016/S0885-3924(00)00185-8

155. Carlsson L, Englund L, Hallqvist J, Wallman T. Early multidisciplinary assessment was associated with longer periods of sick leave: A randomized controlled trial in a primary health care centre. *Scand J Prim Health Care* 2013; **31**(3): 141-146. doi:10.3109/02813432.2013.811943

156. Carlyle D, Green R, Inder M, et al. A Randomized-Controlled Trial of Mentalization-Based Treatment Compared With Structured Case Management for Borderline Personality Disorder in a Mainstream Public Health Service. *Front Psychiatry* 2020; **11**: 561916. doi:10.3389/fpsyt.2020.561916

157. Carpenter KM, Brooks AC, Vosburg SK, Nunes EV. The effect of sertraline and environmental context on treating depression and illicit substance use among methadone maintained opiate dependent patients: A controlled clinical trial. *Drug Alcohol Depend* 2004; **74**(2): 123-134. doi:10.1016/j.drugalcdep.2003.11.015

158. Carpenter MJ, Heckman BW, Wahlquist AE, et al. A naturalistic, randomized pilot trial of e-cigarettes: Uptake, exposure, and behavioral effects. *Cancer Epidemiol Biomarkers Prev* 2017; **26**(12): 1795-1803. doi:10.1158/1055-9965.EPI-17-0460

159. Carrard I, Crépin C, Rouget P, Lam T, Golay A, Van der Linden M. Randomised controlled trial of a guided self-help treatment on the Internet for binge eating disorder. *Behav Res Ther* 2011; **49**(8): 482-491. doi:10.1016/j.brat.2011.05.004

160. Carson JW, Carson KM, Jones KD, Bennett RM, Wright CL, Mist SD. A pilot randomized controlled trial of the Yoga of Awareness program in the management of fibromyalgia. *Pain* 2010; **151**(2): 530-539. doi:10.1016/j.pain.2010.08.020

161. Carson JW, Carson KM, Jones KD, Mist SD, Bennett RM. Follow-up of yoga of awareness for fibromyalgia: Results at 3 months and replication in the wait-list group. *Clin J Pain* 2012; **28**(9): 804-813. doi:10.1097/AJP.0b013e31824549b5

162. Carter FA, Jordan J, McIntosh VVW, et al. The long-term efficacy of three psychotherapies for anorexia nervosa: A randomized, controlled trial. *Int J Eating Disord* 2011; **44**(7): 647-654. doi:10.1002/eat.20879

163. Carter GL, Willcox CH, Lewin TJ, Conrad AM, Bendit N. Hunter DBT project: Randomized controlled trial of dialectical behaviour therapy in women with borderline personality disorder. *AUST NEW ZEALAND J PSYCHIATRY* 2010; **44**(2): 162-173. doi:10.3109/00048670903393621

164. Carter JC, Fairburn CG. Cognitive-behavioral self-help for binge eating disorder: A controlled effectiveness study. *J Consult Clin Psychol* 1998; **66**(4): 616-623. doi:10.1037/0022-006X.66.4.616

165. Carter S, Clifton PM, Keogh JB. Effect of Intermittent Compared With Continuous Energy Restricted Diet on Glycemic Control in Patients With Type 2 Diabetes: A Randomized Noninferiority Trial. *JAMA Netw Open* 2018; **1**(3): e180756. doi:10.1001/jamanetworkopen.2018.0756

166. Carter S, Clifton PM, Keogh JB. The effect of intermittent compared with continuous energy restriction on glycaemic control in patients with type 2 diabetes: 24-month follow-up of a randomised noninferiority trial. *Diabetes Res Clin Pract* 2019; **151**: 11-19. doi:10.1016/j.diabres.2019.03.022

167. Castel A, Cascón R, Padrol A, Sala J, Rull M. Multicomponent cognitive-behavioral group therapy with hypnosis for the treatment of fibromyalgia: Long-Term outcome. *J Pain* 2012; **13**(3): 255-265. doi:10.1016/j.jpain.2011.11.005

168. Castel A, Castro S, Fontova R, et al. Body mass index and response to a multidisciplinary treatment of fibromyalgia. *Rheumatol Int* 2015; **35**(2): 303-314. doi:10.1007/s00296-014-3096-x

169. Castro-Sánchez AM, Matarán-Pearrocha GA, Granero-Molina J, Aguilera-Manrique G, Quesada-Rubio JM, Moreno-Lorenzo C. Benefits of massage-myofascial release therapy on pain, anxiety, quality of sleep, depression, and quality of life in patients with fibromyalgia. *Evid-Based Complement Altern Med* 2011; **2011**(1). doi:10.1155/2011/561753

170. Castro-Sánchez AM, Matarán-Peñarrocha GA, Arroyo-Morales M, Saavedra-Hernández M, Fernández-Sola C, Moreno-Lorenzo C. Effects of myofascial release techniques on pain, physical function, and postural stability in patients with fibromyalgia: A randomized controlled trial. *Clin Rehabil* 2011; **25**(9): 800-813. doi:10.1177/0269215511399476

171. Cederberg M, Alsén S, Ali L, et al. Effects of a Person-Centered eHealth Intervention for Patients on Sick Leave Due to Common Mental Disorders (PROMISE Study): Open Randomized Controlled Trial. *JMIR Ment Heal* 2022; **9**(3): e30966. doi:10.2196/30966

172. Cejudo J, Losada L, Feltrero R. Promoting social and emotional learning and subjective well‐being: Impact of the “aislados” intervention program in adolescents. *Int J Environ Res Public Health* 2020; **17**(2). doi:10.3390/ijerph17020609

173. Celio MA, Lisman SA. Examining the efficacy of a personalized normative feedback intervention to reduce college student gambling. *J Am Coll Health* 2014; **62**(3): 154-164. doi:10.1080/07448481.2013.865626

174. Chan AWK, Barnes IE. A prospective study of cantilever resin-bonded bridges: An intial report. *Aust Dent J* 2000; **45**(1): 31-36. doi:10.1111/j.1834-7819.2000.tb00239.x

175. Chen E, Touyz SW, Beumont PJV, et al. Comparison of group and individual cognitive-behavioral therapy for patients with bulimia nervosa. *Int J Eating Disord* 2003; **33**(3): 241-254. doi:10.1002/eat.10137

176. Chen EY, Cacioppo J, Fettich K, et al. An adaptive randomized trial of dialectical behavior therapy and cognitive behavior therapy for binge-eating. *Psychol Med* 2017; **47**(4): 703-717. doi:10.1017/S0033291716002543

177. Chen Y, Liu C, Wang T, et al. Efficacy and safety of Bufei Huoxue capsules in the management of convalescent patients with COVID-19 infection: A multicentre, double-blind, and randomised controlled trial. *J Ethnopharmacol* 2022; **284**. doi:10.1016/j.jep.2021.114830

178. Chestnutt IG, Playle R, Hutchings S, et al. Fissure Seal or Fluoride Varnish? A Randomized Trial of Relative Effectiveness. *J Dent Res* 2017; **96**(7): 754-761. doi:10.1177/0022034517702094

179. Cheyne H, Dunlop A, Shields N, Mathers AM. A randomised controlled trial of admission electronic fetal monitoring in normal labour. *Midwifery* 2003; **19**(3): 221-229. doi:10.1016/S0266-6138(03)00020-2

180. Chiapa A, Smith JD, Kim H, Dishion TJ, Shaw DS, Wilson MN. The trajectory of fidelity in a multiyear trial of the family check-up predicts change in child problem behavior. *J Consult Clin Psychol* 2015; **83**(5): 1006-1011. doi:10.1037/ccp0000034

181. Chu JTW, Bullen P, Farruggia SP, Dittman CK, Sanders MR. Parent and Adolescent Effects of a Universal Group Program for the Parenting of Adolescents. *Prev Sci* 2015; **16**(4): 609-620. doi:10.1007/s11121-014-0516-9

182. Chung TWH, Zhang H, Wong FKC, et al. A Pilot Study of Short-Course Oral Vitamin A and Aerosolised Diffuser Olfactory Training for the Treatment of Smell Loss in Long COVID. *Brain Sci* 2023; **13**(7). doi:10.3390/brainsci13071014

183. Ciraulo DA, Barlow DH, Gulliver SB, et al. The effects of venlafaxine and cognitive behavioral therapy alone and combined in the treatment of co-morbid alcohol use-anxiety disorders. *Behav Res Ther* 2013; **51**(11): 729-735. doi:10.1016/j.brat.2013.08.003

184. Ciraulo DA, Knapp C, Rotrosen J, et al. Nefazodone treatment of cocaine dependence with comorbid depressive symptoms. *Addiction* 2005; **100**(SUPPL. 1): 23-31. doi:10.1111/j.1360-0443.2005.00984.x

185. Clarke AM, Bunting B, Barry MM. Evaluating the implementation of a school-based emotional well-being programme: A cluster randomized controlled trial of Zippy's Friends for children in disadvantaged primary schools. *Health Educ Res* 2014; **29**(5): 786-798. doi:10.1093/her/cyu047

186. Clarke GN, Hawkins W, Murphy M, Sheeber LB, Lewinsohn PM, Seeley JR. Targeted Prevention of Unipolar Depressive Disorder in an At-Risk Sample of High School Adolescents: A Randomized Trial of a Group Cognitive Intervention. *J AM ACAD CHILD ADOLESC PSYCHIATRY* 1995; **34**(3): 312-321. doi:10.1097/00004583-199503000-00016

187. Clarke GN, Hornbrook M, Lynch F, et al. A randomized trial of a group cognitive intervention for preventing depression in adolescent offspring of depressed parents. *Arch Gen Psychiatry* 2001; **58**(12): 1127-1134. doi:10.1001/archpsyc.58.12.1127

188. Coelho V, Sousa V, Raimundo R, Figueira A. The impact of a Portuguese middle school social-emotional learning program. *Health Promot Int* 2017; **32**(2): 292-300. doi:10.1093/heapro/dav064

189. Coelho VA, Brás P, Matsopoulos A. Differential Effectiveness Of An Elementary School Social And Emotional Learning Program During Middle School Transition In Portugal. *Sch Psychol* 2021; **36**(6): 475-482. doi:10.1037/spq0000454

190. Coelho VA, Marchante M, Jimerson SR. Promoting a Positive Middle School Transition: A Randomized-Controlled Treatment Study Examining Self-Concept and Self-Esteem. *J Youth Adolesc* 2017; **46**(3): 558-569. doi:10.1007/s10964-016-0510-6

191. Coffin ST, Black BK, Biaggioni I, et al. Desmopressin acutely decreases tachycardia and improves symptoms in the postural tachycardia syndrome. *Heart Rhythm* 2012; **9**(9): 1484-1490. doi:10.1016/j.hrthm.2012.05.002

192. Cohen JA, Mannarino AP. A treatment outcome study for sexually abused preschool children: Initial findings. *J AM ACAD CHILD ADOLESC PSYCHIATRY* 1996; **35**(1): 42-50. doi:10.1097/00004583-199601000-00011

193. Comer JS, Furr JM, Miguel EM, et al. Remotely delivering real-time parent training to the home: An initial randomized trial of Internet-delivered parent-child interaction therapy (I-PCIT). *J Consult Clin Psychol* 2017; **85**(9): 909-917. doi:10.1037/ccp0000230

194. Conduct Problems Prevention Research G. The Effects of a Multiyear Universal Social-Emotional Learning Program: The Role of Student and School Characteristics. *J Consult Clin Psychol* 2010; **78**(2): 156-168. doi:10.1037/a0018607

195. Connell AM, Dishion TJ. Long-Term Effects of the Family Check-Up in Public Secondary School on Diagnosed Major Depressive Disorder in Adulthood. *J Youth Adolesc* 2017; **46**(3): 570-581. doi:10.1007/s10964-016-0482-6

196. Connell AM, Dishion TJ, Yasui M, Kavanagh K. An Adaptive Approach to Family Intervention: Linking Engagement in Family-Centered Intervention to Reductions in Adolescent Problem Behavior. *J Consult Clin Psychol* 2007; **75**(4): 568-579. doi:10.1037/0022-006X.75.4.568

197. Connell AM, McKillop HN, Dishion TJ. Long-Term Effects of the Family Check-Up in Early Adolescence on Risk of Suicide in Early Adulthood. *Suicide Life-Threat Behav* 2016; **46**: S15-S22. doi:10.1111/sltb.12254

198. Cornelius JR, Salloum IM, Ehler JG, et al. Fluoxetine in depressed alcoholics: A double-blind, placebo-controlled trial. *Arch Gen Psychiatry* 1997; **54**(8): 700-705. doi:10.1001/archpsyc.1997.01830200024004

199. Cottrell DJ, Wright-Hughes A, Collinson M, et al. Effectiveness of systemic family therapy versus treatment as usual for young people after self-harm: a pragmatic, phase 3, multicentre, randomised controlled trial. *Lancet Psychiatry* 2018; **5**(3): 203-216. doi:10.1016/S2215-0366(18)30058-0

200. Cottrell DJ, Wright-Hughes A, Collinson M, et al. A pragmatic randomised controlled trial and economic evaluation of family therapy versus treatment as usual for young people seen after second or subsequent episodes of self-harm: The self-harm intervention – family therapy (shift) trial. *Health Technol Assess* 2018; **22**(12): 1-222. doi:10.3310/hta22120

201. Cottrell DJ, Wright-Hughes A, Eisler I, et al. Longer-term effectiveness of systemic family therapy compared with treatment as usual for young people after self-harm: An extended follow up of pragmatic randomised controlled trial. *EClinicalMedicine* 2020; **18**: 100246. doi:10.1016/j.eclinm.2019.100246

202. Crean HF, Johnson DB. Promoting Alternative Thinking Strategies (PATHS) and Elementary School Aged Children's Aggression: Results from a Cluster Randomized Trial. *Am J Community Psychol* 2013; **52**(1-2): 56-72. doi:10.1007/s10464-013-9576-4

203. Cunningham CE, Boyle M. Large Group Community‐Based Parenting Programs for Families of Preschoolers at Risk for Disruptive Behaviour Disorders: Utilization, Cost Effectiveness, and Outcome. *Journal of Child Psychology and Psychiatry* 1995; **36**(7): 1141-1159. doi:10.1111/j.1469-7610.1995.tb01362.x

204. Cunningham JA, Hodgins DC, Toneatto T, Murphy M. A randomized controlled trial of a personalized feedback intervention for problem gamblers. *PLoS ONE* 2012; **7**(2). doi:10.1371/journal.pone.0031586

205. Cunningham RM, Chermack ST, Zimmerman MA, et al. Brief motivational interviewing intervention for peer violence and alcohol use in teens: One-year follow-up. *Pediatrics* 2012; **129**(6): 1083-1090. doi:10.1542/peds.2011-3419

206. Cuomo I, Kotzalidis GD, de Persis S, et al. Head-to-head comparison of 1-year aripiprazole long-acting injectable (LAI) versus paliperidone LAI in comorbid psychosis and substance use disorder: Impact on clinical status, substance craving, and quality of life. *Neuropsychiatr Dis Treat* 2018; **14**: 1645-1656. doi:10.2147/NDT.S171002

207. D’Ascanio L, Vitelli F, Cingolani C, Maranzano M, Brenner MJ, Stadio ADI. Randomized clinical trial “olfactory dysfunction after COVID-19: Olfactory rehabilitation therapy vs. intervention treatment with Palmitoylethanolamide and Luteolin”: Preliminary results. *Eur Rev Med Pharmacol Sci* 2021; **25**(11): 4156-4162. doi:10.26355/eurrev_202106_26059

208. Da Costa D, Abrahamowicz M, Lowensteyn I, et al. A randomized clinical trial of an individualized home-based exercise programme for women with fibromyalgia. *Rheumatology (UK)* 2005; **44**(11): 1422-1427. doi:10.1093/rheumatology/kei032

209. Da Mata C, Allen PF, Cronin M, O'Mahony D, McKenna G, Woods N. Cost-effectiveness of ART restorations in elderly adults: A randomized clinical trial. *Community Dent Oral Epidemiol* 2014; **42**(1): 79-87. doi:10.1111/cdoe.12066

210. Da Mata C, Allen PF, McKenna G, Cronin M, O'Mahony D, Woods N. Two-year survival of ART restorations placed in elderly patients: A randomised controlled clinical trial. *J Dent* 2015; **43**(4): 405-411. doi:10.1016/j.jdent.2015.01.003

211. da Mata C, McKenna G, Anweigi L, et al. An RCT of atraumatic restorative treatment for older adults: 5 year results. *J Dent* 2019; **83**: 95-99. doi:10.1016/j.jdent.2019.03.003

212. Dahlberg LE, Holme I, Høye K, Ringertz B. A randomized, multicentre, double-blind, parallel-group study to assess the adverse event-related discontinuation rate with celecoxib and diclofenac in elderly patients with osteoarthritis. *Scand J Rheumatol* 2009; **38**(2): 133-143. doi:10.1080/03009740802419065

213. Dakof GA, Henderson CE, Rowe CL, et al. A randomized clinical trial of family therapy in juvenile drug court. *J Fam Psychol* 2015; **29**(2): 232-241. doi:10.1037/fam0000053

214. Dal Negro RW, Turco P, Povero M. Nebivolol: an effective option against long-lasting dyspnoea following COVID-19 pneumonia - a pivotal double-blind, cross-over controlled study. *Multidiscip Resp Med* 2022; **17**. doi:10.4081/mrm.2022.886

215. Dalgaard VL, Andersen LPS, Andersen JH, Willert MV, Carstensen O, Glasscock DJ. Work-focused cognitive behavioral intervention for psychological complaints in patients on sick leave due to work-related stress: Results from a randomized controlled trial. *J Negat Results Biomed* 2017; **16**(1). doi:10.1186/s12952-017-0078-z

216. Dalgaard VL, Aschbacher K, Andersen JH, et al. Return to work after work-related stress: A randomized controlled trial of a work-focused cognitive behavioral intervention. *Scand J Work Environ Health* 2017; **43**(5): 436-446. doi:10.5271/sjweh.3655

217. Daly LA, Haden SC, Hagins M, Papouchis N, Ramirez PM. Yoga and emotion regulation in high school students: A randomized controlled trial. *Evid-Based Complement Altern Med* 2015; **2015**: 794928. doi:10.1155/2015/794928

218. Danielsson I, Torstensson T, Brodda-Jansen G, Bohm-Starke N. EMG biofeedback versus topical lidocaine gel: A randomized study for the treatment of women with vulvar vestibulitis. *Acta Obstet Gynecol Scand* 2006; **85**(11): 1360-1367. doi:10.1080/00016340600883401

219. Davis NJ, Tomuta N, Isasi CR, Leung V, Wylie-Rosett J. Diabetes-specific Quality of Life After a Low-carbohydrate and Low-fat Dietary Intervention. *Diabetes Educ* 2012; **38**(2): 250-255. doi:10.1177/0145721711436132

220. Davis NJ, Tomuta N, Schechter C, et al. Comparative study of the effects of a 1-year dietary intervention of a low-carbohydrate diet versus a low-fat diet on weight and glycemic control in type 2 diabetes. *Diabetes Care* 2009; **32**(7): 1147-1152. doi:10.2337/dc08-2108

221. Davison G, Perkins E, Jones AW, et al. Coldzyme® Mouth Spray reduces duration of upper respiratory tract infection symptoms in endurance athletes under free living conditions. *Eur J Sport Sci* 2021; **21**(5): 771-780. doi:10.1080/17461391.2020.1771429

222. De Jonge-Heesen KWJ, Rasing SPA, Vermulst AA, et al. Randomized control trial testing the effectiveness of implemented depression prevention in high-risk adolescents. *BMC Med* 2020; **18**(1). doi:10.1186/s12916-020-01656-0

223. de Vente W, Kamphuis JH, Emmelkamp PMG, Blonk RWB. Individual and Group Cognitive-Behavioral Treatment for Work-Related Stress Complaints and Sickness Absence: A Randomized Controlled Trial. *J Occup Health Psychol* 2008; **13**(3): 214-231. doi:10.1037/1076-8998.13.3.214

224. de Vries SLA, Hoeve M, Asscher JJ, Stams GJJM. The Long-Term Effects of the Youth Crime Prevention Program “New Perspectives” on Delinquency and Recidivism. *Int J Offender Ther Comp Criminol* 2018; **62**(12): 3639-3661. doi:10.1177/0306624X17751161

225. De Weerd BJ, Van Dijk MK, Van Der Linden JN, Roelen CAM, Verbraak MJPM. The effectiveness of a convergence dialogue meeting with the employer in promoting return to work as part of the cognitive-behavioural treatment of common mental disorders: A randomized controlled trial. *Work* 2016; **54**(3): 647-655. doi:10.3233/WOR-162307

226. Deblinger E, Stauffer LB, Steer RA. Comparative efficacies of supportive and cognitive behavioral group therapies for young children who have been sexually abused and their nonoffending mothers. *Child Maltreatmet* 2001; **6**(4): 332-343. doi:10.1177/1077559501006004006

227. DeGarmo DS, Forgatch MS. Early development of delinquency within divorced families: Evaluating a randomized preventive intervention trial. *Dev Sci* 2005; **8**(3): 229-239. doi:10.1111/j.1467-7687.2005.00412.x

228. DeGarmo DS, Patterson GR, Forgatch MS. How do outcomes in a specified parent training intervention maintain or wane over time? *Prev Sci* 2004; **5**(2): 73-89. doi:10.1023/B:PREV.0000023078.30191.e0

229. del Corral T, Fabero-Garrido R, Plaza-Manzano G, Fernández-de-las-Peñas C, Navarro-Santana M, López-de-Uralde-Villanueva I. Home-based respiratory muscle training on quality of life and exercise tolerance in long-term post-COVID-19: Randomized controlled trial. *Ann Phys Rehabil Med* 2023; **66**(1). doi:10.1016/j.rehab.2022.101709

230. Dennis CL, Grigoriadis S, Zupancic J, Kiss A, Ravitz P. Telephone-based nurse-delivered interpersonal psychotherapy for postpartum depression: Nationwide randomised controlled trial. *Br J Psychiatry* 2020; **216**(4): 189-196. doi:10.1192/bjp.2019.275

231. Di Stadio A, D’ascanio L, Vaira LA, et al. Ultramicronized Palmitoylethanolamide and Luteolin Supplement Combined with Olfactory Training to Treat Post-COVID-19 Olfactory Impairment: A Multi-Center Double-Blinded Randomized Placebo-Controlled Clinical Trial. *Curr Neuropharmacol* 2022; **20**(10): 2001-2012. doi:10.2174/1570159X20666220420113513

232. Di Stadio A, Gallina S, Cocuzza S, et al. Treatment of COVID-19 olfactory dysfunction with olfactory training, palmitoylethanolamide with luteolin, or combined therapy: a blinded controlled multicenter randomized trial. *Eur Arch Oto-Rhino-Laryngol* 2023; **280**(11): 4949-4961. doi:10.1007/s00405-023-08085-8

233. Dias KR, de Andrade CB, Wait TT, et al. Efficacy of sealing occlusal caries with a flowable composite in primary molars: A 2-year randomized controlled clinical trial. *J Dent* 2018; **74**: 49-55. doi:10.1016/j.jdent.2018.05.014

234. Dimidjian S, Goodman SH, Sherwood NE, et al. A Pragmatic Randomized Clinical Trial of Behavioral Activation for Depressed Pregnant Women. *J Consult Clin Psychol* 2017; **85**(1): 26-36. doi:10.1037/ccp0000151

235. Dingemans AE, Spinhoven P, van Furth EF. Predictors and mediators of treatment outcome in patients with binge eating disorder. *Behav Res Ther* 2007; **45**(11): 2551-2562. doi:10.1016/j.brat.2007.06.003

236. Diomande I, Gabriel N, Kashiwagi M, et al. Subcutaneous botulinum toxin type A injections for provoked vestibulodynia: a randomized placebo-controlled trial and exploratory subanalysis. *Arch Gynecol Obstet* 2019. doi:10.1007/s00404-019-05043-w

237. DiPerna JC, Lei P, Bellinger J, Cheng W. Efficacy of the social skills improvement system classwide intervention program (SSIS-CIP) primary version. *Sch Psychol Q* 2015; **30**(1): 123-141. doi:10.1037/spq0000079

238. Dishion TJ, Brennan LM, Shaw DS, McEachern AD, Wilson MN, Jo B. Prevention of problem behavior through annual family check-ups in early childhood: Intervention effects from home to early elementary school. *J Abnorm Child Psychol* 2014; **42**(3): 343-354. doi:10.1007/s10802-013-9768-2

239. Dishion TJ, Shaw D, Connell A, Gardner F, Weaver C, Wilson M. The family check-up with high-risk indigent families: Preventing problem behavior by increasing parents' positive behavior support in early childhood. *Child Dev* 2008; **79**(5): 1395-1414. doi:10.1111/j.1467-8624.2008.01195.x

240. Ditto A, Morgante G, La Marca A, De Leo V. Evaluation of treatment of hyperemesis gravidarum using parenteral fluid with or without diazepam. A randomized study. *Gynecol Obstet Invest* 1999; **48**(4): 232-236. doi:10.1159/000010189

241. Dobias ML, Schleider JL, Jans L, Fox KR. An online, single-session intervention for adolescent self-injurious thoughts and behaviors: Results from a randomized trial. *Behav Res Ther* 2021; **147**. doi:10.1016/j.brat.2021.103983

242. Dobscha SK, Corson K, Perrin NA, et al. Collaborative care for chronic pain in primary care: A cluster randomized trial. *J Am Med Assoc* 2009; **301**(12): 1242-1252. doi:10.1001/jama.2009.377

243. Dogra S, Beydoun S, Mazzola J, Hopwood M, Wan Y. Oxcarbazepine in painful diabetic neuropathy: A randomized, placebo-controlled study. *Eur J Pain* 2005; **9**(5): 543. doi:10.1016/j.ejpain.2004.11.006

244. Doig E, Fleming J, Kuipers P, Cornwell P, Khan A. Goal-directed outpatient rehabilitation following TBI: A pilot study of programme effectiveness and comparison of outcomes in home and day hospital settings. *Brain Inj* 2011; **25**(11): 1114-1125. doi:10.3109/02699052.2011.607788

245. Domitrovich CE, Cortes RC, Greenberg MT. Improving young children's social and emotional competence: A randomized trial of the preschool "PATHS" curriculum. *J Prim Prev* 2007; **28**(2): 67-91. doi:10.1007/s10935-007-0081-0

246. Donaldson D, Spirito A, Esposito-Smythers C. Treatment for adolescents following a suicide attempt: Results of a pilot trial. *J AM ACAD CHILD ADOLESC PSYCHIATRY* 2005; **44**(2): 113-120. doi:10.1097/00004583-200502000-00003

247. Donati MA, Primi C, Chiesi F. Prevention of problematic gambling behavior among adolescents: testing the efficacy of an integrative intervention. *J Gambl Stud* 2014; **30**(4): 803-818. doi:10.1007/s10899-013-9398-1

248. Donders GG, Bellen G. Cream with cutaneous fibroblast lysate for the treatment of provoked vestibulodynia: A double-blind randomized placebo-controlled crossover study. *J Lower Genital Tract Dis* 2012; **16**(4): 427-436. doi:10.1097/LGT.0b013e31825a2274

249. Dorrepaal E, Thomaes K, Smit JH, et al. Stabilizing group treatment for complex posttraumatic stress disorder related to child abuse based on psychoeducation and cognitive behavioural therapy: A multisite randomized controlled trial. *Psychother Psychosom* 2012; **81**(4): 217-225. doi:10.1159/000335044

250. Dorsett R, Robins PK. A Multilevel Analysis of the Impacts of Services Provided by the U.K. Employment Retention and Advancement Demonstration. *Eval Rev* 2013; **37**(2): 63-108. doi:10.1177/0193841X13517383

251. Dowling K, Simpkin AJ, Barry MM. A Cluster Randomized-Controlled Trial of the MindOut Social and Emotional Learning Program for Disadvantaged Post-Primary School Students. *J Youth Adolesc* 2019; **48**(7): 1245-1263. doi:10.1007/s10964-019-00987-3

252. Drugli MB, Fossum S, Larsson B, Morch WT. Characteristics of young children with persistent conduct problems 1 year after treatment with the Incredible Years program. *Eur Child Adolesc Psychiatry* 2010; **19**(7): 559-565. doi:10.1007/s00787-009-0083-y

253. Drugli MB, Larsson B. Children aged 4-8 years treated with parent training and child therapy because of conduct problems: Generalisation effects to day-care and school settings. *Eur Child Adolesc Psychiatry* 2006; **15**(7): 392-399. doi:10.1007/s00787-006-0546-3

254. Du Bois M, Donceel P. Guiding low back claimants to work: A randomized controlled trial. *Spine* 2012; **37**(17): 1425-1431. doi:10.1097/BRS.0b013e31824e4ada

255. Duangthip D, Chu CH, Lo ECM. A randomized clinical trial on arresting dentine caries in preschool children by topical fluorides - 18 month results. *J Dent* 2016; **44**: 57-63. doi:10.1016/j.jdent.2015.05.006

256. Duangthip D, Wong MCM, Chu CH, Lo ECM. Caries arrest by topical fluorides in preschool children: 30-month results. *J Dent* 2018; **70**: 74-79. doi:10.1016/j.jdent.2017.12.013

257. Duarté-Vélez Y, Jimenez-Colon G, Jones RN, Spirito A. Socio-Cognitive Behavioral Therapy for Latinx Adolescent with Suicidal Behaviors: A Pilot Randomized Trial. *Child Psychiatry Hum Dev* 2024; **55**(3): 754-767. doi:10.1007/s10578-022-01439-z

258. Dumarkaite A, Truskauskaite-Kuneviciene I, Andersson G, Kazlauskas E. The Effects of Online Mindfulness-Based Intervention on Posttraumatic Stress Disorder and Complex Posttraumatic Stress Disorder Symptoms: A Randomized Controlled Trial With 3-Month Follow-Up. *Front Psychiatry* 2022; **13**: 799259. doi:10.3389/fpsyt.2022.799259

259. Dvořáková K, Kishida M, Li J, et al. Promoting healthy transition to college through mindfulness training with first-year college students: Pilot randomized controlled trial. *J Am Coll Health* 2017; **65**(4): 259-267. doi:10.1080/07448481.2017.1278605

260. Eccles R, Meier C, Jawad M, Weinmüllner R, Grassauer A, Prieschl-Grassauer E. Efficacy and safety of an antiviral Iota-Carrageenan nasal spray: A randomized, double-blind, placebo-controlled exploratory study in volunteers with early symptoms of the common cold. *Respir Res* 2010; **11**. doi:10.1186/1465-9921-11-108

261. Eisner M, Nagin D, Ribeaud D, Malti T. Effects of a Universal Parenting Program for Highly Adherent Parents: A Propensity Score Matching Approach. *Prev Sci* 2012; **13**(3): 252-266. doi:10.1007/s11121-011-0266-x

262. Eklund M. Minor long-Term effects 3-4 years after the ReDO™ intervention for women with stress-related disorders: A focus on sick leave rate, everyday occupations and well-being. *Work* 2017; **58**(4): 527-536. doi:10.3233/WOR-172639

263. Eklund M, Erlandsson LK. Return to Work Outcomes of the Redesigning Daily Occupations (ReDO) Program for Women with Stress-Related Disorders-A Comparative Study. *Women Health* 2011; **51**(7): 676-692. doi:10.1080/03630242.2011.618215

264. El Nakeeb A, El Geidie A, El Hanafy E, et al. Management and Outcome of Borderline Common Bile Duct with Stones: A Prospective Randomized Study. *J Laparoendosc Adv Surg Techn* 2016; **26**(3): 161-167. doi:10.1089/lap.2015.0493

265. El-Geidie AAR. Laparoendoscopic management of concomitant gallbladder stones and common bile duct stones: What is the best technique? *Surg Laparoscopy Endosc Percutaneous Tech* 2011; **21**(4): 282-287. doi:10.1097/SLE.0b013e3182218908

266. Elhennawy K, Finke C, Paris S, Reda S, Jost-Brinkmann PG, Schwendicke F. Selective vs stepwise removal of deep carious lesions in primary molars: 12-Months results of a randomized controlled pilot trial. *J Dent* 2018; **77**: 72-77. doi:10.1016/j.jdent.2018.07.011

267. Emanuelsson P, Gunnarsson U, Dahlstrand U, Strigård K, Stark B. Operative correction of abdominal rectus diastasis (ARD) reduces pain and improves abdominal wall muscle strength: A randomized, prospective trial comparing retromuscular mesh repair to double-row, self-retaining sutures. *Surgery* 2016; **160**(5): 1367-1375. doi:10.1016/j.surg.2016.05.035

268. Emanuelsson P, Gunnarsson U, Strigård K, Stark B. Early complications, pain, and quality of life after reconstructive surgery for abdominal rectus muscle diastasis: A 3-month follow-up. *J Plast Reconstr Aesthetic Surg* 2014; **67**(8): 1082-1088. doi:10.1016/j.bjps.2014.04.015

269. Endler M, Petro G, Gemzell Danielsson K, et al. A telemedicine model for abortion in South Africa: a randomised, controlled, non-inferiority trial. *Lancet* 2022; **400**(10353): 670-679. doi:10.1016/S0140-6736(22)01474-X

270. Engel CC, Litz B, Magruder KM, et al. Delivery of self training and education for stressful situations (DESTRESS-PC): A randomized trial of nurse assisted online self-management for PTSD in primary care. *Gen Hosp Psychiatry* 2015; **37**(4): 323-328. doi:10.1016/j.genhosppsych.2015.04.007

271. Enomoto H, Yasuda H, Nishiyori A, et al. Duloxetine in patients with diabetic peripheral neuropathic pain in Japan: A randomized, double-blind, noninferiority comparative study with pregabalin. *J Pain Res* 2018; **11**: 1857-1868. doi:10.2147/JPR.S170646

272. Ericksen J, Loughlin E, Holt C, et al. A THERAPEUTIC PLAYGROUP FOR DEPRESSED MOTHERS AND THEIR INFANTS: FEASIBILITY STUDY AND PILOT RANDOMIZED TRIAL OF COMMUNITY HUGS. *Infant Ment Health J* 2018; **39**(4): 396-409. doi:10.1002/imhj.21723

273. Espeland M, Pi-Sunyer X, Blackburn G, et al. Reduction in Weight and Cardiovascular Disease Risk Factors in Individuals with Type 2 Diabetes One-year results of the Look AHEAD trial. *Diabetes Care* 2007; **30**(6): 1374-1383. doi:10.2337/dc07-0048

274. Espinoza-Bravo C, Arnal-Gómez A, Martínez-Arnau FM, et al. Effectiveness of Functional or Aerobic Exercise Combined with Breathing Techniques in Telerehabilitation for Patients with Long COVID: A Randomized Controlled Trial. *Phys Ther* 2023; **103**(11). doi:10.1093/ptj/pzad118

275. Esposito K, Maiorino MI, Ciotola M, et al. Effects of a Mediterranean-style diet on the need for antihyperglycemic drug therapy in patients with newly diagnosed type 2 diabetes: A randomized trial. *Ann Intern Med* 2009; **151**(5): 306-314. doi:10.7326/0003-4819-151-5-200909010-00004

276. Esposito K, Maiorino MI, Petrizzo M, Bellastella G, Giugliano D. The effects of a Mediterranean diet on the need for diabetes drugs and remission of newly diagnosed type 2 diabetes: Follow-up of a randomized trial. *Diabetes Care* 2014; **37**(7): 1824-1830. doi:10.2337/dc13-2899

277. Esposito-Smythers C, Spirito A, Kahler CW, Hunt J, Monti P. Treatment of co-occurring substance abuse and suicidality among adolescents: A randomized trial. *J Consult Clin Psychol* 2011; **79**(6): 728-739. doi:10.1037/a0026074

278. Esposito-Smythers C, Wolff JC, Liu RT, et al. Family-focused cognitive behavioral treatment for depressed adolescents in suicidal crisis with co-occurring risk factors: a randomized trial. *J Child Psychol Psychiatry Allied Discip* 2019; **60**(10): 1133-1141. doi:10.1111/jcpp.13095

279. Essau CA, Conradt J, Sasagawa S, Ollendick TH. Prevention of Anxiety Symptoms in Children: Results From a Universal School-Based Trial. *Behav Ther* 2012; **43**(2): 450-464. doi:10.1016/j.beth.2011.08.003

280. Faltin DL, Boulvain M, Floris LA, Irion O. Diagnosis of anal sphincter tears to prevent fecal incontinence: A randomized controlled trial. *Obstet Gynecol* 2005; **106**(1): 6-13. doi:10.1097/01.AOG.0000165273.68486.95

281. Fan Y, Shi Y, Zhang J, et al. The effects of narrative exposure therapy on COVID-19 patients with post-traumatic stress symptoms: A randomized controlled trial. *Journal of Affective Disorders* 2021; **293**: 141-147. doi:10.1016/j.jad.2021.06.019

282. Fancourt D, Perkins R. Effect of singing interventions on symptoms of postnatal depression: Three-arm randomised controlled trial. *Br J Psychiatry* 2018; **212**(2): 119-121. doi:10.1192/bjp.2017.29

283. Farajun Y, Zarfati D, Abramov L, Livoff A, Bornstein J. Enoxaparin treatment for vulvodynia: A randomized controlled trial. *Obstet Gynecol* 2012; **120**(3): 565-572. doi:10.1097/AOG.0b013e3182657de6

284. Fassino S, Leombruni P, Daga GA, et al. Efficacy of citalopram in anorexia nervosa: A pilot study. *Eur Neuropsychopharmacol* 2002; **12**(5): 453-459. doi:10.1016/S0924-977X(02)00058-5

285. Fazekas T, Eickhoff P, Pruckner N, et al. Lessons learned from a double-blind randomised placebo-controlled study with a iota-carrageenan nasal spray as medical device in children with acute symptoms of common cold. *BMC Complement Altern Med* 2012; **12**. doi:10.1186/1472-6882-12-147

286. Feigenbaum JD, Fonagy P, Pilling S, Jones A, Wildgoose A, Bebbington PE. A real-world study of the effectiveness of DBT in the UK National Health Service. *Br J Clin Psychol* 2012; **51**(2): 121-141. doi:10.1111/j.2044-8260.2011.02017.x

287. Fenwick J, Toohill J, Gamble J, et al. Effects of a midwife psycho-education intervention to reduce childbirth fear on women's birth outcomes and postpartum psychological wellbeing. *BMC Pregnancy Childbirth* 2015; **15**(1). doi:10.1186/s12884-015-0721-y

288. Fernández-Barrera MÁ, Lara-Carrillo E, Scougall-Vilchis RJ, et al. Study protocol of the cost-effectiveness comparison of two preventive methods in the incidence of caries: A randomized, controlled clinical trial. *Medicine* 2019; **98**(30): e16634. doi:10.1097/MD.0000000000016634

289. Field T, Diego M, Delgado J, Medina L. Peer support and interpersonal psychotherapy groups experienced decreased prenatal depression, anxiety and cortisol. *Early Hum Dev* 2013; **89**(9): 621-624. doi:10.1016/j.earlhumdev.2013.04.006

290. Figueiredo LP, Paim PVDSL, Cerqueira-Silva T, Barreto CC, Lessa MM. Alpha-lipoic acid does not improve olfactory training results in olfactory loss due to COVID-19: a double-blind randomized trial. *Brazilian J Otorhinolaryngol* 2024; **90**(1). doi:10.1016/j.bjorl.2023.101356

291. Filipčić I, Šimunović Filipčić I, Milovac, et al. Efficacy of repetitive transcranial magnetic stimulation using a figure-8-coil or an H1-Coil in treatment of major depressive disorder; A randomized clinical trial. *J Psychiatr Res* 2019; **114**: 113-119. doi:10.1016/j.jpsychires.2019.04.020

292. Finnes A, Ghaderi A, Dahl J, Nager A, Enebrink P. Randomized controlled trial of acceptance and commitment therapy and a workplace intervention for sickness absence due to mental disorders. *J Occup Health Psychol* 2019; **24**(1): 198-212. doi:10.1037/ocp0000097

293. Finnigan LEM, Cassar MP, Koziel MJ, et al. Efficacy and tolerability of an endogenous metabolic modulator (AXA1125) in fatigue-predominant long COVID: a single-centre, double-blind, randomised controlled phase 2a pilot study. *EClinicalMedicine* 2023; **59**: 101946. doi:10.1016/j.eclinm.2023.101946

294. Fischer-Rasmussen W, Kjær SK, Dahl C, Asping U. Ginger treatment of hyperemesis gravidarum. *Eur J Obstet Gynecol Reprod Biol* 1991; **38**(1): 19-24. doi:10.1016/0028-2243(91)90202-V

295. Fishbein DH, Domitrovich C, Williams J, et al. Short-Term Intervention Effects of the PATHS Curriculum in Young Low-Income Children: Capitalizing on Plasticity. *J Prim Prev* 2016; **37**(6): 493-511. doi:10.1007/s10935-016-0452-5

296. Fletcher SJ, Waterman H, Nelson L, et al. Holistic assessment of women with hyperemesis gravidarum: A randomised controlled trial. *Int J Nurs Stud* 2015; **52**(11): 1669-1677. doi:10.1016/j.ijnurstu.2015.06.007

297. Fleten N, Johnsen R. Reducing sick leave by minimal postal intervention: A randomised, controlled intervention study. *Occupational and Environmental Medicine* 2006; **63**(10): 676-682. doi:10.1136/oem.2005.020438

298. Flook L, Goldberg SB, Pinger L, Davidson RJ. Promoting prosocial behavior and self-regulatory skills in preschool children through a mindfulness-based kindness curriculum. *Dev Psychol* 2015; **51**(1): 44-51. doi:10.1037/a0038256

299. Floyd K, Whelan JP, Meyers AW. Use of warning messages to modify gambling beliefs and behavior in a laboratory investigation. *Psychol Addict Behav* 2006; **20**(1): 69-74. doi:10.1037/0893-164X.20.1.69

300. Fluge Ø, Rekeland IG, Lien K, et al. B-lymphocyte depletion in patients with myalgic encephalomyelitis/chronic fatigue syndrome: A randomized, double-blind, placebo-controlled trial. *Ann Intern Med* 2019; **170**(9): 585-593. doi:10.7326/M18-1451

301. Foa EB, Yusko DA, McLean CP, et al. Concurrent naltrexone and prolonged exposure therapy for patients with comorbid alcohol dependence and PTSD: A randomized clinical trial. *J Am Med Assoc* 2013; **310**(5): 488-495. doi:10.1001/jama.2013.8268

302. Fogelkvist M, Gustafsson SA, Kjellin L, Parling T. Acceptance and commitment therapy to reduce eating disorder symptoms and body image problems in patients with residual eating disorder symptoms: A randomized controlled trial. *Body Image* 2020; **32**: 155-166. doi:10.1016/j.bodyim.2020.01.002

303. Fonagy P, Butler S, Cottrell D, et al. Multisystemic therapy versus management as usual in the treatment of adolescent antisocial behaviour (START): a pragmatic, randomised controlled, superiority trial. *Lancet Psychiatry* 2018; **5**(2): 119-133. doi:10.1016/S2215-0366(18)30001-4

304. Fontaine KR, Conn L, Clauw DJ. Effects of lifestyle physical activity on perceived symptoms and physical function in adults with fibromyalgia: Results of a randomized trial. *Arthritis Res Ther* 2010; **12**(2). doi:10.1186/ar2967

305. Fontaine KR, Conn L, Clauw DJ. Effects of lifestyle physical activity in adults with fibromyalgia: Results at follow-up. *J Clin Rheumatol* 2011; **17**(2): 64-68. doi:10.1097/RHU.0b013e31820e7ea7

306. Forgatch MS, DeGarmo DS. Parenting through change: An effective prevention program for single mothers. *J Consult Clin Psychol* 1999; **67**(5): 711-724. doi:10.1037/0022-006X.67.5.711

307. Forgatch MS, Patterson GR, Degarmo DS, Beldavs ZG. Testing the Oregon delinquency model with 9-year follow-up of the Oregon Divorce Study. *Dev Psychopathol* 2009; **21**(2): 637-660. doi:10.1017/S0954579409000340

308. Forrest GC, Standish E, Baum JD. Support after perinatal death: A study of support and counselling after perinatal bereavement. *Br Med J* 1982; **285**(6353): 1475-1479. doi:10.1136/bmj.285.6353.1475

309. Forsell E, Bendix M, Holländare F, et al. Internet delivered cognitive behavior therapy for antenatal depression: A randomised controlled trial. *Journal of Affective Disorders* 2017; **221**: 56-64. doi:10.1016/j.jad.2017.06.013

310. Foster DC, Kotok MB, Huang LS, et al. Oral desipramine and topical lidocaine for vulvodynia: A randomized controlled trial. *Obstet Gynecol* 2010; **116**(3): 583-593. doi:10.1097/AOG.0b013e3181e9e0ab

311. Frank TJ, Keown LJ, Sanders MR. Enhancing Father Engagement and Interparental Teamwork in an Evidence-Based Parenting Intervention: A Randomized-Controlled Trial of Outcomes and Processes. *Behav Ther* 2014; **46**(6): 749-763. doi:10.1016/j.beth.2015.05.008

312. Franzon R, Guimarães LF, Magalhães CE, Haas AN, Araujo FB. Outcomes of one-step incomplete and complete excavation in primary teeth: A 24-month randomized controlled trial. *Caries Res* 2014; **48**(5): 376-383. doi:10.1159/000357628

313. Freynhagen R, Strojek K, Griesing T, Whalen E, Balkenohl M. Efficacy of pregabalin in neuropathic pain evaluated in a 12-week, randomised, double-blind, multicentre, placebo-controlled trial of flexible- and fixed-dose regimens. *Pain* 2005; **115**(3): 254-263. doi:10.1016/j.pain.2005.02.032

314. Fuhr DC, Weobong B, Lazarus A, et al. Delivering the Thinking Healthy Programme for perinatal depression through peers: an individually randomised controlled trial in India. *Lancet Psychiatry* 2019; **6**(2): 115-127. doi:10.1016/S2215-0366(18)30466-8

315. Gagnon AJ, Waghorn K, Covell C. A Randomized Trial of One-to-One Nurse Support of Women in Labor. *Birth* 1997; **24**(2): 71-77. doi:10.1111/j.1523-536X.1997.tb00344.x

316. Gamboa A, Paranjape SY, Black BK, et al. Inspiratory Resistance Improves Postural Tachycardia: A Randomized Study. *Circ Arrhythmia Electrophysiol* 2015; **8**(3): 651-658. doi:10.1161/CIRCEP.114.002605

317. Gao J, Ding XM, Ke S, et al. Anisodamine accelerates spontaneous passage of single symptomatic bile duct stones ≤ 10 mm. *World J Gastroenterol* 2013; **19**(39): 6618-6624. doi:10.3748/wjg.v19.i39.6618

318. Gao K, Ganocy SJ, Conroy C, Brownrigg B, Serrano MB, Calabrese JR. A placebo controlled study of quetiapine-XR in bipolar depression accompanied by generalized anxiety with and without a recent history of alcohol and cannabis use. *Psychopharmacology* 2017; **234**(15): 2233-2244. doi:10.1007/s00213-017-4642-5

319. Gao Y, Guo X, Han P, et al. Treatment of patients with diabetic peripheral neuropathic pain in China: A double-blind randomised trial of duloxetine vs. placebo. *Int J Clin Pract* 2015; **69**(9): 957-966. doi:10.1111/ijcp.12641

320. Gao Y, Ning G, Jia WP, et al. Duloxetine versus placebo in the treatment of patients with diabetic neuropathic pain in China. *Chin Med J* 2010; **123**(22): 3184-3192. doi:10.3760/cma.j.issn.0366-6999.2010.22.003

321. Garber J, Clarke GN, Weersing VR, et al. Prevention of depression in at-risk adolescents: A randomized controlled trial. *J Am Med Assoc* 2009; **301**(21): 2215-2224. doi:10.1001/jama.2009.788

322. Gardner F, Connell A, Trentacosta CJ, Shaw DS, Dishion TJ, Wilson MN. Moderators of Outcome in a Brief Family-Centered Intervention for Preventing Early Problem Behavior. *J Consult Clin Psychol* 2009; **77**(3): 543-553. doi:10.1037/a0015622

323. Gardner F, Shaw DS, Dishion TJ, Burton J, Supplee L. Randomized Prevention Trial for Early Conduct Problems: Effects on Proactive Parenting and Links to Toddler Disruptive Behavior. *J Fam Psychol* 2007; **21**(3): 398-406. doi:10.1037/0893-3200.21.3.398

324. Gawlytta R, Kesselmeier M, Scherag A, et al. Internet-based cognitive-behavioural writing therapy for reducing post-traumatic stress after severe sepsis in patients and their spouses (REPAIR): Results of a randomised-controlled trial. *BMJ Open* 2022; **12**(3). doi:10.1136/bmjopen-2021-050305

325. Geenen S, Powers LE, Phillips LA, et al. Better Futures: a Randomized Field Test of a Model for Supporting Young People in Foster Care with Mental Health Challenges to Participate in Higher Education. *J Behav Health Serv Res* 2015; **42**(2): 150-171. doi:10.1007/s11414-014-9451-6

326. Geist R, Heinmaa M, Stephens D, Davis R, Katzman DK. Comparison of family therapy and family group psychoeducation in adolescents with anorexia nervosa. *Can J Psychiatry* 2000; **45**(2): 173-178. doi:10.1177/070674370004500208

327. Gelisen O, Caliskan E, Dilbaz S, Ozdas E, Dilbaz B, Haberal A. Induction of labor with three different techniques at 41 weeks of gestation or spontaneous follow-up until 42 weeks in women with definitely unfavorable cervical scores. *Eur J Obstet Gynecol Reprod Biol* 2005; **120**(2): 164-169. doi:10.1016/j.ejogrb.2004.08.013

328. Ghaderi A, Kadesjö C, Björnsdotter A, Enebrink P. Randomized effectiveness Trial of the Family Check-Up versus Internet-delivered Parent Training (iComet) for Families of Children with Conduct Problems. *Sci Rep* 2018; **8**(1). doi:10.1038/s41598-018-29550-z

329. Ghaeminia H, Gerlach NL, Hoppenreijs TJM, et al. Clinical relevance of cone beam computed tomography in mandibular third molar removal: A multicentre, randomised, controlled trial. *J Cranio-Maxillofac Surg* 2015; **43**(10): 2158-2167. doi:10.1016/j.jcms.2015.10.009

330. Giannakopoulos G, Solantaus T, Tzavara C, Kolaitis G. Mental health promotion and prevention interventions in families with parental depression: A randomized controlled trial. *Journal of Affective Disorders* 2021; **278**: 114-121. doi:10.1016/j.jad.2020.09.070

331. Giesbrecht EM, Miller WC. Effect of an mHealth Wheelchair Skills Training Program for Older Adults: A Feasibility Randomized Controlled Trial. *Arch Phys Med Rehabil* 2019; **100**(11): 2159-2166. doi:10.1016/j.apmr.2019.06.010

332. Gillham JE, Hamilton J, Freres DR, Patton K, Gallop R. Preventing depression among early adolescents in the primary care setting: A randomized controlled study of the Penn Resiliency Program. *J Abnorm Child Psychol* 2006; **34**(2): 203-219. doi:10.1007/s10802-005-9014-7

333. Gillham JE, Reivich KJ, Brunwasser SM, et al. Evaluation of a Group Cognitive-Behavioral Depression Prevention Program for Young Adolescents: A Randomized Effectiveness Trial. *J Clin Child Adolesc Psychol* 2012; **41**(5): 621-639. doi:10.1080/15374416.2012.706517

334. Gillham JE, Reivich KJ, Freres DR, et al. School-based prevention of depressive symptoms: A randomized controlled study of the effectiveness and specificity of the Penn Resiliency Program. *J Consult Clin Psychol* 2007; **75**(1): 9-19. doi:10.1037/0022-006X.75.1.9

335. Gilron I, Bailey JM, Tu D, Holden RR, Jackson AC, Houlden RL. Nortriptyline and gabapentin, alone and in combination for neuropathic pain: a double-blind, randomised controlled crossover trial. *Lancet* 2009; **374**(9697): 1252-1261. doi:10.1016/S0140-6736(09)61081-3

336. Gimbel JS, Richards P, Portenoy RK. Controlled-release oxycodone for pain in diabetic neuropathy: A randomized controlled trial. *Neurology* 2003; **60**(6): 927-934. doi:10.1212/01.WNL.0000057720.36503.2C

337. Ginley MK, Whelan JP, Keating HA, Meyers AW. Gambling warning messages: The impact of winning and losing on message reception across a gambling session. *Psychol Addict Behav* 2016; **30**: 931-938. doi:10.1037/adb0000212

338. Ginsburg GS. The Child Anxiety Prevention Study: Intervention Model and Primary Outcomes. *J Consult Clin Psychol* 2009; **77**(3): 580-587. doi:10.1037/a0014486

339. Ginsburg GS, Drake KL, Tein JY, Teetsel R, Riddle MA. Preventing onset of anxiety disorders in offspring of anxious parents: A randomized controlled trial of a family-based intervention. *AM J PSYCHIATRY* 2015; **172**(12): 1207-1214. doi:10.1176/appi.ajp.2015.14091178

340. Ginsburg GS, Tein JY, Riddle MA. Preventing the Onset of Anxiety Disorders in Offspring of Anxious Parents: A Six-Year Follow-up. *Child Psychiatry Hum Dev* 2021; **52**(4): 751-760. doi:10.1007/s10578-020-01080-8

341. Gismervik SØ, Aasdahl L, Vasseljen O, et al. Inpatient multimodal occupational rehabilitation reduces sickness absence among individuals with musculoskeletal and common mental health disorders: A randomized clinical trial. *Scand J Work Environ Health* 2020; **46**(4): 364-372. doi:10.5271/sjweh.3882

342. Glasscock DJ, Carstensen O, Dalgaard VL. Recovery from work-related stress: a randomized controlled trial of a stress management intervention in a clinical sample. *Int Arch Occup Environ Health* 2018; **91**(6): 675-687. doi:10.1007/s00420-018-1314-7

343. Gluppe SL, Hilde G, Tennfjord MK, Engh ME, Bø K. Effect of a postpartum training program on the prevalence of diastasis recti abdominis in postpartum primiparous women: A randomized controlled trial. *Phys Ther* 2018; **98**(4): 260-268. doi:10.1093/ptj/pzy008

344. Goldbach JT, Rhoades H, Mamey MR, Senese J, Karys P, Marsiglia FF. Reducing behavioral health symptoms by addressing minority stressors in LGBTQ adolescents: a randomized controlled trial of Proud & Empowered. *BMC Public Health* 2021; **21**(1). doi:10.1186/s12889-021-12357-5

345. Goldfinger C, Pukall CF, Thibault-Gagnon S, McLean L, Chamberlain S. Effectiveness of Cognitive-Behavioral Therapy and Physical Therapy for Provoked Vestibulodynia: A Randomized Pilot Study. *J Sex Med* 2016; **13**(1): 88-94. doi:10.1016/j.jsxm.2015.12.003

346. Goldstein DJ, Lu Y, Detke MJ, Lee TC, Iyengar S. Duloxetine vs. placebo in patients with painful diabetic neuropathy. *Pain* 2005; **116**(1-2): 109-118. doi:10.1016/j.pain.2005.03.029

347. Goldstein DJ, Wilson MG, Thompson VL, Potvin JH, Rampey Jr AH. Long-term fluoxetine treatment of bulimia nervosa. *Br J Psychiatry* 1995; **166**(MAY): 660-666. doi:10.1192/bjp.166.5.660

348. Goldston DB, Curry JF, Wells KC, et al. Feasibility of an Integrated Treatment Approach for Youth with Depression, Suicide Attempts, and Substance Use Problems. *Evid Based Pract Child Adolesc Mental Heal* 2021; **6**(2): 155-172. doi:10.1080/23794925.2021.1888664

349. Gonzalez ACC, Zuluaga DJM. Clinical outcome of root caries restorations using ART and rotary techniques in institutionalized elders. *Braz Oral Res* 2016; **30**(1): 1-8. doi:10.1590/1807-3107BOR-2016.VOL30.0063

350. González-Robles A, Díaz-García A, García-Palacios A, Roca P, Ramos-Quiroga JA, Botella C. Effectiveness of a transdiagnostic guided internet-delivered protocol for emotional disorders versus treatment as usual in specialized care: Randomized controlled trial. *J Med Internet Res* 2020; **22**(7). doi:10.2196/18220

351. Goodman JH, Prager J, Goldstein R, Freeman M. Perinatal Dyadic Psychotherapy for postpartum depression: a randomized controlled pilot trial. *Arch Women's Ment Health* 2015; **18**(3): 493-506. doi:10.1007/s00737-014-0483-y

352. Gotaas ME, Stiles TC, Bjørngaard JH, Borchgrevink PC, Fors EA. Cognitive Behavioral Therapy Improves Physical Function and Fatigue in Mild and Moderate Chronic Fatigue Syndrome: A Consecutive Randomized Controlled Trial of Standard and Short Interventions. *Front Psychiatry* 2021; **12**: 580924. doi:10.3389/fpsyt.2021.580924

353. Gottfredson DC, Kearley B, Thornberry TP, Slothower M, Devlin D, Fader JJ. Scaling-Up Evidence-Based Programs Using a Public Funding Stream: a Randomized Trial of Functional Family Therapy for Court-Involved Youth. *Prev Sci* 2018; **19**(7): 939-953. doi:10.1007/s11121-018-0936-z

354. Gowers SG, Clark AF, Roberts C, et al. A randomised controlled multicentre trial of treatments for adolescent anorexia nervosa including assessment of cost-effectiveness and patient acceptability - The TOuCAN trial. *Health Technol Assess* 2010; **14**(15): 1-98. doi:10.3310/hta14150

355. Graziano PA, Ros-Demarize R, Hare MM. Condensing parent training: A randomized trial comparing the efficacy of a briefer, more intensive version of parent-child interaction therapy (I-PCIT). *J Consult Clin Psychol* 2020; **88**(7): 669-679. doi:10.1037/ccp0000504

356. Green AI, Brunette MF, Dawson R, et al. Long-acting injectable vs oral risperidone for schizophrenia and co-occurring alcohol use disorder: A randomized trial. *J CLIN PSYCHIATRY* 2015; **76**(10): 1359-1365. doi:10.4088/JCP.13m08838

357. Green AL, Ferrante S, Boaz TL, Kutash K, Wheeldon-Reece B. Evaluation of the SPARK Child Mentoring Program: A Social and Emotional Learning Curriculum for Elementary School Students. *J Prim Prev* 2021; **42**(5): 531-547. doi:10.1007/s10935-021-00642-3

358. Green EA, Black BK, Biaggioni I, et al. Melatonin reduces tachycardia in postural tachycardia syndrome: A randomized, crossover trial. *Cardiovasc Ther* 2014; **32**(3): 105-112. doi:10.1111/1755-5922.12067

359. Green EA, Raj V, Shibao CA, et al. Effects of norepinephrine reuptake inhibition on postural tachycardia syndrome. *J Am Heart Assoc* 2013; **2**(5). doi:10.1161/JAHA.113.000395

360. Green JM, Wood AJ, Kerfoot MJ, et al. Group therapy for adolescents with repeated self harm: randomised controlled trial with economic evaluation. *BMJ* 2011; **342**. doi:10.1136/bmj.d682

361. Griffiths H, Duffy F, Duffy L, et al. Efficacy of Mentalization-based group therapy for adolescents: The results of a pilot randomised controlled trial. *BMC Psychiatry* 2019; **19**(1). doi:10.1186/s12888-019-2158-8

362. Grilo CM, Masheb RM. A randomized controlled comparison of guided self-help cognitive behavioral therapy and behavioral weight loss for binge eating disorder. *Behav Res Ther* 2005; **43**(11): 1509-1525. doi:10.1016/j.brat.2004.11.010

363. Grilo CM, Masheb RM, White MA, et al. Treatment of binge eating disorder in racially and ethnically diverse obese patients in primary care: Randomized placebo-controlled clinical trial of self-help and medication. *Behav Res Ther* 2014; **58**: 1-9. doi:10.1016/j.brat.2014.04.002

364. Grilo CM, Masheb RM, Wilson GT. Efficacy of cognitive behavioral therapy and fluoxetine for the treatment of binge eating disorder: A randomized double-blind placebo-controlled comparison. *Biol Psychiatry* 2005; **57**(3): 301-309. doi:10.1016/j.biopsych.2004.11.002

365. Grooten IJ, Koot MH, Van Der Post JAM, et al. Early enteral tube feeding in optimizing treatment of hyperemesis gravidarum: The Maternal and Offspring outcomes after Treatment of HyperEmesis by Refeeding (MOTHER) randomized controlled trial. *Am J Clin Nutr* 2017; **106**(3): 812-820. doi:10.3945/ajcn.117.158931

366. Gross D, Fogg L, Garvey C, Julion W, Webster-Stratton C, Grady J. Parent training of toddlers in day care in low-income urban communities. *J Consult Clin Psychol* 2003; **71**(2): 261-278. doi:10.1037/0022-006X.71.2.261

367. Gross D, Garvey C, Julion W, Fogg L, Tucker S, Mokros H. Efficacy of the Chicago parent program with low-income African American and latino parents of young children. *Prev Sci* 2009; **10**(1): 54-65. doi:10.1007/s11121-008-0116-7

368. Gross DP, Asante AK, Miciak M, et al. Are performance-based functional assessments superior to semistructured interviews for enhancing return-to-work outcomes? *Arch Phys Med Rehabil* 2014; **95**: 807-815.e801. doi:10.1016/j.apmr.2014.01.017

369. Gross DP, Asante AK, Miciak M, et al. A Cluster Randomized Clinical Trial Comparing Functional Capacity Evaluation and Functional Interviewing as Components of Occupational Rehabilitation Programs. *J Occup Rehabil* 2014; **24**(4): 617-630. doi:10.1007/s10926-013-9491-4

370. Grosskopf J, Mazzola J, Wan Y, Hopwood M. A randomized, placebo-controlled study of oxcarbazepine in painful diabetic neuropathy. *Acta Neurol Scand* 2006; **114**(3): 177-180. doi:10.1111/j.1600-0404.2005.00559.x

371. Grote NK, Katon WJ, Russo JE, et al. COLLABORATIVE CARE for PERINATAL DEPRESSION in SOCIOECONOMICALLY DISADVANTAGED WOMEN: A RANDOMIZED TRIAL. *Depression Anxiety* 2015; **32**(11): 821-834. doi:10.1002/da.22405

372. Grote NK, Katon WJ, Russo JE, et al. A randomized trial of collaborative care for perinatal depression in socioeconomically disadvantaged women: The impact of comorbid posttraumatic stress disorder. *J CLIN PSYCHIATRY* 2016; **77**(11): 1527-1537. doi:10.4088/JCP.15m10477

373. Grote NK, Swartz HA, Geibel SL, Zuckoff A, Houck PR, Frank E. A randomized controlled trial of culturally relevant, brief interpersonal psychotherapy for perinatal depression. *Psychiatr Serv* 2009; **60**(3): 313-321. doi:10.1176/ps.2009.60.3.313

374. Grover M, Naumann U, Mohammad-Dar L, et al. A randomized controlled trial of an Internet-based cognitive-behavioural skills package for carers of people with anorexia nervosa. *Psychol Med* 2011; **41**(12): 2581-2591. doi:10.1017/S0033291711000766

375. Gruenwald I, Gutzeit O, Petruseva A, Gartman I, Lowenstein L. Low-Intensity Shockwave for Treatment of Vestibulodynia: A Randomized Controlled Therapy Trial. *J Sex Med* 2021; **18**(2): 347-352. doi:10.1016/j.jsxm.2020.11.006

376. Gual A, Balcells M, Torres M, Madrigal M, Diez T, Serrano L. Sertraline for the prevention of relapse in detoxicated alcohol dependent patients with a comorbid depressive disorder: A randomized controlled trial. *Alcohol Alcohol* 2003; **38**(6): 619-625. doi:10.1093/alcalc/agg124

377. Guan Y, Ding X, Cheng Y, et al. Efficacy of Pregabalin for Peripheral Neuropathic Pain: Results of an 8-Week, Flexible-Dose, Double-Blind, Placebo-Controlled Study Conducted in China. *Clin Ther* 2011; **33**(2): 159-166. doi:10.1016/j.clinthera.2011.02.007

378. Guardino CM, Dunkel Schetter C, Bower JE, Lu MC, Smalley SL. Randomised controlled pilot trial of mindfulness training for stress reduction during pregnancy. *Psychol Health* 2014; **29**(3): 334-349. doi:10.1080/08870446.2013.852670

379. Guerdjikova AI, McElroy SL, Kotwal R, et al. High-dose escitalopram in the treatment of binge-eating disorder with obesity: A placebo-controlled monotherapy trial. *Hum Psychopharmacol* 2008; **23**(1): 1-11. doi:10.1002/hup.899

380. Guillet AD, Cirino NH, Hart KD, Leclair CM. Mindfulness-Based Group Cognitive Behavior Therapy for Provoked Localized Vulvodynia: A Randomized Controlled Trial. *J Lower Genital Tract Dis* 2019; **23**(2): 170-175. doi:10.1097/LGT.0000000000000456

381. Guldbrand H, Dizdar B, Bunjaku B, et al. In type 2 diabetes, randomisation to advice to follow a low-carbohydrate diet transiently improves glycaemic control compared with advice to follow a low-fat diet producing a similar weight loss. *Diabetologia* 2012; **55**(8): 2118-2127. doi:10.1007/s00125-012-2567-4

382. Guldbrand H, Lindström T, Dizdar B, et al. Randomization to a low-carbohydrate diet advice improves health related quality of life compared with a low-fat diet at similar weight-loss in Type 2 diabetes mellitus. *Diabetes Res Clin Pract* 2014; **106**(2): 221-227. doi:10.1016/j.diabres.2014.08.032

383. Guttuso T, Jr., Messing S, Tu X, et al. Effect of gabapentin on hyperemesis gravidarum: a double-blind, randomized controlled trial. *American J Obstet Gynecol MFM* 2021; **3**(1). doi:10.1016/j.ajogmf.2020.100273

384. Haak T, Scott B. The effect of Qigong on Fibromyalgia (FMS): A controlled randomized study. *Disabil Rehabil* 2008; **30**(8): 625-633. doi:10.1080/09638280701400540

385. Habek D, Barbir A, Habek JČ, Jančuljak D, Bobić-Vuković M. Success of acupuncture and acupressure of the Pc 6 acupoint in the treatment of hyperemesis gravidarum. *Forsch Komplementarmed Klass Naturheilkd* 2004; **11**(1): 20-23. doi:10.1159/000077192

386. Hagen EM, Eriksen HR, Ursin H. Does early intervention with a light mobilization program reduce long-term sick leave for low back pain? *Spine* 2000; **25**(15): 1973-1976. doi:10.1097/00007632-200008010-00017

387. Hagen EM, Grasdal A, Eriksen HR. Does early intervention with a light mobilization program reduce long-term sick leave for low back pain: A 3-year follow-up study. *Spine* 2003; **28**(20): 2309-2315. doi:10.1097/01.BRS.0000085817.33211.3F

388. Hagen EM, Ødelien KH, Lie SA, Eriksen HR. Adding a physical exercise programme to brief intervention for low back pain patients did not increase return to work. *Scand J Public Health* 2010; **38**(7): 731-738. doi:10.1177/1403494810382472

389. Hagen KA, Ogden T, Bjornebekk G. Treatment outcomes and mediators of parent management training: A one-year follow-up of children with conduct problems. *J Clin Child Adolesc Psychol* 2011; **40**(2): 165-178. doi:10.1080/15374416.2011.546050

390. Hajek P, Phillips-Waller A, Przulj D, et al. A randomized trial of E-cigarettes versus nicotine-replacement therapy. *New Engl J Med* 2019; **380**(7): 629-637. doi:10.1056/NEJMoa1808779

391. Halley C, Honeywill C, Kang J, et al. Preventing upper respiratory tract infections with prophylactic nasal carrageenan: a feasibility study. *Future Microbiol* 2023; **18**(18): 1319-1328. doi:10.2217/fmb-2021-0122

392. Hallgren M, Helgadóttir B, Herring MP, et al. Exercise and internet-based cognitive-behavioural therapy for depression: Multicentre randomized controlled trial with 12-month follow-up. *Br J Psychiatry* 2016; **209**(5): 414-420. doi:10.1192/bjp.bp.115.177576

393. Hallgren M, Kraepelien M, Öjehagen A, et al. Physical exercise and internet-based cognitive-behavioural therapy in the treatment of depression: Randomised controlled trial. *Br J Psychiatry* 2015; **207**(3): 227-234. doi:10.1192/bjp.bp.114.160101

394. Hallström H, Lindgren S, Twetman S. Effect of a chlorhexidine-containing brush-on gel on peri-implant mucositis. *Int J Dent Hyg* 2017; **15**(2): 149-153. doi:10.1111/idh.12184

395. Hammond A, Freeman K. Community patient education and exercise for people with fibromyalgia: A parallel group randomized controlled trial. *Clin Rehabil* 2006; **20**(10): 835-846. doi:10.1177/0269215506072173

396. Han DH, Kim SM, Choi JE, Min KJ, Renshaw PF. Adjunctive aripiprazole therapy with escitalopram in patients with co-morbid major depressive disorder and alcohol dependence: Clinical and neuroimaging evidence. *J Psychopharmacol* 2013; **27**(3): 282-291. doi:10.1177/0269881112472563

397. Hanks RA, Rapport LJ, Wertheimer J, Koviak C. Randomized controlled trial of peer mentoring for individuals with traumatic brain injury and their significant others. *Arch Phys Med Rehabil* 2012; **93**(8): 1297-1304. doi:10.1016/j.apmr.2012.04.027

398. Hanna M, O'Brien C, Wilson MC. Prolonged-release oxycodone enhances the effects of existing gabapentin therapy in painful diabetic neuropathy patients. *Eur J Pain* 2008; **12**(6): 804-813. doi:10.1016/j.ejpain.2007.12.010

399. Hantsoo L, Criniti S, Khan A, et al. A mobile application for monitoring and management of depressed mood in a vulnerable pregnant population. *Psychiatr Serv* 2018; **69**(1): 104-107. doi:10.1176/appi.ps.201600582

400. Hara KW, Bjørngaard JH, Brage S, et al. Randomized Controlled Trial of Adding Telephone Follow-Up to an Occupational Rehabilitation Program to Increase Work Participation. *J Occup Rehabil* 2018; **28**(2): 265-278. doi:10.1007/s10926-017-9711-4

401. Haraldson P, Mühlrad H, Heddini U, Nilsson K, Bohm-Starke N. Botulinum Toxin A as a Treatment for Provoked Vestibulodynia: A Randomized Controlled Trial. *Obstet Gynecol* 2020; **136**(3): 524-532. doi:10.1097/AOG.0000000000004008

402. Harati Y, Gooch C, Swenson M, et al. Double-blind randomized trial of tramadol for the treatment of the pain of diabetic neuropathy. *Neurology* 1998; **50**(6): 1842-1846. doi:10.1212/WNL.50.6.1842

403. Harrington R, Kerfoot M, Dyer E, et al. Randomized trial of a home-based family intervention for children who have deliberately poisoned themselves. *J AM ACAD CHILD ADOLESC PSYCHIATRY* 1998; **37**(5): 512-518. doi:10.1016/S0890-8567(14)60001-0

404. Harrington R, Pickles A, Aglan A, Harrington V, Burroughs H, Kerfoot M. Early adult outcomes of adolescents who deliberately poisoned themselves. *J AM ACAD CHILD ADOLESC PSYCHIATRY* 2006; **45**(3): 337-345. doi:10.1097/01.chi.0000194564.78536.f2

405. Harris A, Moe TF, Eriksen HR, et al. Brief intervention, physical exercise and cognitive behavioural group therapy for patients with chronic low back pain (The CINS trial). *Eur J Pain* 2017; **21**(8): 1397-1407. doi:10.1002/ejp.1041

406. Hazell PL, Martin G, McGill K, Wood TK, Trainor G, Harrington R. Group therapy for repeated deliberate self-harm in adolescents: Failure of replication of a randomized trial. *J AM ACAD CHILD ADOLESC PSYCHIATRY* 2009; **48**(6): 662-670. doi:10.1097/CHI.0b013e3181a0acec

407. Heazell A, Thorneycroft J, Walton V, Etherington I. Acupressure for the in-patient treatment of nausea and vomiting in early pregnancy: A randomized control trial. *AM J OBSTET GYNECOL* 2006; **194**(3): 815-820. doi:10.1016/j.ajog.2005.08.042

408. Hedman E, Andersson G, Ljótsson B, et al. Internet-based cognitive behavior therapy vs. cognitive behavioral group therapy for social anxiety disorder: A randomized controlled non-inferiority trial. *PLoS ONE* 2011; **6**(3). doi:10.1371/journal.pone.0018001

409. Hedman E, El Alaoui S, Lindefors N, et al. Clinical effectiveness and cost-effectiveness of Internet- vs. group-based cognitive behavior therapy for social anxiety disorder: 4-Year follow-up of a randomized trial. *Behav Res Ther* 2014; **59**: 20-29. doi:10.1016/j.brat.2014.05.010

410. Hees HL, De Vries G, Koeter MWJ, Schene AH. Adjuvant occupational therapy improves long-term depression recovery and return-to-work in good health in sick-listed employees with major depression: Results of a randomised controlled trial. *Occupational and Environmental Medicine* 2013; **70**(4): 252-260. doi:10.1136/oemed-2012-100789

411. Heinrichs N, Kliem S, Hahlweg K. Four-Year Follow-Up of a Randomized Controlled Trial of Triple P Group for Parent and Child Outcomes. *Prev Sci* 2014; **15**(2): 233-245. doi:10.1007/s11121-012-0358-2

412. Heinrichs N, Kliem S, Hahlweg K. Addendum to “Four-Year Follow-Up of a Randomized Controlled Trial of Triple P Group for Parent and Child Outcomes”. *Prev Sci* 2017; **18**(4): 491-503. doi:10.1007/s11121-017-0782-4

413. Heitz-Mayfield LJA, Salvi GE, Botticelli D, Mombelli A, Faddy M, Lang NP. Anti-infective treatment of peri-implant mucositis: A randomised controlled clinical trial. *Clin Oral Implants Res* 2011; **22**(3): 237-241. doi:10.1111/j.1600-0501.2010.02078.x

414. Hellström L, Bech P, Hjorthøj C, Nordentoft M, Lindschou J, Eplov LF. Effect on return to work or education of individual placement and support modified for people with mood and anxiety disorders: Results of a randomised clinical trial. *Occupational and Environmental Medicine* 2017; **74**(10): 717-725. doi:10.1136/oemed-2016-104248

415. Hendriks V, van der Schee E, Blanken P. Treatment of adolescents with a cannabis use disorder: Main findings of a randomized controlled trial comparing multidimensional family therapy and cognitive behavioral therapy in The Netherlands. *Drug Alcohol Depend* 2011; **119**(1-2): 64-71. doi:10.1016/j.drugalcdep.2011.05.021

416. Henggeler SW, Clingempeel WG, Brondino MJ, Pickrel SG. Four-Year Follow-up of Multisystemic Therapy with Substance-Abusing and Substance-Dependent Juvenile Offenders. *J AM ACAD CHILD ADOLESC PSYCHIATRY* 2002; **41**(7): 868-874. doi:10.1097/00004583-200207000-00021

417. Henry M, Reader AI, Beck M. Effect of penicillin on postoperative endodontic pain and swelling in symptomatic necrotic teeth. *J Endod* 2001; **27**(2): 117-123. doi:10.1097/00004770-200102000-00016

418. Heo J, Jung MK, Cho CM. Should prophylactic cholecystectomy be performed in patients with concomitant gallstones after endoscopic sphincterotomy for bile duct stones? *Surgical Endoscopy* 2015; **29**(6): 1574-1579. doi:10.1007/s00464-014-3844-8

419. Hernandez-Avila CA, Modesto-Lowe V, Feinn R, Kranzler HR. Nefazodone Treatment of Comorbid Alcohol Dependence and Major Depression. *Alcohol Clin Exp Res* 2004; **28**(3): 433-440. doi:10.1097/01.ALC.0000118313.63897.EE

420. Herpertz-Dahlmann B, Schwarte R, Krei M, et al. Day-patient treatment after short inpatient care versus continued inpatient treatment in adolescents with anorexia nervosa (ANDI): A multicentre, randomised, open-label, non-inferiority trial. *Lancet* 2014; **383**(9924): 1222-1229. doi:10.1016/S0140-6736(13)62411-3

421. Hesse D, Bonifácio CC, Mendes FM, Braga MM, Imparato JCP, Raggio DP. Sealing versus partial caries removal in primary molars: A randomized clinical trial. *BMC Oral Health* 2014; **14**(1). doi:10.1186/1472-6831-14-58

422. Heutink M, Post MWM, Bongers-Janssen HMH, et al. The CONECSI trial: Results of a randomized controlled trial of a multidisciplinary cognitive behavioral program for coping with chronic neuropathic pain after spinal cord injury. *Pain* 2012; **153**(1): 120-128. doi:10.1016/j.pain.2011.09.029

423. Heymans MW, De Vet HCW, Bongers PM, Knol DL, Koes BW, Van Mechelen W. The effectiveness of high-intensity versus low-intensity back schools in an occupational setting: A pragmatic randomized controlled trial. *Spine* 2006; **31**(10): 1075-1082. doi:10.1097/01.brs.0000216443.46783.4d

424. Hien DA, Rudnick Levin F, Ruglass LM, et al. Combining seeking safety with sertraline for PTSD and alcohol use disorders: A randomized controlled trial. *J Consult Clin Psychol* 2015; **83**(2): 359-369. doi:10.1037/a0038719

425. Hill DM, Craighead LW, Safer DL. Appetite-focused dialectical behavior therapy for the treatment of binge eating with purging: A preliminary trial. *Int J Eating Disord* 2011; **44**(3): 249-261. doi:10.1002/eat.20812

426. Hlobil H, Staal JB, Twisk J, et al. The effects of a graded activity intervention for low back pain in occupational health on sick leave, functional status and pain: 12-Month results of a randomized controlled trial. *J Occup Rehabil* 2005; **15**: 569-580. doi:10.1007/s10926-005-8035-y

427. Hodnett ED, Lowe NK, Hannah ME, et al. Effectiveness of nurses as providers of birth labor support in North American hospitals: A randomized controlled trial. *J Am Med Assoc* 2002; **288**(11): 1373-1381. doi:10.1001/jama.288.11.1373

428. Hodsoll J, Rhind C, Micali N, et al. A Pilot, Multicentre Pragmatic Randomised Trial to Explore the Impact of Carer Skills Training on Carer and Patient Behaviours: Testing the Cognitive Interpersonal Model in Adolescent Anorexia Nervosa. *Eur Eating Disord Rev* 2017; **25**(6): 551-561. doi:10.1002/erv.2540

429. Hoff A, Fisker J, Poulsen RM, et al. Integrating vocational rehabilitation and mental healthcare to improve the return-to-work process for people on sick leave with stress-related disorders: results from a randomized trial. *Scand J Work Environ Health* 2022; **48**(5): 361-371. doi:10.5271/sjweh.4021

430. Hoff A, Poulsen RM, Fisker J, et al. Integrating vocational rehabilitation and mental healthcare to improve the return-to-work process for people on sick leave with depression or anxiety: results from a three-arm, parallel randomised trial. *Occupational and environmental medicine* 2022; **79**(2): 134-142. doi:10.1136/oemed-2021-107894

431. Hollander E, Pallanti S, Allen A, Sood E, Rossi NB. Does sustained-release lithium reduce impulsive gambling and affective instability versus placebo in pathological gamblers with bipolar spectrum disorders? *AM J PSYCHIATRY* 2005; **162**(1): 137-145. doi:10.1176/appi.ajp.162.1.137

432. Holliday R, Preshaw PM, Ryan V, et al. A feasibility study with embedded pilot randomised controlled trial and process evaluation of electronic cigarettes for smoking cessation in patients with periodontitis. *Pilot Feasibility Stud* 2019; **5**(1): 74. doi:10.1186/s40814-019-0451-4

433. Honey KL, Bennett P, Morgan M. A brief psycho-educational group intervention for postnatal depression. *Br J Clin Psychol* 2002; **41**(4): 405-409. doi:10.1348/014466502760387515

434. Hong DF, Xin Y, Chen DW. Comparison of laparoscopic cholecystectomy combined with intraoperative endoscopic sphincterotomy and laparoscopic exploration of the common bile duct for cholecystocholedocholithiasis. *Surg Endosc Interv Tech* 2006; **20**(3): 424-427. doi:10.1007/s00464-004-8248-8

435. Hosseinpoor M, Kabiri M, Rajati Haghi M, et al. Intranasal Corticosteroid Treatment on Recovery of Long-Term Olfactory Dysfunction Due to COVID-19. *Laryngoscope* 2022; **132**(11): 2209-2216. doi:10.1002/lary.30353

436. Hou Y, Hu P, Zhang Y, et al. Cognitive behavioral therapy in combination with systemic family therapy improves mild to moderate postpartum depression. *Rev Bras Psiquiatr* 2014; **36**(1): 47-52. doi:10.1590/1516-4446-2013-1170

437. Hsieh MY, Ponsford J, Wong D, Schnberger M, Taffe J, McKay A. Motivational interviewing and cognitive behaviour therapy for anxiety following traumatic brain injury: A pilot randomised controlled trial. *Neuropsychol Rehabil* 2012; **22**(4): 585-608. doi:10.1080/09602011.2012.678860

438. Hsu MC, Schubiner H, Lumley MA, Stracks JS, Clauw DJ, Williams DA. Sustained pain reduction through affective self-awareness in fibromyalgia: A randomized controlled trial. *J Gen Intern Med* 2010; **25**(10): 1064-1070. doi:10.1007/s11606-010-1418-6

439. Hu H, Yuan G, Wang X, et al. Effects of a diet with or without physical activity on angiopoietin-like protein 8 concentrations in overweight/obese patients with newly diagnosed type 2 diabetes: A randomized controlled trial. *Endocr J* 2019; **66**(1): 89-105. doi:10.1507/endocrj.EJ18-0191

440. Hudson JI, McElroy SL, Raymond NC, et al. Fluvoxamine in the treatment of binge-eating disorder: A multicenter placebo-controlled, double-blind trial. *AM J PSYCHIATRY* 1998; **155**(12): 1756-1762. doi:10.1176/ajp.155.12.1756

441. Huffman C, Stacey BR, Tuchman M, et al. Efficacy and safety of pregabalin in the treatment of patients with painful diabetic peripheral neuropathy and pain on walking. *Clin J Pain* 2015; **31**(11): 946-958. doi:10.1097/AJP.0000000000000198

442. Huibers MJH, Beurskens AJHM, Van Schayck CP, et al. Efficacy of cognitive-behavioural therapy by general practitioners for unexplained fatigue among employees: Randomised controlled trial. *Br J Psychiatry* 2004; **184**(MAR.): 240-246. doi:10.1192/bjp.184.3.240

443. Hull D, Rennie P, Noronha A, et al. Effects of creating a non-specific, virus-hostile environment in the nasopharynx on symptoms and duration of common cold. *Acta Otorhinolaryngol Ital* 2007; **27**(2): 73-77. doi:N

444. Hullender Rubin LE, Mist SD, Schnyer RN, Chao MT, Leclair CM. Acupuncture Augmentation of Lidocaine for Provoked, Localized Vulvodynia: A Feasibility and Acceptability Study. *J Lower Genital Tract Dis* 2019; **23**(4): 279-286. doi:10.1097/LGT.0000000000000489

445. Humayun S, Herlitz L, Chesnokov M, Doolan M, Landau S, Scott S. Randomized controlled trial of Functional Family Therapy for offending and antisocial behavior in UK youth. *J Child Psychol Psychiatry Allied Discip* 2017; **58**(9): 1023-1032. doi:10.1111/jcpp.12743

446. Humphrey N, Barlow A, Wigelsworth M, et al. A cluster randomized controlled trial of the Promoting Alternative Thinking Strategies (PATHS) curriculum. *J Sch Psychol* 2016; **58**: 73-89. doi:10.1016/j.jsp.2016.07.002

447. Håland Haldorsen EM, Grasdal AL, Skouen JS, Risa AE, Kronholm K, Ursin H. Is there a right treatment for a particular patient group? Comparison of ordinary treatment, light multidisciplinary treatment, and extensive multidisciplinary treatment for long-term sick-listed employees with musculoskeletal pain. *Pain* 2002; **95**(1-2): 49-63. doi:10.1016/S0304-3959(01)00374-8

448. Høgelund J, Falgaard Eplov L. Employment effects of a multidisciplinary health assessment for mentally ill persons – A quasi-randomised controlled trial. *Scand J Public Health* 2018; **46**(3): 389-399. doi:10.1177/1403494817723458

449. Högström J, Olofsson V, Özdemir M, Enebrink P, Stattin H. Two-Year Findings from a National Effectiveness Trial: Effectiveness of Behavioral and Non-Behavioral Parenting Programs. *J Abnorm Child Psychol* 2017; **45**(3): 527-542. doi:10.1007/s10802-016-0178-0

450. Ialongo NS, Domitrovich C, Embry D, et al. A randomized controlled trial of the combination of two school-based universal preventive interventions. *Dev Psychol* 2019; **55**(6): 1313-1325. doi:10.1037/dev0000715

451. Ialongo NS, Werthamer L, Kellam SG, Brown CH, Wang S, Lin Y. Proximal impact of two first-grade preventive interventions on the early risk behaviors for later substance abuse, depression, and antisocial behavior. *Am J Community Psychol* 1999; **27**(5): 599-641. doi:10.1023/A:1022137920532

452. Ibrahim AA, Hussein HM, Ali MS, et al. A randomized controlled trial examining the impact of low vs. moderate-intensity aerobic training in post-discharge COVID-19 older subjects. *Eur Rev Med Pharmacol Sci* 2023; **27**(9): 4280-4291. doi:10.26355/eurrev_202305_32338

453. Impey L, Reynolds M, MacQuillan K, Gates S, Murphy J, Sheil O. Admission cardiotocography: A randomised controlled trial. *Lancet* 2003; **361**(9356): 465-470. doi:10.1016/S0140-6736(03)12464-6

454. Jamilian M, Asemi Z. The effect of soy intake on metabolic profiles of women with gestational diabetes mellitus. *J Clin Endocrinol Metab* 2015; **100**(12): 4654-4661. doi:10.1210/jc.2015-3454

455. Jardin BF, Wulfert E. The use of messages in altering risky gambling behavior in experienced gamblers. *Psychol Addict Behav* 2012; **26**(1): 166-170. doi:10.1037/a0026202

456. Jariani M, Saaki M, Nazari H, Birjandi M. The effect of Olanzapine and Sertraline on personality disorder in patients with methadone maintenance therapy. *Psychiatr Danub* 2010; **22**(4): 544-547. doi:N

457. Jenkins DJA, Kendall CWC, McKeown-Eyssen G, et al. Effect of a low-glycemic index or a high-cereal fiber diet on type 2 diabetes: A randomized trial. *J Am Med Assoc* 2008; **300**(23): 2742-2753. doi:10.1001/jama.2008.808

458. Jensen C, Jensen OK, Christiansen DH, Nielsen CV. One-year follow-up in employees sick-listed because of low back pain: Randomized clinical trial comparing multidisciplinary and brief intervention. *Spine* 2011; **36**(15): 1180-1189. doi:10.1097/BRS.0b013e3181eba711

459. Jensen C, Jensen OK, Nielsen CV. Sustainability of return to work in sick-listed employees with low-back pain. Two-year follow-up in a randomized clinical trial comparing multidisciplinary and brief intervention. *BMC Musculoskelet Disord* 2012; **13**. doi:10.1186/1471-2474-13-156

460. Jensen IB, Bergström G, Ljungquist T, Bodin L. A 3-year follow-up of a multidisciplinary rehabilitation programme for back and neck pain. *Pain* 2005; **115**(3): 273-283. doi:10.1016/j.pain.2005.03.005

461. Jensen IB, Bergström G, Ljungquist T, Bodin L, Nygren AL. A randomized controlled component analysis of a behavioral medicine rehabilitation program for chronic spinal pain: Are the effects dependent on gender? *Pain* 2001; **91**(1-2): 65-78. doi:10.1016/S0304-3959(00)00420-6

462. Jimeno-Almazán A, Buendía-Romero Á, Martínez-Cava A, et al. Effects of a concurrent training, respiratory muscle exercise, and selfmanagement recommendations on recovery from post-COVID-19 conditions: the RECOVE trial. *J Appl Physiol* 2023; **134**(1): 95-104. doi:10.1152/japplphysiol.00489.2022

463. Jimeno-Almazán A, Franco-López F, Buendía-Romero Á, et al. Rehabilitation for post-COVID-19 condition through a supervised exercise intervention: A randomized controlled trial. *Scand J Med Sci Sports* 2022; **32**(12): 1791-1801. doi:10.1111/sms.14240

464. Johansen MY, Macdonald CS, Hansen KB, et al. Effect of an intensive lifestyle intervention on glycemic control in patients with type 2 diabetes: A randomized clinical trial. *JAMA* 2017; **318**(7): 637-646. doi:10.1001/jama.2017.10169

465. Joseph P, Pari R, Miller S, et al. Neurovascular Dysregulation and Acute Exercise Intolerance in Myalgic Encephalomyelitis/Chronic Fatigue Syndrome: A Randomized, Placebo-Controlled Trial of Pyridostigmine. *Chest* 2022; **162**(5): 1116-1126. doi:10.1016/j.chest.2022.04.146

466. Juarascio AS, Parker MN, Hunt R, Murray HB, Presseller EK, Manasse SM. Mindfulness and acceptance-based behavioral treatment for bulimia-spectrum disorders: A pilot feasibility randomized trial. *Int J Eating Disord* 2021; **54**(7): 1270-1277. doi:10.1002/eat.23512

467. Jørgensen CR, Freund C, Bøye R, Jordet H, Andersen D, Kjølbye M. Outcome of mentalization-based and supportive psychotherapy in patients with borderline personality disorder: A randomized trial. *Acta Psychiatr Scand* 2013; **127**(4): 305-317. doi:10.1111/j.1600-0447.2012.01923.x

468. Jørgensen MS, Storebø OJ, Bo S, et al. Mentalization-based treatment in groups for adolescents with Borderline Personality Disorder: 3- and 12-month follow-up of a randomized controlled trial. *Eur Child Adolesc Psychiatry* 2021; **30**(5): 699-710. doi:10.1007/s00787-020-01551-2

469. Kaess M, Edinger A, Fischer-Waldschmidt G, Parzer P, Brunner R, Resch F. Effectiveness of a brief psychotherapeutic intervention compared with treatment as usual for adolescent nonsuicidal self-injury: a single-centre, randomised controlled trial. *Eur Child Adolesc Psychiatry* 2020; **29**(6): 881-891. doi:10.1007/s00787-019-01399-1

470. Kahleova H, Matoulek M, Malinska H, et al. Vegetarian diet improves insulin resistance and oxidative stress markers more than conventional diet in subjects with Type2 diabetes. *Diabet Med* 2011; **28**(5): 549-559. doi:10.1111/j.1464-5491.2010.03209.x

471. Kaminer Y, Burleson JA, Goldston DB, Burke RH. Suicidal ideation among adolescents with alcohol use disorders during treatment and aftercare. *Am J Addict* 2006; **15**(SUPPL. 1): s43-s49. doi:10.1080/10550490601006154

472. Karasimopoulou S, Derri V, Zervoudaki E. Children's perceptions about their health-related quality of life: Effects of a health education-social skills program. *Health Educ Res* 2012; **27**(5): 780-793. doi:10.1093/her/cys089

473. Karlsson B, Burell G, Anderberg UM, Svärdsudd K. Cognitive behaviour therapy in women with fibromyalgia: A randomized clinical trial. *Scand J Pain* 2015; **9**(1): 11-21. doi:10.1016/j.sjpain.2015.04.027

474. Karp JF, Gao X, Wahed AS, et al. Effect of Problem-Solving Therapy Versus Supportive Management in Older Adults with Low Back Pain and Depression While on Antidepressant Pharmacotherapy. *Am J Geriatr Psychiatry* 2018; **26**(7): 765-777. doi:10.1016/j.jagp.2018.01.004

475. Kashifard M, Basirat Z, Kashifard M, Golsorkhtabar-Amiri M, Moghaddamnia A. Ondansetrone or metoclopromide? Which is more effective in severe nausea and vomiting of pregnancy? A randomized trial double-blind study. *Clin Exp Obstet Gynecol* 2013; **40**(1): 127-130. doi:N

476. Kaster TS, Daskalakis ZJ, Noda Y, et al. Efficacy, tolerability, and cognitive effects of deep transcranial magnetic stimulation for late-life depression: a prospective randomized controlled trial. *Neuropsychopharmacology* 2018; **43**(11): 2231-2238. doi:10.1038/s41386-018-0121-x

477. Kaye WH, Nagata T, Weltzin TE, et al. Double-blind placebo-controlled administration of fluoxetine in restricting- and restricting-purging-type anorexia nervosa. *Biol Psychiatry* 2001; **49**(7): 644-652. doi:10.1016/S0006-3223(00)01013-1

478. Kayo AH, Peccin MS, Sanches CM, Trevisani VFM. Effectiveness of physical activity in reducing pain in patients with fibromyalgia: A blinded randomized clinical trial. *Rheumatol Int* 2012; **32**(8): 2285-2292. doi:10.1007/s00296-011-1958-z

479. Kelly AC, Carter JC. Self-compassion training for binge eating disorder: A pilot randomized controlled trial. *Psychol Psychother Theory Res Pract* 2015; **88**(3): 285-303. doi:10.1111/papt.12044

480. Kemp DE, Gao K, Ganocy SJ, et al. A 6-month, double-blind, maintenance trial of lithium monotherapy versus the combination of lithium and divalproex for rapid-cycling bipolar disorder and co-occurring substance abuse or dependence. *J CLIN PSYCHIATRY* 2009; **70**(1): 113-121. doi:10.4088/JCP.07m04022

481. Kendall AD, Emerson EM, Hartmann WE, Zinbarg RE, Donenberg GR. A Two-Week Psychosocial Intervention Reduces Future Aggression and Incarceration in Clinically Aggressive Juvenile Offenders. *J AM ACAD CHILD ADOLESC PSYCHIATRY* 2017; **56**(12): 1053-1061. doi:10.1016/j.jaac.2017.09.424

482. Kennard BD, Goldstein T, Foxwell AA, et al. As safe as possible (ASAP): A brief app-supported inpatient intervention to prevent postdischarge suicidal behavior in Hospitalized, Suicidal Adolescents. *AM J PSYCHIATRY* 2018; **175**(9): 864-872. doi:10.1176/appi.ajp.2018.17101151

483. Kerget B, Çil G, Araz Ö, Alper F, Akgün M. Comparison of two antifibrotic treatments for lung fibrosis in post-COVID-19 syndrome: A randomized, prospective study. *Med Clin* 2023; **160**(12): 525-530. doi:10.1016/j.medcli.2022.12.021

484. Kerling A, Beyer S, Dirks M, et al. Effects of a randomized-controlled and online-supported physical activity intervention on exercise capacity, fatigue and health related quality of life in patients with post-COVID-19 syndrome. *BMC Sports Sci Med Rehabil* 2024; **16**(1): 33. doi:10.1186/s13102-024-00817-5

485. Keshen AR, Dixon L, Ali SI, et al. A feasibility study evaluating lisdexamfetamine dimesylate for the treatment of adults with bulimia nervosa. *Int J Eating Disord* 2021; **54**(5): 872-878. doi:10.1002/eat.23480

486. Keulen JK, Bruinsma A, Kortekaas JC, et al. Induction of labour at 41 weeks versus expectant management until 42 weeks (INDEX): Multicentre, randomised non-inferiority trial. *BMJ (Online)* 2019; **364**. doi:10.1136/bmj.l344

487. Keus Van De Poll M, Nybergh L, Lornudd C, et al. Preventing sickness absence among employees with common mental disorders or stress-related symptoms at work: A cluster randomised controlled trial of a problem-solving-based intervention conducted by the Occupational Health Services. *Occupational and Environmental Medicine* 2020; **77**(7): 454-461. doi:10.1136/oemed-2019-106353

488. Khalsa SBS, Hickey-Schultz L, Cohen D, Steiner N, Cope S. Evaluation of the mental health benefits of yoga in a secondary school: A preliminary randomized controlled trial. *J Behav Health Serv Res* 2012; **39**(1): 80-90. doi:10.1007/s11414-011-9249-8

489. Kharouba J, Ratson T, Somri M, Blumer S. Preemptive analgesia by paracetamol, ibuprofen or placebo in pediatric dental care: A randomized controlled study. *J Clin Pediatr Dent* 2019; **43**(1): 51-55. doi:10.17796/1053-4625-43.1.10

490. Kim DR, Wang E, McGeehan B, et al. Randomized controlled trial of transcranial magnetic stimulation in pregnant women with major depressive disorder. *Brain Stimul* 2019; **12**(1): 96-102. doi:10.1016/j.brs.2018.09.005

491. Kim E, Cain KC, Webster-Stratton C. The preliminary effect of a parenting program for Korean American mothers: A randomized controlled experimental study. *Int J Nurs Stud* 2008; **45**(9): 1261-1273. doi:10.1016/j.ijnurstu.2007.10.002

492. Kim TY, Bauer DC, McNabb BL, et al. Comparison of BMD Changes and Bone Formation Marker Levels 3 Years After Bisphosphonate Discontinuation: FLEX and HORIZON-PFT Extension I Trials. *J Bone Miner Res* 2019; **34**(5): 810-816. doi:10.1002/jbmr.3654

493. Kindt KCM, Kleinjan M, Janssens JMAM, Scholte RHJ. Evaluation of a school-based depression prevention program among adolescents from low-income areas: A randomized controlled effectiveness trial. *Int J Environ Res Public Health* 2014; **11**(5): 5273-5293. doi:10.3390/ijerph110505273

494. Kirby JN, Sanders MR. A randomized controlled trial evaluating a parenting program designed specifically for grandparents. *Behav Res Ther* 2014; **52**(1): 35-44. doi:10.1016/j.brat.2013.11.002

495. Kirby RL, Miller WC, Routhier F, et al. Effectiveness of a Wheelchair Skills Training Program for Powered Wheelchair Users: A Randomized Controlled Trial. *Arch Phys Med Rehabil* 2015; **96**(11): 2017-2026.e2013. doi:10.1016/j.apmr.2015.07.009

496. Kirby RL, Mitchell D, Sabharwal S, McCranie M, Nelson AL. Manual wheelchair skills training for community-dwelling veterans with spinal cord injury: A randomized controlled trial. *PLoS ONE* 2016; **11**(12). doi:10.1371/journal.pone.0168330

497. Kirchner S, Till B, Plöderl M, Niederkrotenthaler T. Effects of "It Gets Better" Suicide Prevention Videos on Youth Identifying as Lesbian, Gay, Bisexual, Transgender, Queer, or Other Sexual or Gender Minorities: A Randomized Controlled Trial. *LGBT Health* 2022; **9**(6): 436-446. doi:10.1089/lgbt.2021.0383

498. Kiropoulos LA, Klein B, Austin DW, et al. Is internet-based CBT for panic disorder and agoraphobia as effective as face-to-face CBT? *J Anxiety Disord* 2008; **22**(8): 1273-1284. doi:10.1016/j.janxdis.2008.01.008

499. Kitchener HC, Almonte M, Thomson C, et al. HPV testing in combination with liquid-based cytology in primary cervical screening (ARTISTIC): a randomised controlled trial. *Lancet Oncol* 2009; **10**(7): 672-682. doi:10.1016/S1470-2045(09)70156-1

500. Kitzman H, Olds DL, Knudtson MD, et al. Prenatal and infancy nurse home visiting and 18-year outcomes of a randomized trial. *Pediatrics* 2019; **144**(6). doi:10.1542/peds.2018-3876

501. Kivi M, Eriksson MCM, Hange D, et al. Internet-Based Therapy for Mild to Moderate Depression in Swedish Primary Care: Short Term Results from the PRIM-NET Randomized Controlled Trial. *Cogn Behav Ther* 2014; **43**(4): 289-298. doi:10.1080/16506073.2014.921834

502. Kiviruusu O, Björklund K, Koskinen HL, et al. Short-term effects of the "Together at School" intervention program on children's socio-emotional skills: A cluster randomized controlled trial. *BMC Psychol* 2016; **4**(1). doi:10.1186/s40359-016-0133-4

503. Kjøbli J, Hukkelberg S, Ogden T. A randomized trial of group parent training: Reducing child conduct problems in real-world settings. *Behav Res Ther* 2013; **51**(3): 113-121. doi:10.1016/j.brat.2012.11.006

504. Kleber HD, Weissman MM, Rounsaville BJ, Wilber CH, Prusoff BA, Riordan CE. Imipramine as Treatment for Depression in Addicts. *Arch Gen Psychiatry* 1983; **40**(6): 649-653. doi:10.1001/archpsyc.1983.04390010059007

505. Kleefman M, Jansen DEMC, Stewart RE, Reijneveld SA. The effectiveness of Stepping Stones Triple P parenting support in parents of children with borderline to mild intellectual disability and psychosocial problems: A randomized controlled trial. *BMC Med* 2014; **12**(1). doi:10.1186/s12916-014-0191-5

506. Klein AS, Skinner JB, Hawley KM. Targeting binge eating through components of dialectical behavior therapy: Preliminary outcomes for individually supported diary card self-monitoring versus group-based DBT. *Psychotherapy* 2013; **50**(4): 543-552. doi:10.1037/a0033130

507. Kling A, Forster M, Sundell K, Melin L. A Randomized Controlled Effectiveness Trial of Parent Management Training With Varying Degrees of Therapist Support. *Behav Ther* 2010; **41**(4): 530-542. doi:10.1016/j.beth.2010.02.004

508. Klírová M, Adamová A, Biačková N, et al. Transcranial direct current stimulation (tDCS) in the treatment of neuropsychiatric symptoms of long COVID. *Sci Rep* 2024; **14**(1). doi:10.1038/s41598-024-52763-4

509. Kogel A, Machatschek M, Scharschmidt R, et al. Physical exercise as a treatment for persisting symptoms post-COVID infection: review of ongoing studies and prospective randomized controlled training study. *Clin Res Cardiol* 2023; **112**(11): 1699-1709. doi:10.1007/s00392-023-02300-6

510. Konstenius M, Jayaram-Lindström N, Guterstam J, Beck O, Philips B, Franck J. Methylphenidate for attention deficit hyperactivity disorder and drug relapse in criminal offenders with substance dependence: A 24-week randomized placebo-controlled trial. *Addiction* 2014; **109**(3): 440-449. doi:10.1111/add.12369

511. Kool J, Bachmann S, Oesch P, et al. Function-Centered Rehabilitation Increases Work Days in Patients With Nonacute Nonspecific Low Back Pain: 1-Year Results From a Randomized Controlled Trial. *Arch Phys Med Rehabil* 2007; **88**(9): 1089-1094. doi:10.1016/j.apmr.2007.05.022

512. Korkmaz YT, Kayıpmaz S, Senel FC, Atasoy KT, Gumrukcu Z. Does additional cone beam computed tomography decrease the risk of inferior alveolar nerve injury in high-risk cases undergoing third molar surgery?Does CBCT decrease the risk of IAN injury? *Int J Oral Maxillofac Surg* 2017; **46**(5): 628-635. doi:10.1016/j.ijom.2017.01.001

513. Kpaeyeh J, Mar PL, Raj V, et al. Hemodynamic profiles and tolerability of modafinil in the treatment of postural tachycardia syndrome a randomized, placebo-controlled trial. *J Clin Psychopharmacol* 2014; **34**(6): 738-741. doi:10.1097/JCP.0000000000000221

514. Kramer U, Pascual-Leone A, Berthoud L, et al. Assertive Anger Mediates Effects of Dialectical Behaviour-informed Skills Training for Borderline Personality Disorder: A Randomized Controlled Trial. *Clin Psychol Psychother* 2016; **23**(3): 189-202. doi:10.1002/cpp.1956

515. Kranzler HR, Mueller T, Cornelius J, et al. Sertraline treatment of co-occurring alcohol dependence and major depression. *J Clin Psychopharmacol* 2006; **26**(1): 13-20. doi:10.1097/01.jcp.0000194620.61868.35

516. Krebs JD, Elley CR, Parry-Strong A, et al. The Diabetes Excess Weight Loss (DEWL) Trial: A randomised controlled trial of high-protein versus high-carbohydrate diets over 2 years in type 2 diabetes. *Diabetologia* 2012; **55**(4): 905-914. doi:10.1007/s00125-012-2461-0

517. Kuut TA, Müller F, Csorba I, et al. Efficacy of Cognitive-Behavioral Therapy Targeting Severe Fatigue Following Coronavirus Disease 2019: Results of a Randomized Controlled Trial. *Clin Infect Dis* 2023; **77**(5): 687-695. doi:10.1093/cid/ciad257

518. Kuyken W, Ball S, Crane C, et al. Effectiveness and cost-effectiveness of universal school-based mindfulness training compared with normal school provision in reducing risk of mental health problems and promoting well-being in adolescence: The MYRIAD cluster randomised controlled trial. *Evid -Based Ment Health* 2022; **25**(3): 99-109. doi:10.1136/ebmental-2021-300396

519. Kääpä EH, Frantsi K, Sarna S, Malmivaara A. Multidisciplinary group rehabilitation Versus individual physiotherapy for chronic nonspecific low back pain: A randomized trial. *Spine* 2006; **31**(4): 371-376. doi:10.1097/01.brs.0000200104.90759.8c

520. Labib ME, Hassanein OE, Moussa M, Yassen A, Schwendicke F. Selective versus stepwise removal of deep carious lesions in permanent teeth: A randomised controlled trial from Egypt - An interim analysis. *BMJ Open* 2019; **9**(9). doi:10.1136/bmjopen-2019-030957

521. Lake MF, Johnson TM, Murphy J, Knuppel RA. Evaluation of a perinatal grief support team. *AM J OBSTET GYNECOL* 1987; **157**(5): 1203-1206. doi:10.1016/S0002-9378(87)80295-8

522. Lammerts L, Schaafsma FG, Bonefaas-Groenewoud K, van Mechelen W, Anema JR. Effectiveness of a return-to-work program for workers without an employment contract, sick-listed due to common mental disorders. *Scand J Work Environ Health* 2016; **42**(6): 469-480. doi:10.5271/sjweh.3588

523. Lander F, Friche C, Tornemand H, Andersen JH, Kirkeskov L. Can we enhance the ability to return to work among workers with stress-related disorders? *BMC Public Health* 2009; **9**. doi:10.1186/1471-2458-9-372

524. Landon MB, Spong CY, Thom E, et al. A multicenter, randomized trial of treatment for mild gestational diabetes. *New Engl J Med* 2009; **361**(14): 1339-1348. doi:10.1056/NEJMoa0902430

525. Landry SH, Zucker TA, Taylor HB, et al. Enhancing early child care quality and learning for toddlers at risk: The responsive early childhood program. *Dev Psychol* 2014; **50**(2): 526-541. doi:10.1037/a0033494

526. Langagergaard V, Jensen OK, Nielsen CV, et al. The comparative effects of brief or multidisciplinary intervention on return to work at 1 year in employees on sick leave due to low back pain: A randomized controlled trial. *Clin Rehabil* 2021; **35**(9): 1290-1304. doi:10.1177/02692155211005387

527. Langlais EL, Lefebvre J, Maheux-Lacroix S, Bujold E, Fortier M, Bouchard C. Treatment of Secondary Vestibulodynia with Conjugated Estrogen Cream: A Pilot, Double-Blind, Randomized Placebo-Controlled Trial. *J Obstet Gynaecol Can* 2017; **39**(6): 453-458. doi:10.1016/j.jogc.2016.10.011

528. Larose MP, Ouellet-Morin I, Vergunst F, et al. Examining the impact of a social skills training program on preschoolers' social behaviors: A cluster-randomized controlled trial in child care centers. *BMC Psychol* 2020; **8**(1). doi:10.1186/s40359-020-00408-2

529. Larsen RN, Mann NJ, Maclean E, Shaw JE. The effect of high-protein, low-carbohydrate diets in the treatment of type 2 diabetes: A 12 month randomised controlled trial. *Diabetologia* 2011; **54**(4): 731-740. doi:10.1007/s00125-010-2027-y

530. Lasa A, Miranda J, Bulló M, et al. Comparative effect of two Mediterranean diets versus a low-fat diet on glycaemic control in individuals with type 2 diabetes. *Eur J Clin Nutr* 2014; **68**(7): 767-772. doi:10.1038/ejcn.2014.1

531. Lassander M, Hintsanen M, Suominen S, Mullola S, Vahlberg T, Volanen SM. Effects of school-based mindfulness intervention on health-related quality of life: moderating effect of gender, grade, and independent practice in cluster randomized controlled trial. *Qual Life Res* 2021; **30**(12): 3407-3419. doi:10.1007/s11136-021-02868-4

532. Lau RI, Su Q, Lau ISF, et al. A synbiotic preparation (SIM01) for post-acute COVID-19 syndrome in Hong Kong (RECOVERY): a randomised, double-blind, placebo-controlled trial. *Lancet Infect Dis* 2024; **24**(3): 256-265. doi:10.1016/S1473-3099(23)00685-0

533. Laurenssen EMP, Luyten P, Kikkert MJ, et al. Day hospital mentalization-based treatment v. specialist treatment as usual in patients with borderline personality disorder: Randomized controlled trial. *Psychol Med* 2018; **48**(15): 2515-2521. doi:10.1017/S0033291718000132

534. Laurenzi A, Bolla AM, Panigoni G, et al. Effects of carbohydrate counting on glucose control and quality of life over 24 weeks in adult patients with type 1 diabetes on continuous subcutaneous insulin infusion: A randomized, prospective clinical trial (GIOCAR). *Diabetes Care* 2011; **34**(4): 823-827. doi:10.2337/dc10-1490

535. Lazo M, Solga SF, Horska A, et al. Effect of a 12-month intensive lifestyle intervention on hepatic steatosis in adults with type 2 diabetes. *Diabetes Care* 2010; **33**(10): 2156-2163. doi:10.2337/dc10-0856

536. Le Grange D, Lock J, Agras WS, Bryson SW, Jo B. Randomized Clinical Trial of Family-Based Treatment and Cognitive-Behavioral Therapy for Adolescent Bulimia Nervosa. *J AM ACAD CHILD ADOLESC PSYCHIATRY* 2015; **54**(11): 886-894.e882. doi:10.1016/j.jaac.2015.08.008

537. Lean ME, Leslie WS, Barnes AC, et al. Primary care-led weight management for remission of type 2 diabetes (DiRECT): an open-label, cluster-randomised trial. *Lancet* 2018; **391**(10120): 541-551. doi:10.1016/S0140-6736(17)33102-1

538. Lean MEJ, Leslie WS, Barnes AC, et al. Durability of a primary care-led weight-management intervention for remission of type 2 diabetes: 2-year results of the DiRECT open-label, cluster-randomised trial. *Lancet Diabetes Endocrinol* 2019; **7**(5): 344-355. doi:10.1016/S2213-8587(19)30068-3

539. Lee SH, Ahn SH, Cheong YS. Effect of electronic cigarettes on smoking reduction and cessation in Korean Male smokers: A randomized controlled study. *J Am Board Fam Med* 2019; **32**(4): 567-574. doi:10.3122/jabfm.2019.04.180384

540. Lee SM, Tenney R, Wallace AW, Arjomandi M. E-cigarettes versus nicotine patches for perioperative smoking cessation: A pilot randomized trial. *PeerJ* 2018; **2018**(9): e5609. doi:10.7717/peerj.5609

541. Lemstra M, Olszynski WP. The effectiveness of multidisciplinary rehabilitation in the treatment of fibromyalgia: A randomized controlled trial. *Clin J Pain* 2005; **21**(2): 166-174. doi:10.1097/00002508-200503000-00008

542. Lenze SN, Potts MA. Brief Interpersonal Psychotherapy for depression during pregnancy in a low-income population: A randomized controlled trial. *Journal of Affective Disorders* 2017; **210**: 151-157. doi:10.1016/j.jad.2016.12.029

543. Leone SS, Huibers MJH, Kant I, et al. Long-term efficacy of cognitive-behavioral therapy by general practitioners for fatigue: A 4-year follow-up study. *J Psychosom Res* 2006; **61**(5): 601-607. doi:10.1016/j.jpsychores.2006.04.010

544. Lera S, Gelman SM, López MJ, et al. Multidisciplinary treatment of fibromyalgia: Does cognitive behavior therapy increase the response to treatment? *J Psychosom Res* 2009; **67**(5): 433-441. doi:10.1016/j.jpsychores.2009.01.012

545. Lerner DK, Garvey KL, Arrighi-Allisan A, et al. Omega-3 Fatty Acid Supplementation for the Treatment of Persistent COVID-Related Olfactory Dysfunction. *Am J Rhinol and Allergy* 2023; **37**(5): 531-540. doi:10.1177/19458924231174799

546. Lesser H, Sharma U, LaMoreaux L, Poole RM. Pregabalin relieves symptoms of painful diabetic neuropathy: A randomized controlled trial. *Neurology* 2004; **63**(11): 2104-2110. doi:10.1212/01.WNL.0000145767.36287.A1

547. Leung SS, Lee AM, Wong DF, et al. A brief group intervention using a cognitive-behavioural approach to reduce postnatal depressive symptoms: a randomised controlled trial. *Hong Kong Med J* 2016; **22**: S4-S8. doi:N

548. Lev-Sagie A, Kopitman A, Brzezinski A. Low-Level Laser Therapy for the Treatment of Provoked Vestibulodynia—A Randomized, Placebo-Controlled Pilot Trial. *J Sex Med* 2017; **14**(11): 1403-1411. doi:10.1016/j.jsxm.2017.09.004

549. Lévesque S, Lessard MR, Nicole PC, et al. Efficacy of a T-piece system and a continuous positive airway pressure system for apnea testing in the diagnosis of brain death. *Crit Care Med* 2006; **34**(8): 2213-2216. doi:10.1097/01.CCM.0000215114.46127.DA

550. Levin FR, Evans SM, Brooks DJ, Garawi F. Treatment of cocaine dependent treatment seekers with adult ADHD: Double-blind comparison of methylphenidate and placebo. *Drug Alcohol Depend* 2007; **87**(1): 20-29. doi:10.1016/j.drugalcdep.2006.07.004

551. Levin FR, Evans SM, Brooks DJ, Kalbag AS, Garawi F, Nunes EV. Treatment of methadone-maintained patients with adult ADHD: Double-blind comparison of methylphenidate, bupropion and placebo. *Drug Alcohol Depend* 2006; **81**(2): 137-148. doi:10.1016/j.drugalcdep.2005.06.012

552. Levin FR, Mariani J, Brooks DJ, et al. A randomized double-blind, placebo-controlled trial of venlafaxine-extended release for co-occurring cannabis dependence and depressive disorders. *Addiction* 2013; **108**(6): 1084-1094. doi:10.1111/add.12108

553. Levin FR, Mariani JJ, Specker S, et al. Extended-release mixed amphetamine salts vs placebo for comorbid adult attention-deficit/hyperactivity disorder and cocaine use disorder a randomized clinical trial. *JAMA Psychiatry* 2015; **72**(6): 593-602. doi:10.1001/jamapsychiatry.2015.41

554. Levine LR, Fluoxetine Bulimia Nervosa Collaborative Study G. Fluoxetine in the Treatment of Bulimia Nervosa: A Multicenter, Placebo-Controlled, Double-blind Trial. *Arch Gen Psychiatry* 1992; **49**(2): 139-147. doi:10.1001/archpsyc.1992.01820020059008

555. Levkovitz Y, Isserles M, Padberg F, et al. Efficacy and safety of deep transcranial magnetic stimulation for major depression: A prospective multicenter randomized controlled trial. *World Psychiatry* 2015; **14**(1): 64-73. doi:10.1002/wps.20199

556. Li HJ, Martinez PE, Li X, et al. Transdermal estradiol for postpartum depression: results from a pilot randomized, double-blind, placebo-controlled study. *Arch Women's Ment Health* 2020; **23**(3): 401-412. doi:10.1007/s00737-019-00991-3

557. Li J, Xia W, Zhan C, et al. A telerehabilitation programme in post-discharge COVID-19 patients (TERECO): A randomised controlled trial. *Thorax* 2022; **77**(7): 697-706. doi:10.1136/thoraxjnl-2021-217382

558. Lie SA, Eriksen HR, Ursin H, Hagen EM. A multi-state model for sick-leave data applied to a randomized control trial study of low back pain. *Scand J Public Health* 2008; **36**(3): 279-283. doi:10.1177/1403494807086979

559. Lin TJ, Ko HC, Wu JYW, Oei TP, Lane HY, Chen CH. The Effectiveness of Dialectical Behavior Therapy Skills Training Group vs. Cognitive Therapy Group on Reducing Depression and Suicide Attempts for Borderline Personality Disorder in Taiwan. *Arch Suicide Res* 2019; **23**(1): 82-99. doi:10.1080/13811118.2018.1436104

560. Lindberg F, Nelson I, Ranstam J, Riker DK. Early intervention with a glycerol throat spray containing cold-adapted cod trypsin after selfdiagnosis of common cold: A randomised trial. *PLoS ONE* 2022; **17**(7 July). doi:10.1371/journal.pone.0270699

561. Lindell O, Johansson SE, Strender LE. Subacute and chronic, non-specific back and neck pain: Cognitive- behavioural rehabilitation versus primary care. A randomized controlled trial. *BMC Musculoskelet Disord* 2008; **9**. doi:10.1186/1471-2474-9-172

562. Lindquist B, Emilson CG. Sealing Proximal Non- And Micro-Cavitated Carious Lesions Using a One-Session Separator Technique: A 2-Year Randomised Clinical Study. *Caries Res* 2020; **54**(5-6): 483-490. doi:10.1159/000509679

563. Linehan MM, Armstrong HE, Suarez A, Allmon D, Heard HL. Cognitive-Behavioral Treatment of Chronically Parasuicidal Borderline Patients. *Arch Gen Psychiatry* 1991; **48**(12): 1060-1064. doi:10.1001/archpsyc.1991.01810360024003

564. Linehan MM, Comtois KA, Murray AM, et al. Two-year randomized controlled trial and follow-up of dialectical behavior therapy vs therapy by experts for suicidal behaviors and borderline personality disorder. *Arch Gen Psychiatry* 2006; **63**(7): 757-766. doi:10.1001/archpsyc.63.7.757

565. Linehan MM, Heard HL, Armstrong HE. Naturalistic Follow-up of a Behavioral Treatment for Chronically Parasuicidal Borderline Patients. *Arch Gen Psychiatry* 1993; **50**(12): 971-974. doi:10.1001/archpsyc.1993.01820240055007

566. Linehan MM, Korslund KE, Harned MS, et al. Dialectical behavior therapy for high suicide risk in individuals with borderline personality disorder: A randomized clinical trial and component analysis. *JAMA Psychiatry* 2015; **72**(5): 475-482. doi:10.1001/jamapsychiatry.2014.3039

567. Linehan MM, Schmidt Iii H, Dimeff LA, Craft JC, Kanter J, Comtois KA. Dialectical behavior therapy for patients with borderline personality disorder and drug-dependence. *Am J Addict* 1999; **8**(4): 279-292. doi:10.1080/105504999305686

568. Linehan MM, Tutek DA, Heard HL, Armstrong HE. Interpersonal outcome of cognitive behavioral treatment for chronically suicidal borderline patients. *AM J PSYCHIATRY* 1994; **151**(12): 1771-1776. doi:10.1176/ajp.151.12.1771

569. Liss DT, Finch EA, Cooper A, et al. One-year effects of a group-based lifestyle intervention in adults with type 2 diabetes: A randomized encouragement trial. *Diabetes Res Clin Pract* 2018; **140**: 36-44. doi:10.1016/j.diabres.2018.03.030

570. Liu BY, Lo ECM, Chu CH, Lin HC. Randomized trial on fluorides and sealants for fissure caries prevention. *J Dent Res* 2012; **91**(8): 753-758. doi:10.1177/0022034512452278

571. Liu X, Li Z, Liu S, et al. Potential therapeutic effects of dipyridamole in the severely ill patients with COVID-19. *Acta Pharm Sin B* 2020; **10**(7): 1205-1215. doi:10.1016/j.apsb.2020.04.008

572. Lo ECM, Luo Y, Tan HP, Dyson JE, Corbet EF. ART and conventional root restorations in elders after 12 months. *J Dent Res* 2006; **85**(10): 929-932. doi:10.1177/154405910608501011

573. Lochman JE, Dishion TJ, Powell NP, Boxmeyer CL, Qu L, Sallee M. Evidence-based preventive intervention for preadolescent aggressive children: One-year outcomes following randomization to group versus individual delivery. *J Consult Clin Psychol* 2015; **83**(4): 728-735. doi:10.1037/ccp0000030

574. Lochman JE, Wells KC. Contextual social-cognitive mediators and child outcome: A test of the theoretical model in the Coping Power program. *Dev Psychopathol* 2002; **14**(4): 945-967. doi:10.1017/S0954579402004157

575. Lochman JE, Wells KC. The coping power program at the middle-school transition: Universal and indicated prevention effects. *Psychol Addict Behav* 2002; **16**(SUPPL. 14): S40-S54. doi:10.1037/0893-164x.16.4s.s40

576. Lochman JE, Wells KC. The coping power program for preadolescent aggressive boys and their parents: Outcome effects at the 1-year follow-up. *J Consult Clin Psychol* 2004; **72**(4): 571-578. doi:10.1037/0022-006X.72.4.571

577. Lochman JE, Wells KC, Qu L, Chen L. Three Year Follow-Up of Coping Power Intervention Effects: Evidence of Neighborhood Moderation? *Prev Sci* 2013; **14**(4): 364-376. doi:10.1007/s11121-012-0295-0

578. Lock J, Le Grange D, Agras WS, Moye A, Bryson SW, Jo B. Randomized clinical trial comparing family-based treatment with adolescent-focused individual therapy for adolescents with anorexia nervosa. *Arch Gen Psychiatry* 2010; **67**(10): 1025-1032. doi:10.1001/archgenpsychiatry.2010.128

579. Longobardi I, Goessler K, De Oliveira Júnior GN, et al. Effects of a 16-week home-based exercise training programme on health-related quality of life, functional capacity, and persistent symptoms in survivors of severe/critical COVID-19: A randomised controlled trial. *Br J Sports Med* 2023; **57**(20): 1295-1303. doi:10.1136/bjsports-2022-106681

580. Loughnan SA, Butler C, Sie AA, et al. A randomised controlled trial of ‘MUMentum postnatal’: Internet-delivered cognitive behavioural therapy for anxiety and depression in postpartum women. *Behav Res Ther* 2019; **116**: 94-103. doi:10.1016/j.brat.2019.03.001

581. Loughnan SA, Sie A, Hobbs MJ, et al. A randomized controlled trial of ‘MUMentum Pregnancy’: Internet-delivered cognitive behavioral therapy program for antenatal anxiety and depression. *Journal of Affective Disorders* 2019; **243**: 381-390. doi:10.1016/j.jad.2018.09.057

582. Louie JCY, Markovic TP, Perera N, et al. A randomized controlled trial investigating the effects of a low-glycemic index diet on pregnancy outcomes in gestational diabetes mellitus. *Diabetes Care* 2011; **34**(11): 2341-2346. doi:10.2337/dc11-0985

583. Louie JCY, Markovic TP, Ross GP, Foote D, Brand-Miller JC. Effect of a low glycaemic index diet in gestational diabetes mellitus on post-natal outcomes after 3 months of birth: A pilot follow-up study. *Matern Child Nutr* 2015; **11**(3): 409-414. doi:10.1111/mcn.12039

584. Low S, Cook CR, Smolkowski K, Buntain-Ricklefs J. Promoting social-emotional competence: An evaluation of the elementary version of Second Step®. *J Sch Psychol* 2015; **53**(6): 463-477. doi:10.1016/j.jsp.2015.09.002

585. Low S, Smolkowski K, Cook C, Desfosses D. Two-year impact of a universal social-emotional learning curriculum: Group differences from developmentally sensitive trends over time. *Dev Psychol* 2019; **55**(2): 415-433. doi:10.1037/dev0000621

586. Luciano JV, Guallar JA, Aguado J, et al. Effectiveness of group acceptance and commitment therapy for fibromyalgia: A 6-month randomized controlled trial (EFFIGACT study). *Pain* 2014; **155**(4): 693-702. doi:10.1016/j.pain.2013.12.029

587. Luciano JV, Martínez N, Peñarrubia-María MT, et al. Effectiveness of a psychoeducational treatment program implemented in general practice for fibromyalgia patients: A randomized controlled trial. *Clin J Pain* 2011; **27**(5): 383-391. doi:10.1097/AJP.0b013e31820b131c

588. Luciano JV, Sabes-Figuera R, Cardeñosa E, et al. Cost-utility of a psychoeducational intervention in fibromyalgia patients compared with usual care: An economic evaluation alongside a 12-month randomized controlled trial. *Clin J Pain* 2013; **29**(8): 702-711. doi:10.1097/AJP.0b013e318270f99a

589. Ludwig M, Enzenhofer E, Schneider S, et al. Efficacy of a Carrageenan nasal spray in patients with common cold: A randomized controlled trial. *Respir Res* 2013; **14**(1). doi:10.1186/1465-9921-14-124

590. Lumley MA, Schubiner H, Lockhart NA, et al. Emotional awareness and expression therapy, cognitive behavioral therapy, and education for fibromyalgia: A cluster-randomized controlled trial. *Pain* 2017; **158**(12): 2354-2363. doi:10.1097/j.pain.0000000000001036

591. Lund C, Schneider M, Garman EC, et al. Task-sharing of psychological treatment for antenatal depression in Khayelitsha, South Africa: Effects on antenatal and postnatal outcomes in an individual randomised controlled trial. *Behav Res Ther* 2020; **130**. doi:10.1016/j.brat.2019.103466

592. Löbner M, Pabst A, Stein J, et al. Computerized cognitive behavior therapy for patients with mild to moderately severe depression in primary care: A pragmatic cluster randomized controlled trial (@ktiv). *Journal of Affective Disorders* 2018; **238**: 317-326. doi:10.1016/j.jad.2018.06.008

593. Löfholm CA, Eichas K, Sundell K. The Swedish Implementation of Multisystemic Therapy for Adolescents: Does Treatment Experience Predict Treatment Adherence? *J Clin Child Adolesc Psychol* 2014; **43**(4): 643-655. doi:10.1080/15374416.2014.883926

594. Lôo H, Malka R, Defiance R, et al. Tianeptine and amitriptyline: Controlled double-blind trial in depressed alcoholic patients. *Neuropsychobiology* 1988; **19**(2): 79-85. doi:10.1159/000118439

595. Ma WJ, Huang ZH, Huang BX, et al. Intensive low-glycaemic-load dietary intervention for the management of glycaemia and serum lipids among women with gestational diabetes: A randomized control trial. *Public Health Nutr* 2015; **18**(8): 1506-1513. doi:10.1017/S1368980014001992

596. Machielsen MWJ, Veltman DJ, van den Brink W, de Haan L. Comparing the effect of clozapine and risperidone on cue reactivity in male patients with schizophrenia and a cannabis use disorder: A randomized fMRI study. *Schizophr Res* 2018; **194**: 32-38. doi:10.1016/j.schres.2017.03.030

597. Machtei EE, Frankenthal S, Levi G, et al. Treatment of peri-implantitis using multiple applications of chlorhexidine chips: A double-blind, randomized multi-centre clinical trial. *J Clin Periodontol* 2012; **39**(12): 1198-1205. doi:10.1111/jcpe.12006

598. MacPhee AH, Kirby RL, Coolen AL, Smith C, MacLeod DA, Dupuis DJ. Wheelchair Skills Training Program: A Randomized Clinical Trial of Wheelchair Users Undergoing Initial Rehabilitation. *Arch Phys Med Rehabil* 2004; **85**(1): 41-50. doi:10.1016/S0003-9993(03)00364-2

599. Madden S, Miskovic-Wheatley J, Wallis A, et al. A randomized controlled trial of in-patient treatment for anorexia nervosa in medically unstable adolescents. *Psychol Med* 2015; **45**(2): 415-427. doi:10.1017/S0033291714001573

600. Madjd A, Taylor MA, Delavari A, Malekzadeh R, Macdonald IA, Farshchi HR. Beneficial effects of replacing diet beverages with water on type 2 diabetic obese women following a hypo-energetic diet: A randomized, 24-week clinical trial. *Diabetes Obes Metab* 2017; **19**(1): 125-132. doi:10.1111/dom.12793

601. Magill N, Rhind C, Hibbs R, et al. Two-year Follow-up of a Pragmatic Randomised Controlled Trial Examining the Effect of Adding a Carer's Skill Training Intervention in Inpatients with Anorexia Nervosa. *Eur Eating Disord Rev* 2016; **24**(2): 122-130. doi:10.1002/erv.2422

602. Maiorino MI, Bellastella G, Caputo M, et al. Effects of Mediterranean diet on sexual function in people with newly diagnosed type 2 diabetes: The MÈDITA trial. *J Diabetes Complications* 2016; **30**(8): 1519-1524. doi:10.1016/j.jdiacomp.2016.08.007

603. Malcolm R, Anton RF, Randall CL, Johnston A, Brady K, Thevos A. A Placebo‐Controlled Trial of Buspirone in Anxious Inpatient Alcoholics. *Alcohol Clin Exp Res* 1992; **16**(6): 1007-1013. doi:10.1111/j.1530-0277.1992.tb00691.x

604. Malmberg Gavelin H, Eskilsson T, Boraxbekk CJ, Josefsson M, Stigsdotter Neely A, Slunga Järvholm L. Rehabilitation for improved cognition in patients with stress-related exhaustion disorder: RECO–a randomized clinical trial. *Stress* 2018; **21**(4): 279-291. doi:10.1080/10253890.2018.1461833

605. Malti T, Ribeaud D, Eisner MP. The effectiveness of two universal preventive interventions in reducing children's externalizing behavior: A cluster randomized controlled trial. *J Clin Child Adolesc Psychol* 2011; **40**(5): 677-692. doi:10.1080/15374416.2011.597084

606. Maltz M, Koppe B, Jardim JJ, et al. Partial caries removal in deep caries lesions: a 5-year multicenter randomized controlled trial. *Clin Oral Invest* 2018; **22**(3): 1337-1343. doi:10.1007/s00784-017-2221-0

607. Man DWK, Poon WS, Lam C. The effectiveness of artificial intelligent 3-D virtual reality vocational problem-solving training in enhancing employment opportunities for people with traumatic brain injury. *Brain Inj* 2013; **27**(9): 1016-1025. doi:10.3109/02699052.2013.794969

608. Mannerkorpi K, Nordeman L, Cider Å, Jonsson G. Does moderate-to-high intensity Nordic walking improve functional capacity and pain in fibromyalgia? A prospective randomized controlled trial. *Arthritis Res Ther* 2010; **12**(5). doi:10.1186/ar3159

609. Mar PL, Raj V, Black BK, et al. Acute hemodynamic effects of a selective serotonin reuptake inhibitor in postural tachycardia syndrome: A randomized, crossover trial. *J Psychopharmacol* 2014; **28**(2): 155-161. doi:10.1177/0269881113512911

610. Marchand GH, Myhre K, Leivseth G, et al. Change in pain, disability and influence of fear-avoidance in a work-focused intervention on neck and back pain: A randomized controlled trial. *BMC Musculoskelet Disord* 2015; **16**(1). doi:10.1186/s12891-015-0553-y

611. Marhold C, Linton SJ, Melin L. A cognitive-behavioral return-to-work program: Effects on pain patients with a history of long-term versus short-term sick leave. *Pain* 2001; **91**(1-2): 155-163. doi:10.1016/S0304-3959(00)00431-0

612. Marks MN, Siddle K, Warwick C. Can we prevent postnatal depression? A randomized controlled trial to assess the effect of continuity of midwifery care on rates of postnatal depression in high-risk women. *J Matern-Fetal Neonatal Med* 2003; **13**(2): 119-127. doi:10.1080/jmf.13.2.119.127

613. Martens MP, Arterberry BJ, Takamatsu SK, Masters J, Dude K. The efficacy of a personalized feedback-only intervention for at-risk college gamblers. *J Consult Clin Psychol* 2015; **83**(3): 494-499. doi:10.1037/a0038843

614. Martignon S, Ekstrand KR, Ellwood R. Efficacy of sealing proximal early active lesions: An 18-month clinical study evaluated by conventional and subtraction radiography. *Caries Res* 2006; **40**(5): 382-388. doi:10.1159/000094282

615. Martignon S, Ekstrand KR, Gomez J, Lara JS, Cortes A. Infiltrating/sealing proximal caries lesions: A 3-year randomized clinical trial. *J Dent Res* 2012; **91**(3): 288-292. doi:10.1177/0022034511435328

616. Martin MHT, Nielsen MBD, Pedersen J, Rugulies R. Stability of return to work after a coordinated and tailored intervention for sickness absence compensation beneficiaries with mental health problems: Results of a two-year follow-up study. *Disabil Rehabil* 2015; **37**(22): 2107-2113. doi:10.3109/09638288.2014.1001524

617. Martinez Jr CR, Forgatch MS. Preventing problems with boys' noncompliance: Effects of a parent training intervention for divorcing mothers. *J Consult Clin Psychol* 2001; **69**(3): 416-428. doi:10.1037/0022-006X.69.3.416

618. Martínez MP, Miró E, Sánchez AI, et al. Cognitive-behavioral therapy for insomnia and sleep hygiene in fibromyalgia: A randomized controlled trial. *J Behav Med* 2014; **37**(4): 683-697. doi:10.1007/s10865-013-9520-y

619. Masiero M, Lucchiari C, Mazzocco K, et al. E-cigarettes may support smokers with high smoking-related risk awareness to stop smoking in the short run: Preliminary results by randomized controlled trial. *Nicotine Tob Res* 2019; **21**(1): 119-126. doi:10.1093/ntr/nty047

620. Masson PC, Von Ranson KM, Wallace LM, Safer DL. A randomized wait-list controlled pilot study of dialectical behaviour therapy guided self-help for binge eating disorder. *Behav Res Ther* 2013; **51**(11): 723-728. doi:10.1016/j.brat.2013.08.001

621. Matsuda Y, Kito S, Igarashi Y, Shigeta M. Efficacy and Safety of Deep Transcranial Magnetic Stimulation in Office Workers with Treatment-Resistant Depression: A Randomized, Double-Blind, Sham-Controlled Trial. *Neuropsychobiology* 2020; **79**(3): 208-213. doi:10.1159/000505405

622. Mayer-Davis EJ, D'Antonio AM, Smith SM, et al. Pounds off with empowerment (POWER): A clinical trial of weight management strategies for black and white adults with diabetes who live in medically underserved rural communities. *Am J Public Health* 2004; **94**(10): 1736-1742. doi:10.2105/AJPH.94.10.1736

623. Mazzeo SE, Lydecker J, Harney M, et al. Development and preliminary effectiveness of an innovative treatment for binge eating in racially diverse adolescent girls. *Eating Behav* 2016; **22**: 199-205. doi:10.1016/j.eatbeh.2016.06.014

624. McCabe K, Yeh M. Parent-child interaction therapy for Mexican Americans: A randomized clinical trial. *J Clin Child Adolesc Psychol* 2009; **38**(5): 753-759. doi:10.1080/15374410903103544

625. McCabe K, Yeh M, Lau A, Argote CB. Parent-Child Interaction Therapy for Mexican Americans: Results of a Pilot Randomized Clinical Trial at Follow-up. *Behav Ther* 2012; **43**(3): 606-618. doi:10.1016/j.beth.2011.11.001

626. McCarthy FP, Murphy A, Khashan AS, et al. Day care compared with inpatient management of nausea and vomiting of pregnancy: A randomized controlled trial. *Obstet Gynecol* 2014; **124**(4): 743-748. doi:10.1097/AOG.0000000000000449

627. McCauley E, Berk MS, Asarnow JR, et al. Efficacy of dialectical behavior therapy for adolescents at high risk for suicide a randomized clinical trial. *JAMA Psychiatry* 2018; **75**(8): 777-785. doi:10.1001/jamapsychiatry.2018.1109

628. McCrae CS, Williams J, Roditi D, et al. Cognitive behavioral treatments for insomnia and pain in adults with comorbid chronic insomnia and fibromyalgia: clinical outcomes from the SPIN randomized controlled trial. *Sleep* 2019; **42**(3). doi:10.1093/sleep/zsy234

629. McDowell D, Nunes EV, Seracini AM, et al. Desipramine treatment of cocaine-dependent patients with depression: A placebo-controlled trial. *Drug Alcohol Depend* 2005; **80**(2): 209-221. doi:10.1016/j.drugalcdep.2005.03.026

630. McElroy SL, Casuto LS, Nelson EB, et al. Placebo-controlled trial of sertraline in the treatment of binge eating disorder. *AM J PSYCHIATRY* 2000; **157**(6): 1004-1006. doi:10.1176/appi.ajp.157.6.1004

631. McElroy SL, Hudson JI, Malhotra S, Welge JA, Nelson EB, Keck Jr PE. Citalopram in the treatment of binge-eating disorder: A placebo-controlled trial. *J CLIN PSYCHIATRY* 2003; **64**(7): 807-813. doi:10.4088/JCP.v64n0711

632. McEvoy PM, Targowski K, McGrath D, et al. Efficacy of a brief group intervention for carers of individuals with eating disorders: A randomized control trial. *Int J Eating Disord* 2019; **52**(9): 987-995. doi:10.1002/eat.23121

633. McGrath PJ, Nunes EV, Stewart JW, et al. Imipramine treatment of alcoholics with primary depression: A placebo-controlled clinical trial. *Arch Gen Psychiatry* 1996; **53**(3): 232-240. doi:10.1001/archpsyc.1996.01830030054009

634. McGregor G, Sandhu H, Bruce J, et al. Clinical effectiveness of an online supervised group physical and mental health rehabilitation programme for adults with post-covid-19 condition (REGAIN study): Multicentre randomised controlled trial. *BMJ* 2024. doi:10.1136/bmj-2023-076506

635. McIntosh VVW, Jordan J, Carter FA, et al. Three psychotherapies for anorexia nervosa: A randomized, controlled trial. *AM J PSYCHIATRY* 2005; **162**(4): 741-747. doi:10.1176/appi.ajp.162.4.741

636. McIntyre RS, Phan L, Kwan ATH, et al. Vortioxetine for the treatment of post-COVID-19 condition: a randomized controlled trial. *Brain* 2024; **147**(3): 849-857. doi:10.1093/brain/awad377

637. McLean CP, Foa EB, Dondanville KA, et al. The effects of web-prolonged exposure among military personnel and veterans with posttraumatic stress disorder. *Psychol Trauma Theory Res Pract Policy* 2020. doi:10.1037/tra0000978

638. McMain SF, Guimond T, Barnhart R, Habinski L, Streiner DL. A randomized trial of brief dialectical behaviour therapy skills training in suicidal patients suffering from borderline disorder. *Acta Psychiatr Scand* 2017; **135**(2): 138-148. doi:10.1111/acps.12664

639. McMain SF, Guimond T, Streiner DL, Cardish RJ, Links PS. Dialectical behavior therapy compared with general psychiatric management for borderline personality disorder: Clinical outcomes and functioning over a 2-year follow-up. *AM J PSYCHIATRY* 2012; **169**(6): 650-661. doi:10.1176/appi.ajp.2012.11091416

640. McMain SF, Links PS, Gnam WH, et al. A randomized trial of dialectical behavior therapy versus general psychiatric management for borderline personality disorder. *AM J PSYCHIATRY* 2009; **166**(12): 1365-1374. doi:10.1176/appi.ajp.2009.09010039

641. McManama O'Brien KH, Sellers CM, Battalen AW, et al. Feasibility, acceptability, and preliminary effects of a brief alcohol intervention for suicidal adolescents in inpatient psychiatric treatment. *J Subst Abuse Treat* 2018; **94**: 105-112. doi:10.1016/j.jsat.2018.08.013

642. McNarry MA, Berg RMG, Shelley J, et al. Inspiratory muscle training enhances recovery post-COVID-19: a randomised controlled trial. *Eur Respir J* 2022; **60**(4). doi:10.1183/13993003.03101-2021

643. McParlin C, Carrick-Sen D, Steen IN, Robson SC. Hyperemesis in Pregnancy Study: A pilot randomised controlled trial of midwife-led outpatient care. *Eur J Obstet Gynecol Reprod Biol* 2016; **200**: 6-10. doi:10.1016/j.ejogrb.2016.02.016

644. McRae AL, Sonne SC, Brady KT, Durkalski V, Palesch Y. A Randomized, Placebo-controlled trial of Buspirone for the Treatment of Anxiety in Opioid-dependent Individuals. *Am J Addict* 2004; **13**(1): 53-63. doi:10.1080/10550490490265325

645. McRae-Clark AL, Carter RE, Killeen TK, Carpenter MJ, White KG, Brady KT. A placebo-controlled trial of atomoxetine in marijuana-dependent individuals with attention deficit hyperactivity disorder. *Am J Addict* 2010; **19**(6): 481-489. doi:10.1111/j.1521-0391.2010.00076.x

646. Mehlum L, Ramberg M, Tørmoen AJ, et al. Dialectical Behavior Therapy Compared with Enhanced Usual Care for Adolescents with Repeated Suicidal and Self-Harming Behavior: Outcomes over a One-Year Follow-Up. *J AM ACAD CHILD ADOLESC PSYCHIATRY* 2016; **55**(4): 295-300. doi:10.1016/j.jaac.2016.01.005

647. Mehlum L, Ramleth RK, Tørmoen AJ, et al. Long term effectiveness of dialectical behavior therapy versus enhanced usual care for adolescents with self-harming and suicidal behavior. *J Child Psychol Psychiatry Allied Discip* 2019; **60**(10): 1112-1122. doi:10.1111/jcpp.13077

648. Mehlum L, Tørmoen AJ, Ramberg M, et al. Dialectical behavior therapy for adolescents with repeated suicidal and self-harming behavior: A randomized trial. *J AM ACAD CHILD ADOLESC PSYCHIATRY* 2014; **53**(10): 1082-1091. doi:10.1016/j.jaac.2014.07.003

649. Mendelson T, Greenberg MT, Dariotis JK, Gould LF, Rhoades BL, Leaf PJ. Feasibility and preliminary outcomes of a school-based mindfulness intervention for urban youth. *J Abnorm Child Psychol* 2010; **38**(7): 985-994. doi:10.1007/s10802-010-9418-x

650. Meng K, Seekatz B, Roband H, Worringen U, Vogel H, Faller H. Intermediate and long-term effects of a standardized back school for inpatient orthopedic rehabilitation on illness knowledge and self-management behaviors: A randomized controlled trial. *Clin J Pain* 2011; **27**(3): 248-257. doi:10.1097/AJP.0b013e3181ffbfaf

651. Meyer-Lueckel H, Balbach A, Schikowsky C, Bitter K, Paris S. Pragmatic RCT on the Efficacy of Proximal Caries Infiltration. *J Dent Res* 2016; **95**(5): 531-536. doi:10.1177/0022034516629116

652. Meyer-Lueckel H, Bitter K, Paris S. Randomized controlled clinical trial on proximal caries infiltration: Three-year follow-up. *Caries Res* 2012; **46**(6): 544-548. doi:10.1159/000341807

653. Mhalla A, Baudic S, De Andrade DC, et al. Long-term maintenance of the analgesic effects of transcranial magnetic stimulation in fibromyalgia. *Pain* 2011; **152**(7): 1478-1485. doi:10.1016/j.pain.2011.01.034

654. Mijatovic, Yu Louie JC, Buso MEC, et al. Effects of a modestly lower carbohydrate diet in gestational diabetes: A randomized controlled trial. *Am J Clin Nutr* 2020; **112**(2): 284-292. doi:10.1093/ajcn/nqaa137

655. Miki K, Murakami M, Oka H, Onozawa K, Yoshida S, Osada K. Efficacy of mirtazapine for the treatment of fibromyalgia without concomitant depression: A randomized, double-blind, placebo-controlled phase IIa study in Japan. *Pain* 2016; **157**(9): 2089-2096. doi:10.1097/j.pain.0000000000000622

656. Milgrom J, Danaher BG, Gemmill AW, et al. Internet cognitive behavioral therapy for women with postnatal depression: A randomized controlled trial of MumMoodBooster. *J Med Internet Res* 2016; **18**(3). doi:10.2196/jmir.4993

657. Milgrom J, Danaher BG, Seeley JR, et al. Internet and Face-to-face Cognitive Behavioral Therapy for Postnatal Depression Compared with Treatment as Usual: Randomized Controlled Trial of MumMoodBooster. *J Med Internet Res* 2021; **23**(12). doi:10.2196/17185

658. Milgrom J, Holt C, Holt CJ, Ross J, Ericksen J, Gemmill AW. Feasibility study and pilot randomised trial of an antenatal depression treatment with infant follow-up. *Arch Women's Ment Health* 2015; **18**(5): 717-730. doi:10.1007/s00737-015-0512-5

659. Milgrom J, Holt CJ, Gemmill AW, et al. Treating postnatal depressive symptoms in primary care: A randomised controlled trial of GP management, with and without adjunctive counselling. *BMC Psychiatry* 2011; **11**. doi:10.1186/1471-244X-11-95

660. Miller WC, Best KL, Eng JJ, Routhier F. Influence of Peer-led Wheelchair Training on Wheelchair Skills and Participation in Older Adults: Clinical Outcomes of a Randomized Controlled Feasibility Trial. *Arch Phys Med Rehabil* 2019; **100**(6): 1023-1031. doi:10.1016/j.apmr.2018.10.018

661. Mires G, Williams F, Howie P. Randomised controlled trial of cardiotocography versus Doppler auscultation of fetal heart at admission in labour in low risk obstetric population. *Br Med J* 2001; **322**(7300): 1457-1460. doi:10.1136/bmj.322.7300.1457

662. Mitchell JE, Crosby RD, Wonderlich SA, et al. A randomized trial comparing the efficacy of cognitive-behavioral therapy for bulimia nervosa delivered via telemedicine versus face-to-face. *Behav Res Ther* 2008; **46**(5): 581-592. doi:10.1016/j.brat.2008.02.004

663. Mitchell JE, Fletcher L, Hanson K, et al. The relative efficacy of fluoxetine and manual-based self-help in the treatment of outpatients with bulimia nervosa. *J Clin Psychopharmacol* 2001; **21**(3): 298-304. doi:10.1097/00004714-200106000-00008

664. Mitchell-Jones N, Farren JA, Tobias A, Bourne T, Bottomley C. Ambulatory versus inpatient management of severe nausea and vomiting of pregnancy: A randomised control trial with patient preference arm. *BMJ Open* 2017; **7**(12). doi:10.1136/bmjopen-2017-017566

665. Mitri J, Tomah S, Mottalib A, et al. Effect of dairy consumption and its fat content on glycemic control and cardiovascular disease risk factors in patients with type 2 diabetes: A randomized controlled study. *Am J Clin Nutr* 2020; **112**(2): 293-302. doi:10.1093/ajcn/nqaa138

666. Moak DH, Anton RF, Latham PK, Voronin KE, Waid RL, Durazo-Arvizu R. Sertraline and Cognitive Behavioral Therapy for Depressed Alcoholics: Results of a Placebo-Controlled Trial. *J Clin Psychopharmacol* 2003; **23**(6): 553-562. doi:10.1097/01.jcp.0000095346.32154.41

667. Molander A, Warfvinge J, Reit C, Kvist T. Clinical and Radiographic Evaluation of One- and Two-visit Endodontic Treatment of Asymptomatic Necrotic Teeth with Apical Periodontitis: A Randomized Clinical Trial. *J Endod* 2007; **33**(10): 1145-1148. doi:10.1016/j.joen.2007.07.005

668. Moll LT, Jensen OK, Schiøttz-Christensen B, et al. Return to Work in Employees on Sick Leave due to Neck or Shoulder Pain: A Randomized Clinical Trial Comparing Multidisciplinary and Brief Intervention with One-Year Register-Based Follow-Up. *J Occup Rehabil* 2018; **28**(2): 346-356. doi:10.1007/s10926-017-9727-9

669. Momtazmanesh S, Ansari S, Izadi Z, et al. Effect of famotidine on cognitive and behavioral dysfunctions induced in post-COVID-19 infection: A randomized, double-blind, and placebo-controlled study. *J Psychosom Res* 2023; **172**. doi:10.1016/j.jpsychores.2023.111389

670. Montero-Marín J, Araya R, Pérez-Yus MC, et al. An internet-based intervention for depression in primary care in Spain: A randomized controlled trial. *J Med Internet Res* 2016; **18**(8). doi:10.2196/jmir.5695

671. Monticone M, Ambrosini E, Rocca B, Cazzaniga D, Liquori V, Foti C. Group-based task-oriented exercises aimed at managing kinesiophobia improved disability in chronic low back pain. *Eur J Pain* 2016; **20**(4): 541-551. doi:10.1002/ejp.756

672. Monticone M, Ambrosini E, Rocca B, et al. Group-based multimodal exercises integrated with cognitive-behavioural therapy improve disability, pain and quality of life of subjects with chronic neck pain: A randomized controlled trial with one-year follow-up. *Clin Rehabil* 2017; **31**(6): 742-752. doi:10.1177/0269215516651979

673. Monticone M, Ferrante S, Rocca B, Baiardi P, Farra FD, Foti C. Effect of a long-lasting multidisciplinary program on disability and fear-avoidance behaviors in patients with chronic low back pain: Results of a randomized controlled trial. *Clin J Pain* 2013; **29**(11): 929-938. doi:10.1097/AJP.0b013e31827fef7e

674. Moon J, Kim DY, Lee WJ, et al. Efficacy of Propranolol, Bisoprolol, and Pyridostigmine for Postural Tachycardia Syndrome: a Randomized Clinical Trial. *Neurotherapeutics* 2018; **15**(3): 785-795. doi:10.1007/s13311-018-0612-9

675. Moreno-Castilla C, Hernandez M, Bergua M, et al. Low-Carbohydrate diet for the treatment of gestational diabetes mellitus: A randomized controlled trial. *Diabetes Care* 2013; **36**(8): 2233-2238. doi:10.2337/dc12-2714

676. Morin A, Leonard G, Gougeon V, et al. Efficacy of transcranial direct-current stimulation in women with provoked vestibulodynia. *Am J Obstet Gynecol* 2017; **216**(6): 584 e581-584 e511. doi:10.1016/j.ajog.2017.02.049

677. Morin M, Dumoulin C, Bergeron S, et al. Multimodal physical therapy versus topical lidocaine for provoked vestibulodynia: a multicenter, randomized trial. *AM J OBSTET GYNECOL* 2021; **224**(2): 189.e181-189.e112. doi:10.1016/j.ajog.2020.08.038

678. Mountain AD, Kirby RL, Smith C, Eskes G, Thompson K. Powered wheelchair skills training for persons with stroke: A randomized controlled trial. *Am J Phys Med Rehabil* 2014; **93**(12): 1031-1043. doi:10.1097/PHM.0000000000000229

679. Mu Y, Liu X, Li Q, et al. Efficacy and safety of pregabalin for painful diabetic peripheral neuropathy in a population of Chinese patients: A randomized placebo-controlled trial. *J Diabetes* 2018; **10**(3): 256-265. doi:10.1111/1753-0407.12585

680. Muhonen LH, Lahti J, Sinclair D, Lönnqvist J, Alho H. Treatment of alcohol dependence in patients with co-morbid major depressive disorder - Predictors for the outcomes with memantine and escitalopram medication. *Subst Abuse Treat Prev Policy* 2008; **3**. doi:10.1186/1747-597X-3-20

681. Muhonen LH, Lönnqvist J, Juva K, Alho H. Double-blind, randomized comparison of memantine and escitalopram for the treatment of major depressive disorder comorbid with alcohol dependence. *J CLIN PSYCHIATRY* 2008; **69**(3): 392-399. doi:10.4088/JCP.v69n0308

682. Mulcahy R, Reay RE, Wilkinson RB, Owen C. A randomised control trial for the effectiveness of group interpersonal psychotherapy for postnatal depression. *Arch Women's Ment Health* 2010; **13**(2): 125-139. doi:10.1007/s00737-009-0101-6

683. Murakami M, Osada K, Mizuno H, Ochiai T, Alev L, Nishioka K. A randomized, double-blind, placebo-controlled phase III trial of duloxetine in Japanese fibromyalgia patients. *Arthritis Res Ther* 2015; **17**(1). doi:10.1186/s13075-015-0718-y

684. Muratori P, Bertacchi I, Giuli C, et al. First Adaptation of Coping Power Program as a Classroom-Based Prevention Intervention on Aggressive Behaviors Among Elementary School Children. *Prev Sci* 2015; **16**(3): 432-439. doi:10.1007/s11121-014-0501-3

685. Muratori P, Bertacchi I, Giuli C, Nocentini A, Ruglioni L, Lochman JE. Coping Power Adapted as Universal Prevention Program: Mid Term Effects on Children’s Behavioral Difficulties and Academic Grades. *J Prim Prev* 2016; **37**(4): 389-401. doi:10.1007/s10935-016-0435-6

686. Murina F, Bianco V, Radici G, Felice R, Di Martino M, Nicolini U. Transcutaneous electrical nerve stimulation to treat vestibulodynia: A randomised controlled trial. *BJOG Int J Obstet Gynaecol* 2008; **115**(9): 1165-1170. doi:10.1111/j.1471-0528.2008.01803.x

687. Murina F, Felice R, Di Francesco S, Oneda S. Vaginal diazepam plus transcutaneous electrical nerve stimulation to treat vestibulodynia: A randomized controlled trial. *Eur J Obstet Gynecol Reprod Biol* 2018; **228**: 148-153. doi:10.1016/j.ejogrb.2018.06.026

688. Murina F, Graziottin A, Felice R, Radici G, Tognocchi C. Vestibulodynia: Synergy between palmitoylethanolamide + transpolydatin and transcutaneous electrical nerve stimulation. *J Lower Genital Tract Dis* 2013; **17**(2): 111-116. doi:10.1097/LGT.0b013e3182652316

689. Murphy TK, Parker-Athill EC, Lewin AB, Storch EA, Mutch PJ. Cefdinir for recent-onset pediatric neuropsychiatric disorders: A pilot randomized trial. *J Child Adolesc Psychopharmacol* 2015; **25**(1): 57-64. doi:10.1089/cap.2014.0010

690. Murray DW, Rabiner DL, Kuhn L, Pan Y, Sabet RF. Investigating teacher and student effects of the Incredible Years Classroom Management Program in early elementary school. *J Sch Psychol* 2018; **67**: 119-133. doi:10.1016/j.jsp.2017.10.004

691. Murray JA, Terry DJ, Vance JC, Battistutta D, Connolly Y. Effects of a program of intervention on parental distress following infant death. *Death Stud* 2000; **24**(4): 275-305. doi:10.1080/074811800200469

692. Musekamp G, Gerlich C, Ehlebracht-Knig I, et al. Evaluation of a self-management patient education programme for fibromyalgia - results of a cluster-RCT in inpatient rehabilitation. *Health Educ Res* 2019; **34**(2): 209-222. doi:10.1093/her/cyy055

693. Myhre K, Marchand GH, Leivseth G, et al. The effect of work-focused rehabilitation among patients with neck and back pain: A randomized controlled trial. *Spine* 2014; **39**(24): 1999-2006. doi:10.1097/BRS.0000000000000610

694. Na PJ, Ralevski E, Jegede O, Wolfgang A, Petrakis IL. Depression and/or PTSD Comorbidity Affects Response to Antidepressants in Those With Alcohol Use Disorder. *Front Psychiatry* 2022; **12**. doi:10.3389/fpsyt.2021.768318

695. Navas-Otero A, Calvache-Mateo A, Calles-Plata I, et al. A lifestyle adjustments program in long COVID-19 improves symptomatic severity and quality of life. A randomized control trial. *Patient Educ Couns* 2024; **122**. doi:10.1016/j.pec.2024.108180

696. Naylor KE, McCloskey EV, Jacques RM, et al. Clinical utility of bone turnover markers in monitoring the withdrawal of treatment with oral bisphosphonates in postmenopausal osteoporosis. *Osteoporosis International* 2019; **30**(4): 917-922. doi:10.1007/s00198-018-04823-5

697. Neighbors C, Rodriguez LM, Rinker DV, et al. Efficacy of personalized normative feedback as a brief intervention for college student gambling: A randomized controlled trial. *J Consult Clin Psychol* 2015; **83**(3): 500-511. doi:10.1037/a0039125

698. Nejtek VA, Avila M, Chen LA, et al. Do atypical antipsychotics effectively treat co-occurring bipolar disorder and stimulant dependence? A randomized, double-blind trial. *J CLIN PSYCHIATRY* 2008; **69**(8): 1257-1266. doi:10.4088/JCP.v69n0808

699. Nelson-Piercy C, Fayers P, De Swiet M. Randomised, double-blind, placebo-controlled trial of corticosteroids for the treatment of hyperemesis gravidarum. *Br J Obstet Gynaecol* 2001; **108**(1): 9-15. doi:10.1016/S0306-5456(00)00017-6

700. Neri I, Allais G, Schiapparelli P, Blasi I, Benedetto C, Facchinetti F. Acupuncture versus pharmacological approach to reduce Hyperemesis gravidarum discomfort. *Minerva Ginecol* 2005; **57**(4): 471-475. doi:N

701. Netterstrøm B, Bech P. Effect of a multidisciplinary stress treatment programme on the return to work rate for persons with work-related stress. A non-randomized controlled study from a stress clinic. *BMC Public Health* 2010; **10**. doi:10.1186/1471-2458-10-658

702. Netterstrøm B, Friebel L, Ladegaard Y. Effects of a multidisciplinary stress treatment programme on patient return to work rate and symptom reduction: Results from a randomised, wait-list controlled trial. *Psychother Psychosom* 2013; **82**(3): 177-186. doi:10.1159/000346369

703. Nicholas MK, Asghari A, Blyth FM, et al. Self-management intervention for chronic pain in older adults: A randomised controlled trial. *Pain* 2013; **154**(6): 824-835. doi:10.1016/j.pain.2013.02.009

704. Nieuwenhuijsen K, Schoutens AMC, Frings-Dresen MHW, Sluiter JK. Evaluation of a randomized controlled trial on the effect on return to work with coaching combined with light therapy and pulsed electromagnetic field therapy for workers with work-related chronic stress. *BMC Public Health* 2017; **17**(1). doi:10.1186/s12889-017-4720-y

705. Nik TH, Shahsavari N, Ghadirian H, Ostad SN. Acetaminophen versus liquefied ibuprofen for control of pain during separation in orthodontic patients: A randomized triple blinded clinical trial. *Acta Med Iran* 2016; **54**(7): 418-421. doi:N

706. Nilsson MKL, Zachrisson O, Gottfries CG, et al. A randomised controlled trial of the monoaminergic stabiliser (-)-OSU6162 in treatment of myalgic encephalomyelitis/chronic fatigue syndrome. *Acta Neuropsychiatr* 2018; **30**(3): 148-157. doi:10.1017/neu.2017.35

707. Nishi D, Su KP, Usuda K, et al. Plasma estradiol levels and antidepressant effects of omega-3 fatty acids in pregnant women. *Brain Behav Immun* 2020; **85**: 29-34. doi:10.1016/j.bbi.2019.02.014

708. Nishi D, Su KP, Usuda K, et al. The efficacy of omega-3 fatty acids for depressive symptoms among pregnant women in Japan and Taiwan: A randomized, double-blind, placebo-controlled trial (SYNCHRO; NCT01948596). *Psychother Psychosom* 2019; **88**(2): 122-124. doi:10.1159/000495296

709. Nix RL, Bierman KL, Heinrichs BS, Gest SD, Welsh JA, Domitrovich CE. The randomized controlled trial of Head Start REDI: Sustained effects on developmental trajectories of social-emotional functioning. *J Consult Clin Psychol* 2016; **84**(4): 310-322. doi:10.1037/a0039937

710. Noggle JJ, Steiner NJ, Minami T, Khalsa SBS. Benefits of yoga for psychosocial well-being in a us high school curriculum: A preliminary randomized controlled trial. *J Dev Behav Pediatr* 2012; **33**(3): 193-201. doi:10.1097/DBP.0b013e31824afdc4

711. Noordik E, van der Klink JJ, Geskus RB, de Boer MR, van Dijk FJH, Nieuwenhuijsen K. Effectiveness of an exposure-based return-to-work program for workers on sick leave due to common mental disorders: A cluster-randomized controlled trial. *Scand J Work Environ Health* 2013; **39**(2): 144-154. doi:10.5271/sjweh.3320

712. Notzon DP, Mariani JJ, Pavlicova M, et al. Mixed-amphetamine salts increase abstinence from marijuana in patients with co-occurring attention-deficit/hyperactivity disorder and cocaine dependence. *Am J Addict* 2016; **25**(8): 666-672. doi:10.1111/ajad.12467

713. Novak M, Mihić J, Bašić J, Nix RL. PATHS in Croatia: A school-based randomised-controlled trial of a social and emotional learning curriculum. *Int J Psychol* 2017; **52**(2): 87-95. doi:10.1002/ijop.12262

714. Nunes EV, McGrath PJ, Quitkin FM, et al. Imipramine treatment of cocaine abuse: possible boundaries of efficacy. *Drug Alcohol Depend* 1995; **39**(3): 185-195. doi:10.1016/0376-8716(95)01161-6

715. Nunes EV, McGrath PJ, Quitkin FM, et al. Imipramine treatment of alcoholism with comorbid depression. *AM J PSYCHIATRY* 1993; **150**(6): 963-965. doi:10.1176/ajp.150.6.963

716. Nunes EV, Quitkin FM, Donovan SJ, et al. Imipramine treatment of opiate-dependent patients with depressive disorders: A placebo-controlled trial. *Arch Gen Psychiatry* 1998; **55**(2): 153-160. doi:10.1001/archpsyc.55.2.153

717. Nygaard AS, Rydningen MB, Stedenfeldt M, et al. Group-based multimodal physical therapy in women with chronic pelvic pain: A randomized controlled trial. *Acta Obstet Gynecol Scand* 2020; **99**(10): 1320-1329. doi:10.1111/aogs.13896

718. Nyirjesy P, Sobel JD, Weitz MV, Leaman DJ, Small MJ, Gelone SP. Cromolyn cream for recalcitrant idiopathic vulvar vestibulitis: Results of a placebo controlled study. *Sex Transm Infect* 2001; **77**(1): 53-57. doi:10.1136/sti.77.1.53

719. Nyman-Carlsson E, Norring C, Engström I, et al. Individual cognitive behavioral therapy and combined family/individual therapy for young adults with Anorexia nervosa: A randomized controlled trial. *Psychother Res* 2020; **30**(8): 1011-1025. doi:10.1080/10503307.2019.1686190

720. Nystuen P, Hagen KB. Solution-focused intervention for sick listed employees with psychological problems or muscle skeletal pain: A randomised controlled trial [ISRCTN39140363]. *BMC Public Health* 2006; **6**. doi:10.1186/1471-2458-6-69

721. O'Brien B, Knight-West O, Walker N, Parag V, Bullen C. E-cigarettes versus NRT for smoking reduction or cessation in people with mental illness: Secondary analysis of data from the ASCEND trial Dr Maciej L. Goniewicz. *Tob Induced Dis* 2015; **13**(1): 5. doi:10.1186/s12971-015-0030-2

722. O'Hara MW, Pearlstein T, Stuart S, Long JD, Mills JA, Zlotnick C. A placebo controlled treatment trial of sertraline and interpersonal psychotherapy for postpartum depression. *Journal of Affective Disorders* 2019; **245**: 524-532. doi:10.1016/j.jad.2018.10.361

723. O'Hara MW, Scott S, Gorman Laura L, Amy W. Efficacy of interpersonal psychotherapy for postpartum depression. *Arch Gen Psychiatry* 2000; **57**(11): 1039-1045. doi:10.1001/archpsyc.57.11.1039

724. O'Mahen HA, Richards DA, Woodford J, et al. Netmums: A phase II randomized controlled trial of a guided Internet behavioural activation treatment for postpartum depression. *Psychol Med* 2014; **44**(8): 1675-1689. doi:10.1017/S0033291713002092

725. O'Mahen HA, Woodford J, McGinley J, et al. Internet-based behavioral activation-Treatment for postnatal depression (Netmums): A randomized controlled trial. *Journal of Affective Disorders* 2013; **150**(3): 814-822. doi:10.1016/j.jad.2013.03.005

726. O'Malley SS, Sinha R, Grilo CM, et al. Naltrexone and cognitive behavioral coping skills therapy for the treatment of alcohol drinking and eating disorder features in alcohol-dependent women: A randomized controlled trial. *Alcohol Clin Exp Res* 2007; **31**(4): 625-634. doi:10.1111/j.1530-0277.2007.00347.x

727. O'Neil PM, Miller-Kovach K, Tuerk PW, et al. Randomized controlled trial of a nationally available weight control program tailored for adults with type 2 diabetes. *Obesity* 2016; **24**(11): 2269-2277. doi:10.1002/oby.21616

728. Ogonowska-Slodownik A, Labecka MK, Maciejewska-Skrendo A, et al. Effect of Water-Based vs. Land-Based Exercise Intervention (postCOVIDkids) on Exercise Capacity, Fatigue, and Quality of Life in Children with Post COVID-19 Condition: A Randomized Controlled Trial. *J Clin Med* 2023; **12**(19). doi:10.3390/jcm12196244

729. Ohta H, Oka H, Usui C, Ohkura M, Suzuki M, Nishioka K. A randomized, double-blind, multicenter, placebo-controlled phase III trial to evaluate the efficacy and safety of pregabalin in Japanese patients with fibromyalgia. *Arthritis Res Ther* 2012; **14**(5). doi:10.1186/ar4056

730. Okan F, Okan S, Duran Yücesoy F. Evaluating the Efficiency of Breathing Exercises via Telemedicine in Post-Covid-19 Patients: Randomized Controlled Study. *Clin Nurs Res* 2022; **31**(5): 771-781. doi:10.1177/10547738221097241

731. Ólason M, Andrason RH, Jónsdóttir IH, Kristbergsdóttir H, Jensen MP. Cognitive Behavioral Therapy for Depression and Anxiety in an Interdisciplinary Rehabilitation Program for Chronic Pain: a Randomized Controlled Trial with a 3-Year Follow-up. *Int J Behav Med* 2018; **25**(1): 55-66. doi:10.1007/s12529-017-9690-z

732. Omar MA, Abdelshafy M, Ahmed MY, Rezk AG, Taha AM, Hussein HM. Endoscopic Papillary Large Balloon Dilation Versus Endoscopic Sphincterotomy for Retrieval of Large Choledocholithiasis: A Prospective Randomized Trial. *J Laparoendosc Adv Surg Techn* 2017; **27**(7): 704-709. doi:10.1089/lap.2016.0601

733. Orhan AI, Oz FT, Orhan K. Pulp exposure occurrence and outcomes after 1- or 2-visit indirect pulp therapy vs complete caries removal in primary and permanent molars. *Pediatr Dent* 2010; **32**(4): 347-355. doi:N

734. Oslin DW. Treatment of late-life depression complicated by alcohol dependence. *Am J Geriatr Psychiatry* 2005; **13**(6): 491-500. doi:10.1097/00019442-200506000-00008

735. Ougrin D, Boege I, Stahl D, Banarsee R, Taylor E. Randomised controlled trial of therapeutic assessment versus usual assessment in adolescents with self-harm: 2-year follow-up. *Arch Dis Child* 2013; **98**(10): 772-776. doi:10.1136/archdischild-2012-303200

736. Ougrin D, Zundel T, Kyriakopoulos M, Banarsee R, Stahl D, Taylor E. Adolescents with suicidal and nonsuicidal self-harm: Clinical characteristics and response to therapeutic assessment. *Psychol Assess* 2012; **24**(1): 11-20. doi:10.1037/a0025043

737. Ougrin D, Zundel T, Ng A, Banarsee R, Bottle A, Taylor E. Trial of Therapeutic Assessment in London: Randomised controlled trial of Therapeutic Assessment versus standard psychosocial assessment in adolescents presenting with self-harm. *Arch Dis Child* 2011; **96**(2): 148-153. doi:10.1136/adc.2010.188755

738. Overbeek G, van Aar J, de Castro BO, et al. Longer-Term Outcomes of the Incredible Years Parenting Intervention. *Prev Sci* 2021; **22**(4): 419-431. doi:10.1007/s11121-020-01176-6

739. Ownsworth T, Fleming J, Shum D, Kuipers P, Strong J. Comparison of individual, group and combined intervention formats in a randomized controlled trial for facilitating goal attainment and improving psychosocial function following acquired brain injury. *J Rehabil Med* 2008; **40**(2): 81-88. doi:10.2340/16501977-0124

740. Pachankis JE, Soulliard ZA, Layland EK, et al. Guided LGBTQ-affirmative internet cognitive-behavioral therapy for sexual minority youth's mental health: A randomized controlled trial of a minority stress treatment approach. *Behav Res Ther* 2023; **169**. doi:10.1016/j.brat.2023.104403

741. Palau P, Domínguez E, Gonzalez C, et al. Effect of a home-based inspiratory muscle training programme on functional capacity in postdischarged patients with long COVID: The InsCOVID trial. *BMJ Open Respir Res* 2022; **9**(1). doi:10.1136/bmjresp-2022-001439

742. Palmer ML, Keown LJ, Sanders MR, Henderson M. Enhancing Outcomes of Low-Intensity Parenting Groups Through Sufficient Exemplar Training: A Randomized Control Trial. *Child Psychiatry Hum Dev* 2019; **50**(3): 384-399. doi:10.1007/s10578-018-0847-z

743. Pannebakker FD, van Genugten L, Diekstra RFW, et al. A Social Gradient in the Effects of the Skills for Life Program on Self-Efficacy and Mental Wellbeing of Adolescent Students. *J Sch Health* 2019; **89**(7): 587-595. doi:10.1111/josh.12779

744. Paolucci T, Baldari C, Di Franco M, et al. A New Rehabilitation Tool in Fibromyalgia: The Effects of Perceptive Rehabilitation on Pain and Function in a Clinical Randomized Controlled Trial. *Evid-Based Complement Altern Med* 2016; **2016**: 7574589. doi:10.1155/2016/7574589

745. Paredes-Vieyra J, Enriquez FJJ. Success rate of single-versus two-visit root canal treatment of teeth with apical periodontitis: A randomized controlled trial. *J Endod* 2012; **38**(9): 1164-1169. doi:10.1016/j.joen.2012.05.021

746. Paris S, Bitter K, Krois J, Meyer-Lueckel H. Seven-year-efficacy of proximal caries infiltration – Randomized clinical trial. *J Dent* 2020; **93**. doi:10.1016/j.jdent.2020.103277

747. Paris S, Hopfenmuller W, Meyer-Lueckel H. Resin infiltration of caries lesions: An efficacy randomized trial. *J Dent Res* 2010; **89**(8): 823-826. doi:10.1177/0022034510369289

748. Parra-Cardona JR, Bybee D, Sullivan CM, et al. Examining the Impact of Differential Cultural Adaptation with Latina/o Immigrants Exposed to Adapted Parent Training Interventions. *J Consult Clin Psychol* 2017; **85**(1): 58-71. doi:10.1037/ccp0000160

749. Parry BL, Meliska CJ, Lopez AM, et al. Early versus late wake therapy improves mood more in antepartum versus postpartum depression by differentially altering melatonin-sleep timing disturbances. *Journal of Affective Disorders* 2019; **245**: 608-616. doi:10.1016/j.jad.2018.11.064

750. Patterson J, Barlow J, Mockford C, Klimes I, Pyper C, Stewart-Brown S. Improving mental health through parenting programmes: Block randomised controlled trial. *Arch Dis Child* 2002; **87**(6): 472-477. doi:10.1136/adc.87.6.472

751. Pauer L, Winkelmann A, Arsenault P, et al. An international, randomized, double-blind, placebo-controlled,phase III trial of pregabalin monotherapy in treatment of patients with fibromyalgia. *J Rheumatol* 2011; **38**(12): 2643-2652. doi:10.3899/jrheum.110569

752. Pearlstein T, Spurell E, Hohlstein LA, et al. A double-blind, placebo-controlled trial of fluvoxamine in binge eating disorder: A high placebo response. *Arch Women's Ment Health* 2003; **6**(2): 147-151. doi:10.1007/s00737-003-0172-8

753. Pedersen KKW, Langagergaard V, Jensen OK, Nielsen CV, Sørensen VN, Pedersen P. Two-Year Follow-Up on Return to Work in a Randomised Controlled Trial Comparing Brief and Multidisciplinary Intervention in Employees on Sick Leave Due to Low Back Pain. *J Occup Rehabil* 2022; **32**(4): 697-704. doi:10.1007/s10926-022-10030-1

754. Pedersen P, Nielsen CV, Jensen OK, Jensen C, Labriola M. Employment status five years after a randomised controlled trial comparing multidisciplinary and brief intervention in employees on sick leave due to low back pain. *Scand J Public Health* 2018; **46**(3): 383-388. doi:10.1177/1403494817722290

755. Pedersen P, Søgaard HJ, Labriola M, Nohr EA, Jensen C. Effectiveness of psychoeducation in reducing sickness absence and improving mental health in individuals at risk of having a mental disorder: A randomised controlled trial. *BMC Public Health* 2015; **15**(1). doi:10.1186/s12889-015-2087-5

756. Pelham WE, III, Dishion TJ, Tein JY, Shaw DS, Wilson MN. What Doesn’t Work for Whom? Exploring Heterogeneity in Responsiveness to the Family Check-Up in Early Childhood Using a Mixture Model Approach. *Prev Sci* 2017; **18**(8): 911-922. doi:10.1007/s11121-017-0805-1

757. Perez-Aranda A, Feliu-Soler A, Montero-Marin J, et al. A randomized controlled efficacy trial of mindfulness-based stress reduction compared with an active control group and usual care for fibromyalgia: The EUDAIMON study. *Pain* 2019; **160**(11): 2508-2523. doi:10.1097/j.pain.0000000000001655

758. Perkins R, Yorke S, Fancourt D. How group singing facilitates recovery from the symptoms of postnatal depression: A comparative qualitative study. *BMC Psychol* 2018; **6**(1). doi:10.1186/s40359-018-0253-0

759. Perrin EC, Sheldrick RC, McMenamy JM, Henson BS, Carter AS. Improving parenting skills for families of young children in pediatric settings: A randomized clinical trial. *JAMA Pediatr* 2014; **168**(1): 16-24. doi:10.1001/jamapediatrics.2013.2919

760. Peters MC, Hopkins AR, Jr., Yu Q. Resin infiltration: An effective adjunct strategy for managing high caries risk—A within-person randomized controlled clinical trial. *J Dent* 2018; **79**: 24-30. doi:10.1016/j.jdent.2018.09.005

761. Peters MC, Hopkins AR, Jr., Zhu L, Yu Q. Efficacy of Proximal Resin Infiltration on Caries Inhibition: Results from a 3-Year Randomized Controlled Clinical Trial. *J Dent Res* 2019; **98**(13): 1497-1502. doi:10.1177/0022034519876853

762. Petersen CD, Giraldi A, Lundvall L, Kristensen E. Botulinum toxin type A - A novel treatment for provoked vestibulodynia? Results from a randomized, placebo controlled, double blinded study. *J Sex Med* 2009; **6**(9): 2523-2537. doi:10.1111/j.1743-6109.2009.01378.x

763. Petersen KS, Clifton PM, Blanch N, Keogh JB. Effect of improving dietary quality on carotid intima media thickness in subjects with type 1 and type 2 diabetes: A 12-mo randomized controlled trial. *Am J Clin Nutr* 2015; **102**(4): 771-779. doi:10.3945/ajcn.115.112151

764. Petersen LB, Vaeth M, Wenzel A. Neurosensoric disturbances after surgical removal of the mandibular third molar based on either panoramic imaging or cone beam CT scanning: A randomized controlled trial (RCT). *Dentomaxillofac Radiol* 2015; **45**(2). doi:10.1259/dmfr.20150224

765. Peterson CB, Mitchell JE, Crow SJ, Crosby RD, Wonderlich SA. The efficacy of self-help group treatment and therapist-led group treatment for binge eating disorder. *AM J PSYCHIATRY* 2009; **166**(12): 1347-1354. doi:10.1176/appi.ajp.2009.09030345

766. Petrakis I, M. Carroll K, Nich C, Gordon L, Kosten T, Rounsaville B. Fluoxetine treatment of depressive disorders in methadone-maintained opioid addicts. *Drug Alcohol Depend* 1998; **50**(3): 221-226. doi:10.1016/S0376-8716(98)00032-5

767. Petrakis I, Ralevski E, Nich C, et al. Naltrexone and disulfiram in patients with alcohol dependence and current depression. *J Clin Psychopharmacol* 2007; **27**(2): 160-165. doi:10.1097/jcp.0b13e3180337fcb

768. Petrakis IL, Desai N, Gueorguieva R, et al. Prazosin for Veterans with Posttraumatic Stress Disorder and Comorbid Alcohol Dependence: A Clinical Trial. *Alcohol Clin Exp Res* 2016; **40**(1): 178-186. doi:10.1111/acer.12926

769. Petrakis IL, O'Malley S, Rounsaville B, et al. Naltrexone augmentation of neuroleptic treatment in alcohol abusing patients with schizophrenia. *Psychopharmacology* 2004; **172**(3): 291-297. doi:10.1007/s00213-003-1658-9

770. Petrakis IL, Poling J, Levinson C, et al. Naltrexone and Disulfiram in Patients with Alcohol Dependence and Comorbid Post-Traumatic Stress Disorder. *Biol Psychiatry* 2006; **60**(7): 777-783. doi:10.1016/j.biopsych.2006.03.074

771. Petrakis IL, Poling J, Levinson C, Nich C, Carroll K, Rounsaville B. Naltrexone and disulfiram in patients with alcohol dependence and comorbid psychiatric disorders. *Biol Psychiatry* 2005; **57**(10): 1128-1137. doi:10.1016/j.biopsych.2005.02.016

772. Petrakis IL, Ralevski E, Desai N, et al. Noradrenergic vs serotonergic antidepressant with or without naltrexone for veterans with PTSD and comorbid alcohol dependence. *Neuropsychopharmacology* 2012; **37**(4): 996-1004. doi:10.1038/npp.2011.283

773. Pettinati HM, Oslin DW, Kampman KM, et al. A double-blind, placebo-controlled trial combining sertraline and naltrexone for treating co-occurring depression and alcohol dependence. *AM J PSYCHIATRY* 2010; **167**(6): 668-675. doi:10.1176/appi.ajp.2009.08060852

774. Pfeiffer PN, Pope B, Houck M, et al. Effectiveness of peer-supported computer-based CBT for depression among veterans in primary care. *Psychiatr Serv* 2020; **71**(3): 256-262. doi:10.1176/appi.ps.201900283

775. Philip KEJ, Owles H, McVey S, et al. An online breathing and wellbeing programme (ENO Breathe) for people with persistent symptoms following COVID-19: a parallel-group, single-blind, randomised controlled trial. *Lancet Respir Med* 2022; **10**(9): 851-862. doi:10.1016/S2213-2600(22)00125-4

776. Philips B, Wennberg P, Konradsson P, Franck J. Mentalization-Based Treatment for Concurrent Borderline Personality Disorder and Substance Use Disorder: A Randomized Controlled Feasibility Study. *Eur Addict Res* 2018; **24**(1): 1-8. doi:10.1159/000485564

777. Pinxsterhuis I, Sandvik L, Strand EB, Bautz-Holter E, Sveen U. Effectiveness of a group-based self-management program for people with chronic fatigue syndrome: A randomized controlled trial. *Clin Rehabil* 2017; **31**(1): 93-103. doi:10.1177/0269215515621362

778. Pistorello J, Fruzzetti AE, MacLane C, Gallop R, Iverson KM. Dialectical behavior therapy (DBT) applied to college students: A randomized clinical trial. *J Consult Clin Psychol* 2012; **80**(6): 982-994. doi:10.1037/a0029096

779. Plant KM, Sanders MR. Reducing problem behavior during care-giving in families of preschool-aged children with developmental disabilities. *Res Dev Disabil* 2007; **28**(4): 362-385. doi:10.1016/j.ridd.2006.02.009

780. Pleguezuelos E, Del Carmen A, Moreno E, Miravitlles M, Serra M, Garnacho-Castaño MV. Effects of a telerehabilitation program and detraining on cardiorespiratory fitness in patients with post-COVID-19 sequelae: A randomized controlled trial. *Scand J Med Sci Sports* 2024; **34**(1). doi:10.1111/sms.14543

781. Pol TM, Hendriks V, Rigter H, et al. Multidimensional family therapy in adolescents with a cannabis use disorder: Long-term effects on delinquency in a randomized controlled trial. *Child Adolesc Psychiatry Ment Health* 2018; **12**(1). doi:10.1186/s13034-018-0248-x

782. Polman NJ, Ebisch RMF, Heideman DAM, et al. Performance of human papillomavirus testing on self-collected versus clinician-collected samples for the detection of cervical intraepithelial neoplasia of grade 2 or worse: a randomised, paired screen-positive, non-inferiority trial. *Lancet Oncol* 2019; **20**(2): 229-238. doi:10.1016/S1470-2045(18)30763-0

783. Poppelaars M, Tak YR, Lichtwarck-Aschoff A, et al. A randomized controlled trial comparing two cognitive-behavioral programs for adolescent girls with subclinical depression: A school-based program (Op Volle Kracht) and a computerized program (SPARX). *Behav Res Ther* 2016; **80**: 33-42. doi:10.1016/j.brat.2016.03.005

784. Potter SDS, Brown RG, Fleminger S. Randomised, waiting list controlled trial of cognitive-behavioural therapy for persistent postconcussional symptoms after predominantly mild-moderate traumatic brain injury. *Journal of Neurology, Neurosurgery and Psychiatry* 2016; **87**(10): 1075-1083. doi:10.1136/jnnp-2015-312838

785. Powell BJ, Campbell JL, Landon JF, et al. A Double‐Blind, Placebo‐Controlled Study of Nortriptyline and Bromocriptine in Male Alcoholics Subtyped by Comorbid Psychiatric Disorders. *Alcohol Clin Exp Res* 1995; **19**(2): 462-468. doi:10.1111/j.1530-0277.1995.tb01532.x

786. Powell J, Heslin J, Greenwood R. Community based rehabilitation after severe traumatic brain injury: A randomised controlled trial. *J Neurol Neurosurg Psychiatry* 2002; **72**(2): 193-202. doi:10.1136/jnnp.72.2.193

787. Pownall HJ, Bray GA, Wagenknecht LE, et al. Changes in body composition over 8 years in a randomized trial of a lifestyle intervention: The look AHEAD study. *Obesity* 2015; **23**(3): 565-572. doi:10.1002/oby.21005

788. Priebe S, Bhatti N, Barnicot K, et al. Effectiveness and cost-effectiveness of dialectical behaviour therapy for self-harming patients with personality disorder: A pragmatic randomised controlled trial. *Psychother Psychosom* 2012; **81**(6): 356-365. doi:10.1159/000338897

789. Primosch RE, Antony SJ, Courts FJ. The efficacy of preoperative analgesic administration for postoperative pain management of pediatric dental patients. *Anesth Pain Control Dent* 1993; **2**(2): 102-106. doi:N

790. Primosch RE, Nichols DL, Courts FJ. Comparison of preoperative ibuprofen, acetaminophen, and placebo administration on the parental report of postextraction pain in children. *Pediatr Dent* 1995; **17**(3): 187-191. doi:N

791. Proudfoot J, Goldberg D, Mann A, Everitt B, Marks I, Gray JA. Computerized, interactive, multimedia cognitive-behavioural program for anxiety and depression in general practice. *Psychol Med* 2003; **33**(2): 217-227. doi:10.1017/S0033291702007225

792. Pugh NE, Hadjistavropoulos HD, Dirkse D. A randomised controlled trial of Therapist-Assisted, Internet-delivered Cognitive Behavior Therapy for women with maternal depression. *PLoS ONE* 2016; **11**(3). doi:10.1371/journal.pone.0149186

793. Pulcini A, Bollaín J, Sanz-Sánchez I, et al. Clinical effects of the adjunctive use of a 0.03% chlorhexidine and 0.05% cetylpyridinium chloride mouth rinse in the management of peri-implant diseases: A randomized clinical trial. *J Clin Periodontol* 2019; **46**(3): 342-353. doi:10.1111/jcpe.13088

794. Punamäki RL, Paavonen J, Toikka S, Solantaus T. Effectiveness of preventive family intervention in improving cognitive attributions among children of depressed parents: A randomized study. *J Fam Psychol* 2013; **27**(4): 683-690. doi:10.1037/a0033466

795. Raby WN, Rubin EA, Garawi F, et al. A randomized, double-blind, placebo-controlled trial of venlafaxine for the treatment of depressed cocaine-dependent patients. *Am J Addict* 2014; **23**(1): 68-75. doi:10.1111/j.1521-0391.2013.12065.x

796. Radford K, Sutton C, Sach T, et al. Early, specialist vocational rehabilitation to facilitate return to work after traumatic brain injury: The FRESH feasibility RCT. *Health Technol Assess* 2018; **22**(33): 1-123. doi:10.3310/hta22330

797. Raevuori A, Vahlberg T, Korhonen T, Hilgert O, Aittakumpu-Hyden R, Forman-Hoffman V. A therapist-guided smartphone app for major depression in young adults: A randomized clinical trial. *Journal of Affective Disorders* 2021; **286**: 228-238. doi:10.1016/j.jad.2021.02.007

798. Raitio K, Kaunonen M, Aho AL. Evaluating a bereavement follow-up intervention for grieving mothers after the death of a child. *Scand J Caring Sci* 2015; **29**(3): 510-520. doi:10.1111/scs.12183

799. Raj SR, Black BK, Biaggioni I, Harris PA, Robertson D. Acetylcholinesterase inhibition improves tachycardia in postural tachycardia syndrome. *Circulation* 2005; **111**(21): 2734-2740. doi:10.1161/CIRCULATIONAHA.104.497594

800. Ramzy EA. Comparative Efficacy of Newer Antidepressants in Combination with Pregabalin for Fibromyalgia Syndrome: A Controlled, Randomized Study. *Pain Pract* 2017; **17**(1): 32-40. doi:10.1111/papr.12409

801. Raskin J, Pritchett YL, Wang F, et al. A double-blind, randomized multicenter trial comparing duloxetine with placebo in the management of diabetic peripheral neuropathic pain. *Pain Med (USA)* 2005; **6**(5): 346-356. doi:10.1111/j.1526-4637.2005.00061.x

802. Raskin J, Wang F, Pritchett YL, Goldstein DJ. Duloxetine for patients with diabetic peripheral neuropathic pain: A 6-month open-label safety study. *Pain Med (USA)* 2006; **7**(5): 373-385. doi:10.1111/j.1526-4637.2006.00207.x

803. Raskind MA, Williams T, Holmes H, et al. A randomized controlled clinical trial of prazosin for alcohol use disorder in active duty soldiers: Predictive effects of elevated cardiovascular parameters. *Alcohol: Clinical and Experimental Research* 2023; **47**(2): 348-360. doi:10.1111/acer.14989

804. Rasmussen IE, Løk M, Durrer CG, et al. Impact of high-intensity interval training on cardiac structure and function after COVID-19: an investigator-blinded randomized controlled trial. *J Appl Physiol* 2023; **135**(2): 421-435. doi:10.1152/japplphysiol.00078.2023

805. Reay RE, Owen C, Shadbolt B, Raphael B, Mulcahy R, Wilkinson RB. Trajectories of long-term outcomes for postnatally depressed mothers treated with group interpersonal psychotherapy. *Arch Women's Ment Health* 2012; **15**(3): 217-228. doi:10.1007/s00737-012-0280-4

806. Rebergen DS, Bruinvels DJ, Bezemer PD, Van Der Beek AJ, Van Mechelen W. Guideline-based care of common mental disorders by occupational physicians (CO-OP study): A randomized controlled trial. *J Occup Environ Med* 2009; **51**(3): 305-312. doi:10.1097/JOM.0b013e3181990d32

807. Redmon JB, Bertoni AG, Connelly S, et al. Effect of the Look AHEAD study intervention on medication use and related cost to treat cardiovascular disease risk factors in individuals with type 2 diabetes. *Diabetes Care* 2010; **33**(6): 1153-1158. doi:10.2337/dc09-2090

808. Reedtz C, Handegård BH, Mørch WT. Promoting positive parenting practices in primary pare: Outcomes and mechanisms of change in a randomized controlled risk reduction trial. *Scand J Psychol* 2011; **52**(2): 131-137. doi:10.1111/j.1467-9450.2010.00854.x

809. Reedtz C, Klest S. Improved parenting maintained four years following a brief parent training intervention in a non-clinical sample. *BMC Psychol* 2016; **4**(1). doi:10.1186/s40359-016-0150-3

810. Reinke WM, Herman KC, Dong N. The Incredible Years Teacher Classroom Management Program: Outcomes from a Group Randomized Trial. *Prev Sci* 2018; **19**(8): 1043-1054. doi:10.1007/s11121-018-0932-3

811. Reme SE, Grasdal AL, Løvvik C, Lie SA, Øverland S. Work-focused cognitive-behavioural therapy and individual job support to increase work participation in common mental disorders: A randomised controlled multicentre trial. *Occupational and Environmental Medicine* 2015; **72**(10): 745-752. doi:10.1136/oemed-2014-102700

812. Reme SE, Tveito TH, Harris A, et al. Cognitive interventions and nutritional supplements (The CINS trial) a randomized controlled, multicenter trial comparing a brief intervention with additional cognitive behavioral therapy, seal oil, and soy oil for sick-listed low back pain patients. *Spine* 2016; **41**(20): 1557-1564. doi:10.1097/BRS.0000000000001596

813. Reuben JD, Shaw DS, Brennan LM, Dishion TJ, Wilson MN. A family-based intervention for improving children's emotional problems through effects on maternal depressive symptoms. *J Consult Clin Psychol* 2015; **83**(6): 1142-1148. doi:10.1037/ccp0000049

814. Ricca V, Castellini G, Mannucci E, et al. Comparison of individual and group cognitive behavioral therapy for binge eating disorder. A randomized, three-year follow-up study. *Appetite* 2010; **55**(3): 656-665. doi:10.1016/j.appet.2010.09.019

815. Rice IM, Pohlig RT, Gallagher JD, Boninger ML. Handrim wheelchair propulsion training effect on overground propulsion using biomechanical real-time visual feedback. *Arch Phys Med Rehabil* 2013; **94**(2): 256-263. doi:10.1016/j.apmr.2012.09.014

816. Rice LA, Smith I, Kelleher AR, Greenwald K, Boninger ML. Impact of a wheelchair education protocol based on practice guidelines for preservation of upper-limb function: A randomized trial. *Arch Phys Med Rehabil* 2014; **95**(1): 10-19.e11. doi:10.1016/j.apmr.2013.06.028

817. Richard S, Baud-Bovy G, Clerc-Georgy A, Gentaz E. The effects of a ‘pretend play-based training’ designed to promote the development of emotion comprehension, emotion regulation, and prosocial behaviour in 5- to 6-year-old Swiss children. *Br J Psychol* 2021; **112**(3): 690-719. doi:10.1111/bjop.12484

818. Richards SCM, Scott DL. Prescribed exercise in people with fibromyalgia: Parallel group randomised controlled trial. *Br Med J* 2002; **325**(7357): 185-187. doi:10.1136/bmj.325.7357.185

819. Richardson JS, Fann JR, Bell KR, Temkin N. Impact of Telephone-Based Problem-Solving Treatment on the Use of Medical and Psychological Services in the Military. *J Head Trauma Rehabil* 2018; **33**(2): E1-E6. doi:10.1097/HTR.0000000000000299

820. Ritvo P, Knyahnytska Y, Pirbaglou M, et al. Online mindfulness-based cognitive behavioral therapy intervention for youth with major depressive disorders: Randomized controlled trial. *J Med Internet Res* 2021; **23**(3). doi:10.2196/24380

821. Rizzi-Maia CC, Maia-Filho EM, Nelson-Filho P, et al. Single vs two-session root canal treatment: A preliminary randomized clinical study using cone beam computed tomography. *J Contemp Dental Pract* 2016; **17**(7): 515-521. doi:10.5005/jp-journals-10024-1882

822. Roberts C, Kane R, Thomson H, Bishop B, Hart B. The prevention of depressive symptoms in rural school children: A randomized controlled trial. *J Consult Clin Psychol* 2003; **71**(3): 622-628. doi:10.1037/0022-006X.71.3.622

823. Roberts CM, Kane R, Bishop B, Cross D, Fenton J, Hart B. The prevention of anxiety and depression in children from disadvantaged schools. *Behav Res Ther* 2010; **48**(1): 68-73. doi:10.1016/j.brat.2009.09.002

824. Robin AL, Siegel PT, Koepke T, Move AW, Tice S. Family therapy versus individual therapy for adolescent females with anorexia nervosa. *J Dev Behav Pediatr* 1994; **15**(2): 111-116. doi:10.1097/00004703-199404000-00008

825. Robin AL, Siegel PT, Moye AW, Gilroy M, Dennis AB, Sikand A. A controlled comparison of family versus individual therapy for adolescents with anorexia nervosa. *J AM ACAD CHILD ADOLESC PSYCHIATRY* 1999; **38**(12): 1482-1489. doi:10.1097/00004583-199912000-00008

826. Robinson P, Hellier J, Barrett B, et al. The NOURISHED randomised controlled trial comparing mentalisation-based treatment for eating disorders (MBT-ED) with specialist supportive clinical management (SSCM-ED) for patients with eating disorders and symptoms of borderline personality disorder. *Trials* 2016; **17**(1). doi:10.1186/s13063-016-1606-8

827. Robson S, McParlin C, Mossop H, et al. Ondansetron and metoclopramide as second-line antiemetics in women with nausea and vomiting in pregnancy: The empower pilot FACTORIAL RCT. *Health Technol Assess* 2021; **25**(63): VII-78. doi:10.3310/HTA25630

828. Roche G, Ponthieux A, Parot-Shinkel E, et al. Comparison of a Functional Restoration Program With Active Individual Physical Therapy for Patients With Chronic Low Back Pain: A Randomized Controlled Trial. *Arch Phys Med Rehabil* 2007; **88**(10): 1229-1235. doi:10.1016/j.apmr.2007.07.014

829. Rock CL, Flatt SW, Pakiz B, et al. Weight loss, glycemic control, and cardiovascular disease risk factors in response to differential diet composition in a weight loss program in type 2 diabetes: A randomized controlled trial. *Diabetes Care* 2014; **37**(6): 1573-1580. doi:10.2337/dc13-2900

830. Rockloff MJ, Donaldson P, Browne M. Jackpot expiry: An experimental investigation of a new EGM player-protection feature. *J Gambl Stud* 2015; **31**(4): 1505-1514. doi:10.1007/s10899-014-9472-3

831. Rohde P, Stice E, Shaw H, Gau JM. Cognitive-behavioral group depression prevention compared to bibliotherapy and brochure control: Nonsignificant effects in pilot effectiveness trial with college students. *Behav Res Ther* 2014; **55**(1): 48-53. doi:10.1016/j.brat.2014.02.003

832. Rohde P, Stice E, Shaw H, Gau JM. Effectiveness trial of an indicated cognitive-behavioral group adolescent depression prevention program versus bibliotherapy and brochure control at 1- and 2-year follow-up. *J Consult Clin Psychol* 2015; **83**(4): 736-747. doi:10.1037/ccp0000022

833. Rollman BL, Belnap BH, Abebe KZ, et al. Effectiveness of online collaborative care for treating mood and anxiety disorders in primary care: A randomized clinical trial. *JAMA Psychiatry* 2018; **75**(1): 56-64. doi:10.1001/jamapsychiatry.2017.3379

834. Romanet C, Wormser J, Fels A, et al. Effectiveness of exercise training on the dyspnoea of individuals with long COVID: A randomised controlled multicentre trial. *Ann Phys Rehabil Med* 2023; **66**(5). doi:10.1016/j.rehab.2023.101765

835. Ronzi Y, Roche-Leboucher G, Bègue C, et al. Efficiency of three treatment strategies on occupational and quality of life impairments for chronic low back pain patients: Is the multidisciplinary approach the key feature to success? *Clin Rehabil* 2017; **31**(10): 1364-1373. doi:10.1177/0269215517691086

836. Rooney R, Hassan S, Kane R, Roberts CM, Nesa M. Reducing depression in 9-10 year old children in low SES schools: Alongitudinal universal randomized controlled trial. *Behav Res Ther* 2013; **51**(12): 845-854. doi:10.1016/j.brat.2013.09.005

837. Rose K, Hawes DJ, Hunt CJ. Randomized controlled trial of a friendship skills intervention on adolescent depressive symptoms. *J Consult Clin Psychol* 2014; **82**(3): 510-520. doi:10.1037/a0035827

838. Rosenstock J, Tuchman M, Lamoreaux L, Sharma U. Pregabalin for the treatment of painful diabetic peripheral neuropathy: A double-blind, placebo-controlled trial. *Pain* 2004; **110**(3): 628-638. doi:10.1016/j.pain.2004.05.001

839. Rossignol M, Abenhaim L, Séguin P, et al. Coordination of primary health care for back pain: A randomized controlled trial. *Spine* 2000; **25**(2): 251-259. doi:10.1097/00007632-200001150-00018

840. Rossouw TI, Fonagy P. Mentalization-based treatment for self-harm in adolescents: A randomized controlled trial. *J AM ACAD CHILD ADOLESC PSYCHIATRY* 2012; **51**(12): 1304-1313.e1303. doi:10.1016/j.jaac.2012.09.018

841. Rouhe H, Salmela-Aro K, Toivanen R, et al. Group psychoeducation with relaxation for severe fear of childbirth improves maternal adjustment and childbirth experience-a randomised controlled trial. *J Psychosom Obstet Gynecol* 2015; **36**(1): 1-9. doi:10.3109/0167482X.2014.980722

842. Rouhe H, Salmela-Aro K, Toivanen R, Tokola M, Halmesmäki E, Saisto T. Obstetric outcome after intervention for severe fear of childbirth in nulliparous women - Randomised trial. *BJOG Int J Obstet Gynaecol* 2013; **120**(1): 75-84. doi:10.1111/1471-0528.12011

843. Rouhe H, Salmela-Aro K, Toivanen R, Tokola M, Halmesmäki E, Saisto T. Life satisfaction, general well-being and costs of treatment for severe fear of childbirth in nulliparous women by psychoeducative group or conventional care attendance. *Acta Obstet Gynecol Scand* 2015; **94**(5): 527-533. doi:10.1111/aogs.12594

844. Routhier F, Kirby RL, Demers L, Depa M, Thompson K. Efficacy and retention of the french-canadian version of the wheelchair skills training program for manual wheelchair users: A randomized controlled trial. *Arch Phys Med Rehabil* 2012; **93**(6): 940-948. doi:10.1016/j.apmr.2012.01.017

845. Rowbotham MC, Goli V, Kunz NR, Lei D. Venlafaxine extended release in the treatment of painful diabetic neuropathy: A double-blind, placebo-controlled study. *Pain* 2004; **110**(3): 697-706. doi:10.1016/j.pain.2004.05.010

846. Rowhani-Rahbar A, Oesterle S, Gause EL, et al. Effect of the Communities That Care Prevention System on Adolescent Handgun Carrying: A Cluster-Randomized Clinical Trial. *JAMA Netw Open* 2023; **6**(4): E236699. doi:10.1001/jamanetworkopen.2023.6699

847. Roy A. Placebo-controlled study of sertraline in depressed recently abstinent alcoholics. *Biol Psychiatry* 1998; **44**(7): 633-637. doi:10.1016/S0006-3223(97)00509-X

848. Roy-Byrne PP, Pages KP, Russo JE, et al. Nefazodone treatment of major depression in alcohol-dependent patients: A double-blind, placebo-controlled trial. *J Clin Psychopharmacol* 2000; **20**(2): 129-136. doi:10.1097/00004714-200004000-00003

849. Rubio G, Martínez I, Ponce G, Jiménez-Arriero MA, López-Muñoz F, Álamo C. Long-acting injectable risperidone compared with zuclopenthixol in the treatment of schizophrenia with substance abuse comorbidity. *Can J Psychiatry* 2006; **51**(8): 531-539. doi:10.1177/070674370605100808

850. Ruggenenti P, Abbate M, Ruggiero B, et al. Renal and systemic effects of calorie restriction in patients with type 2 diabetes with abdominal obesity: A randomized controlled trial. *Diabetes* 2017; **66**(1): 75-86. doi:10.2337/db16-0607

851. Rytter HM, Westenbaek K, Henriksen H, Christiansen P, Humle F. Specialized interdisciplinary rehabilitation reduces persistent post-concussive symptoms: a randomized clinical trial. *Brain Inj* 2019; **33**(3): 266-281. doi:10.1080/02699052.2018.1552022

852. Rössler W, Kawohl W, Nordt C, Haker H, Rüsch N, Hengartner MP. 'Placement budgets' for supported employment: Impact on employment rates in a multicentre randomised controlled trial. *Br J Psychiatry* 2020; **216**(6): 308-313. doi:10.1192/bjp.2019.154

853. Safari HR, Fassett MJ, Souter IC, Alsulyman OM, Goodwin TM. The efficacy of methylprednisolone in the treatment of hyperemesis gravidarum: A randomized, double-blind, controlled study. *AM J OBSTET GYNECOL* 1998; **179**(4): 921-924. doi:10.1016/S0002-9378(98)70189-9

854. Safer DL, Robinson AH, Jo B. Outcome from a randomized controlled trial of group therapy for binge eating disorder: comparing dialectical behavior therapy adapted for binge eating to an active comparison group therapy. *Behav Ther* 2010; **41**(1): 106-120. doi:10.1016/j.beth.2009.01.006

855. Safer DL, Telch CF, Agras WS. Dialectical behavior therapy for bulimia nervosa. *AM J PSYCHIATRY* 2001; **158**(4): 632-634. doi:10.1176/appi.ajp.158.4.632

856. Sahakian V, Rouse D, Sipes S, Rose N, Niebyl J. Vitamin B6 is effective therapy for nausea and vomiting of pregnancy: A randomized, double-blind placebo-controlled study. *Obstet Gynecol* 1991; **78**(1): 33-36. doi:10.1016/0020-7292(92)90077-V

857. Saisto T, Salmela-Aro K, Nurmi JE. A randomized controlled trial of intervention in fear of childbirth. *Obstet Gynecol* 2001; **98**(5): 820-826. doi:10.1016/S0029-7844(01)01552-6

858. Salazar AM, Warden DL, Schwab K, et al. Cognitive rehabilitation for traumatic brain injury: A randomized trial. *J Am Med Assoc* 2000; **283**(23): 3075-3081. doi:10.1001/jama.283.23.3075

859. Salloum IM, Cornelius JR, Daley DC, Kirisci L, Himmelhoch JM, Thase ME. Efficacy of valproate maintenance in patients with bipolar disorder and alcoholism: A double-blind placebo-controlled study. *Arch Gen Psychiatry* 2005; **62**(1): 37-45. doi:10.1001/archpsyc.62.1.37

860. Salomonsson S, Santoft F, Lindsäter E, et al. Effects of cognitive behavioural therapy and return-to-work intervention for patients on sick leave due to stress-related disorders: Results from a randomized trial. *Scand J Psychol* 2020; **61**(2): 281-289. doi:10.1111/sjop.12590

861. Salomonsson S, Santoft F, Lindsäter E, et al. Cognitive-behavioural therapy and return-to-work intervention for patients on sick leave due to common mental disorders: A randomised controlled trial. *Occupational and Environmental Medicine* 2017; **74**(12): 905-912. doi:10.1136/oemed-2017-104342

862. Samaha FF, Iqbal N, Seshadri P, et al. A low-carbohydrate as compared with a low-fat diet in severe obesity. *New Engl J Med* 2003; **348**(21): 2074-2081. doi:10.1056/NEJMoa022637

863. Samper-Pardo M, León-Herrera S, Oliván-Blázquez B, Méndez-López F, Domínguez-García M, Sánchez-Recio R. Effectiveness of a telerehabilitation intervention using ReCOVery APP of long COVID patients: a randomized, 3-month follow-up clinical trial. *Sci Rep* 2023; **13**(1). doi:10.1038/s41598-023-35058-y

864. Sánchez-Mil Z, Abuín-Porra V, Romero-Morale C, Almazán-Pol J, Saornil JV. Effectiveness of a respiratory rehabilitation program including an inspiration training device versus traditional respiratory rehabilitation: a randomized controlled trial. *PeerJ* 2023; **11**: e16360. doi:10.7717/peerj.16360

865. Sanders MR, Baker S, Turner KMT. A randomized controlled trial evaluating the efficacy of Triple P Online with parents of children with early-onset conduct problems. *Behav Res Ther* 2012; **50**(11): 675-684. doi:10.1016/j.brat.2012.07.004

866. Sanders MR, Dittman CK, Farruggia SP, Keown LJ. A comparison of online versus workbook delivery of a self-help positive parenting program. *J Prim Prev* 2014; **35**(3): 125-133. doi:10.1007/s10935-014-0339-2

867. Sanders MR, Markie-Dadds C, Tully LA, Bor W. The Triple P-positive parenting program: A comparison of enhanced, standard, and self-directed behavioral family intervention for parents of children with early onset conduct problems. *J Consult Clin Psychol* 2000; **68**(4): 624-640. doi:10.1037/0022-006X.68.4.624

868. Sandler I, Gunn H, Mazza G, et al. Effects of a Program to Promote High Quality Parenting by Divorced and Separated Fathers. *Prev Sci* 2018; **19**(4): 538-548. doi:10.1007/s11121-017-0841-x

869. Sandler I, Wolchik S, Mazza G, et al. Randomized Effectiveness Trial of the New Beginnings Program for Divorced Families with Children and Adolescents. *J Clin Child Adolesc Psychol* 2020; **49**(1): 60-78. doi:10.1080/15374416.2018.1540008

870. Santamarina-Perez P, Mendez I, Singh MK, et al. Adapted Dialectical Behavior Therapy for Adolescents with a High Risk of Suicide in a Community Clinic: A Pragmatic Randomized Controlled Trial. *Suicide Life-Threat Behav* 2020; **50**(3): 652-667. doi:10.1111/sltb.12612

871. Santana K, França E, Sato J, et al. Non-invasive brain stimulation for fatigue in post-acute sequelae of SARS-CoV-2 (PASC). *Brain Stimul* 2023; **16**(1): 100-107. doi:10.1016/j.brs.2023.01.1672

872. Saral I, Sindel D, Esmaeilzadeh S, Sertel-Berk HO, Oral A. The effects of long- and short-term interdisciplinary treatment approaches in women with fibromyalgia: a randomized controlled trial. *Rheumatol Int* 2016; **36**(10): 1379-1389. doi:10.1007/s00296-016-3473-8

873. Sato J, Kanazawa A, Makita S, et al. A randomized controlled trial of 130 g/day low-carbohydrate diet in type 2 diabetes with poor glycemic control. *Clin Nutr* 2017; **36**(4): 992-1000. doi:10.1016/j.clnu.2016.07.003

874. Satoh J, Yagihashi S, Baba M, et al. Efficacy and safety of pregabalin for treating neuropathic pain associated with diabetic peripheral neuropathy: A 14 week, randomized, double-blind, placebo-controlled trial. *Diabet Med* 2011; **28**(1): 109-116. doi:10.1111/j.1464-5491.2010.03152.x

875. Sawyer A, Kaim A, Le HN, et al. The effectiveness of an app-based nurse-moderated program for new mothers with depression and parenting problems (EMUMS Plus): Pragmatic randomized controlled trial. *J Med Internet Res* 2019; **21**(6). doi:10.2196/13689

876. Sawyer AM, Borduin CM. Effects of multisystemic therapy through midlife: A 21.9-year follow-up to a randomized clinical trial with serious and violent juvenile offenders. *J Consult Clin Psychol* 2011; **79**(5): 643-652. doi:10.1037/a0024862

877. Scavenius C, Chacko A, Lindberg MR, et al. Parent Management Training Oregon Model and Family-Based Services as Usual for Behavioral Problems in Youth: A National Randomized Controlled Trial in Denmark. *Child Psychiatry Hum Dev* 2020; **51**(5): 839-852. doi:10.1007/s10578-020-01028-y

878. Schaeffer CM, Henggeler SW, Ford JD, Mann M, Chang R, Chapman JE. RCT of a promising vocational/employment program for high-risk juvenile offenders. *J Subst Abuse Treat* 2014; **46**(2): 134-143. doi:10.1016/j.jsat.2013.06.012

879. Scheel IB, Birger Hagen K, Herrin J, Carling C, Oxman AD. Blind faith? The effects of promoting active sick leave for back pain patients: A cluster-randomized controlled trial. *Spine* 2002; **27**(23): 2734-2740. doi:10.1097/00007632-200212010-00014

880. Scheenen ME, Visser-Keizer AC, De Koning ME, et al. Cognitive Behavioral Intervention Compared to Telephone Counseling Early after Mild Traumatic Brain Injury: A Randomized Trial. *J Neurotrauma* 2017; **34**(19): 2713-2720. doi:10.1089/neu.2016.4885

881. Scheidt CE, Waller E, Endorf K, et al. Is brief psychodynamic psychotherapy in primary fibromyalgia syndrome with concurrent depression an effective treatment? A randomized controlled trial. *Gen Hosp Psychiatry* 2013; **35**(2): 160-167. doi:10.1016/j.genhosppsych.2012.10.013

882. Schepens EJA, Blijleven EE, Boek WM, et al. Prednisolone does not improve olfactory function after COVID-19: a randomized, double-blind, placebo-controlled trial. *BMC Med* 2022; **20**(1). doi:10.1186/s12916-022-02625-5

883. Schilling EA, Lawless M, Buchanan L, Aseltine RH, Jr. "Signs of suicide" shows promise as a middle school suicide prevention program. *Suicide Life-Threat Behav* 2014; **44**(6): 653-667. doi:10.1111/sltb.12097

884. Schlup B, Munsch S, Meyer AH, Margraf J, Wilhelm FH. The efficacy of a short version of a cognitive-behavioral treatment followed by booster sessions for binge eating disorder. *Behav Res Ther* 2009; **47**(7): 628-635. doi:10.1016/j.brat.2009.04.003

885. Schmidt AM, Schiøttz-Christensen B, Foster NE, Laurberg TB, Maribo T. The effect of an integrated multidisciplinary rehabilitation programme alternating inpatient interventions with home-based activities for patients with chronic low back pain: a randomized controlled trial. *Clin Rehabil* 2020; **34**(3): 382-393. doi:10.1177/0269215519897968

886. Schmidt K, Worrack S, Von Korff M, et al. Effect of a primary care management intervention on mental health-related qualityof life among survivors of sepsis a randomized clinical trial. *JAMA* 2016; **315**(24): 2703-2711. doi:10.1001/jama.2016.7207

887. Schmidt KF, Schwarzkopf D, Baldwin LM, et al. Long-Term Courses of Sepsis Survivors: Effects of a Primary Care Management Intervention. *Am J Med* 2020; **133**(3): 381-385.e385. doi:10.1016/j.amjmed.2019.08.033

888. Schmidt S, Grossman P, Schwarzer B, Jena S, Naumann J, Walach H. Treating fibromyalgia with mindfulness-based stress reduction: Results from a 3-armed randomized controlled trial. *Pain* 2011; **152**(2): 361-369. doi:10.1016/j.pain.2010.10.043

889. Schmidt U, Lee S, Beecham J, et al. A randomized controlled trial of family therapy and cognitive behavior therapy guided self-care for adolescents with bulimia nervosa and related disorders. *AM J PSYCHIATRY* 2007; **164**(4): 591-598. doi:10.1176/ajp.2007.164.4.591

890. Schmidt U, Magill N, Renwick B, et al. The Maudsley Outpatient Study of Treatments for Anorexia Nervosa and Related Conditions (MOSAIC): Comparison of the Maudsley Model of Anorexia Nervosa Treatment for Adults (MANTRA) with specialist supportive clinical management (SSCM) in outpatients with broadly defined anorexia nervosa: A randomized controlled trial. *J Consult Clin Psychol* 2015; **83**(4): 796-807. doi:10.1037/ccp0000019

891. Schmidt U, Oldershaw A, Jichi F, et al. Out-patient psychological therapies for adults with anorexia nervosa: Randomised controlled trial. *Br J Psychiatry* 2012; **201**(5): 392-399. doi:10.1192/bjp.bp.112.112078

892. Schmitz JM, Averill P, Stotts AL, Moeller FG, Rhoades HM, Grabowski J. Fluoxetine treatment of cocaine-dependent patients with major depressive disorder. *Drug Alcohol Depend* 2001; **63**(3): 207-214. doi:10.1016/S0376-8716(00)00208-8

893. Schneider R. Low-frequency vibrotherapy considerably improves the effectiveness of manual lymphatic drainage (MLD) in patients with lipedema: A two-armed, randomized, controlled pragmatic trial. *Physiother Theory Pract* 2020; **36**(1): 63-70. doi:10.1080/09593985.2018.1479474

894. Schnell T, Koethe D, Krasnianski A, et al. Ziprasidone versus clozapine in the treatment of dually diagnosed (DD) patients with schizophrenia and cannabis use disorders: A randomized study. *Am J Addict* 2014; **23**(3): 308-312. doi:10.1111/j.1521-0391.2014.12126.x

895. Schonert-Reichl KA, Oberle E, Lawlor MS, et al. Enhancing cognitive and social-emotional development through a simple-to-administer mindfulness-based school program for elementary school children: A randomized controlled trial. *Dev Psychol* 2015; **51**(1): 52-66. doi:10.1037/a0038454

896. Schubiner H, Saules KK, Arfken CL, et al. Double-blind placebo-controlled trial of methylphenidate in the treatment of adult ADHD patients with comorbid cocaine dependence. *Exp Clin Psychopharmacol* 2002; **10**(3): 286-294. doi:10.1037/1064-1297.10.3.286

897. Schweikert B, Jacobi E, Seitz R, et al. Effectiveness and cost-effectiveness of adding a cognitive behavioral treatment to the rehabilitation of chronic low back pain. *J Rheumatol* 2006; **33**(12): 2519-2526. doi:N

898. Scott S, O'Connor TG, Futh A, Matias C, Price J, Doolan M. Impact of a parenting program in a high-risk, multi-ethnic community: The PALS trial. *J Child Psychol Psychiatry Allied Discip* 2010; **51**(12): 1331-1341. doi:10.1111/j.1469-7610.2010.02302.x

899. Scott S, Sylva K, Doolan M, et al. Randomised controlled trial of parent groups for child antisocial behaviour targeting multiple risk factors: The SPOKES project. *J Child Psychol Psychiatry Allied Discip* 2010; **51**(1): 48-57. doi:10.1111/j.1469-7610.2009.02127.x

900. Seo YR, Moon JH, Choi HJ, et al. Comparison of endoscopic papillary balloon dilation and sphincterotomy in young patients with CBD stones and gallstones. *Dig Dis Sci* 2014; **59**(5): 1042-1047. doi:10.1007/s10620-013-2949-6

901. Sephton SE, Salmon P, Weissbecker I, et al. Mindfulness meditation alleviates depressive symptoms in women with fibromyalgia: Results of a randomized clinical trial. *Arthritis Care Res* 2007; **57**(1): 77-85. doi:10.1002/art.22478

902. Sepúlveda AR, Anastasiadou D, Parks M, Gutiérrez E. A controlled study of the Collaborative Care Skills Workshops versus Psycho-educational Workshops among Spanish caregivers of relatives with an eating disorder. *Eur Eating Disord Rev* 2019; **27**(3): 247-262. doi:10.1002/erv.2658

903. Serrie A, Lange B, Steup A. Tapentadol prolonged-release for moderate-to-severe chronic osteoarthritis knee pain: a double-blind, randomized, placebo- and oxycodone controlled release-controlled study. *Curr Med Res Opin* 2017; **33**(8): 1423-1432. doi:10.1080/03007995.2017.1335189

904. Shaibani A, Fares S, Selam JL, et al. Lacosamide in Painful Diabetic Neuropathy: An 18-Week Double-Blind Placebo-Controlled Trial. *J Pain* 2009; **10**(8): 818-828. doi:10.1016/j.jpain.2009.01.322

905. Shamohammadi I, Kazemeyni S, Sadighi M, Hasanzadeh T, Dizavi A. Efficacy of tadalafil on improvement of men with erectile dysfunction caused by COVID-19: A randomized placebo-controlled trial. *Asian J Urol* 2024; **11**(1): 128-133. doi:10.1016/j.ajur.2022.05.006

906. Shapiro JR, Reba-Harrelson L, Dymek-Valentine M, Woolson SL, Hamer RM, Bulik CM. Feasibility and acceptability of CD-ROM-based cognitive-behavioural treatment for binge-eating disorder. *Eur Eating Disord Rev* 2007; **15**(3): 175-184. doi:10.1002/erv.787

907. Shaw DS, Connell A, Dishion TJ, Wilson MN, Gardner F. Improvements in maternal depression as a mediator of intervention effects on early childhood problem behavior. *Dev Psychopathol* 2009; **21**(2): 417-439. doi:10.1017/S0954579409000236

908. Shaw DS, Supplee L, Dishion TJ, Gardner F, Arnds K. Randomized trial of a family-centered approach to the prevention of early conduct problems: 2-Year effects of the family check-up in early childhood. *J Consult Clin Psychol* 2006; **74**(1): 1-9. doi:10.1037/0022-006X.74.1.1

909. Shelleby EC, Shaw DS, Dishion TJ, Wilson MN, Gardner F. Effects of the family check-up on reducing growth in conduct problems from toddlerhood through school age: An analysis of moderated mediation. *J Consult Clin Psychol* 2018; **86**(10): 856-867. doi:10.1037/ccp0000337

910. Sherwood Brown E, Davila D, Nakamura A, et al. A randomized, double-blind, placebo-controlled trial of quetiapine in patients with bipolar disorder, mixed or depressed phase, and alcohol dependence. *Alcohol Clin Exp Res* 2014; **38**(7): 2113-2118. doi:10.1111/acer.12445

911. Sherwood Brown E, McArdle M, Palka J, et al. A randomized, double-blind, placebo-controlled proof-of-concept study of ondansetron for bipolar and related disorders and alcohol use disorder. *Eur Neuropsychopharmacol* 2021; **43**: 92-101. doi:10.1016/j.euroneuro.2020.12.006

912. Shin HS, Song YA, Seo S. Effect of Nei-Guan point (P6) acupressure on ketonuria levels, nausea and vomiting in women with hyperemesis gravidarum. *J Adv Nurs* 2007; **59**(5): 510-519. doi:10.1111/j.1365-2648.2007.04342.x

913. Shirai K, Saiki A, Oikawa S, et al. The effects of partial use of formula diet on weight reduction and metabolic variables in obese type 2 diabetic patients - Multicenter trial. *Obes Res Clin Pract* 2013; **7**(1): e43-e54. doi:10.1016/j.orcp.2012.03.002

914. Sikander S, Ahmad I, Atif N, et al. Delivering the Thinking Healthy Programme for perinatal depression through volunteer peers: a cluster randomised controlled trial in Pakistan. *Lancet Psychiatry* 2019; **6**(2): 128-139. doi:10.1016/S2215-0366(18)30467-X

915. Silverberg ND, Hallam BJ, Rose A, et al. Cognitive-behavioral prevention of postconcussion syndrome in at-risk patients: A pilot randomized controlled trial. *J Head Trauma Rehabil* 2013; **28**(4): 313-322. doi:10.1097/HTR.0b013e3182915cb5

916. Simister HD, Tkachuk GA, Shay BL, Vincent N, Pear JJ, Skrabek RQ. Randomized Controlled Trial of Online Acceptance and Commitment Therapy for Fibromyalgia. *J Pain* 2018; **19**(7): 741-753. doi:10.1016/j.jpain.2018.02.004

917. Simpson DM, Robinson-Papp J, Van J, et al. Capsaicin 8% Patch in Painful Diabetic Peripheral Neuropathy: A Randomized, Double-Blind, Placebo-Controlled Study. *J Pain* 2017; **18**(1): 42-53. doi:10.1016/j.jpain.2016.09.008

918. Simpson RW, Wlodarczyk JH. Transdermal buprenorphine relieves neuropathic pain: A randomized, double-blind, parallel-group, placebo-controlled trial in diabetic peripheral neuropathic pain. *Diabetes Care* 2016; **39**(9): 1493-1500. doi:10.2337/dc16-0123

919. Simpson TL, Malte CA, Dietel B, et al. A pilot trial of Prazosin, an alpha-1 adrenergic antagonist, for comorbid alcohol dependence and posttraumatic stress disorder. *Alcohol Clin Exp Res* 2015; **39**(5): 808-817. doi:10.1111/acer.12703

920. Skagseth M, Fimland MS, Rise MB, Johnsen R, Borchgrevink PC, Aasdahl L. Effectiveness of adding a workplace intervention to an inpatient multimodal occupational rehabilitation program: A randomized clinical trial. *Scand J Work Environ Health* 2020; **46**(4): 356-363. doi:10.5271/sjweh.3873

921. Skouen JS, Grasdal A, Haldorsen EMH. Return to work after comparing outpatient multidisciplinary treatment programs versus treatment in general practice for patients with chronic widespread pain. *Eur J Pain* 2006; **10**(2): 145. doi:10.1016/j.ejpain.2005.02.005

922. Skouen JS, Grasdal AL, Haldorsen EMH, Ursin H. Relative cost-effectiveness of extensive and light multidisciplinary treatment programs versus treatment as usual for patients with chronic low back pain on long-term sick leave: Randomized controlled study. *Spine* 2002; **27**(9): 901-909. doi:10.1097/00007632-200205010-00002

923. Skryabina E, Taylor G, Stallard P. Effect of a universal anxiety prevention programme (FRIENDS) on children's academic performance: results from a randomised controlled trial. *J Child Psychol Psychiatry Allied Discip* 2016; **57**(11): 1297-1307. doi:10.1111/jcpp.12593

924. Smeets RJEM, Vlaeyen JWS, Hidding A, et al. Active rehabilitation for chronic low back pain: Cognitive-behavioral, physical, or both? First direct post-treatment results from a randomized controlled trial [ISRCTN22714229]. *BMC Musculoskelet Disord* 2006; **7**. doi:10.1186/1471-2474-7-5

925. Smelson DA, Ziedonis D, Williams J, et al. The efficacy of olanzapine for decreasing cue-elicited craving in individuals with schizophrenia and cocaine dependence: A preliminary report. *J Clin Psychopharmacol* 2006; **26**(1): 9-12. doi:10.1097/01.jcp.0000194624.07611.5e

926. Smith EC, Diedrich A, Raj SR, et al. Splanchnic venous compression enhances the effects of ß-blockade in the treatment of postural tachycardia syndrome. *J Am Heart Assoc* 2020; **9**(14). doi:10.1161/JAHA.120.016196

927. Smith EP, Osgood DW, Oh Y, Caldwell LC. Promoting Afterschool Quality and Positive Youth Development: Cluster Randomized Trial of the Pax Good Behavior Game. *Prev Sci* 2018; **19**(2): 159-173. doi:10.1007/s11121-017-0820-2

928. Smith JD, Dishion TJ, Shaw DS, Wilson MN. Indirect effects of fidelity to the family check-up on changes in parenting and early childhood problem behaviors. *J Consult Clin Psychol* 2013; **81**(6): 962-974. doi:10.1037/a0033950

929. Smith JD, Knoble NB, Zerr AA, Dishion TJ, Stormshak EA. Family Check-Up Effects Across Diverse Ethnic Groups: Reducing Early-Adolescence Antisocial Behavior by Reducing Family Conflict. *J Clin Child Adolesc Psychol* 2014; **43**(3): 400-414. doi:10.1080/15374416.2014.888670

930. Smith JD, Stormshak EA, Kavanagh K. Results of a Pragmatic Effectiveness–Implementation Hybrid Trial of the Family Check-Up in Community Mental Health Agencies. *Adm Policy Ment Health Ment Health Serv Res* 2015; **42**(3): 265-278. doi:10.1007/s10488-014-0566-0

931. Smith V, Begley C, Newell J, et al. Admission cardiotocography versus intermittent auscultation of the fetal heart in low-risk pregnancy during evaluation for possible labour admission – a multicentre randomised trial: the ADCAR trial. *BJOG Int J Obstet Gynaecol* 2019; **126**(1): 114-121. doi:10.1111/1471-0528.15448

932. Soler J, Pascual JC, Tiana T, et al. Dialectical behaviour therapy skills training compared to standard group therapy in borderline personality disorder: A 3-month randomised controlled clinical trial. *Behav Res Ther* 2009; **47**(5): 353-358. doi:10.1016/j.brat.2009.01.013

933. Spence SH, Donovan CL, March S, et al. A randomized controlled trial of online versus clinic-based CBT for adolescent anxiety. *J Consult Clin Psychol* 2011; **79**(5): 629-642. doi:10.1037/a0024512

934. Spijkers W, Jansen DEMC, Reijneveld SA. Effectiveness of Primary Care Triple P on child psychosocial problems in preventive child healthcare: A randomized controlled trial. *BMC Med* 2013; **11**(1). doi:10.1186/1741-7015-11-240

935. Spinelli MG, Endicott J, Leon AC, et al. A controlled clinical treatment trial of interpersonal psychotherapy for depressed pregnant women at 3 New York city sites. *J CLIN PSYCHIATRY* 2013; **74**(4): 393-399. doi:10.4088/JCP.12m07909

936. Staal JB, Hlobil H, Twisk JWR, Smid T, Köke AJA, Van Mechelen W. Graded Activity for Low Back Pain in Occupational Health Care: A Randomized, Controlled Trial. *Ann Intern Med* 2004; **140**(2): 77-84+I24. doi:10.7326/0003-4819-140-2-200401200-00007

937. Stallard P, Phillips R, Montgomery AA, et al. A cluster randomised controlled trial to determine the clinical effectiveness and cost-effectiveness of classroom-based cognitive-behavioural therapy (CBT) in reducing symptoms of depression in high-risk adolescents. *Health Technol Assess* 2013; **17**(47): i-xvii+1-109. doi:10.3310/hta17470

938. Stallard P, Skryabina E, Taylor G, et al. Classroom-based cognitive behaviour therapy (FRIENDS): A cluster randomised controlled trial to Prevent Anxiety in Children through Education in Schools (PACES). *Lancet Psychiatry* 2014; **1**(3): 185-192. doi:10.1016/S2215-0366(14)70244-5

939. Stattin H, Enebrink P, Özdemir M, Giannotta F. A national evaluation of parenting programs in Sweden: The short-term effects using an RCT effectiveness design. *J Consult Clin Psychol* 2015; **83**(6): 1069-1084. doi:10.1037/a0039328

940. Steardo L, Jr., Caivano V, Sampogna G, et al. Psychoeducational intervention for perinatal depression: Study protocol of a randomized controlled trial. *Front Psychiatry* 2019; **10**(FEB): 55. doi:10.3389/fpsyt.2019.00055

941. Stedman M, Pettinati HM, Brown ES, Kotz M, Calabrese JR, Raines S. A double-blind, placebo-controlled study with quetiapine as adjunct therapy with lithium or divalproex in bipolar I patients with coexisting alcohol dependence. *Alcohol Clin Exp Res* 2010; **34**(10): 1822-1831. doi:10.1111/j.1530-0277.2010.01270.x

942. Steen SL, Southard KA, Alan S, Logan HL, Jakobsen JR. An evaluation of preoperative ibuprofen for treatment of pain associated with orthodontic separator placement. *Am J Orthod Dentofacial Orthop* 2000; **118**(6): 629-635. doi:10.1067/mod.2000.110638

943. Steenstra IA, Anema JR, Bongers PM, De Vet HCW, Knol DL, Van Mechelen W. The effectiveness of graded activity for low back pain in occupational healthcare. *Occupational and Environmental Medicine* 2006; **63**(11): 718-725. doi:10.1136/oem.2005.021675

944. Stein A, Netsi E, Lawrence PJ, et al. Mitigating the effect of persistent postnatal depression on child outcomes through an intervention to treat depression and improve parenting: a randomised controlled trial. *Lancet Psychiatry* 2018; **5**(2): 134-144. doi:10.1016/S2215-0366(18)30006-3

945. Stein MD, Solomon DA, Anderson BJ, et al. Persistence of antidepressant treatment effects in a pharmacotherapy plus psychotherapy trial for active injection drug users. *Am J Addict* 2005; **14**(4): 346-357. doi:10.1080/10550490591003684

946. Stein MD, Solomon DA, Herman DS, et al. Pharmacotherapy Plus Psychotherapy for Treatment of Depression in Active Injection Drug Users. *Arch Gen Psychiatry* 2004; **61**(2): 152-159. doi:10.1001/archpsyc.61.2.152

947. Sterner Isaksson S, Bensow Bacos M, Eliasson B, et al. Effects of nutrition education using a food-based approach, carbohydrate counting or routine care in type 1 diabetes: 12 months prospective randomized trial. *BMJ Open Diabetes Res Care* 2021; **9**(1). doi:10.1136/bmjdrc-2020-001971

948. Steuwe C, Berg M, Beblo T, Driessen M. Narrative Exposure Therapy in Patients With Posttraumatic Stress Disorder and Borderline Personality Disorder in a Naturalistic Residential Setting: A Randomized Controlled Trial. *Front Psychiatry* 2021; **12**: 765348. doi:10.3389/fpsyt.2021.765348

949. Stewart-Brown S, Patterson J, Mockford C, Barlow J, Klimes I, Pyper C. Impact of a general practice based group parenting programme: Quantitative and qualitative results from a controlled trial at 12 months. *Arch Dis Child* 2004; **89**(6): 519-525. doi:10.1136/adc.2003.028365

950. Stice E, Burton E, Kate Bearman S, Rohde P. Randomized trial of a brief depression prevention program: An elusive search for a psychosocial placebo control condition. *Behav Res Ther* 2007; **45**(5): 863-876. doi:10.1016/j.brat.2006.08.008

951. Stice E, Rohde P, Gau JM, Wade E. Efficacy trial of a brief cognitive-behavioral depression prevention program for high-risk adolescents: Effects at 1- and 2-year follow-up. *J Consult Clin Psychol* 2010; **78**(6): 856-867. doi:10.1037/a0020544

952. Stice E, Rohde P, Seeley JR, Gau JM. Brief Cognitive-Behavioral Depression Prevention Program for High-Risk Adolescents Outperforms Two Alternative Interventions: A Randomized Efficacy Trial. *J Consult Clin Psychol* 2008; **76**(4): 595-606. doi:10.1037/a0012645

953. Stormshak EA, Connell AM, Véronneau MH, et al. An Ecological Approach to Promoting Early Adolescent Mental Health and Social Adaptation: Family-Centered Intervention in Public Middle Schools. *Child Dev* 2011; **82**(1): 209-225. doi:10.1111/j.1467-8624.2010.01551.x

954. Stormshak EA, Fosco GM, Dishion TJ. Implementing Interventions with Families in Schools to Increase Youth School Engagement: The Family Check-Up Model. *Sch Ment Health* 2010; **2**(2): 82-92. doi:10.1007/s12310-009-9025-6

955. Strand LI, Ljunggren AE, Haldorsen EMH, Espehaug B. The impact of physical function and pain on work status at 1-year follow-up in patients with back pain. *Spine* 2001; **26**(7): 800-808. doi:10.1097/00007632-200104010-00022

956. Streimann K, Selart A, Trummal A. Effectiveness of a Universal, Classroom-Based Preventive Intervention (PAX GBG) in Estonia: a Cluster-Randomized Controlled Trial. *Prev Sci* 2020; **21**(2): 234-244. doi:10.1007/s11121-019-01050-0

957. Stuifbergen AK, Blozis SA, Becker H, et al. A randomized controlled trial of a wellness intervention for women with fibromyalgia syndrome. *Clin Rehabil* 2010; **24**(4): 305-318. doi:10.1177/0269215509343247

958. Sullivan CA, Johnson CA, Roach H, Martin RW, Stewart DK, Morrison JC. A pilot study of intravenous ondansetron for hyperemesis gravidarum. *AM J OBSTET GYNECOL* 1996; **174**(5): 1565-1568. doi:10.1016/S0002-9378(96)70607-5

959. Swedenhammar E, Strigård K, Emanuelsson P, Gunnarsson U, stark B. Long-term follow-up after surgical repair of abdominal rectus diastasis: A Prospective Randomized Study. *Scand J Surg* 2021; **110**(3): 283-289. doi:10.1177/1457496920913677

960. Szolnoky G, Borsos B, Bársony K, Balogh M, Kemény L. Complete decongestive physiotherapy with and without pneumatic compression for treatment of lipedema: A pilot study. *Lymphology* 2008; **41**(1): 40-44. doi:N

961. Taheri S, Zaghloul H, Chagoury O, et al. Effect of intensive lifestyle intervention on bodyweight and glycaemia in early type 2 diabetes (DIADEM-I): an open-label, parallel-group, randomised controlled trial. *Lancet Diabetes Endocrinol* 2020; **8**(6): 477-489. doi:10.1016/S2213-8587(20)30117-0

962. Tak YR, Kleinjan M, Lichtwarck-Aschoff A, Engels RCME. Secondary outcomes of a school-based universal resiliency training for adolescents: A cluster randomized controlled trial. *BMC Public Health* 2014; **14**(1). doi:10.1186/1471-2458-14-1171

963. Tak YR, Lichtwarck-Aschoff A, Gillham JE, Van Zundert RMP, Engels RCME. Universal School-Based Depression Prevention ‘Op Volle Kracht’: a Longitudinal Cluster Randomized Controlled Trial. *J Abnorm Child Psychol* 2016; **44**(5): 949-961. doi:10.1007/s10802-015-0080-1

964. Tamaki A. Effectiveness of home visits by mental health nurses for Japanese women with post-partum depression. *Int J Ment Health Nurs* 2008; **17**(6): 419-427. doi:10.1111/j.1447-0349.2008.00568.x

965. Tamminga SJ, Verbeek JHAM, Bos MMEM, et al. Effectiveness of a Hospital-Based Work Support Intervention for Female Cancer Patients - A Multi-Centre Randomised Controlled Trial. *PLoS ONE* 2013; **8**(5). doi:10.1371/journal.pone.0063271

966. Tamminga SJ, Verbeek JHAM, Bos MMEM, et al. Two-Year Follow-Up of a Multi-centre Randomized Controlled Trial to Study Effectiveness of a Hospital-Based Work Support Intervention for Cancer Patients. *J Occup Rehabil* 2019; **29**(4): 701-710. doi:10.1007/s10926-019-09831-8

967. Tan PC, Abdussyukur SA, Lim BK, Win ST, Omar SZ. Twelve-hour fasting compared with expedited oral intake in the initial inpatient management of hyperemesis gravidarum: a randomised trial. *BJOG Int J Obstet Gynaecol* 2020; **127**(11): 1430-1437. doi:10.1111/1471-0528.16290

968. Tan PC, Khine PP, Vallikkannu N, Omar SZ. Promethazine compared with metoclopramide for hyperemesis gravidarum: A randomized controlled trial. *Obstet Gynecol* 2010; **115**(5): 975-981. doi:10.1097/AOG.0b013e3181d99290

969. Tan PC, Norazilah MJ, Omar SZ. Dextrose saline compared with normal saline rehydration of hyperemesis gravidarum: A randomized controlled trial. *Obstet Gynecol* 2013; **121**(2 PART 1): 291-298. doi:10.1097/AOG.0b013e31827c5e99

970. Tan PC, Yow CM, Omar SZ. A placebo-controlled trial of oral pyridoxine in hyperemesis gravidarum. *Gynecol Obstet Invest* 2009; **67**(3): 151-157. doi:10.1159/000181182

971. Tanenberg RJ, Irving GA, Risser RC, et al. Duloxetine, pregabalin, and duloxetine plus gabapentin for diabetic peripheral neuropathic pain management in patients with inadequate pain response to gabapentin: An open-label, randomized, noninferiority comparison. *Mayo Clin Proc* 2011; **86**(7): 615-626. doi:10.4065/mcp.2010.0681

972. Targino RA, Imamura M, Kaziyama HHS, et al. A randomized controlled trial of acupuncture added to usual treatment for fibromyalgia. *J Rehabil Med* 2008; **40**(7): 582-588. doi:10.2340/16501977-0216

973. Taub PR, Zadourian A, Lo HC, Ormiston CK, Golshan S, Hsu JC. Randomized Trial of Ivabradine in Patients With Hyperadrenergic Postural Orthostatic Tachycardia Syndrome. *J Am Coll Cardiol* 2021; **77**(7): 861-871. doi:10.1016/j.jacc.2020.12.029

974. Tavafian SS, Jamshidi AR, Mohammad K. Treatment of chronic low back pain: A randomized clinical trial comparing multidisciplinary group-based rehabilitation program and oral drug treatment with oral drug treatment alone. *Clin J Pain* 2011; **27**(9): 811-818. doi:10.1097/AJP.0b013e31821e7930

975. Tavares DF, Myczkowski ML, Alberto RL, et al. Treatment of bipolar depression with deep TMS: Results from a double-blind, randomized, parallel group, sham-controlled clinical trial. *Neuropsychopharmacology* 2017; **42**(13): 2593-2601. doi:10.1038/npp.2017.26

976. Tay J, Luscombe-Marsh ND, Thompson CH, et al. A very low-carbohydrate, low-saturated fat diet for type 2 diabetes management: A randomized trial. *Diabetes Care* 2014; **37**(11): 2909-2918. doi:10.2337/dc14-0845

977. Tay J, Luscombe-Marsh ND, Thompson CH, et al. Comparison of low- and high-carbohydrate diets for type 2 diabetes management: A randomized trial. *Am J Clin Nutr* 2015; **102**(4): 780-790. doi:10.3945/ajcn.115.112581

978. Tay J, Thompson CH, Luscombe-Marsh ND, et al. Effects of an energy-restricted low-carbohydrate, high unsaturated fat/low saturated fat diet versus a high-carbohydrate, low-fat diet in type 2 diabetes: A 2-year randomized clinical trial. *Diabetes Obes Metab* 2018; **20**(4): 858-871. doi:10.1111/dom.13164

979. Tellegen CL, Sanders MR. A randomized controlled trial evaluating a brief parenting program with children with autism spectrum disorders. *J Consult Clin Psychol* 2014; **82**(6): 1193-1200. doi:10.1037/a0037246

980. Telles S, Singh N, Bhardwaj AK, Kumar A, Balkrishna A. Effect of yoga or physical exercise on physical, cognitive and emotional measures in children: A randomized controlled trial. *Child Adolesc Psychiatry Ment Health* 2013; **7**(1): 37. doi:10.1186/1753-2000-7-37

981. Thackwray DE, Smith MC, Bodfishk JW, Meyers AW. A Comparison of Behavioral and Cognitive-Behavioral Interventions for Bulimia Nervosa. *J Consult Clin Psychol* 1993; **61**(4): 639-645. doi:10.1037/0022-006X.61.4.639

982. Thayer AJ, Campa DM, Weeks MR, et al. Examining the Differential Effects of a Universal SEL Curriculum on Student Functioning Through the Dual Continua Model of Mental Health. *J Prim Prev* 2019; **40**(4): 405-427. doi:10.1007/s10935-019-00557-0

983. The Capsaicin Study G. Treatment of Painful Diabetic Neuropathy With Topical Capsaicin: A Multicenter, Double-blind, Vehicle-Controlled Study. *Arch Intern Med* 1991; **151**(11): 2225-2229. doi:10.1001/archinte.1991.00400110079017

984. Thienel U, Neto W, Schwabe SK, Vijapurkar U. Topiramate in painful diabetic polyneuropathy: Findings from three double-blind placebo-controlled trials. *Acta Neurol Scand* 2004; **110**(4): 221-231. doi:10.1111/j.1600-0404.2004.00338.x

985. Thomas SE, Randall PK, Book SW, Randall CL. A complex relationship between co-occurring social anxiety and alcohol use disorders: What effect does treating social anxiety have on drinking? *Alcohol Clin Exp Res* 2008; **32**(1): 77-84. doi:10.1111/j.1530-0277.2007.00546.x

986. Thorsteinsson EB, Loi NM, Farr K. Changes in stigma and help-seeking in relation to postpartum depression: Non-clinical parenting intervention sample. *PeerJ* 2018; **2018**(11): e5893. doi:10.7717/peerj.5893

987. Thöne-Mühling M, Swierkot K, Nonnenmacher C, Mutters R, Flores-de-Jacoby L, Mengel R. Comparison of two full-mouth approaches in the treatment of peri-implant mucositis: A pilot study. *Clin Oral Implants Res* 2010; **21**(5): 504-512. doi:10.1111/j.1600-0501.2009.01861.x

988. Tiersky LA, Anselmi V, Johnston MV, et al. A trial of neuropsychologic rehabilitation in mild-spectrum traumatic brain injury. *Arch Phys Med Rehabil* 2005; **86**(8): 1565-1574. doi:10.1016/j.apmr.2005.03.013

989. Timmons-Mitchell J, Bender MB, Kishna MA, Mitchell CC. An independent effectiveness trial of multisystemic therapy with juvenile justice youth. *J Clin Child Adolesc Psychol* 2006; **35**(2): 227-236. doi:10.1207/s15374424jccp3502_6

990. Titievsky J, Seco G, Barranco M, Kyle EM. Doxepin as adjunctive therapy for depressed methadone maintenance patients: A double-blind study. *J CLIN PSYCHIATRY* 1982; **43**(11 I): 454-456. doi:N

991. Tollefson GD, Montague-Clouse J, Tollefson SL. Treatment of comorbid generalized anxiety in a recently detoxified alcoholic population with a selective serotonergic drug (buspirone). *J Clin Psychopharmacol* 1992; **12**(1): 19-26. doi:10.1097/00004714-199202000-00004

992. Tolliver BK, Desantis SM, Brown DG, Prisciandaro JJ, Brady KT. A randomized, double-blind, placebo-controlled clinical trial of acamprosate in alcohol-dependent individuals with bipolar disorder: A preliminary report. *Bipolar Disord* 2012; **14**(1): 54-63. doi:10.1111/j.1399-5618.2011.00973.x

993. Toohill J, Callander E, Gamble J, Creedy DK, Fenwick J. A cost effectiveness analysis of midwife psycho-education for fearful pregnant women - a health system perspective for the antenatal period. *BMC Pregnancy Childbirth* 2017; **17**(1). doi:10.1186/s12884-017-1404-7

994. Toohill J, Fenwick J, Gamble J, et al. A Randomized Controlled Trial of a Psycho-Education Intervention by Midwives in Reducing Childbirth Fear in Pregnant Women. *Birth* 2014; **41**(4): 384-394. doi:10.1111/birt.12136

995. Tosato M, Calvani R, Picca A, et al. Effects of l-Arginine Plus Vitamin C Supplementation on Physical Performance, Endothelial Function, and Persistent Fatigue in Adults with Long COVID: A Single-Blind Randomized Controlled Trial. *Nutrients* 2022; **14**(23). doi:10.3390/nu14234984

996. Touyz S, Le Grange D, Lacey H, et al. Treating severe and enduring anorexia nervosa: A randomized controlled trial. *Psychol Med* 2013; **43**(12): 2501-2511. doi:10.1017/S0033291713000949

997. Treasure J, Schmidt U, Troop N, et al. First step in managing bulimia nervosa: Controlled trial of therapeutic manual. *BMJ* 1994; **308**(6930): 686. doi:10.1136/bmj.308.6930.686

998. Trento M, Trinetta A, Kucich C, et al. Carbohydrate counting improves coping ability and metabolic control in patients with Type 1 diabetes managed by Group Care. *J Endocrinol Invest* 2011; **34**(2): 101-105. doi:10.3275/7027

999. Trevillion K, Ryan EG, Pickles A, et al. An exploratory parallel-group randomised controlled trial of antenatal Guided Self-Help (plus usual care) versus usual care alone for pregnant women with depression: DAWN trial. *Journal of Affective Disorders* 2020; **261**: 187-197. doi:10.1016/j.jad.2019.10.013

1000. Trexler LE, Parrott DR, Malec JF. Replication of a Prospective Randomized Controlled Trial of Resource Facilitation to Improve Return to Work and School after Brain Injury. *Arch Phys Med Rehabil* 2016; **97**(2): 204-210. doi:10.1016/j.apmr.2015.09.016

1001. Trope M, Delano EO, Ørstavik D. Endodontic treatment of teeth with apical periodontitis: Single vs. multivisit treatment. *J Endod* 1999; **25**(5): 345-350. doi:10.1016/S0099-2399(06)81169-6

1002. Tully LA, Hunt C. A randomized controlled trial of a brief versus standard group parenting program for toddler aggression. *Aggress Behav* 2017; **43**(3): 291-303. doi:10.1002/ab.21689

1003. Turner AJ, Sutton M, Harrison M, Hennessey A, Humphrey N. Cost-Effectiveness of a School-Based Social and Emotional Learning Intervention: Evidence from a Cluster-Randomised Controlled Trial of the Promoting Alternative Thinking Strategies Curriculum. *Appl Health Econ Health Policy* 2020; **18**(2): 271-285. doi:10.1007/s40258-019-00498-z

1004. Turner-Stokes L, Erkeller-Yuksel F, Miles A, Pincus T, Shipley M, Pearce S. Outpatient cognitive behavioral pain management programs: A randomized comparison of a group-based multidisciplinary versus an individual therapy model. *Arch Phys Med Rehabil* 2003; **84**(6): 781-788. doi:10.1016/S0003-9993(03)00015-7

1005. Twamley EW, Thomas KR, Gregory AM, et al. CogSMART compensatory cognitive training for traumatic brain injury: Effects over 1 year. *J Head Trauma Rehabil* 2015; **30**(6): 391-401. doi:10.1097/HTR.0000000000000076

1006. Tölle T, Freynhagen R, Versavel M, Trostmann U, Young Jr JP. Pregabalin for relief of neuropathic pain associated with diabetic neuropathy: A randomized, double-blind study. *Eur J Pain* 2008; **12**(2): 203-213. doi:10.1016/j.ejpain.2007.05.003

1007. Uijen AA, Bischoff EWMA, Schellevis FG, Bor HHJ, Van Den Bosch WJHM, Schers HJ. Continuity in different care modes and its relationship to quality of life: A randomised controlled trial in patients with COPD. *Br J Gen Pract* 2012; **62**(599): e422-e428. doi:10.3399/bjgp12X649115

1008. Ulfsdotter M, Enebrink P, Lindberg L. Effectiveness of a universal health-promoting parenting program: A randomized waitlistcontrolled trial of All Children in Focus. *BMC Public Health* 2014; **14**(1). doi:10.1186/1471-2458-14-1083

1009. Upshur C, Wenz-Gross M, Reed G. A pilot study of a primary prevention curriculum to address preschool behavior problems. *J Prim Prev* 2013; **34**(5): 309-327. doi:10.1007/s10935-013-0316-1

1010. Uusitupa M, Laitinen J, Siitonen O, Vanninen E, Pyörälä K. The maintenance of improved metabolic control after intensified diet therapy in recent type 2 diabetes. *Diabetes Res Clin Pract* 1993; **19**(3): 227-238. doi:10.1016/0168-8227(93)90118-O

1011. Vallejo MA, Ortega J, Rivera J, Comeche MI, Vallejo-Slocker L. Internet versus face-to-face group cognitive-behavioral therapy for fibromyalgia: A randomized control trial. *J Psychiatr Res* 2015; **68**: 106-113. doi:10.1016/j.jpsychires.2015.06.006

1012. van Aar J, Leijten P, Orobio de Castro B, et al. Families Who Benefit and Families Who Do Not: Integrating Person- and Variable-Centered Analyses of Parenting Intervention Responses. *J AM ACAD CHILD ADOLESC PSYCHIATRY* 2019; **58**(10): 993-1003.e1001. doi:10.1016/j.jaac.2019.02.004

1013. van Beurden KM, Brouwers EPM, Joosen MCW, et al. Effectiveness of an Intervention to Enhance Occupational Physicians’ Guideline Adherence on Sickness Absence Duration in Workers with Common Mental Disorders: A Cluster-Randomized Controlled Trial. *J Occup Rehabil* 2017; **27**(4): 559-567. doi:10.1007/s10926-016-9682-x

1014. Van Den Bosch LMC, Koeter MWJ, Stijnen T, Verheul R, Van Den Brink W. Sustained efficacy of dialectical behaviour therapy for borderline personality disorder. *Behav Res Ther* 2005; **43**(9): 1231-1241. doi:10.1016/j.brat.2004.09.008

1015. Van den Hout JHC, Vlaeyen JWS, Heuts PHTG, Zijlema JHL, Wijnen JAG. Secondary prevention of work-related disability in nonspecific low back pain: Does problem-solving therapy help? A randomized clinical trial. *Clin J Pain* 2003; **19**(2): 87-96. doi:10.1097/00002508-200303000-00003

1016. Van Der Klink JJL, Blonk RWB, Schene AH, Van Dijk FJH. Reducing long term sickness absence by an activating intervention in adjustment disorders: A cluster randomised controlled design. *Occupational and Environmental Medicine* 2003; **60**(6): 429-437. doi:10.1136/oem.60.6.429

1017. Van Der Maas LCC, Köke A, Pont M, et al. Improving the Multidisciplinary Treatment of Chronic Pain by Stimulating Body Awareness: A Cluster-randomized Trial. *Clin J Pain* 2015; **31**(7): 660-669. doi:10.1097/AJP.0000000000000138

1018. Van Gordon W, Shonin E, Dunn TJ, Garcia-Campayo J, Griffiths MD. Meditation awareness training for the treatment of fibromyalgia syndrome: A randomized controlled trial. *Br J Health Psychol* 2017; **22**(1): 186-206. doi:10.1111/bjhp.12224

1019. Van Lier PAC, Muthén BO, Van Der Sar RM, Crijnen AAM. Preventing disruptive behavior in elementary schoolchildren: Impact of a universal classroom-based intervention. *J Consult Clin Psychol* 2004; **72**(3): 467-478. doi:10.1037/0022-006X.72.3.467

1020. Van Lier PAC, Vuijk P, Crijnen AAM. Understanding mechanisms of change in the development of antisocial behavior: The impact of a universal intervention. *J Abnorm Child Psychol* 2005; **33**(5): 521-535. doi:10.1007/s10802-005-6735-7

1021. Van Lieshout RJ, Layton H, Savoy CD, et al. Effect of Online 1-Day Cognitive Behavioral Therapy-Based Workshops plus Usual Care vs Usual Care Alone for Postpartum Depression: A Randomized Clinical Trial. *JAMA Psychiatry* 2021; **78**(11): 1200-1207. doi:10.1001/jamapsychiatry.2021.2488

1022. Van Lieshout RJ, Layton H, Savoy CD, et al. Public Health Nurse-delivered Group Cognitive Behavioural Therapy for Postpartum Depression: A Randomized Controlled Trial. *Can J Psychiatry* 2022; **67**(6): 432-440. doi:10.1177/07067437221074426

1023. Van Oostrom SH, Van Mechelen W, Terluin B, De Vet HCW, Knol DL, Anema JR. A workplace intervention for sick-listed employees with distress: Results of a randomised controlled trial. *Occupational and Environmental Medicine* 2010; **67**(9): 596-602. doi:10.1136/oem.2009.050849

1024. Van Ryzin MJ, Nowicka P. Direct and indirect effects of a family-based intervention in early adolescence on parent-youth relationship quality, late adolescent health, and early adult obesity. *J Fam Psychol* 2013; **27**(1): 106-116. doi:10.1037/a0031428

1025. Van Ryzin MJ, Stormshak EA, Dishion TJ. Engaging parents in the family check-up in middle school: Longitudinal effects on family conflict and problem behavior through the high school transition. *J Adolesc Health* 2012; **50**(6): 627-633. doi:10.1016/j.jadohealth.2011.10.255

1026. Vas J, Santos-Rey K, Navarro-Pablo R, et al. Acupuncture for fibromyalgia in primary care: A randomised controlled trial. *Acupunct Med* 2016; **34**(4): 257-266. doi:10.1136/acupmed-2015-010950

1027. Verbeek JH, Van Der Weide WE, Van Dijk FJ. Early occupational health management of patients with back pain: A randomized controlled trial. *Spine* 2002; **27**(17): 1844-1851. doi:10.1097/00007632-200209010-00006

1028. Verheul R, Van Den Bosch LMC, Koeter MWJ, De Ridder MAJ, Stijnen T, Van Den Brink W. Dialectical behaviour therapy for women with borderline personality disorder: 12-Month, randomised clinical trial in The Netherlands. *Br J Psychiatry* 2003; **182**(FEB.): 135-140. doi:10.1192/bjp.182.2.135

1029. Verkleij SPJ, Luijsterburg PAJ, Willemsen SP, Koes BW, Bohnen AM, Bierma-Zeinstra SMA. Effectiveness of diclofenac versus paracetamol in knee osteoarthritis: A randomised controlled trial in primary care. *Br J Gen Pract* 2015; **65**(637): e530-e537. doi:10.3399/bjgp15X686101

1030. Vermeulen SJ, Anema JR, Schellart AJM, Knol DL, Van Mechelen W, Van Der Beek AJ. A participatory return-to-work intervention for temporary agency workers and unemployed workers sick-listed due to musculoskeletal disorders: Results of a randomized controlled trial. *J Occup Rehabil* 2011; **21**(3): 313-324. doi:10.1007/s10926-011-9291-7

1031. Verra ML, Angst F, Brioschi R, et al. Effectiveness of subgroup-specific pain rehabilitation: A randomized controlled trial in patients with chronic back pain. *Eur J Phys Rehabil Med* 2018; **54**(3): 358-370. doi:10.23736/S1973-9087.17.04716-5

1032. Vidot DC, Huang S, Poma S, Estrada Y, Lee TK, Prado G. Familias Unidas' Crossover Effects on Suicidal Behaviors among Hispanic Adolescents: Results from an Effectiveness Trial. *Suicide Life-Threat Behav* 2016; **46**: S8-S14. doi:10.1111/sltb.12253

1033. Vieten C, Astin J. Effects of a mindfulness-based intervention during pregnancy on prenatal stress and mood: Results of a pilot study. *Arch Women's Ment Health* 2008; **11**(1): 67-74. doi:10.1007/s00737-008-0214-3

1034. Vigod SN, Hussain-Shamsy N, Stewart DE, et al. A patient decision aid for antidepressant use in pregnancy: Pilot randomized controlled trial. *Journal of Affective Disorders* 2019; **251**: 91-99. doi:10.1016/j.jad.2019.01.051

1035. Vigod SN, Murphy KE, Dennis CL, et al. Transcranial direct current stimulation (tDCS) for depression in pregnancy: A pilot randomized controlled trial. *Brain Stimul* 2019; **12**(6): 1475-1483. doi:10.1016/j.brs.2019.06.019

1036. Vigod SN, Slyfield Cook G, Macdonald K, et al. Mother Matters: Pilot randomized wait-list controlled trial of an online therapist-facilitated discussion board and support group for postpartum depression symptoms. *Depression Anxiety* 2021; **38**(8): 816-825. doi:10.1002/da.23163

1037. Viikari-Juntura E, Kausto J, Shiri R, et al. Return to work after early part-time sick leave due to musculoskeletal disorders: A randomized controlled trial. *Scand J Work Environ Health* 2012; **38**(2): 134-143. doi:10.5271/sjweh.3258

1038. Vikane E, Hellstrøm T, Røe C, Bautz-Holter E, Aßmus J, Skouen JS. Multidisciplinary outpatient treatment in patients with mild traumatic brain injury: A randomised controlled intervention study. *Brain Inj* 2017; **31**(4): 475-484. doi:10.1080/02699052.2017.1280852

1039. Vinik AI, Perrot S, Vinik EJ, et al. Capsaicin 8% patch repeat treatment plus standard of care (SOC) versus SOC alone in painful diabetic peripheral neuropathy: A randomised, 52-week, open-label, safety study. *BMC Neurol* 2016; **16**(1). doi:10.1186/s12883-016-0752-7

1040. Vlasveld MC, Van der Feltz-Cornelis CM, Adèr HJ, et al. Collaborative care for sick-listed workers with major depressive disorder: A randomised controlled trial from the netherlands depression initiative aimed at return to work and depressive symptoms. *Occupational and Environmental Medicine* 2013; **70**(4): 223-230. doi:10.1136/oemed-2012-100793

1041. Volanen SM, Lassander M, Hankonen N, et al. Healthy learning mind – Effectiveness of a mindfulness program on mental health compared to a relaxation program and teaching as usual in schools: A cluster-randomised controlled trial. *Journal of Affective Disorders* 2020; **260**: 660-669. doi:10.1016/j.jad.2019.08.087

1042. Volker D, Zijlstra-Vlasveld MC, Anema JR, et al. Effectiveness of a blended web-based intervention on return to work for sick-listed employees with common mental disorders: Results of a cluster randomized controlled trial. *J Med Internet Res* 2015; **17**(5). doi:10.2196/jmir.4097

1043. Vuijk P, van Lier PAC, Crijnen AAM, Huizink AC. Testing sex-specific pathways from peer victimization to anxiety and depression in early adolescents through a randomized intervention trial. *Journal of Affective Disorders* 2007; **100**(1-3): 221-226. doi:10.1016/j.jad.2006.11.003

1044. Vuksan V, Jenkins AL, Brissette C, et al. Salba-chia (Salvia hispanica L.) in the treatment of overweight and obese patients with type 2 diabetes: A double-blind randomized controlled trial. *Nutr Metab Cardiovasc Dis* 2017; **27**(2): 138-146. doi:10.1016/j.numecd.2016.11.124

1045. Wadden TA. Impact of intensive lifestyle intervention on depression and health-related quality of life in type 2diabetes: The lookahead trial. *Diabetes Care* 2014; **37**(6): 1544-1553. doi:10.2337/dc13-1928

1046. Wadden TA. Eight-year weight losses with an intensive lifestyle intervention: The look AHEAD study. *Obesity* 2014; **22**(1): 5-13. doi:10.1002/oby.20662

1047. Wade TD, Frayne A, Edwards SA, Robertson T, Gilchrist P. Motivational change in an inpatient anorexia nervosa population and implications for treatment. *AUST NEW ZEALAND J PSYCHIATRY* 2009; **43**(3): 235-243. doi:10.1080/00048670802653356

1048. Walker N, Parag V, Verbiest M, Laking G, Laugesen M, Bullen C. Nicotine patches used in combination with e-cigarettes (with and without nicotine) for smoking cessation: a pragmatic, randomised trial. *Lancet Respir Med* 2020; **8**(1): 54-64. doi:10.1016/S2213-2600(19)30269-3

1049. Walsh BT, Kaplan AS, Attia E, et al. Fluoxetine after weight restoration in anorexia nervosa: A randomized controlled trial. *J Am Med Assoc* 2006; **295**(22): 2605-2612. doi:10.1001/jama.295.22.2605

1050. Walton CJ, Bendit N, Baker AL, Carter GL, Lewin TJ. A randomised trial of dialectical behaviour therapy and the conversational model for the treatment of borderline personality disorder with recent suicidal and/or non-suicidal self-injury: An effectiveness study in an Australian public mental health service. *AUST NEW ZEALAND J PSYCHIATRY* 2020; **54**(10): 1020-1034. doi:10.1177/0004867420931164

1051. Wang C, Schmid CH, Fielding RA, et al. Effect of tai chi versus aerobic exercise for fibromyalgia: Comparative effectiveness randomized controlled trial. *BMJ (Online)* 2018; **360**. doi:10.1136/bmj.k851

1052. Wang C, Schmid CH, Rones R, et al. A randomized trial of tai chi for fibromyalgia. *New Engl J Med* 2010; **363**(8): 743-754. doi:10.1056/NEJMoa0912611

1053. Wang FL, Feldman JS, Lemery-Chalfant K, Wilson MN, Shaw DS. Family-Based Prevention of Adolescents’ Co-Occurring Internalizing/Externalizing Problems Through Early Childhood Parent Factors. *J Consult Clin Psychol* 2019; **87**(11): 1056-1067. doi:10.1037/ccp0000439

1054. Wang Z, Gao K, Kemp DE, et al. Lamotrigine adjunctive therapy to lithium and divalproex in depressed patients with rapid cycling bipolar disorder and a recent substance use disorder: A 12-week, double-blind, placebo-controlled pilot study. *Psychopharmacol Bull* 2010; **43**(4): 5-21. doi:N

1055. Watson N, Dyer K, Buckley J, et al. Effects of low-fat diets differing in protein and carbohydrate content on cardiometabolic risk factors during weight loss and weight maintenance in obese adults with type 2 diabetes. *Nutrients* 2016; **8**(5). doi:10.3390/nu8050289

1056. Watson NA, Dyer KA, Buckley JD, et al. Comparison of two low-fat diets, differing in protein and carbohydrate, on psychological wellbeing in adults with obesity and type 2 diabetes: A randomised clinical trial. *Nutr J* 2018; **17**(1). doi:10.1186/s12937-018-0367-5

1057. Webster-Stratton C. Preventing conduct problems in head start children: Strengthening parenting competencies. *J Consult Clin Psychol* 1998; **66**(5): 715-730. doi:10.1037/0022-006X.66.5.715

1058. Webster-Stratton C, Reid MJ, Hammond M. Preventing Conduct Problems, Promoting Social Competence: A Parent and Teacher Training Partnership in Head Start. *J Clin Child Adolesc Psychol* 2001; **30**(3): 283-302. doi:10.1207/S15374424JCCP3003_2

1059. Weeland J, Chhangur RR, van der Giessen D, Matthys W, de Castro BO, Overbeek G. Intervention Effectiveness of The Incredible Years: New Insights Into Sociodemographic and Intervention-Based Moderators. *Behav Ther* 2017; **48**(1): 1-18. doi:10.1016/j.beth.2016.08.002

1060. Weiger R, Rosendahl R, Löst C. Influence of calcium hydroxide intracanal dressings on the prognosis of teeth with endodontically induced periapical lesions. *Int Endod J* 2000; **33**(3): 219-226. doi:10.1046/j.1365-2591.1999.00298.x

1061. Weiss B, Han S, Harris V, et al. An independent randomized clinical trial of multisystemic therapy with non-court-referred adolescents with serious conduct problems. *J Consult Clin Psychol* 2013; **81**(6): 1027-1039. doi:10.1037/a0033928

1062. Wennerholm UB, Saltvedt S, Wessberg A, et al. Induction of labour at 41 weeks versus expectant management and induction of labour at 42 weeks (SWEdish Post-term Induction Study, SWEPIS): Multicentre, open label, randomised, superiority trial. *The BMJ* 2019; **367**. doi:10.1136/bmj.l6131

1063. Wernicke JF, Pritchett YL, D'Souza DN, et al. A randomized controlled trial of duloxetine in diabetic peripheral neuropathic pain. *Neurology* 2006; **67**(8): 1411-1420. doi:10.1212/01.wnl.0000240225.04000.1a

1064. Wheatley-Guy CM, Shea MG, Parks JK, et al. Semi-supervised exercise training program more effective for individuals with postural orthostatic tachycardia syndrome in randomized controlled trial. *Clin Auton Res* 2023; **33**(6): 659-672. doi:10.1007/s10286-023-00970-w

1065. Wickberg B, Hwang CP. Counselling of postnatal depression: A controlled study on a population based Swedish sample. *Journal of Affective Disorders* 1996; **39**(3): 209-216. doi:10.1016/0165-0327(96)00034-1

1066. Wien M, Oda K, Sabaté J. A randomized controlled trial to evaluate the effect of incorporating peanuts into an American Diabetes Association meal plan on the nutrient profile of the total diet and cardiometabolic parameters of adults with type 2 diabetes. *Nutr J* 2014; **13**(1). doi:10.1186/1475-2891-13-10

1067. Wijnhoven LAMW, Creemers DHM, Vermulst AA, Scholte RHJ, Engels RCME. Randomized controlled trial testing the effectiveness of a depression prevention program ('op volle kracht') among adolescent girls with elevated depressive symptoms. *J Abnorm Child Psychol* 2014; **42**(2): 217-228. doi:10.1007/s10802-013-9773-5

1068. Wilens TE, Adler LA, Weiss MD, et al. Atomoxetine treatment of adults with ADHD and comorbid alcohol use disorders. *Drug Alcohol Depend* 2008; **96**(1-2): 145-154. doi:10.1016/j.drugalcdep.2008.02.009

1069. Wilfley DE, Robinson Welch R, Stein RI, et al. A randomized comparison of group cognitive-behavioral therapy and group interpersonal psychotherapy for the treatment of overweight individuals with binge-eating disorder. *Arch Gen Psychiatry* 2002; **59**(8): 713-721. doi:10.1001/archpsyc.59.8.713

1070. Williams RJ, Wood RT, Currie SR. Stacked deck: An effective, school-based program for the prevention of problem gambling. *J Prim Prev* 2010; **31**(3): 109-125. doi:10.1007/s10935-010-0212-x

1071. Williamson DA, Rejeski J, Lang W, Van Dorsten B, Fabricatore AN, Toledo K. Impact of a weight management program on health-related quality of life in overweight adults with type 2 diabetes. *Arch Intern Med* 2009; **169**(2): 163-171. doi:10.1001/archinternmed.2008.544

1072. Wilson GT, Wilfley DE, Agras WS, Bryson SW. Psychological treatments of binge eating disorder. *Arch Gen Psychiatry* 2010; **67**(1): 94-101. doi:10.1001/archgenpsychiatry.2009.170

1073. Wilson IC, Alltop LB, Riley L. Tofranil in the Treatment of Post Alcoholic Depressions. *Psychosomatics* 1970; **11**(5): 488-494. doi:10.1016/S0033-3182(70)71614-9

1074. Wing RR, Bolin P, Brancati FL, et al. Cardiovascular effects of intensive lifestyle intervention in type 2 diabetes. *New Engl J Med* 2013; **369**(2): 145-154. doi:10.1056/NEJMoa1212914

1075. Witham MD, Adams F, McSwiggan S, et al. Effect of intermittent vitamin D3 on vascular function and symptoms in chronic fatigue syndrome - A randomised controlled trial. *Nutr Metab Cardiovasc Dis* 2015; **25**(3): 287-294. doi:10.1016/j.numecd.2014.10.007

1076. Witvliet M, van Lier PAC, Cuijpers P, Koot HM. Testing Links Between Childhood Positive Peer Relations and Externalizing Outcomes Through a Randomized Controlled Intervention Study. *J Consult Clin Psychol* 2009; **77**(5): 905-915. doi:10.1037/a0014597

1077. Wolchik SA, Sandler IN, Millsap RE, et al. Six-year follow-up of preventive interventions for children of divorce: A randomized controlled trial. *J Am Med Assoc* 2002; **288**(15): 1874-1881. doi:10.1001/jama.288.15.1874

1078. Wolchik SA, Sandler IN, Tein JY, et al. Fifteen-year follow-up of a randomized trial of a preventive intervention for divorced families: Effects on mental health and substance use outcomes in young adulthood. *J Consult Clin Psychol* 2013; **81**(4): 660-673. doi:10.1037/a0033235

1079. Wolchik SA, West SG, Sandler IN, et al. An experimental evaluation of theory-based mother and mother-child programs for children of divorce. *J Consult Clin Psychol* 2000; **68**(5): 843-856. doi:10.1037/0022-006X.68.5.843

1080. Wolever TMS, Gibbs AL, Mehling C, et al. The Canadian Trial of Carbohydrates in Diabetes (CCD), a 1-y controlled trial of low-glycemic-index dietary carbohydrate in type 2 diabetes: No effect on glycated hemoglobin but reduction in C-reactive protein. *Am J Clin Nutr* 2008; **87**(1): 114-125. doi:10.1093/ajcn/87.1.114

1081. Wolf E, Dragicevic M, Fuhrmann M. Alleviation of acute dental pain from localised apical periodontitis: A prospective randomised study comparing two emergency treatment procedures. *J Oral Rehabil* 2019; **46**(2): 120-126. doi:10.1111/joor.12730

1082. Wong SYS, Chan FWK, Wong RLP, et al. Comparing the effectiveness of mindfulness-based stress reduction and multidisciplinary intervention programs for chronic pain: A randomized comparative trial. *Clin J Pain* 2011; **27**(8): 724-734. doi:10.1097/AJP.0b013e3182183c6e

1083. Wood A, Trainor G, Rothwell J, Moore A, Harrington R. Randomized trial of group therapy for repeated deliberate self-harm in adolescents. *J AM ACAD CHILD ADOLESC PSYCHIATRY* 2001; **40**(11): 1246-1253. doi:10.1097/00004583-200111000-00003

1084. Woody GE, O'Brien CP, Rickels K. Depression and anxiety in heroin addicts: a placebo controlled study of doxepin in combination with methadone. *AM J PSYCHIATRY* 1975; **132**(4): 447-450. doi:10.1176/ajp.132.4.447

1085. Wormgoor MEA, Indahl A, Andersen E, Egeland J. Effectiveness of Briefer Coping-Focused Psychotherapy for Common Mental Complaints on Work-Participation and Mental Health: A Pragmatic Randomized Trial with 2-Year Follow-Up. *J Occup Rehabil* 2020; **30**(1): 22-39. doi:10.1007/s10926-019-09841-6

1086. Worobey LA, Kirby RL, Heinemann AW, et al. Effectiveness of Group Wheelchair Skills Training for People With Spinal Cord Injury: A Randomized Controlled Trial. *Arch Phys Med Rehabil* 2016; **97**(10): 1777-1784.e1773. doi:10.1016/j.apmr.2016.04.006

1087. Wozney L, Olthuis J, Lingley-Pottie P, et al. Strongest Families™ Managing Our Mood (MOM): a randomized controlled trial of a distance intervention for women with postpartum depression. *Arch Women's Ment Health* 2017; **20**(4): 525-537. doi:10.1007/s00737-017-0732-y

1088. Wycherley TP, Thompson CH, Buckley JD, et al. Long-term effects of weight loss with a very-low carbohydrate, low saturated fat diet on flow mediated dilatation in patients with type 2 diabetes: A randomised controlled trial. *Atherosclerosis* 2016; **252**: 28-31. doi:10.1016/j.atherosclerosis.2016.07.908

1089. Yan CH, Jang SS, Lin HFC, et al. Use of platelet-rich plasma for COVID-19–related olfactory loss: a randomized controlled trial. *Int Forum Allergy Rhinol* 2023; **13**(6): 989-997. doi:10.1002/alr.23116

1090. Yancy WS, Crowley MJ, Dar MS, et al. Comparison of Group Medical Visits Combined with Intensive Weight Management vs Group Medical Visits Alone for Glycemia in Patients with Type 2 Diabetes: A Noninferiority Randomized Clinical Trial. *JAMA Intern Med* 2020; **180**(1): 70-79. doi:10.1001/jamainternmed.2019.4802

1091. Yang M, Jia G, Sun S, Ye C, Zhang R, Yu X. Effects of an Online Mindfulness Intervention Focusing on Attention Monitoring and Acceptance in Pregnant Women: A Randomized Controlled Trial. *J Midwifery Women's Health* 2019; **64**(1): 68-77. doi:10.1111/jmwh.12944

1092. Yang R, Vigod SN, Hensel JM. Optional web-based videoconferencing added to office-based care for women receiving psychotherapy during the postpartum period: Pilot randomized controlled trial. *J Med Internet Res* 2019; **21**(6). doi:10.2196/13172

1093. Yasuda H, Hotta N, Nakao K, Kasuga M, Kashiwagi A, Kawamori R. Superiority of duloxetine to placebo in improving diabetic neuropathic pain: Results of a randomized controlled trial in Japan. *J Diabetes Invest* 2011; **2**(2): 132-139. doi:10.1111/j.2040-1124.2010.00073.x

1094. Yeo SS, Kwon JW. Wheelchair Skills Training for Functional Activity in Adults with Cervical Spinal Cord Injury. *Int J Sports Med* 2018; **39**(12): 924-928. doi:10.1055/a-0635-0941

1095. Yeung A, Wang F, Feng F, et al. Outcomes of an online computerized cognitive behavioral treatment program for treating chinese patients with depression: A pilot study. *Asian J Psychiatry* 2018; **38**: 102-107. doi:10.1016/j.ajp.2017.11.007

1096. Ylikorkala O, Kauppila A, Ollanketo ML. INTRAMUSCULAR ACTH OR PLACEBO IN THE TREATMENT OF HYPEREMESIS GRAVIDARUM. *Acta Obstet Gynecol Scand* 1979; **58**(5): 453-455. doi:10.3109/00016347909154066

1097. Yost NP, McIntire DD, Wians Jr FH, Ramin SM, Balko JA, Leveno KJ. A randomized, placebo-controlled trial of corticosteroids for hyperemesis due to pregnancy. *Obstet Gynecol* 2003; **102**(6): 1250-1254. doi:10.1016/j.obstetgynecol.2003.08.013

1098. Zargar SA, Mushtaq M, Beg MA, et al. Wait-and-see policy versus cholecystectomy after endoscopic sphincterotomy for bile-duct stones in high-risk patients with co-existing gallbladder stones: A prospective randomised trial. *Arab J Gastroenterol* 2014; **15**(1): 24-26. doi:10.1016/j.ajg.2014.01.005

1099. Zemestani M, Fazeli Nikoo Z. Effectiveness of mindfulness-based cognitive therapy for comorbid depression and anxiety in pregnancy: a randomized controlled trial. *Arch Women's Ment Health* 2020; **23**(2): 207-214. doi:10.1007/s00737-019-00962-8

1100. Zhao Y, Munro-Kramer ML, Shi S, Wang J, Zhao Q. Effects of antenatal depression screening and intervention among Chinese high-risk pregnant women with medically defined complications: A randomized controlled trial. *Early Intervent Psychiatry* 2019; **13**(5): 1090-1098. doi:10.1111/eip.12731

1101. Zilberman-Itskovich S, Catalogna M, Sasson E, et al. Hyperbaric oxygen therapy improves neurocognitive functions and symptoms of post-COVID condition: randomized controlled trial. *Sci Rep* 2022; **12**(1). doi:10.1038/s41598-022-15565-0

1102. Ziser K, Rheindorf N, Keifenheim K, et al. Motivation-Enhancing Psychotherapy for Inpatients With Anorexia Nervosa (MANNA): A Randomized Controlled Pilot Study. *Front Psychiatry* 2021; **12**. doi:10.3389/fpsyt.2021.632660

1103. Øverland S, Grasdal AL, Reme SE. Long-term effects on income and sickness benefits after work-focused cognitive-behavioural therapy and individual job support: A pragmatic, multicentre, randomised controlled trial. *Occupational and Environmental Medicine* 2018; **75**(10): 703-708. doi:10.1136/oemed-2018-105137
