# Supplementary material for: Combining search filters for randomized controlled trials with the Cochrane RCT Classifier in Covidence: a methodological validation study
Source: Res Synth Methods. 2025 Aug 28;16(6):953–60. doi: 10.1017/rsm.2025.10023 (PMC12657657; doi:10.1017/rsm.2025.10023)
Supplement: Moberg and Gornitzki supplementary material [file S1759287925100239sup001.zip › Appendix S3.docx]

**Appendix S3**

**Case study: Ovid MEDLINE search strategies**

*SBU Assessment 337: Internet-delivered psychological treatment versus other available treatment options for common mental disorders*

| **#** | **Searches** |
| --- | --- |
| 1 | Adjustment Disorders/ |
| 2 | exp Anxiety/ |
| 3 | exp Anxiety Disorders/ |
| 4 | Body Dysmorphic Disorders/ |
| 5 | Depression/ |
| 6 | Hoarding/ |
| 7 | Hypochondriasis/ |
| 8 | exp Mood Disorders/ |
| 9 | Mutism/ |
| 10 | Panic/ |
| 11 | exp Premenstrual Syndrome/ |
| 12 | exp Psychological Trauma/ |
| 13 | Reactive Attachment Disorder/ |
| 14 | Stress Disorders, Post-Traumatic/ |
| 15 | Stress Disorders, Traumatic/ |
| 16 | Stress Disorders, Traumatic, Acute/ |
| 17 | Trichotillomania/ |
| 18 | "Trauma and Stressor Related Disorders"/ |
| 19 | (acrophobi* or acute stress or adjustment disorder* or adjustment react* or aerophobi* or affective disorder* or agoraphobi* or anxiety* or anxieties or anxiodepressive or anxious* or arachnophobi* or attachment disorder* or aviophobi* or body dysmorphic disorder* or body image disfunction* or body image disorder* or dentophobi* or depressed or depression or depressive or depressiveness or depressivity or dermatillomani* or disinhibited social engagement disorder* or disruptive mood dysregulation disorder or dysmorphophobi* or dysphoria or dysphori* disorder* or dysphori* syndrom* or dysthymi* or emetophobi* or excoriation disorder* or GAD or glossophobi* or hair pulling or hoarding or hypochondri* or MDD or melanchol* or mood disorder* or mute or mutism or obsessive compulsive or OCD or odontophobi* or olfactory reference syndrome* or olfactory reference disorder* or ophidiophobi* or panic or phobi* or ((posttrauma* or post trauma*) adj3 (disorder* or neuros* or psych* or stress* or symptom* or syndrom*)) or premenstrual syndrom* or prolonged grief disorder* or PTSD or reactive disorder* or skin picking or transient situational disturbance or traumatic stress or trichotillomani*).ti,ab,kf. |
| 20 | or/1-19 |
| 21 | Computer-Assisted Instruction/ |
| 22 | Internet-Based Intervention/ |
| 23 | exp Remote Consultation/ |
| 24 | Telemedicine/ |
| 25 | Therapy, Computer-Assisted/ |
| 26 | (cCBT or c-CBT or cyber-counseling or cyber-counselling or cybercounseling or cybercounselling or digital health or e-consultation or eCBT or e-CBT or econsultation or e-counseling or e-counselling or ecounseling or ecounselling or e-health or ehealth or emedicine or e-medicine or emental health* or e-mental health* or e-portal or eportal or epsych* or e-psych* or e-therapy or etherapy or i-CBT or ICBT or m-health or mhealth or mobile health or Deprexis or Interapy or WeChat*).ti,ab,kf. |
| 27 | ((android or app or apps or blackberry or cellphone or ((cell* or mobile or smart) adj3 (phone* or telephone*)) or chat* or Computer* or cyber* or digital or electronic mail* or e-mail or email or handheld-device* or instant messag* or Internet* or ipad* or i-pad* or iphone* or i-phone* or messaging or mobile application* or Online* or on-line* or personal digital assistant* or short messag* or Smartphone* or SMS or tablet* or text messag* or texting or webbased or webdeliver* or web-based or web-deliver* or Website*) adj10 (ACT or (attention adj2 modification) or attention training or ((behavio* or cognitive) adj2 (activat* or component? or contracting or defusion or guidance or management or modif* or rehab* or restructur* or technique* or train*)) or CBT or Ccbt or Cognitive behavio* or consultation* or counceling or councelling or DBT or dialectical behavio* or hypnosis or hypnotherapy or intervention* or meditation or memory specificity training or mindfulness or minimal therapist support or "motivational interviewing" or program* or psychoanaly* or psycho-analy* or psychodrama or psycho-drama* or psychodynamic or psychoeducat* or psycho-educat* or psychotherap* or relaxation or ((self-care or selfcare or Self-help or selfhelp) adj3 (administered or guided or instruct* or strateg* or supervised or tool*)) or ((self-administered or selfadministered) adj3 (guided or instruct* or strateg* or supervised)) or self-management or support or therap* or treatment*)).ti,ab,kf. |
| 28 | or/21-27 |
| 29 | exp Cell Phone/ |
| 30 | exp Computers, Handheld/ |
| 31 | Electronic Mail/ |
| 32 | Internet/ |
| 33 | Mobile Applications/ |
| 34 | (android or app or apps or blackberry or cellphone or ((cell* or mobile or smart) adj3 (phone* or telephone*)) or chat* or computer* or cyber* or digital or electronic mail* or e-mail or email or handheld device* or instant messag* or internet* or ipad* or i-pad* or iphone* or i-phone* or messaging or mobile application* or online* or on-line* or personal digital assistant* or short messag* or smartphone* or SMS or tablet* or text messag* or texting or webbased or webdeliver* or web-based or web-deliver* or website*).ti. |
| 35 | or/29-34 |
| 36 | Counseling/ |
| 37 | exp Directive Counseling/ |
| 38 | Motivation/ |
| 39 | exp Psychotherapy/ |
| 40 | exp Relaxation Therapy/ |
| 41 | Self Care/ |
| 42 | Self-Help Groups/ |
| 43 | Self-Management/ |
| 44 | therapy.fs. |
| 45 | (behavio* or cognitive).ti. |
| 46 | (ACT or (attention adj2 modification) or attention training or ((behavio* or cognitive) adj2 (activat* or component? or contracting or defusion or guidance or management or modif* or rehab* or restructur* or technique* or train*)) or CBT or Cognitive behavio* or consultation* or counceling or councelling or DBT or dialectical behavio* or hypnosis or hypnotherapy or intervention* or meditation or memory specificity training or mindfulness or minimal therapist support or "motivational interviewing" or program* or psychoanaly* or psycho-analy* or psychodrama or psycho-drama* or psychodynamic or psychoeducat* or psycho-educat* or psychotherap* or relaxation or ((self-care or selfcare or Self-help or selfhelp) adj3 (administered or guided or instruct* or strateg* or supervised or tool*)) or ((self-administered or selfadministered) adj3 (guided or instruct* or strateg* or supervised)) or self-management or support or therap* or treatment*).ti,ab,kf. |
| 47 | or/36-46 |
| 48 | 20 and 28 |
| 49 | 20 and 35 and 47 |
| 50 | 48 or 49 |
| 51 | limit 50 to yr="2013 -Current" |
| 52 | limit 51 to (danish or english or norwegian or swedish) |
| 53 | exp randomized controlled trial/ |
| 54 | controlled clinical trial.pt. |
| 55 | randomized.ab. |
| 56 | placebo.ab. |
| 57 | drug therapy.fs. |
| 58 | randomly.ab. |
| 59 | trial.ab. |
| 60 | groups.ab. |
| 61 | or/53-60 |
| 62 | exp animals/ not humans.sh. |
| 63 | 61 not 62 |
| 64 | 52 and 63 |
| 65 | Adjustment Disorders/ |
| 66 | Body Dysmorphic Disorders/ |
| 67 | Hoarding/ |
| 68 | Hoarding Disorder/ |
| 69 | Hypochondriasis/ |
| 70 | Mutism/ |
| 71 | exp Premenstrual Syndrome/ |
| 72 | Reactive Attachment Disorder/ |
| 73 | Stress Disorders, Post-Traumatic/ |
| 74 | Stress Disorders, Traumatic, Acute/ |
| 75 | Trichotillomania/ |
| 76 | (Acute Stress or Adjustment Disorder* or Adjustment react* or Body Dysmorphic Disorder* or Body Image Disfunction* or Body Image Disorder* or dermatillomani* or Disinhibited Social Engagement Disorder* or Disruptive Mood Dysregulation Disorder* or Dysmorphophobi* or Excoriation disorder* or hair pulling or health anxiety or Hoarding or Hypochondri* or illness anxiety or mixed anxi* or mixed depressive or mutism or Olfactory reference disorder* or Olfactory reference syndrom* or ((posttrauma* or post trauma*) adj3 (disorder* or neuros* or psych* or stress* or symptom* or syndrom*)) or Premenstrual dysphori* or Premenstrual syndrom* or Prolonged grief disorder* or PTSD or Reactive Attachment Disorder* or skin picking or Trichotillomani*).ti,ab,kf. |
| 77 | or/65-76 |
| 78 | 77 and 28 |
| 79 | 77 and 35 and 47 |
| 80 | 78 or 79 |
| 81 | limit 80 to (danish or english or norwegian or swedish) |
| 82 | limit 81 to dt=18600101-20210826 |
| 83 | limit 52 to dt=18600101-20210826 |
| 84 | 82 or 83 |
| 85 | 63 and 81 |
| 86 | 64 or 85 |
| 87 | limit 86 to dt=18600101-20210826 |
| 88 | ("28270335" or "24035673" or "24245707" or "33974020" or "32401286" or "25929985" or "32673226" or "27609813" or "26089305" or "24949908" or "24911260" or "29902736" or "33211517" or "27565118" or "31931686" or "12622301" or "33743385" or "33688840" or "29117275" or "29146042").ui. |
| 89 | 88 not 84 |
| 90 | 88 not 87 |

*SBU Assessment 372: Treatment and social support for adults with co-occurring addictive and psychiatric disorders*

| **#** | **Searches** |
| --- | --- |
| 1 | Substance-Related Disorders/ or exp Alcohol-Related Disorders/ or Alcohol-Induced Disorders/ or Alcoholism/ or Amphetamine-Related Disorders/ or Cocaine-Related Disorders/ or Inhalant Abuse/ or Marijuana Abuse/ or Narcotic-Related Disorders/ or Opioid-Related Disorders/ or Heroin Dependence/ or Morphine Dependence/ or Opium Dependence/ or Phencyclidine Abuse/ or Psychoses, Substance-Induced/ or Substance Abuse, Intravenous/ or Substance Abuse, Oral/ or exp Illicit Drugs/ or exp Synthetic Drugs/ or Hallucinogens/ or Prescription Drug Misuse/ or Prescription Drug Overuse/ |
| 2 | ((amphetamine* or cannabis or cocaine or crack or hallucinogen* or hashish or heroin or inhalant* or marihuana or marijuana or narcotic* or phencyclidine or psychedelic* or sedative* or substance* or prescription) adj3 (abuse or abusing or addict* or dependen* or disorder* or misuse)).ti,ab,kf. |
| 3 | (((alcohol* or drug or opiate* or opioid*) adj3 (abuse or abusing or addict* or dependen* or disorder* or misuse)) or ((illicit or unsanctioned or non-medical or prescription) adj3 ("use" or misuse or abuse or abusing or addict* or depend* or disorder*) adj3 (drug* or opioid* or analgesic*))).ti,ab,kf. |
| 4 | (alcoholism or alcoholic* or chronic alcohol misuse or chronic excessive alcohol or "alcohol use disorder*").ti,ab,kf. |
| 5 | Gambling/ |
| 6 | (gambl* or betting).ti,ab,kf. |
| 7 | (roulette or casino* or poker or electronic blackjack* or gaming machine* or slot machine* or pokie* or "fruit machine" or bingo or sports bet* or bets or keno or lottery or lotteries or lotto or scratch card* or football pool* or wagering).ti,ab,kf. |
| 8 | 1 or 2 or 3 or 4 or 5 or 6 or 7 |
| 9 | Mental Disorders/ or Mentally Ill Persons/ |
| 10 | (((persistent or serious or severe*) adj3 (psychiatric or mental)) or psychiatric inpatient* or psychiatric in-patients or psychiatric disorder* or psychiatric condition* or mental healthcare or mental disorders* or mental illness or mentally ill).ti,ab,kf. |
| 11 | exp Anxiety Disorders/ or Agoraphobia/ or Anxiety, Separation/ or Neurocirculatory Asthenia/ or Neurotic Disorders/ or exp Obsessive-Compulsive Disorder/ or Panic Disorder/ or exp Phobic Disorders/ or Phobia, Social/ |
| 12 | (anxiet* or obsessive-compulsive disorder or phobic disorder* or neurotic disorder* or agoraphobia or phobia or panic disorder).ti,ab,kf. or ocd.ti. |
| 13 | exp "Bipolar and Related Disorders"/ or Bipolar Disorder/ |
| 14 | bipol*.ti,ab,kf. |
| 15 | "Disruptive, Impulse Control, and Conduct Disorders"/ or Firesetting Behavior/ or Trichotillomania/ |
| 16 | (impulse control disorder* or kleptomania or pyromania or arson* or trichotillomania*).ti,ab,kf. |
| 17 | exp Dissociative Disorders/ or Dissociative Identity Disorder/ |
| 18 | (dissociative disorder* or dissociative identity disorder* or dual personality or multiple identity disorder or multiple personalit*).ti,ab,kf. |
| 19 | exp Elimination Disorders/ |
| 20 | elimination disorder*.ti,ab,kf. |
| 21 | exp "Feeding and Eating Disorders"/ or Anorexia Nervosa/ or Avoidant Restrictive Food Intake Disorder/ or Binge-Eating Disorder/ or Bulimia Nervosa/ or Diabulimia/ or "Feeding and Eating Disorders of Childhood"/ or Food Addiction/ or Night Eating Syndrome/ or Orthorexia Nervosa/ or Pica/ or exp Relative Energy Deficiency in Sport/ or Rumination Syndrome/ |
| 22 | (anorexi* or bulimi* or eating disorder* or binge-eating disorder).ti,ab,kf. |
| 23 | Mood Disorders/ or Depressive Disorder/ or Depression, Postpartum/ or Depressive Disorder, Major/ or Depressive Disorder, Treatment-Resistant/ or Dysthymic Disorder/ or Premenstrual Dysphoric Disorder/ or Seasonal Affective Disorder/ or Cyclothymic Disorder/ or Depression/ |
| 24 | (depressive disorder* or depression or major depressive or mood disorder*).ti,ab,kf. |
| 25 | Motor Disorders/ or Motor Skills Disorders/ |
| 26 | (motor disorder* or motor skills disorder* or motor skill disorder*).ti,ab,kf. |
| 27 | Neurocognitive Disorders/ |
| 28 | neurocognitive disorder*.ti,ab,kf. |
| 29 | Neurodevelopmental Disorders/ or Anxiety, Separation/ or exp "Attention Deficit and Disruptive Behavior Disorders"/ or Child Behavior Disorders/ or exp Child Development Disorders, Pervasive/ or exp Communication Disorders/ or Developmental Disabilities/ or exp Intellectual Disability/ |
| 30 | (adhd or "attention-deficit/hyperactivity-disorder" or "attention deficit disorder*" or "attention deficit hyperactivity" or autism or autistic or "disruptive behavior" or "disruptive behaviour" or "hyperactivity syndrome*" or "hyperactivity syndrome*" or "hyperkinetic syndrome*" or "hyperkinetic disorder*" or "hyperkinetic conduct disorder*" or "inattentive hyperactive" or "intellectual disability" or "minimal brain dysfunction*" or neurodevelop*).ti,ab,kf. |
| 31 | Neurotic Disorders/ |
| 32 | (neurotic disorder* or neuroticism or neuroses).ti,ab,kf. |
| 33 | exp Paraphilic Disorders/ |
| 34 | (paraphilic disorder* or paraphilia* or sex deviation* or exhibitionism or psychiatric fetishism or masochism or pedophilia or sadism or transvestism or voyeurism).ti,ab,kf. |
| 35 | exp Personality Disorders/ or Antisocial Personality Disorder/ or Borderline Personality Disorder/ or Compulsive Personality Disorder/ or Dependent Personality Disorder/ or exp Histrionic Personality Disorder/ or Paranoid Personality Disorder/ or Passive-Aggressive Personality Disorder/ or Schizoid Personality Disorder/ or Schizotypal Personality Disorder/ |
| 36 | ((borderline or anti-social or antisocial or paranoid) adj3 (personality disorder* or behaviour or behavior)).mp. or (schizoid or schizotypal).ti,ab,kf. [mp=title, book title, abstract, original title, name of substance word, subject heading word, floating sub-heading word, keyword heading word, organism supplementary concept word, protocol supplementary concept word, rare disease supplementary concept word, unique identifier, synonyms, population supplementary concept word, anatomy supplementary concept word] |
| 37 | exp "Schizophrenia Spectrum and Other Psychotic Disorders"/ or Affective Disorders, Psychotic/ or Capgras Syndrome/ or Delusional Parasitosis/ or Morgellons Disease/ or Paranoid Disorders/ or exp Psychotic Disorders/ or exp Schizophrenia/ or Schizophrenia, Catatonic/ or Schizophrenia, Disorganized/ or Schizophrenia, Paranoid/ or Shared Paranoid Disorder/ |
| 38 | (schizophren* or psychosis or psychotic disorder* or schizoaffective or schizophrenia).ti,ab,kf. |
| 39 | exp Sexual Dysfunctions, Psychological/ or Dyspareunia/ or Erectile Dysfunction/ or Gender Dysphoria/ or Premature Ejaculation/ or "Sexual and Gender Disorders"/ or Vaginismus/ |
| 40 | (psychosexual disorder* or sexual aversion disorder* or psychological sexual dysfunction* or dyspareunia or erectile dysfunction or gender dysphoria or premature ejaculation or "sexual and gender disorders" or vaginismus).ti,ab,kf. |
| 41 | exp Sleep Wake Disorders/ or exp Dyssomnias/ or exp Parasomnias/ |
| 42 | sleep disorder*.ti,ab,kf. |
| 43 | exp Somatoform Disorders/ or exp Body Dysmorphic Disorders/ or exp Body Integrity Identity Disorder/ or exp Conversion Disorder/ or exp Factitious Disorders/ or exp Hypochondriasis/ or exp Neurasthenia/ |
| 44 | (somatoform disorder* or somatization disorder* or body dysmorphic disorder* or body integrity identity disorder* or conversion disorder* or globus sensation or factitious disorder* or munchausen syndrome or hypochondriasis or neurasthenia or medically unexplained syndrome*).ti,ab,kf. |
| 45 | "Trauma and Stressor Related Disorders"/ or Adjustment Disorders/ or exp Stress Disorders, Traumatic/ or Combat Disorders/ or Stress Disorders, Post-Traumatic/ or Stress Disorders, Traumatic, Acute/ |
| 46 | (chronic stress or posttraumatic stress or post-traumatic stress or PTSD or trauma-related mental health problem*).ti,ab,kf. |
| 47 | or/9-46 |
| 48 | exp randomized controlled trial/ |
| 49 | controlled clinical trial.pt. |
| 50 | randomized.ab. |
| 51 | placebo.ab. |
| 52 | drug therapy.fs. |
| 53 | randomly.ab. |
| 54 | trial.ab. |
| 55 | groups.ab. |
| 56 | 48 or 49 or 50 or 51 or 52 or 53 or 54 or 55 |
| 57 | exp animals/ not humans.sh. |
| 58 | 56 not 57 |
| 59 | 8 and 47 |
| 60 | "Diagnosis, Dual (Psychiatry)"/ or ((co-occurr* or comorbid* or co-morbid* or concurrent) adj1 (mental or psychiatric* or substance*)).ti,ab,kf. or (dual diagnosis or dually diagnosed).ti,ab,kf. |
| 61 | 59 or 60 |
| 62 | 58 and 61 |
| 63 | 62 not ((exp Infant/ or exp Child/ or Adolescent/) not Adult/) |
| 64 | limit 63 to (danish or english or norwegian or swedish) |
| 65 | limit 64 to dt=18600101-20230412 |
| 66 | 61 not ((exp Infant/ or exp Child/ or Adolescent/) not Adult/) |
| 67 | limit 66 to dt=18600101-20230412 |
| 68 | ("25679122" or "29148881" or "2079386" or "18327889" or "27736051" or "25092377" or "17448631" or "15770115" or "12233988" or "18312058" or "17873684" or "22669171" or "25998279" or "32160422" or "25914610" or "15099656" or "24055681" or "15730347" or "9283504" or "29950846" or "23925619" or "28536866" or "26302441" or "14633652" or "23325372" or "15084901" or "25622199" or "15625212" or "21169896" or "19192457" or "6342564" or "24118269" or "16415699" or "16930863" or "16102908" or "23297841" or "25887096" or "3067116" or "28351544" or "1335217" or "15913920" or "8611060" or "14766438" or "20958842" or "14624185" or "18834506" or "18348597" or "35058816" or "18681757" or "28051838" or "8556967" or "8494079" or "9477929" or "17374042" or "15956269" or "9649975" or "17414239" or "26683790" or "14634716" or "17008146" or "15866552" or "22089316" or "20231324" or "7625583" or "24313244" or "36809662" or "9787889" or "10770449" or "16933590" or "15630071" or "11418225" or "24628830" or "12233989" or "24976394" or "33402258" or "25827659" or "16415698" or "20626727" or "16188715" or "14757591" or "18028529" or "7174622" or "1552035" or "22329472" or "21240149" or "18403134" or "5470676" or "1091161").ui. |
| 69 | 68 not 67 |
| 70 | 68 not 65 |

*SBU Policy support 379: Treatment and rehabilitation of post-covid and other post-infectious conditions*

| **#** | **Searches** |
| --- | --- |
| 1 | COVID-19/rh |
| 2 | Coronavirus Infections/rh |
| 3 | ((covid* or coronavirus* or corona virus* or sars cov 2 or 2019 ncov) adj3 (after care or aftercare or chronic* or continuing or convalesc* or enduring or late or lingering or long* or non specific or nonspecific or permanent or persist* or post* or prolong* or rehab* or recover* or recurr* or relaps* or remaining or residing or residual or sequela* or sequelae* or telerehab*)).ti,kf. |
| 4 | ((covid* or coronavirus* or corona virus* or sars cov 2 or 2019 ncov) adj5 (after care or aftercare or chronic* or continuing or convalesc* or enduring or late or lingering or long* or longterm* or non specific or nonspecific or permanent or persist* or post* or prolong* or rehab* or recover* or recurr* or relaps* or remaining or residing or residual or sequela* or sequelae* or telerehab*) adj5 (anxiet* or arrhythmia* or arrythmia* or asthma* or brain or breath* or cardiac* or cognit* or depress* or dizz* or dyspnea* or dysrhythmia* or fatigue or fever* or gustat* or heart* or insomnia or kidney* or lung* or nausea or neuro* or olfact* or pain or palpitation* or postural orthostatic tachycardia syndrome or pots or pneumoni* or pulmonary or respirat* or sleep* or smell* or spinning or symptom* or tast* or vertigo*)).ab. |
| 5 | post-acute COVID-19 syndrome/ |
| 6 | postcovid*.ti,ab,kf. |
| 7 | or/1-6 |
| 8 | COVID-19/ |
| 9 | Coronavirus Infections/ |
| 10 | (covid* or coronavirus* or corona virus* or sars cov 2 or 2019 ncov).ti,ab,kf. |
| 11 | or/8-10 |
| 12 | (persist* adj5 symptom*).ti,ab,kf. |
| 13 | (long haul* or post acute or post viral or post virus or sequela* or sequelae*).ti,ab,kf. |
| 14 | or/12-13 |
| 15 | 11 and 14 |
| 16 | (long covid* or post covid*).ab. |
| 17 | 7 or 15 or 16 |
| 18 | exp randomized controlled trial/ |
| 19 | controlled clinical trial.pt. |
| 20 | randomized.ab. |
| 21 | placebo.ab. |
| 22 | drug therapy.fs. |
| 23 | randomly.ab. |
| 24 | trial.ab. |
| 25 | groups.ab. |
| 26 | 18 or 19 or 20 or 21 or 22 or 23 or 24 or 25 |
| 27 | exp animals/ not humans.sh. |
| 28 | 26 not 27 |
| 29 | 17 and 28 |
| 30 | limit 29 to dt=20220101-20240304 |
| 31 | limit 17 to dt=20220101-20240304 |
| 32 | ("37797937" or "36529118" or "38320238" or "35882451" or "37508945" or "36636645" or "36191860" or "37380908" or "37658773" or "37944311" or "37223439" or "36054369" or "37203854" or "36476156" or "36111386" or "37055254" or "38308307" or "38272997" or "37698618" or "37155736" or "38071990" or "37261995" or "37164620" or "38325873" or "37936330" or "37327698" or "38330704" or "37834888" or "36549786" or "38009852" or "37391888" or "37271020" or "37193738" or "38111659" or "36693536" or "36384737" or "35992730" or "36501014" or "36507615" or "35821512").ui. |
| 34 | 32 not 31 |
| 35 | 32 not 30 |
